# Novel 2-phenyloxypyrimidine derivative induces apoptosis and autophagy via inhibiting PI3K pathway and activating MAPK/ERK signaling in hepatocellular carcinoma cells

**DOI:** 10.1038/s41598-018-29199-8

**Published:** 2018-07-19

**Authors:** Jing Wang, Peng Sun, Yijun Chen, Hequan Yao, Shuzhen Wang

**Affiliations:** 1 State Key Laboratory of Natural Medicines (SKLNM) and Laboratory of Chemical Biology, School of Life Science and Technology, China Pharmaceutical University, Nanjing, 210009 China; 2 State Key Laboratory of Natural Medicines (SKLNM) and Department of Medicinal Chemistry, School of Pharmacy, China Pharmaceutical University, Nanjing, 210009 China; 3 Artemisinine Research Center, Institute of Chinese Materia Medica, China Academy of Chinese Medical Sciences, Beijing, 100700 China

**Keywords:** Targeted therapies, Medicinal chemistry

## Abstract

Hepatocellular carcinoma (HCC) is the second leading cause of cancer-related mortality globally. Because most patients are diagnosed at advanced stages of the disease, multi-targeted tyrosine kinase inhibitor sorafenib is the only available drug to show limited effectiveness. Novel and effective therapies are unmet medical need for advanced HCC patients. Given that the aberrant expression and activity of platelet-derived growth factor receptor α (PDGFRα) are closely associated with the pathogenesis of HCC, here we present the discovery and identification of a novel PDGFRα inhibitor, N-(3-((4-(benzofuran-2-yl)pyrimidin-2-yl)oxy)-4-methylphenyl)-4-((4-methylpiperazin-1-yl)methyl)benzamide (E5) after comparison of different derivatives. We found that E5 inhibited proliferation and induced apoptosis in HCC cells. Since the pan-caspase inhibitor Z-VAD-FMK partially rescued HCC cells from E5-reduced cell viability, autophagic cell death triggered by E5 was subsequently investigated. E5 could induce the conversion of LC3-I to LC3-II, increase the expression of Atg5 and restore the autophagy flux blocked by chloroquine. Meanwhile, E5 was able to downregulate the PDGFRα/PI3K/AKT/mTOR pathway and to activate MAPK/ERK signaling pathway. Taken together, in addition to the possibility of E5 as a valuable drug candidate, the present study further supports the notion that targeted inhibition of PDGFRα is a promising therapeutic strategy for HCC.

## Introduction

Hepatocellular carcinoma (HCC) is one of the common aggressive malignancies and the second leading cause of cancer-related deaths worldwide^1,2^. Although there are several different types of treatments currently available for HCC patients, the molecular targeted agent sorafenib, a broad-spectrum tyrosine kinase inhibitor (TKI), is the only US Food and Drug Administration (FDA)-approved drug to show significant survival advantages in late-stage HCC patients^3^. Unfortunately, sorafenib only extends patient survival by approximately 3 months and is not effective to all advanced stage patients from clinical treatment^2^. More seriously, primary and acquired resistances to sorafenib have been reported^4,5^. Therefore, extensive exploration of other novel and effective molecular targeted therapies are urgently needed to enhance current therapy and to provide more treatment options for advanced HCC patients.

Platelet-derived growth factor receptors (PDGFRs), including PDGFRα and PDGFRβ, are cell surface receptors for PDGF and belong to the class III receptor tyrosine kinases (RTKs). In the same class, PDGFRα and PDGFRβ share certain similarity to the stem cell factor receptor (c-KIT), colony stimulating factor 1 receptor (CSF1R) and fms like tyrosine kinase 3 (FLT3)^6^. At molecular level, upon binding to a PDGF dimer, two PDGFR molecules dimerize and activate downstream signaling events, most frequently the phosphatidylinositol 3-kinase (PI3K)/Akt (Protein kinase B, PKB)/mammalian target of rapamycin (mTOR) pathway or mitogenactivated protein kinases/extracellular signal-regulated kinase (MAPK/ERK) pathway in HCC^7,8^. Although high expression of PDGFRβ has been reported in HCC patients, growing evidence has indicated that the aberrant expression and activity of PDGFRα are more closely associated with the pathogenesis of HCC, indicating that the inhibition of PDGFRα may represent a new potential therapeutic strategy for HCC^8,9^.

As the first rationally designed TKI, imatinib has revolutionized the therapy of chronic myeloid leukemia (CML) by inhibiting BCR-ABL1 kinase and gastrointestinal stromal tumors (GISTs) by acting the mutations of c-KIT^10^. Meanwhile, in GIST patients without c-KIT mutations, imatinib also showed activity to inhibit PDGFRa^11^. In a previous attempt to compare different chemotypes, we found that substitution of the 2phenylaminopyrimidine core of imatinib with 2-phenyloxypyrimidine abolished the inhibition to most kinases, while preserved the inhibitory activity to PDGFRα^12^, suggesting that 2-phenyloxypyrimidine core can be explored as a scaffold for the design of next generation of selective PDGFRα inhibitors.

To address PDGFRα as a therapeutic target for HCC and to identify novel PDGFRα inhibitors with better biological function, a 2-phenyloxypyrimidine-based compound library containing 47 derivatives was synthesized and compared in the present study. Among them, compound E5 (N-(3-((4-(benzofuran-2-yl)pyrimidin-2-yl)oxy)-4-methylphenyl)-4-((4-methylpiperazin-1-yl)methyl)benzamide) exhibited potent inhibitory activity both to PDGFRα and to HCC cells. We further investigated its potential mechanism of action, involving G2/M cycle arrest, apoptosis and autophagy. We found that the downregulation of PI3K/AKT/mTOR pathway and the activation of MAPK/ERK signaling are responsible for the cell death induced by E5. Taken together, we identified a novel 2-phenyloxypyrimidine derivative compound E5, which inhibits PDGFRα kinase and induces two forms of cell death including apoptosis and autophagy in HCC cells, generating a basis for the development of new therapeutics for HCC.

## Results

### Design and synthesis of the 2-phenyloxypyrimidine derivatives

**Figure 1:**
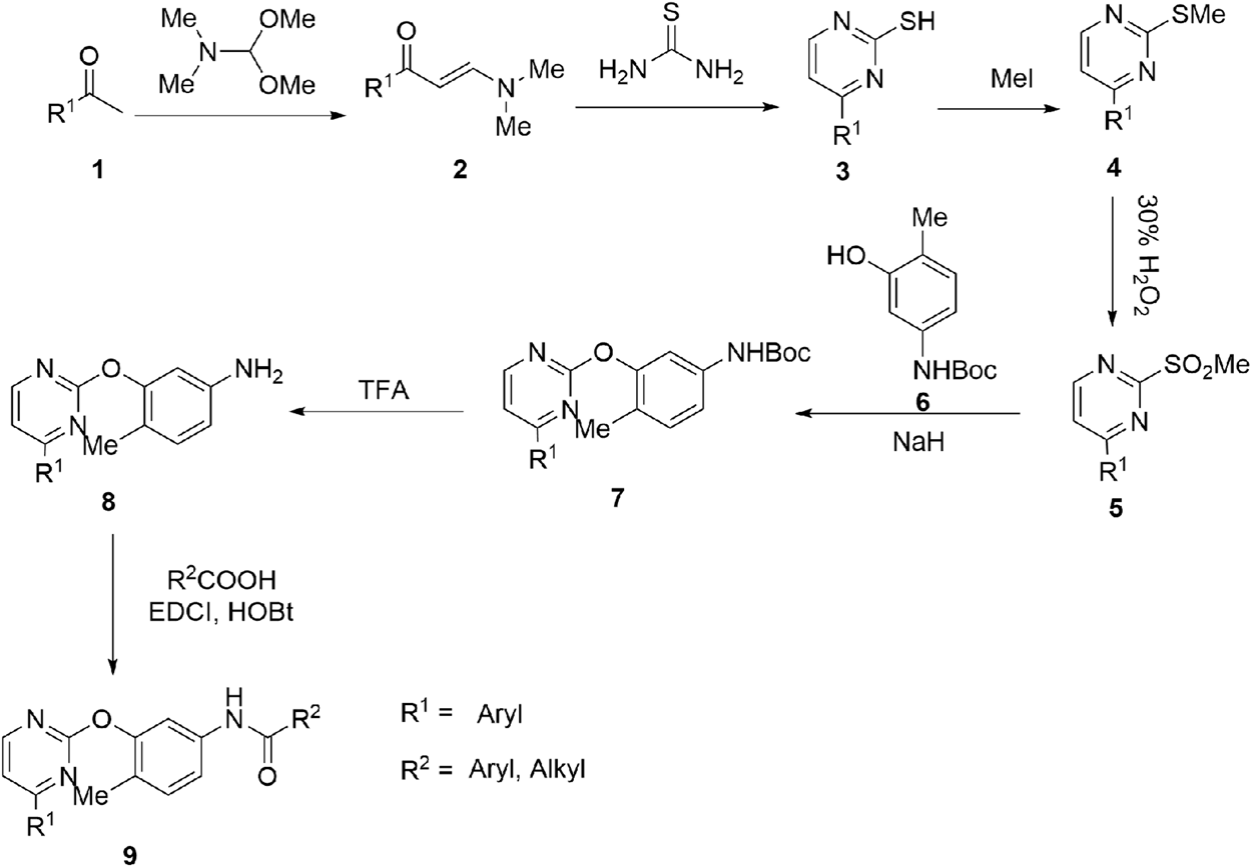
Synthetic scheme of 2-phenyloxypyrimidine derivatives.

In order to design novel 2-phenyloxypyrimidine derivatives with a better PDGFRα inhibitory activity and cellular activity, structural modifications based on 2-phenyloxypyrimidine core were carried out by introducing other functional moieties. After screening the synthetic conditions, 47 new compounds in A-F series (Table 1) were synthesized according to the synthetic route shown in Fig. 1. All compounds were purified by chromatography and structurally characterized by spectroscopic techniques (^1^H NMR, ^13^C NMR and HRMS in Supplementary data).

**Table 1:** Chemical structures of 2-phenyloxypyrimidine derivatives.

	
A1			A7			B1			B7	
A2			A8			B2			B8	
A3			A9			B3			B9	
A4			A10			B4			B10	
A5			A11			B5			B11	
A6			A12			B6			B12	
	
C1	Me		C4			D1			D4	
C2	Et		C5			D2			D5	
C3			C6			D3			D6	
	
E1	Me	E5				F1	Me			
E2	Et	E6				F2	Et			
E3		E7				F3				
E4		E8

### *In vitro* inhibition of 2-phenyloxypyrimidine derivatives on PDGFR

**Figure 2:**
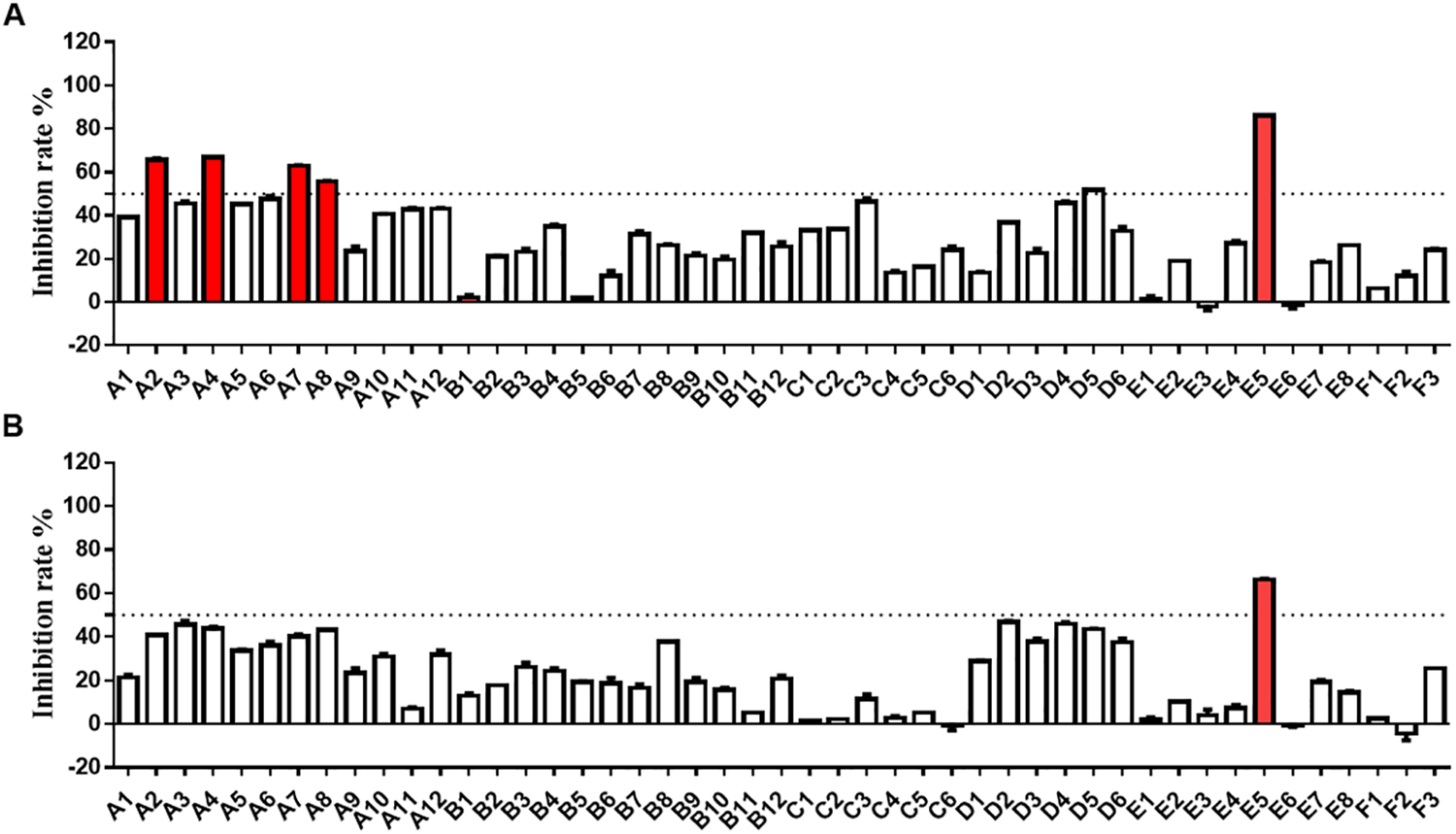
PDGFR kinase inhibition of 2-phenyloxypyrimidine derivatives. (**A**) Inhibition of the compounds at 1 μM against PDGFRα. (**B**) Inhibition of the compounds at 1 μM against PDGFRβ. Both kinase activities were measured using Caliper microfluidic mobility shift technology. Inhibition rate >50% were shown in red. Data shown were averages of two separate experiments.

*In vitro* inhibitory activities of all compounds at 1 μM against PDGFRα and PDGFRβ were evaluated using Caliper microfluidic mobility shift technology^13^. As shown in Fig. 2A, the inhibitory activity on PDGFRα by five compounds, including A2, A4, A7, A8 and E5, at 1 μM was significant, of which E5 gave an inhibition ratio above 85% (Supplementary Table S1). At the same concentration, only E5 inhibited greater than 50% of PDGFRβ activity (Fig. 2B).

### Cellular activity of 2-phenyloxypyrimidine derivatives

The *in vitro* antitumor activity of all compounds was screened against six HCC cell lines by CellTiter-Glo luminescent cell viability assay as described earlier^12^. The results indicated that all compounds exhibited certain degree of inhibition to HCC cells, in which E5 displayed excellent cellular activity as anticipated (Supplementary Table S2). Intriguingly, the cellular activity of a few compounds was not directly proportional to PDGFR kinase inhibition (Fig. 1, Supplementary Table S2), suggesting the existence of a differential intracellular concentration of the compounds and the subtle differential role of PDGFR signaling cascades among different cell lines. After a comprehensive assessment of PDGFR inhibition, cellular activity against HCC cells as well as potential druggability, E5 was chosen to investigate its kinase profiling, antitumor activity and molecular mechanism in the following study.

### Profiling of kinase inhibition by compound E5

To better characterize kinase inhibition, the ability of E5 to inhibit a panel of purified kinases was assessed. The results showed that E5 is a potent inhibitor of class III RTKs. The IC_50_ values of E5 for PDGFRα and c-KIT was 0.40 and 0.51 μM, respectively (Table 2). Compound E5 also showed moderate inhibitory activity to PDGFRβ and CSF1R, while no inhibition to FLT3. Besides class III RTKs, E5 did not show any significant inhibition against other known tyrosine or serine/threonine kinases. Notably, the replacement of 2-aminophenylpyrimidine core of imatinib with 2-phenyloxypyrimidine scaffold almost completely abolishes the inhibition of ABL1 (IC_50_ = 9.35 μM).

**Table 2:** Profiling compound E5 on kinase inhibition.

Kinase	IC_50_ (µM)^a^	Kinase	IC_50_ (µM)^a^
PDGFRα	0.40	FGFR1	>25
PDGFRβ	0.93	FGFR2	>25
c-KIT	0.51	B-raf	>25
CSF1R	1.12	Raf-1	>25
FLT3	>25	EGFR	>25
ABL1	9.35	BLK	>25
ABL2	4.36	LCK	>25

^a^IC_50_: 50% inhibitory concentration (averages of two separate experiments).

### Molecular docking

**Figure 3:**
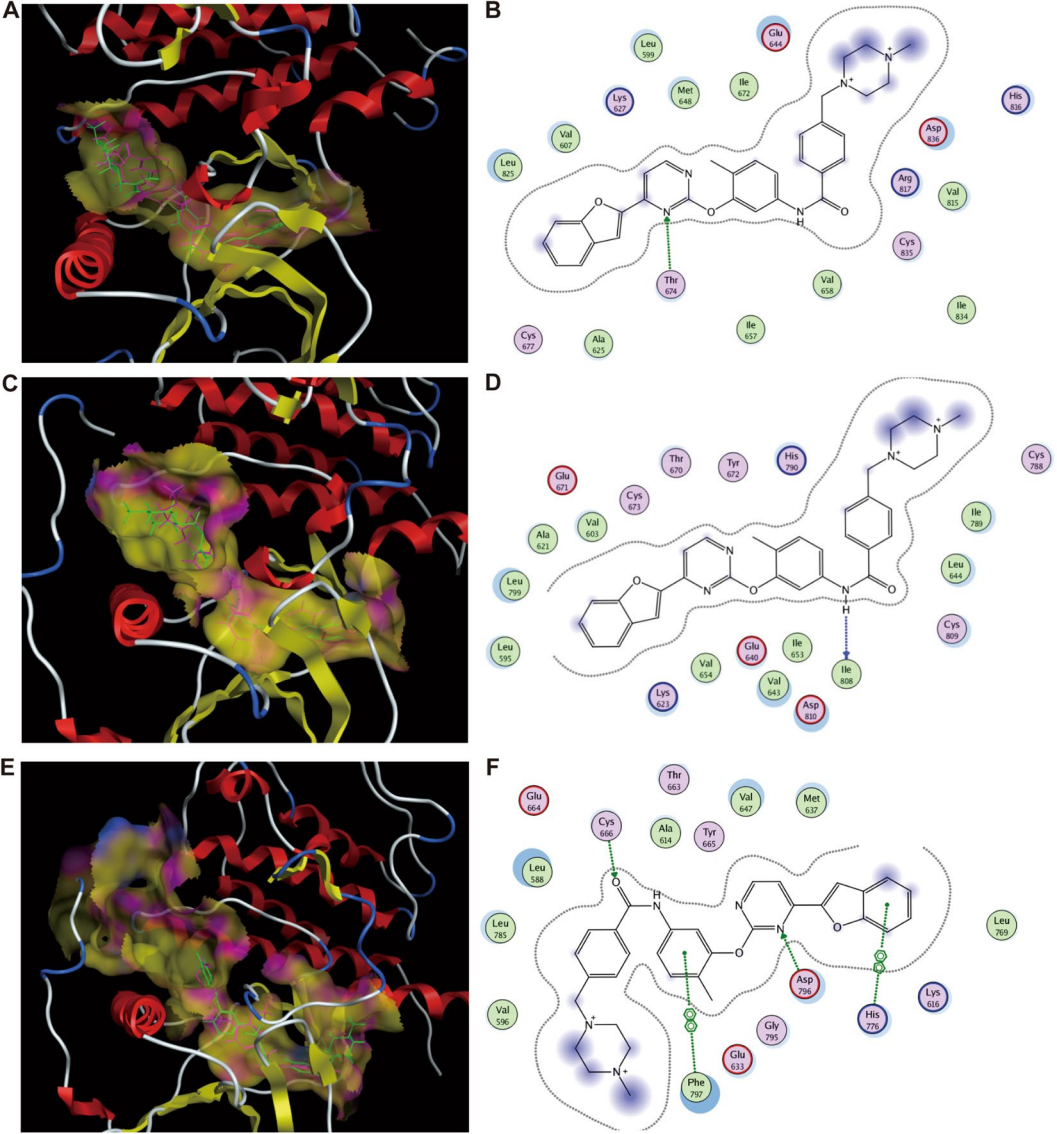
Binding and interactions of compound E5 with PDGFRα, c-KIT and CSF1R. (**A**,**C**,**E**) The superimposition of docked E5 on native ligand co-crystallized with PDGFRα, c-KIT and CSF1R, respectively. The carbons of E5 are colored green while those of native ligand are in purple. (**B**,**D**,**F**) 2-D schematic diagram of docking model of E5 with PDGFRα, c-KIT and CSF1R.

To evaluate the binding modes of E5 with respect to four class III RTKs, molecular docking was performed using software package MOE. Given that the crystal structure of PDGFRβ is currently not available, we only carried out the docking of E5 with PDGFRα, c-KIT and CSF1R. Comparison of docked poses to the original co-crystallized poses indicated that E5 is superimposed very well with native co-crystallized ligand (Fig. 3A,C,E). Compound E5 exhibits 1 hydrogen-bond with residue Thr674 of PDGFRα, which is adjacent to the ATP binding sites Ala625 and Glu675 (Fig. 3B). Compound E5 also possesses 1 hydrogen-bond with residue Ile808 of c-KIT (Fig. 3D), which is very close to conserved Asp810-Phe811-Gly812 (DFG) region in c-KIT. Moreover, E5 could interact simultaneously with two ATP binding sites in CSF1R (Cys666 and Asp796, Fig. 3F). The docking results suggested that E5, possessing a larger side chain with a piperizine ring than all other 2-phenyloxypyrimidine derivatives, potentially serves as a type II multi-kinase inhibitor, which could be able to bind both ATP site and adjacent allosteric site^14^. We speculate that the larger side chain of E5 might help to anchor the entire molecule into an appropriate position for better fitting and occupancy of the binding packet in these kinases.

### Compound E5 inhibits the proliferation of HCC cells

**Figure 4:**
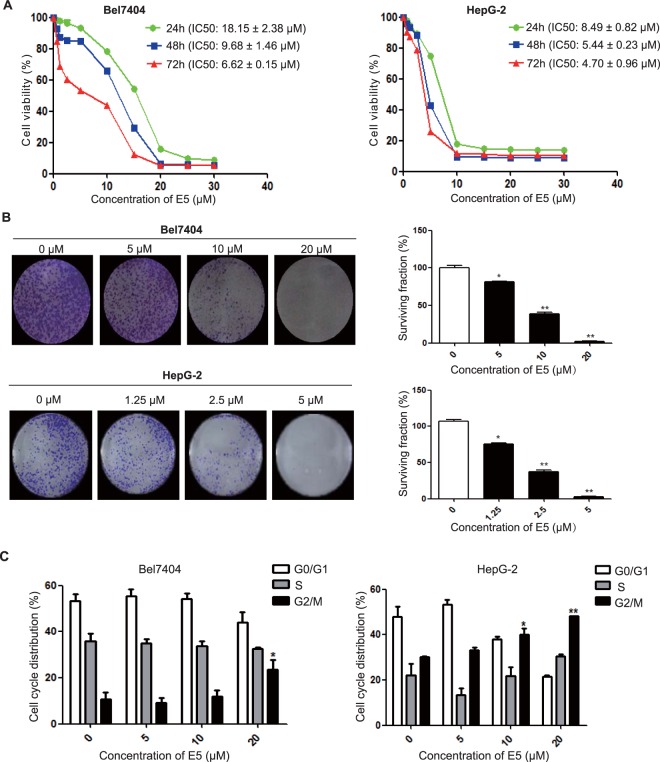
Compound E5 inhibited the proliferation of HCC cells. (**A**) Compound E5 inhibited cell viability of both Bel7404 cells and HepG2 cells. HCC cells were exposed to incremental concentrations of E5 for 24, 48 and 72 h, cell viability was measured by MTT assay. (**B**) Effects of compound E5 on the clonogenicity of Bel7404 and HepG2 cells. Representative images and results from three independent experiments in the graph are shown. (**C**) Compound E5 induced G2/M phase arrest in HCC cells. The distribution of cell cycle was calculated by Mod Fit LT Version 3.0. Bars represented means ± SD of three independent experiments. *P < 0.05; **P < 0.01 compared to DMSO control; one-way ANOVA, *post-hoc* intergroup comparisons, Tukey’s test.

To reconfirm the biological activity, we chose the most sensitive cell line Bel7404 from cellular assay and the widely used and the most characterized cell line HepG2 to examine the effects of E5 on cell proliferation by MTT assay. After treating the cells with E5 at incremental concentrations for 72 hours, MTT assay showed that E5 could markedly inhibit cell viability in a dose- and time-dependent manner with an IC_50_ of 6.62 ± 0.15 μM in Bel7404 cells and 4.72 ± 0.96 μM in HepG2 cells (Fig. 4A). The effects of E5 on colony-forming potential of HCC cells were explored, indicating that E5 significantly inhibited the clonogenicity in both Bel7404 and HepG2 cells (Fig. 4B). Given that cell cycle arrest plays a significant role in inhibiting cancer cell growth, we subsequently examined the effects of E5 on cell cycle distribution of Bel7404 and HepG2 cells. Flow cytometry analysis revealed that treatment with E5 for 24 h elicited G2/M phase arrest in a dose-dependent manner (Fig. 4C).

### Compound E5 induces apoptosis in HCC cells

**Figure 5:**
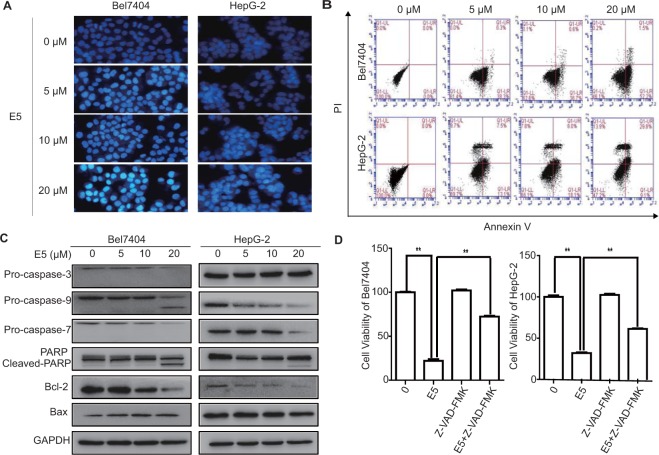
Compound E5 induced apoptosis in HCC cells. (**A**) Apoptotic nuclei manifested condensed or fragmented DNA that were brightly stained by Hoechst 33258 (24 h). Magnification, × 200. (**B**) Flow cytometry analysis after AnnexinV-FITC/PI double staining. Representative histograms for each treatment are shown. (**C**) Western blot analysis of the caspase cascade and apoptosis related proteins after treatment with E5 for 24 h in Bel7404 and HepG2 cells. Original gel images are presented in Supplementary Fig. S1. (**D**) Pan-caspase inhibitor Z-VAD-FMK rescued HCC cells from E5-reduced cell viability. HCC cells were exposed to Z-VAD-FMK (10 μM) with or without E5 (20 μM) for 24 h, cell viability was measured by MTT assay. The data were presented as mean ± SD. *P < 0.05; **P < 0.01 compared to DMSO control; one-way ANOVA, *post-hoc* intergroup comparisons, Tukey’s test.

We next explored the apoptosis-inducing ability of E5 in HCC cells. Bel7404 and HepG2 cells were treated with E5 for 24 h and stained with the fluorescent DNA-binding dye, Hoechst 33258. The cells were brightly stained in a dose-dependent manner, showing a typical morphological sign of apoptosis (Fig. 5A). Analysis of flow cytometry using Annexin V/PI double staining further confirmed that E5 induced apoptosis in a dose-dependent manner (Fig. 5B). Furthermore, E5 induced a specific cleavage of PARP and a decrease of pro-form of caspase-3, caspase-7 and caspase-9 (Fig. 5C). Immunoblotting analysis of apoptosis-related proteins showed that E5 decreased the expression of anti-apoptotic Bcl-2 and increased the expression of pro-apoptotic Bax (Fig. 5C). In addition, the pan-caspase inhibitor Z-VAD-FMK partially rescued the viability of HCC cells reduced by E5, implying that E5 might also induce non-apoptotic form of cell death (Fig. 5D).

### Compound E5 induces autophagy in HCC cells

**Figure 6:**
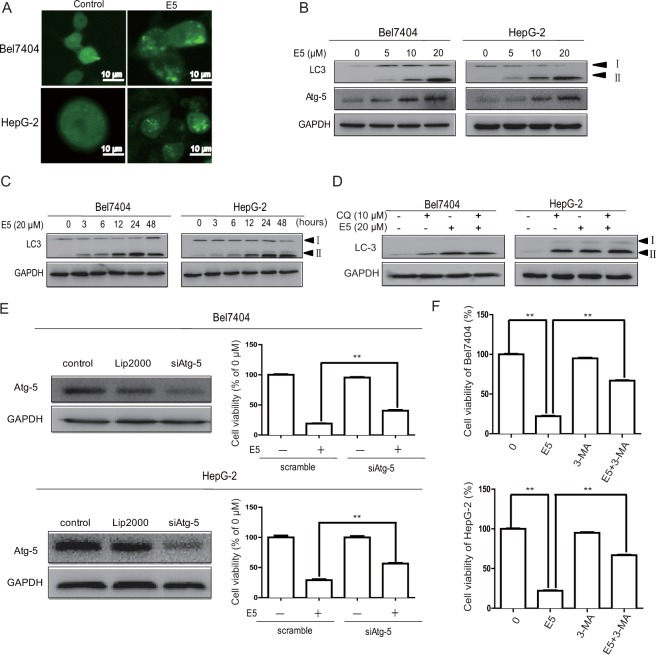
Compound E5 induced autophagy in HCC cells. (**A**) Representative fluorescent images of Bel7404 and HepG2 cells transfected with adenovirus expressing GFP-LC3 fusion protein and treated with E5 (20 μM) for 24 h. The images show GFP-LC3 dots formation in E5-treated cells. (**B**) and (**C**) Western blotting of the expression of LC3-I, LC3-II and Atg5. (**D**) Western blotting of the expression of LC3 after treatment with 20 μM E5 with or without CQ (10 μM) for 24 h in Bel7404 and HepG2 cells. (**E**) Western bloting of siRNA mediated Atg5 knockdown (Left). Knockdown of Atg5 rescued HCC cells from E5-reduced cell viability as assessed by MTT assay (Right). (**F**) Autophagy inhibitor 3-MA rescued HCC cells from E5-reduced cell viability. HCC cells were exposed to 3-MA (2 mM) with or without E5 (20 μM) for 24 h, cell viability was measured by MTT assay. The viability of untreated cells was considered 100%. The data were presented as mean ± SD. *P < 0.05; **P < 0.01 compared to DMSO control; one-way ANOVA, *post-hoc* intergroup comparisons, Tukey’s test. Original gel images are presented in Supplementary Figs S2–S5.

To investigate whether E5 triggers autophagic cell death in HCC cells, several characteristic events of autophagy were examined using different techniques. First, E5 stimulated LC3 puncta formation in HCC cells transfected with adenovirus expressing GFP-LC3 fusion protein as detected by fluorescence microscopy (Fig. 6A). Second, E5 induced the conversion of LC3-I to LC3-II in a dose- and time-dependent manner from Western blots (Fig. 6B,C). Third, the effects of E5 on autophagy flux was examined by co-treatment of E5 with chloroquine (CQ), an inhibitor that prevents the formation of autolysosomes by inhibiting autophagosome-lysosome fusion and LC3-I degradation. The results indicated that E5 could restore the autophagy flux blocked by CQ (Fig. 6D). Fourth, E5 increased the expression of Atg5, which is essential for the elongation of autophagosomes (Fig. 6B), and the cell viability by E5 treatment was increased in HCC cells by knockdown of Atg5 (Fig. 6E). Finally, treatment with 3-methyladenine (3-MA), an autophagy inhibitor, partially reversed E5-induced cell death in HCC cells (Fig. 6F). Together, the experimental evidence strongly indicated that E5 is able to induce autophagy in HCC cells.

### Compound E5 suppressed PDGFRα activation and the downstream PI3K pathway and activated MAPK/ERK pathway

**Figure 7:**
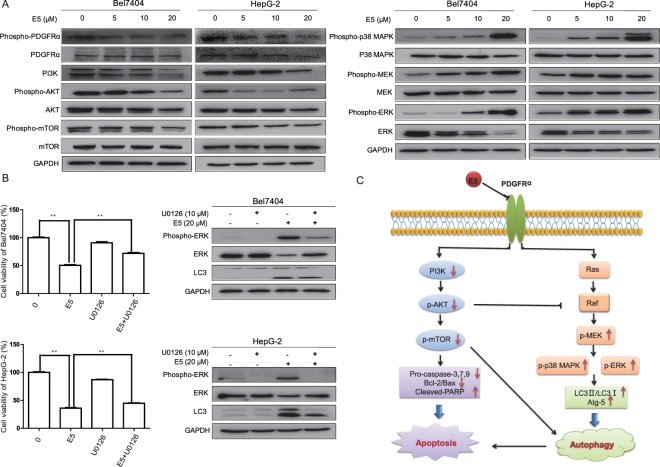
Compound E5 inhibited PDGFRα/PI3K/Akt/mTOR pathway and activateed MAPK/ERK pathway in HCC cells. (**A**) Bel7404 cells and HepG-2 cells were treated with incremental concentrations of E5 for 24 h. The protein levels of PDGFRα, PI3K/AKT/mTOR, MEK/ERK and their phosphorylation status were examined by Western blotting. (**B**) MEK/ERK inhibitor U0126 rescued HCC cells from E5-reduced cell viability and inhibited the conversion of LC3-I to LC3-II. HCC cells were exposed to U0126 (10 μM) with or without E5 (20 μM) for 24 h, cell viability was measured by MTT assay, and the expression of ERK and LC-3 was examined by Western blot. The data were presented as mean ± SD. *P < 0.05; **P < 0.01 compared to DMSO control; one-way ANOVA, *post-hoc* intergroup comparisons, Tukey’s test. Original gel images are presented in Supplementary Figs S6 and S7. (**C**) Schematic illustration of possible pathways involved in the apoptosis and autophagy by compound E5 in HCC cells.

To determine the on-target effects of E5, the expressions of PDGFRα, PDGFRβ, c-KIT and CSF1R of HCC cells were examined by immunoblotting. Indeed, E5 could markedly downregulate the expression of PDGFRα in a dose-dependent manner (Fig. 7A). Given that the basal expression of PDGFRβ and c-KIT was not detectable in HepG2 cells both in our and previous reports and the relationship between CSF1R and HCC was rarely reported^15,16^, we speculated that these protein kinases might play negligible roles in the regulation of cellular signaling mediated by E5.

Because the levels of phospho-PDGFRα and total PDGFRα were decreased from E5 treatment, we postulated that the downstream signaling events would also be suppressed. Since it has been widely recognized that the over-expression of PDGFRα promotes tumorigenesis most frequently through PI3K signaling in human malignancies^17,18^, we next investigated the effects of E5 on PI3K/AKT/mTOR cascade. Western blotting indicated that E5 indeed led to the decrease of protein expressions of PI3K, AKT and mTOR and their endogenous phosphorylated forms in both Bel7404 and HepG2 cells (Fig. 7A).

As another downstream effector of PDGFRα, MAPK/ERK plays a crucial role in different antiproliferative events including apoptosis and autophagy^8^. While ERK activation induced by E5 was observed in our study as shown in Fig. 7A, treatments with E5 elevated the levels of phosphorylated-MEK and phosphorylated-ERK in a dose-dependent manner. Moreover, the presence of MEK/ERK inhibitor U0126 could also partially reverse the cell death induced by E5 and suppress the expression of LC3-II in both Bel7404 and HepG2 cells (Fig. 7B), suggesting that E5-induced autophagy was regulated by MAPK/ERK pathway. Together, these results indicated that the effects of E5 on proliferation, apoptosis and autophagy in HCC cells are mediated through a synergetic effect of inhibiting PDGFRα/PI3K/AKT/mTOR pathway and activating MAPK/ERK pathway (Fig. 7C).

## Discussion

Significant progress in the understanding of cellular and molecular mechanisms of HCC has provided a clear picture that hepatocarcinogenesis depends on aberrant activation of different RTKs, such as epidermal growth factor receptor (EGFR), VEGFR, PDGFR, fibroblast growth factor receptor (FGFR), and hence activate various intracellular signaling pathways^19^. Therefore, small-molecule TKIs have been actively pursued as promising targeted therapeutics during the past years. Among 23 TKIs approved by FDA, only sorafenib, a multi-kinase inhibitor with activity against Raf and several other RTKs, including VEGFR2, PDGFR, FLT3 and c-KIT, has shown survival benefits for advanced HCC patients^1,2,14^. However, the benefits of sorafenib remain modest and resistance to sorafenib has been observed in clinics, suggesting a continued need and opportunity for developing other potent TKIs for HCC^3–5^.

To examine PDGFRα as a target for the therapy of HCC, we synthesized several series of 2-phenyloxypyrimidine derivatives based on our previous finding that substitution of the 2-phenylaminopyrimidine core of imatinib with 2-phenyloxypyrimidine abolished the inhibition to most kinases, while preserved the inhibitory activity against PDGFRα^12^. After evaluation of kinase inhibition and antitumor activity, E5 was identified as the most potent PDGFRα inhibitor with a pharmacological potential against HCC. Therefore, the potential of E5 as a PDGFRα inhibitor and its mechanism of action were further investigated.

It has been shown that many rationally designed kinase inhibitors actually target more than one particular kinase owing to the conserved nature of the ATP-binding pocket^20^, a similar situation applied to E5. In addition to PDGFRα, E5 also showed inhibitory activities against other class III RTKs including PDGFRβ, c-KIT and CSF1R. However, given that the intracellular signaling pathways mediated by these receptors were not found to be major players in the present study, the antitumor activity of E5 is unlikely to closely associate with these receptor kinases. First, similar to previously reported^15,16^, both PDGFRβ and c-KIT receptors were not detectable in HCC cells used in the present study. Second, rare expression of c-KIT but strong PDGFRα activation in HCC patients also implies a more important role of PDGFRα in HCC development^21^. Third, although targeting CSF1R has shown as a novel therapeutic strategy for inflammatory, autoimmune disorders and cancer, such as rheumatoid arthritis and breast cancer, a direct association between CSF1R and HCC development has not been evidenced^22,23^. Taken together, although the present study indicated that the major function of E5 is associated with the inhibition of PDGFRα, we are unable to rule out the possibility of a combined inhibition of multiple kinases by E5, which requires further investigation.

In the present study, the effects of E5 on cell viability of HCC were measured using MTT and colony formation assays. The results showed that E5 significantly inhibited the growth and clonogenicity of HCC cells in both dose- and time-dependent manner. Inhibition of PDGFRα by E5 also led to cell cycle arrest at G2/M phase of HCC cells. Regarding the induction of apoptosis by E5, the apoptotic effects were verified by Hoechst 33258 staining and Annexin V-PI/FITC staining assays. Meanwhile, the caspase inhibitor Z-VAD-FMK partially rescued the cells treated with E5, suggesting that E5 could induce other forms of cell death in addition to apoptosis. Subsequently, autophagic cell death induced by E5 was confirmed by several techniques and partial reversal by the autophagy inhibitor 3-MA, indicating that apoptosis and autophagy are mainly responsible for the cell death by the treatment of E5.

At molecular level, E5 significantly suppressed PDGFRα activation and its downstream PI3K/AKT/mTOR pathway, suggesting that combination of E5 and sorafenib might overcome the development of acquired resistance to sorafenib, in which activation of PI3K/AKT signaling plays a vital role^5^. In addition, we observed that E5 could induce the activation of MAPK/ERK pathway in HCC cells, which seems to be paradoxically contrary to our expectation but is in accordance with the previous conclusion that MAPK/ERK activation in certain conditions can promote cell death^24^. Based on previous reports, cell death induced by numerous stimuli including classic antitumor compounds, such as doxorubicin or cisplatin, requires sustained ERK activation in various cellular and *in vivo* models^24^. However, the mechanism of ERK-mediated cell death is currently not well understood, and conflicting results exist in the literatures, suggesting that it may associate with the upregulation of gene products with death-promoting activity or the regulation of subcellular localization of ERK^24,25^. Therefore, why and how E5 induces ERK activation and the relationship between ERK activation and cell death remain further clarification. Although none of the combinations tested in clinic have truly improved survival compared to sorafenib monotherapy^26^, the synergies of E5 with other therapeutic regimens, such as cytotoxic compounds or monoclonal antibodies against various targets, are currently under investigation in our laboratory. Besides, to assess E5 as a novel lead compound for further development of therapeutics to treat HCC, we are also currently performing a detailed study on its pharmacokinetic, pharmacodynamic and safety study in animals.

In summary, we report a novel 2-phenyloxypyrimidine derivative E5 to induce apoptosis and autophagy *via* the inhibition of PDGFRα/PI3K/AKT/mTOR signaling and the activation of MAPK/ERK pathway in HCC cells, providing further evidence to support the notion that PDGFRα could be a promising therapeutic target for HCC or other PDGFRα-induced diseases and also a new chemotype for class III RTKs inhibition.

## Materials and Methods

### Chemicals, synthesis and characterization

All 2-phenyloxypyrimidine derivatives (A1-A12, B1-B12, C1-C6, D1-D6, E1-E8, F1-F4) were synthesized from the corresponding aryl ketones **1** *via* a synthetic route as shown in Fig. 1. Herein, the synthetic routes to A1 were described in detail as model procedure and other 2-phenyloxypyrimidine derivatives were prepared in the similar way (Supplementary data).

Synthesis of (E)-3-(dimethylamino)-1-(pyridin-3-yl)prop-2-en-1-one (**2**): A round bottom flask was charged with 1-(pyridin-3-yl)ethenone (0.1 mol) and DMF-DMA (1.1 mol), then 100 mL dry DMF was added as solvent, the mixture was heated to reflux for 3 hours. The solvent was removed under reduced pressure, then 100 mL aether was added. Light red precipitate appeared and 18.2 g of **2** was obtained after vacuum suction filtration.

Synthesis of 4-(pyridin-4-yl)pyrimidine-2-thiol (**3**): A round bottom flask was charged with 200 mL EtOH, 2 g sodium was add in portion. After the sodium was totally dissolved, 18.2 g of **2** and 8.3 g of thiourea were added to the flask and the mixture was refluxed for 6 hours. 1000 mL distilled water was added and modulate the PH of the solution to acidity by adding acetic acid. After the solution was cooled to 30 °C, 13 g of **3** was obtained as light yellow solid after vacuum suction filtration.

Synthesis of 2-(methylthio)-4-(pyridin-4-yl)pyrimidine (**4**): 9.4 g of **3** was dissolved in 200 mL 1 N NaOH solution, then 1.2 mol of MeI was added at room temperature, the mixture was stirred for 3 hours. 8.5 g of **4** was obtained as white solid after vacuum suction filtration.

Synthesis of 2-(methylsulfonyl)-4-(pyridin-3-yl)pyrimidine (**5**): A round bottom flask was charged with 6.1 g **4** and 200 mL acetone. The mixture was heated to reflux and 3 equiv. of 70% H_2_O_2_ was added to the solution drop-wise. After refluxing for 6 hours, 200 mL saturated thiosulfate solution was added to the mixture. After extracting the solution with 60 mL dichloromethane for 2 times, the organic layers were combined and dried with Na_2_SO_4_. The solvent was removed under reduced pressure and 5.8 g of **5** was obtained after flash chromatography on silica gel with PE:EA = 10:1(v:v) as eluent.

Synthesis of tert-butyl-(4-methyl-3-((4-(pyridin-3-yl)pyrimidin-2-yl)oxy)phenyl)carbamate (**7**): A round bottom flask was charged with 4.7 g of **5**, 4.5 g of 6 and 100 mL DMF, a suspension liquid of 2.8 g NaH in 50 mL DMF was added to the solution drop-wise at 0 °C. Keeping the reaction temperature at 25 °C for 12 hours, the reaction mixture was dumped to 100 mL ice water, then extracted the solution with 60 mL dichloromethane for 2 times and the organic layers were combined and dried with Na_2_SO_4_. The solvent was removed under reduced pressure and 7.3 g of **6** was obtained after flash chromatography on silica gel with DCM:MeOH = 20:1(v:v) as eluent.

Synthesis of 4-methyl-3-((4-(pyridin-3-yl)pyrimidin-2-yl)oxy)aniline (**8**): A round bottom flask was charged with 7.3 g **6** and 100 mL DCM. 3 equiv TFA was added to the solution and keep stirring at room temperature for 6 hours. The solution was neutralized with saturated sodium bicarbonate solution. After extracting the solution with 60 mL dichloromethane for 2 times, the organic layers were combined and dried with Na_2_SO_4_. The solvent was removed under reduced pressure and 5.8 g of **8** was obtained after flash chromatography on silica gel with PE:EA = 2:1(v:v) as eluent.

Synthesis of 4-methyl-3-((4-(pyridin-3-yl)pyrimidin-2-yl)oxy)aniline (**A1**): A round bottom flask was charged with 0.28 g of **8**, 0.13 g of benzoic acid and 10 mL DCM. 0.15 g HOBt and 0.21 g of EDCI were added to the solution at room temperature. After stirring for 2 hours, 20 mL distilled water was added and the mixture was extracted with 20 mL DCM for two times. The organic layers were combined and washed with water and brine. The solvent was removed under reduced pressure and the residue was purified through flash chromatography on silica gel with DCM:MeOH = 100:1(v:v) as eluent. 0.36 g of **A1** was obtained in the yield of 93%. HPLC R_t_ = 12.7 min, purity: 98%; ^1^H NMR(300 MHz, CDCl_3_) δ 9.14 (s, 1H), 8.63 (s, 1H), 8.50 (d, J = 5.1 Hz, 1H), 8.37–8.19 (m, 2H), 8.01 (d, J = 7.4 Hz, 1H), 7.73 (d, J = 7.3 Hz, 2H), 7.59 (s, 1H), 7.48–7.25 (m, 7H), 7.16 (t, J = 7.5 Hz, 1H), 2.09 (s, 3H) ppm; ^13^C NMR(75 MHz, CDCl_3_) δ 164.8, 164.0, 160.1, 159.7, 151.0, 147.7, 136.6, 134.8, 134.4, 132.4, 131.2, 130.9, 129.4, 128.2, 127.8, 126.6, 126.1, 123.5, 117.1, 113.6, 111.2, 15.5 ppm; HRMS (ESI) calcd. for [C_23_H_18_N_4_O_2_ + H]^+^ 383.1430, found 383.1508.

### Antibodies and reagents

Antibodies against PDGFRα (#5241), Caspase-3 (#9665), PARP (#9542), Caspase-9 (#9508), Bax (#2772), Bcl-2 (#2872), PI3K (#4257), AKT (#4691), phospho-AKT (#4060), mTOR (#2983), phospho-mTOR (#5536), p38-MAPK (#8690), phospho-p38MAPK (#4511), MEK (#9126), phospho-MEK (#9154), ERK1/2 (#4695), phospho-ERK1/2 (#4370), GAPDH (#2118) were purchased from Cell Signaling Technology (Danvers, MA, USA). Antibody to LC3 (#AL221) was purchased from Beyotime Institute of Biotechnology (Shanghai, China). Antibody to Atg-5 (#ab108327) was obtained from Abcam (Cambridge, MA, USA). Z-VAD-FMK, chloroquine, 3-Methyladenine, U0126-EtOH were from Selleck Chemicals (Houston, TX, USA).

### Cell culture

Liver cancer cell lines of Bel7404 and HepG2 were obtained from American Type Culture Collection (ATCC) and kept by Nanjing KeyGen Biotech. Co. Ltd. (Nanjing, China). Bel7404 cells were cultured in RPMI 1640 medium and HepG2 cells in DMEM medium. All media contained 10% fetal bovine serum (FBS) (Bioind, US origin) and all cells were incubated in a humidified atmosphere of 5% CO_2_ at 37 °C. All cell lines were authenticated by using short tandem repeat (STR) matching analysis. No mycoplasma contamination was detected.

### *In vitro* kinase assay

PDGFR kinase activity was measured using Caliper microfluidic mobility shift technology^27^. Briefly, 2 μL compound solution (25 μM) in 25% DMSO per well were added into the 384 well microtiter plates. Then, 10 μL per well of enzyme solution (100 mM HEPES, 10 mM MgCl2, Brij35 (30%) 100 μL, 1 mM DTT, 20 ng enzyme) was added. After a brief centrifugation at 1,000 rpm, the kinase reactions were started by addition of 38 μL per well of peptide/ATP-solution (40 μM ATP, 6.5 μM peptide 5′-FAM-KKKKEEIYFFF-NH2, Tris-HCl buffer, pH 7.0) incubated at 30 °C for 60 min and stopped by the addition of 20 μL 35 mM EDTA per well. The electrophoretic separation of the phosphorylated and non-phosphorylated peptides was then carried out on LabChip EZ Reader II (Caliper Life Sciences). All other kinase reactions were carried out as previously reported using the Kinase-Glo Plus or ADP-Glo assay^12^. Kinase inhibition rates were calculated per manufacturer’s instructions.

### Molecular docking

Molecular docking was performed using Molecular Operating Environment (MOE) software version 2008.10 (Chemical Computing Group, Montreal, Canada) according to reported process^28^. The X-ray crystallographic complex of PDGFRA (PDB ID: 5GRN), c-KIT (PDB ID: 1T46) and CSF1R (PDB ID: 3LCO) with respective bound ligand were used as templates. All water molecules in PDB files were removed and hydrogen atoms were subsequently added to each protein structure. The 3-D structure of E5 was built using the builder interface of MOE program and energy minimized using MMFF94x force field. Then, E5 was docked into the active site of each protein and the best 5 poses of the molecules were retained and scored.

### MTT assay

Cell viability was measured by methyl thiazolyl tetrazolium (MTT) assay. Cells were seeded in 96-well plate at a density of 1 × 10^4^ cells/well. After overnight incubation, solution of E5 in DMSO with incremental concentrations (0–30 μM) were added to the cells and further incubated for 3 days. During this period, three wells were selected from each group of cells every day for the MTT (50 μg/well) assay. After the cells had been incubated at 37 °C for 4 h, the reaction was stopped by adding 150 μL/well of DMSO, and incubating for 10 min. The absorbance was measured at a wavelength of 490 nm by a Microplate Reader (BioTek, USA).

### Flow cytometry analysis

Cells were incubated with E5 for 24 h, after which cells were washed, stained and subjected to flow cytometry analysis. The cell cycle was analyzed by staining with propidium iodide (PI), and the extent of cell death/apoptosis was evaluated by double staining with annexin V-FITC and PI. The fluorescence was measured with a FACScan flow cytometer (Becton Dickinson, USA).

### Colony formation assay

Cells (300/well) were seeded in 24-well plates and incubated overnight in a humidified atmosphere of 5% CO_2_ at 37 °C. Incremental concentrations of E5 (0–20 μM) were then added, and the medium was replaced with fresh culture medium containing 10% FBS after 24 h. After two weeks of culture, colonies were fixed with methanol, stained with 1% crystal violet and colonies containing more than 50 cells were counted.

### Hoechst 33258 staining

Cells were treated with incremental concentrations of E5 (0–20 μM) for 24 h and washed with ice-cold PBS twice, then fixed with 4% paraformaldehyde for 15 min. Then, cells were stained with Hoechst 33258 (1 μg/mL) for 15 min in the dark and examined by fluorescence microscope.

### Ad-GFP-LC3 transfection

Cells were seeded in 6-well plates and reached 30% confluence at the time of transfection. After a wash with fresh culture medium, cells were transfected with Ad-GFP-LC3 adenovirus at a MOI of 20 in 1 mL culture medium for 24 h at 37 °C. Then, culture supernatant was replaced with fresh culture medium containing 10% FBS. Following the treatment by E5 for 24 h, autophagy was evaluated under laser scanning confocal microscope (FV1000 Olympus).

### siRNA transfection

Human Atg5 siRNA (sense, 5′-UUGGUAACUGACAAAGUGAAAAAdTdT-3′; antisense, 5′-UUUUUCACUUUGUCAGUUACCAAdTdT-3′) was synthesized by Biomics Biotech (Nantong, China) and transfected followed the manufacturer’s instructions. Briefly, cells were plated in 6-well plates at 5 × 10^5^ cells/well and cultured overnight. Transfection was performed by adding 5 μL liposome 2000 reagent and 5 μL siRNA (final concentration 50 nM). After transfection for 48 h, the efficiency of siRNA-induced gene silencing was assessed by Western blots. Cell viability was determined by MTT assay as described above.

### Western blotting

After treatment with E5 for 24 h, cells were lysed in RIPA cell lysis buffer. Equal amount of protein (30–40 μg) was separated on a 12% or 15% SDS-PAGE and transferred electrophoretically onto a 0.45 μm PVDF membrane. Then, the membrane was blocked for 2 h at room temperature with 5% non-fat dried milk in TBS buffer and incubated with appropriate antibodies at 1:1000 (overnight, 4 °C). After washing, the membrane was incubated with HRP-conjugated secondary antibody (1:10000) for 2 h at room temperature and visualized using ECL Western blot detection reagents (Millipore, USA).

### Statistical analysis

Each experiment was repeated for more than 3 times. Results were expressed as the mean ± standard deviation (SD). Statistical analysis of multiple-group comparisons was performed by one-way analysis of variance (ANOVA) followed by the Bonferroni *post hoc* test using SPSS software. Comparisons between 2 groups were analyzed using 2-tailed Student *t*-tests. A *P* value < 0.05 or <0.01 was considered statistically significant.

### Data availability statement

All data generated or analyzed during this study are included in this published article (and its Supplementary Information files).

## Electronic supplementary material

Supplementary information
