# Author Correction: Novel 2-phenyloxypyrimidine derivative induces apoptosis and autophagy via inhibiting PI3K pathway and activating MAPK/ERK signaling in hepatocellular carcinoma cells

**DOI:** 10.1038/s41598-019-56287-0

**Published:** 2019-12-19

**Authors:** Jing Wang, Peng Sun, Yijun Chen, Hequan Yao, Shuzhen Wang

**Affiliations:** 1 State Key Laboratory of Natural Medicines (SKLNM) and Laboratory of Chemical Biology, School of Life Science and Technology, China Pharmaceutical University, Nanjing, 210009 China; 2 State Key Laboratory of Natural Medicines (SKLNM) and Department of Medicinal Chemistry, School of Pharmacy, China Pharmaceutical University, Nanjing, 210009 China; 3 Artemisinine Research Center, Institute of Chinese Materia Medica, China Academy of Chinese Medical Sciences, Beijing, 100700 China

Correction to: *Scientific Reports* 10.1038/s41598-018-29199-8, published online 19 July 2018

In Figure 6F, the figure for ‘Cell viability of HepG-2 (%)’ is incorrect. The correct Figure 6 appears below as Figure 1.

**Figure 1:**
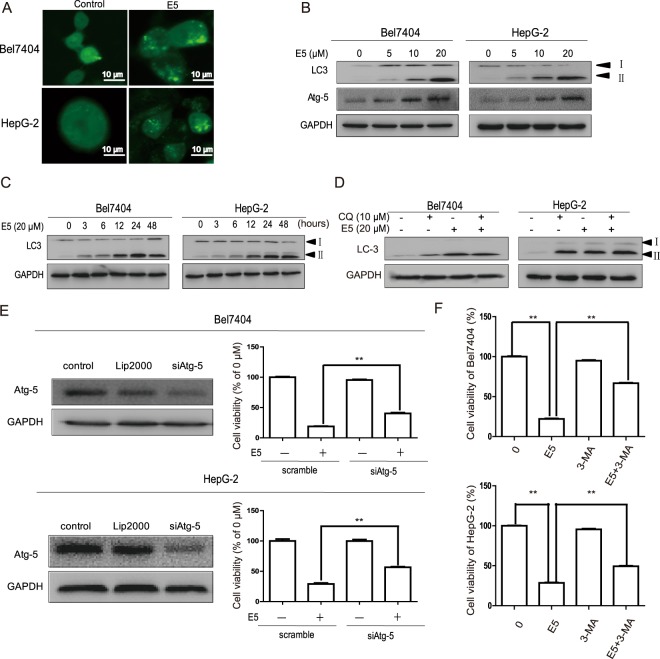
.

