# Supplementary material for: Novel 2-phenyloxypyrimidine derivative induces apoptosis and autophagy via inhibiting PI3K pathway and activating MAPK/ERK signaling in hepatocellular carcinoma cells
Source: Sci Rep. 2018 Jul 19;8:10923. doi: 10.1038/s41598-018-29199-8 (PMC6053381; doi:10.1038/s41598-018-29199-8)

Supplementary information

Novel 2-phenyloxypyrimidine derivative induces apoptosis and autophagy via inhibiting PI3K pathway and activating MAPK/ERK signaling in hepatocellular carcinoma cells

Jing Wang [1, +], Peng Sun [2, +], Yijun Chen [1*,] Hequan Yao [2, *], Shuzhen Wang [1*]

Table of contents

Materials & Methods………………………………………………………. 2-26

Supplementary Table S1 PDGFR kinase inhibition of 2-phenyloxypyrimidine derivatives………………………………………………...………….… 27-28

Supplementary Table S2 Anti-proliferative activity of 2-phenyloxypyrimidine derivatives on HCC cell lines determined by CellTiter-Glo luminescent cell viability assay (IC_{50}, μM)……………………………………………….……..... 29-30

Supplementary Figure S1 Original gel images for Manuscript Fig. 5C…..…. 31-33

Supplementary Figure S2 Original gel images for Manuscript Fig. 6B…..…. 34-35

Supplementary Figure S3 Original gel images for Manuscript Fig. 6C…..…. 36

Supplementary Figure S4 Original gel images for Manuscript Fig. 6D…..…. 37

Supplementary Figure S5 Original gel images for Manuscript Fig. 6E…..…. 38

Supplementary Figure S6 Original gel images for Manuscript Fig. 7A…..…. 39-44

Supplementary Figure S7 Original gel images for Manuscript Fig. 7B….…... 45-46

Supplementary NMR and HRMS data for all compounds 47-186

Materials & Methods

Chemical synthesis

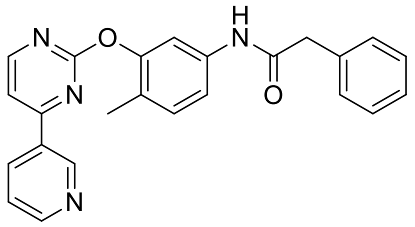

A2

N-(4-methyl-3-((4-(pyridin-3-yl)pyrimidin-2-yl)oxy)phenyl)-2-phenylacetamide: A round bottom flask was charged with 0.28 g of **8**, 0.13 g of benzoic acid and 10 mL DCM. 0.15 g HOBt and 0.21 g of EDCI were added to the solution at room temperature. After stirring for 2 hours, 20 mL distilled water was added and the mixture was extracted with 20 mL DCM for two times. The organic layers were combined and washed with water and brine. The solvent was removed under reduced pressure and the residue was purified through flash chromatography on silica gel with DCM:MeOH = 100:1(v:v) as eluent. 0.38 g of **A2** was obtained in the yield of 96%. HPLC R_t = 13.3 min, purity: 99%; ^{1}H NMR(300 MHz, $CDCl_3$) δ 9.18 (d, J = 1.8 Hz, 1H), 8.69 – 8.68 (m, 1H), 8.32 (d, J = 8.0 Hz, 1H), 7.99 (s, 1H), 7.51 (s, 1H), 7.42 – 7.38 (m, 2H), 7.36 – 7.27 (m, 5H), 7.25 – 7.16 (m, 2H), 3.64 (s, 2H), 2.12 (s, 3H) ppm; ^{13}C NMR(75 MHz, $CDCl_3$) δ 169.2, 164.0, 165.3, 164.6, 160.6, 151.9, 151.3, 148.5, 136.9, 134.9, 134.5, 131.8, 131.2, 127.5, 126.4, 123.8, 117.0, 113.6, 111.6, 44.6, 15.9 ppm; HRMS (ESI) calcd for $[C_{24}H_{20}N_4O_2+H]^+$ 397.1586, found 397.1465.

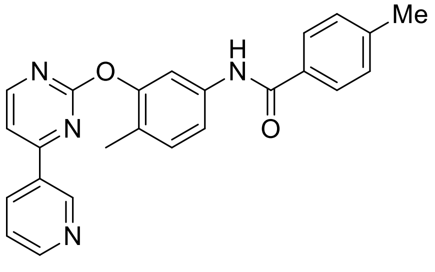

A3

N-(4-methyl-3-((4-(pyridin-3-yl)pyrimidin-2-yl)oxy)phenyl)-2-phenylacetamide: A round bottom flask was charged with 0.28 g of **8**, 0.13 g of 4-methylbenzoic acid and 10 mL DCM. 0.15 g HOBt and 0.21 g of EDCI were added to the solution at room temperature. After stirring for 2 hours, 20 mL distilled water was added and the mixture was extracted with 20 mL DCM for two times. The organic layers were combined and washed with water and brine. The solvent was removed under reduced pressure and the residue was purified through flash chromatography on silica gel with DCM:MeOH = 100:1(v:v) as eluent. 0.33 g of **A3** was obtained in the yield of 85%. HPLC R_t = 11.8 min, purity: 99%; ^{1}H NMR(300 MHz, $CDCl_3$) δ 9.20 (s, 1H), 8.70 (d, J = 3.4 Hz, 1H), 8.68 – 8.58 (m, 1H), 8.37 – 8.34 (m, 1H), 7.98 (d, J = 8.1 Hz 1H), 7.73 – 7.67 (m, 3H), 7.45 – 7.40 (m, 3H), 7.39 – 7.18 (m, 3H), 2.36 (s, 3H), 2.17 (s, 3H) ppm; ^{13}C NMR(75 MHz, $CDCl_3$) δ 165.6, 165.4, 164.6, 160.6, 151.8, 151.4, 148.4, 142.3, 137.2, 135.0, 132.0, 131.8, 130.0, 129.4, 129.0, 127.0, 126.4, 123.9,

117.4, 114.0, 111.6, 21.5, 16.0 ppm; HRMS (ESI) calcd for [$C_{24}H_{20}N_4O_2$+H]+ 397.1586, found 397.1665.

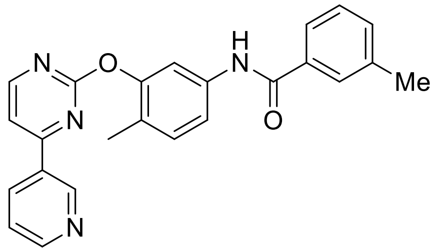

A4

3-methyl-N-(4-methyl-3-((4-(pyridin-3-yl)pyrimidin-2-yl)oxy)phenyl)benzamide: A round bottom flask was charged with 0.28 g of **8**, 0.13 g of 3-methylbenzoic acid and 10 mL DCM. 0.15 g HOBt and 0.21 g of EDCI were added to the solution at room temperature. After stirring for 2 hours, 20 mL distilled water was added and the mixture was extracted with 20 mL DCM for two times. The organic layers were combined and washed with water and brine. The solvent was removed under reduced pressure and the residue was purified through flash chromatography on silica gel with DCM:MeOH = 100:1(v:v) as eluent. 0.35 g of **A4** was obtained in the yield of 90%. HPLC R_t = 12.0 min, purity: 99%; ^{1}H NMR(300 MHz, $CDCl_3$) δ 9.12 (d, J = 1.2 Hz 1H), 8.61 (d, J = 3.5 Hz, 1H), 8.50 (d, J = 5.2 Hz, 1H), 8.27 (d, J = 8.0 Hz, 1H), 7.98 (d, J = 8.1 Hz 1H), 7.73 – 7.67 (m, 1H), 7.45 – 7.40 (m, 1H), 8.21 (s, 1H), 7.60 – 7.50 (s, 3H), 7.37 – 7.30 (m, 3H), 7.20 – 7.14 (m, 3H), 2.26 (s, 3H), 2.09 (s, 3H) ppm; ^{13}C NMR(75 MHz, $CDCl_3$) δ 166.2, 165.3, 164.5, 160.6, 151.7, 151.4, 148.4, 138.4, 137.4, 135.0, 134.8, 132.4, 131.8, 131.3, 128.4, 127.9, 126.3, 124.2, 123.8, 117.6, 114.1, 111.6, 21.2, 15.9 ppm; HRMS (ESI) calcd for [$C_{24}H_{20}N_4O_2$+H]+ 397.1586, found 397.1664.

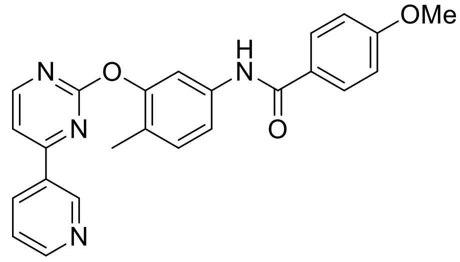

A5

4-methoxy-N-(4-methyl-3-((4-(pyridin-3-yl)pyrimidin-2-yl)oxy)phenyl)benzamide: A round bottom flask was charged with 0.28 g of **8**, 0.17 g of 4-methoxybenzoic acid and 10 mL DCM. 0.15 g HOBt and 0.21 g of EDCI were added to the solution at room temperature. After stirring for 2 hours, 20 mL distilled water was added and the mixture was extracted with 20 mL DCM for two times. The organic layers were combined and washed with water and brine. The solvent was removed under reduced pressure and the residue was purified through flash chromatography on silica gel with DCM:MeOH = 100:1(v:v) as eluent. 0.37 g of **A5** was obtained in the yield of 90%. HPLC R_t = 14.2 min, purity: 99%; ^{1}H NMR(300 MHz, $CDCl_3$) δ 9.12 (d, J = 1.2 Hz 1H), 8.61 (d, J = 3.5 Hz, 1H), 8.50 (d, J = 5.2 Hz, 1H), 8.27 (d, J = 8.0 Hz, 1H), 7.98 (d, J = 8.1 Hz 1H), 7.73 – 7.67 (m, 1H), 7.45 – 7.40 (m, 1H), 8.21 (s, 1H), 7.60 – 7.50 (s, 3H), 7.37 – 7.30 (m, 3H), 7.20 – 7.14 (m, 3H), 2.26 (s, 3H), 2.09 (s, 3H) ppm; ^{13}C

NMR(75 MHz, $CDCl_3$) δ 165.4, 164.7, 162.4, 160.6, 151.9, 151.4, 148.5, 137.3, 134.9, 131.8, 129.0, 127.0, 126.3, 123.8, 117.5, 114.0, 113.8, 111.6, 55.4, 16.0 ppm; HRMS (ESI) calcd for $[C_{24}H_{20}N_4O_3+H]^+$ 413.1535, found 413.1614.

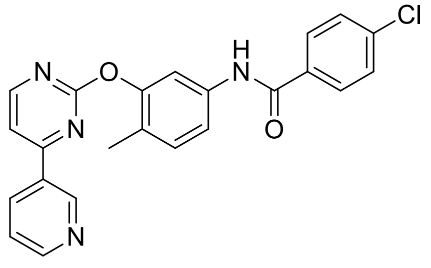

A6

4-chloro-N-(4-methyl-3-((4-(pyridin-3-yl)pyrimidin-2-yl)oxy)phenyl)benzamide: A round bottom flask was charged with 0.28 g of **8**, 0.17 g of 4-chlorobenzoic acid and 10 mL DCM. 0.15 g HOBt and 0.21 g of EDCI were added to the solution at room temperature. After stirring for 2 hours, 20 mL distilled water was added and the mixture was extracted with 20 mL DCM for two times. The organic layers were combined and washed with water and brine. The solvent was removed under reduced pressure and the residue was purified through flash chromatography on silica gel with DCM:MeOH = 100:1(v:v) as eluent. 0.36 g of **A6** was obtained in the yield of 87%. HPLC R_t = 13.7 min, purity: 99%; ^{1}H NMR (300 MHz, $CDCl_3$) δ 9.18 (s, 1H), 8.68 (d, J = 3.5 Hz, 1H), 8.57– 8.54 (m, 2H), 8.32 (d, J = 8.0 Hz, 1H), 7.74 (d, J = 8.5 Hz, 2H), 7.65 (s, 1H), 7.44 – 7.31 (m, 5H), 7.22 (d, J = 8.0 Hz, 1H), 2.16 (s, 3H) ppm; ^{13}C NMR (75 MHz, $CDCl_3$) δ 165.3, 164.9, 160.6, 151.9, 151.4, 148.5, 137.9, 137.0, 134.9, 133.2, 131.7, 131.4, 128.8, 128.6, 126.8, 123.8, 117.6, 114.2, 111.7, 16.0 ppm; HRMS (ESI) calcd for $[C_{23}H_{17}N_4O_2Cl+H]^+$ 417.1040, found 417.1119.

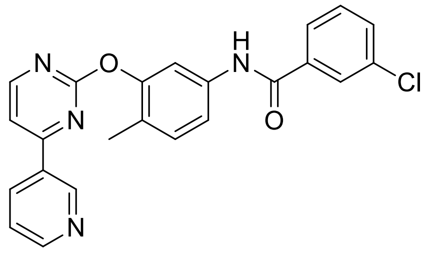

A7

3-chloro-N-(4-methyl-3-((4-(pyridin-3-yl)pyrimidin-2-yl)oxy)phenyl)benzamide: A round bottom flask was charged with 0.28 g of **8**, 0.17 g of 3-chlorobenzoic acid and 10 mL DCM. 0.15 g HOBt and 0.21 g of EDCI were added to the solution at room temperature. After stirring for 2 hours, 20 mL distilled water was added and the mixture was extracted with 20 mL DCM for two times. The organic layers were combined and washed with water and brine. The solvent was removed under reduced pressure and the residue was purified through flash chromatography on silica gel with DCM:MeOH = 100:1(v:v) as eluent. 0.38 g of **A7** was obtained in the yield of 91%. HPLC R_t = 12.5 min, purity: 99%; ^{1}H NMR (300 MHz, $CDCl_3$) δ 9.17 (s, 1H), 8.80 (s, 1H), 8.66 (d, J = 4.5 Hz, 1H), 8.55 (d, J = 5.1 Hz, 1H), 8.32 (d, J = 7.9 Hz, 2H), 7.78 (s, 1H), 7.67 (d, J = 7.9 Hz, 2H), 7.44 – 7.31 (m, 4H), 7.28 – 7.30 (m, 1H), 7.20 (d, J = 8.2 Hz, 1H), 2.14 (s, 3H) ppm; ^{13}C NMR (75 MHz, $CDCl_3$) δ 165.3, 164.7, 164.4, 160.6, 152.0, 151.4, 148.6, 136.8, 136.7, 134.9, 134.8, 131.8, 131.4, 130.0, 127.4,

126.9, 125.2, 123.8, 117.6, 114.2, 111.7, 16.0 ppm; HRMS (ESI) calcd for $[C_{23}H_{17}N_4O_2Cl+H]^+$ 417.1040, found 417.1119.

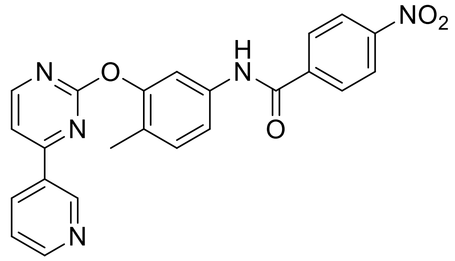

A8

N-(4-methyl-3-((4-(pyridin-3-yl)pyrimidin-2-yl)oxy)phenyl)-4-nitrobenzamide: A round bottom flask was charged with 0.28 g of **8**, 0.18 g of 4-nitrobenzoic acid and 10 mL DCM. 0.15 g HOBt and 0.21 g of EDCI were added to the solution at room temperature. After stirring for 2 hours, 20 mL distilled water was added and the mixture was extracted with 20 mL DCM for two times. The organic layers were combined and washed with water and brine. The solvent was removed under reduced pressure and the residue was purified through flash chromatography on silica gel with DCM:MeOH = 50:1(v:v) as eluent. 0.32 g of **A8** was obtained in the yield of 75%. HPLC R_t = 9.8 min, purity: 99%; ^1H NMR (300 MHz, $CDCl_3$) δ 9.22 (d, J = 1.7 Hz, 1H), 8.72 (dd, J = 4.8 Hz, J = 1.5 Hz, 1H), 8.33 (dt, J = 8.0 Hz, J = 1.7 Hz, 1H), 8.30 (d, J = 8.6 Hz, 2H), 8.14 (s, 1H), 8.01 (d, J = 8.7 Hz, 2H), 7.67 (s, 1H), 7.49 (d, J = 5.2 Hz, 1H), 7.46 – 7.39 (m, 2H), 7.30 (d, J = 8.3 Hz, 1H), 2.21 (s, 3H) ppm; ^{13}C NMR (75 MHz, $CDCl_3$) δ 165.2, 164.6, 163.8, 160.6, 151.8, 151.4, 149.6, 148.5, 140.4, 136.4, 134.9, 131.7, 131.5, 128.4, 127.5, 123.9, 117.7, 114.4, 111.7, 16.0 ppm; HRMS (ESI) calcd for $[C_{23}H_{17}N_5O_4+H]^+$ 427.1281, found 427.1407.

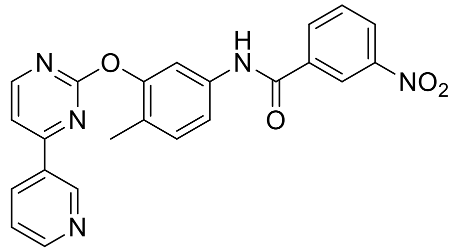

A9

N-(4-methyl-3-((4-(pyridin-3-yl)pyrimidin-2-yl)oxy)phenyl)-3-nitrobenzamide: A round bottom flask was charged with 0.28 g of **8**, 0.18 g of 3-nitrobenzoic acid and 10 mL DCM. 0.15 g HOBt and 0.21 g of EDCI were added to the solution at room temperature. After stirring for 2 hours, 20 mL distilled water was added and the mixture was extracted with 20 mL DCM for two times. The organic layers were combined and washed with water and brine. The solvent was removed under reduced pressure and the residue was purified through flash chromatography on silica gel with DCM:MeOH = 50:1(v:v) as eluent. 0.33 g of **A9** was obtained in the yield of 77%. HPLC R_t = 8.1 min, purity: 99%; ^1H NMR (300 MHz, $CDCl_3$) δ 9.27 (s, 1H), 9.16 (d, J = 1.5 Hz, 1H), 8.67 – 8.66 (m, 2H), 8.57 (d, J = 5.2Hz, 1H), 8.32 – 8.20 (m, 3H), 7.65 (d, J = 1.3 Hz, 1H), 7.57 (d, J = 8.0 Hz, 1H), 7.46 – 7.37 (m, 3H), 7.20 (d, J = 8.3 Hz, 1H), 2.13 (s, 3H) ppm; ^{13}C NMR (75 MHz, $CDCl_3$) δ 165.2, 164.6, 163.6, 160.6, 151.9, 151.4, 148.5, 148.0, 136.6, 136.4, 134.9, 133.6, 131.7, 131.4, 129.9, 127.2,

126.2, 123.9, 122.0, 117.9, 114.5, 111.7, 16.0 ppm; HRMS (ESI) calcd for [$C_{23}H_{17}N_5O_4$+H]+ 427.1281, found 427.1405.

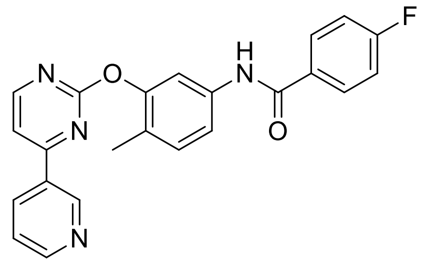

A10

4-fluoro-N-(4-methyl-3-((4-(pyridin-3-yl)pyrimidin-2-yl)oxy)phenyl)benzamide: A round bottom flask was charged with 0.28 g of **8**, 0.15 g of 4-fluorobenzoic acid and 10 mL DCM. 0.15 g HOBt and 0.21 g of EDCI were added to the solution at room temperature. After stirring for 2 hours, 20 mL distilled water was added and the mixture was extracted with 20 mL DCM for two times. The organic layers were combined and washed with water and brine. The solvent was removed under reduced pressure and the residue was purified through flash chromatography on silica gel with DCM:MeOH = 50:1(v:v) as eluent. 0.38 g of **A10** was obtained in the yield of 95%. HPLC R_t = 11.9 min, purity: 98%; ^{1}H NMR (300 MHz, $CDCl_3$) δ 9.11 (s, 1H), 8.60 (d, J = 3.9 Hz, 1H), 8.48 (d, J = 5.0 Hz, 1H), 8.38 (s, 1H), 8.25 (d, J = 7.8 Hz, 1H), 7.75 (dd, J = 7.8, 5.5 Hz, 2H), 7.56 (s, 1H), 7.33 (dd, J = 14.9, 6.5 Hz, 3H), 7.14 (d, J = 8.2 Hz, 1H), 6.96 (t, J = 8.4 Hz, 2H), 2.08 (s, 3H) ppm; ^{13}C NMR (75 MHz, $CDCl_3$) δ 166.4, 165.3, 164.6, 160.6, 151.8, 151.4, 148.5, 137.0, 134.9, 131.8, 131.4, 129.6, 129.5, 126.7, 123.8, 117.6, 115.8, 115.5, 114.2, 111.6, 15.9 ppm; HRMS (ESI) calcd for [$C_{23}H_{17}N_4O_2F$+H]+ 401.1336, found 401.1414.

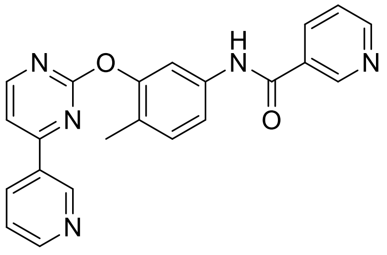

A11

N-(4-methyl-3-((4-(pyridin-3-yl)pyrimidin-2-yl)oxy)phenyl)nicotinamide: A round bottom flask was charged with 0.28 g of **8**, 0.13 g of nicotinic acid and 10 mL DCM. 0.15 g HOBt and 0.21 g of EDCI were added to the solution at room temperature. After stirring for 2 hours, 20 mL distilled water was added and the mixture was extracted with 20 mL DCM for two times. The organic layers were combined and washed with water and brine. The solvent was removed under reduced pressure and the residue was purified through flash chromatography on silica gel with DCM:MeOH = 30:1(v:v) as eluent. 0.34 g of **A11** was obtained in the yield of 89%. HPLC R_t = 7.6 min, purity: 99%; ^{1}H NMR (300 MHz, $CDCl_3$) δ 9.19 (s, 1H), 8.76 (s, 1H), 8.69 (s, 1H), 8.59 (d, J = 5.1 Hz, 1H), 8.33 (d, J = 8.0 Hz, 1H), 7.68 – 7.65 (m, 3H), 7.47 – 7.40 (m, 3H), 7.26 (d, J = 8.4 Hz, 1H), 2.18 (s, 3H) ppm; ^{13}C NMR (75 MHz, $CDCl_3$) δ 165.3, 164.7, 163.9, 160.6, 152.0, 151.4, 150.5, 148.6, 142.1, 136.5, 134.8, 131.7, 131.5, 127.4, 123.8, 121.0, 117.7, 114.4, 111.7, 16.0 ppm; HRMS (ESI)

calcd for $[C_{22}H_{17}N_5O_2+H]+$ 384.1382, found 384.1463.

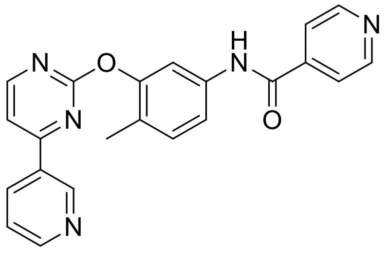

A12

N-(4-methyl-3-((4-(pyridin-3-yl)pyrimidin-2-yl)oxy)phenyl)isonicotinamide: A round bottom flask was charged with 0.28 g of **8**, 0.13 g of isonicotinic acid and 10 mL DCM. 0.15 g HOBt and 0.21 g of EDCI were added to the solution at room temperature. After stirring for 2 hours, 20 mL distilled water was added and the mixture was extracted with 20 mL DCM for two times. The organic layers were combined and washed with water and brine. The solvent was removed under reduced pressure and the residue was purified through flash chromatography on silica gel with DCM:MeOH = 30:1(v:v) as eluent. 0.31 g of **A12** was obtained in the yield of 82%. HPLC R_t = 8.3 min, purity: 99%; ^{1}H NMR (300 MHz, $CDCl_3$) 9.20 (s, 1H), 9.08 (d, J = 1.4 Hz, 1H), 8.96 (s, 1H), 8.66 – 8.50 (m, 2H), 8.47 (d, J = 5.2 Hz, 1H), 8.27 – 8.16 (m, 1H), 8.07 (d, J = 8.0 Hz, 1H), 7.57 (d, J = 1.5 Hz, 1H), 7.41 – 7.26 (m, 3H), 7.22 (dd, J = 7.5, 4.6 Hz, 1H), 7.12 (d, J = 8.3 Hz, 1H), 2.06 (s, 3H) ppm; ^{13}C NMR (75 MHz, $CDCl_3$) δ 165.2, 164.6, 164.1, 160.6, 152.0, 151.8, 151.3, 148.4, 148.0, 136.9, 135.6, 134.9, 131.7, 131.4, 130.9, 127.0, 123.9, 123.6, 117.9, 114.4, 111.7, 15.9 ppm; HRMS (ESI) calcd for $[C_{22}H_{17}N_5O_2+H]+$ 384.1382, found 384.1459.

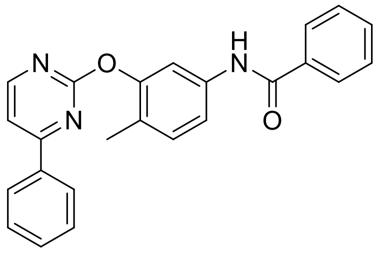

B1

N-(4-methyl-3-((4-phenylpyrimidin-2-yl)oxy)phenyl)benzamide: A round bottom flask was charged with 0.28 g **8 (4-methyl-3-((4-phenylpyrimidin-2-yl)oxy)aniline)**, 0.13 g benzoic acid and 10 mL DCM. 0.15 g HOBt and 0.21 g of EDCI were added to the solution at room temperature. After stirring for 2 hours, 20 mL distilled water was added and the mixture was extracted with 20 mL DCM for two times. The organic layers were combined and washed with water and brine. The solvent was removed under reduced pressure and the residue was purified through flash chromatography on silica gel with DCM:MeOH = 150:1(v:v) as eluent. 0.33 g of **B1** was obtained in the yield of 86%. HPLC R_t = 15.1 min, purity: 99%; ^{1}H NMR (300 MHz, $CDCl_3$) 8.85 (s, 1H), 8.37 (d, J = 5.2 Hz, 1H), 7.96 (d, J = 6.3 Hz, 2H), 7.74 (d, J = 7.3 Hz, 2H), 7.61 (s, 1H), 7.51 (d, J = 6.9 Hz, 1H), 7.41 – 7.30 (m, 5H), 7.24 (t, J = 7.6 Hz, 2H), 7.13 (d, J = 8.3 Hz, 1H), 2.09 (s, 3H) ppm; ^{13}C NMR (75 MHz, $CDCl_3$) δ 167.2, 166.3, 165.2, 159.8, 151.4, 137.4, 135.8, 134.8, 131.6, 131.4, 131.3, 128.9, 128.5, 127.4, 127.2, 126.5, 117.8, 114.3, 111.7, 16.0 ppm; HRMS (ESI) calcd for $[C_{24}H_{19}N_3O_2+H]+$

382.1477, found 382.1554.

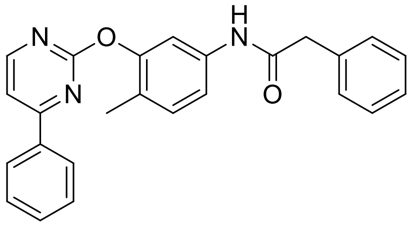

B2

N-(4-methyl-3-((4-phenylpyrimidin-2-yl)oxy)phenyl)-2-phenylacetamide: A round bottom flask was charged with 0.28 g **8** **(4-methyl-3-((4-phenylpyrimidin-2-yl)oxy)aniline)**, 0.15 g 2-phenylacetic acid and 10 mL DCM. 0.15 g HOBt and 0.21 g of EDCI were added to the solution at room temperature. After stirring for 2 hours, 20 mL distilled water was added and the mixture was extracted with 20 mL DCM for two times. The organic layers were combined and washed with water and brine. The solvent was removed under reduced pressure and the residue was purified through flash chromatography on silica gel with DCM:MeOH = 150:1(v:v) as eluent. 0.37 g of **B2** was obtained in the yield of 94%. HPLC R_t = 16.9 min, purity: 99%; ^{1}H NMR (300 MHz, $CDCl_3$) δ 8.42 (d, J = 5.1 Hz, 1H), 7.97 (d, J = 6.5 Hz, 2H), 7.41 (d, J = 6.5 Hz, 4H), 7.34 (d, J = 5.1 Hz, 1H), 7.29 – 7.21 (m, 6H), 7.11 (d, J = 8.1 Hz, 1H), 3.61 (s, 2H), 2.09 (s, 3H) ppm; ^{13}C NMR (75 MHz, $CDCl_3$) δ 169.0, 167.2, 165.3, 160.0, 151.500, 136.708, 136.0, 134.5, 131.4, 131.2, 129.5, 129.1, 128.9, 127.6, 127.4, 126.6, 117.0, 113.6, 111.6, 44.7, 16.0 ppm; HRMS (ESI) calcd for $[C_{25}H_{21}N_3O_2+H]+$ 396.1634, found 396.1711.

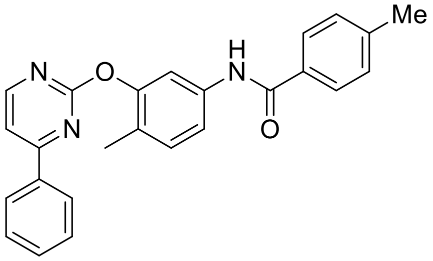

B3

4-methyl-N-(4-methyl-3-((4-phenylpyrimidin-2-yl)oxy)phenyl)benzamide: A round bottom flask was charged with 0.28 g **8** **(4-methyl-3-((4-phenylpyrimidin-2-yl)oxy)aniline)**, 0.13 g of 4-methylbenzoic acid and 10 mL DCM. 0.15 g HOBt and 0.21 g of EDCI were added to the solution at room temperature. After stirring for 2 hours, 20 mL distilled water was added and the mixture was extracted with 20 mL DCM for two times. The organic layers were combined and washed with water and brine. The solvent was removed under reduced pressure and the residue was purified through flash chromatography on silica gel with DCM:MeOH = 150:1(v:v) as eluent. 0.36 g of **B3** was obtained in the yield of 91%. HPLC R_t = 14.6 min, purity: 99%; ^{1}H NMR (300 MHz, $CDCl_3$) δ 8.48 (d, J = 5.2 Hz, 1H), 8.22 (s, 1H), 8.03 – 8.00 (m, 2H), 7.62 – 7.56 (m, 3H), 7.51 – 7.38 (m, 5H), 7.27 – 7.20 (m, 3H), 2.33 (s, 3H), 2.15 (s, 3H) ppm; ^{13}C NMR (75 MHz, $CDCl_3$) δ 167.2, 166.0, 165.3, 159.9, 151.5, 138.5, 137.2, 135.9, 134.9, 134.0, 132.5, 131.4, 131.3, 130.6, 128.9, 128.3, 127.4, 127.2, 126.6, 124.1, 117.5, 114.0, 111.6, 21.3, 16.0 ppm; HRMS (ESI) calcd for $[C_{25}H_{21}N_3O_2+H]+$ 396.1634, found 396.1714.

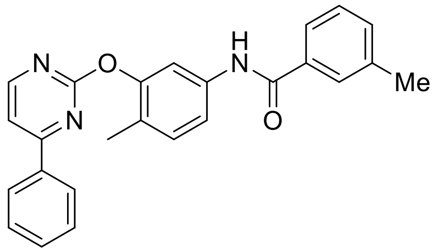

B4

3-methyl-N-(4-methyl-3-((4-phenylpyrimidin-2-yl)oxy)phenyl)benzamide: A round bottom flask was charged with 0.28 g **8** **(4-methyl-3-((4-phenylpyrimidin-2-yl)oxy)aniline)**, 0.13 g of 4-methylbenzoic acid and 10 mL DCM. 0.15 g HOBt and 0.21 g of EDCI were added to the solution at room temperature. After stirring for 2 hours, 20 mL distilled water was added and the mixture was extracted with 20 mL DCM for two times. The organic layers were combined and washed with water and brine. The solvent was removed under reduced pressure and the residue was purified through flash chromatography on silica gel with DCM:MeOH = 150:1(v:v) as eluent. 0.32 g of **B4** was obtained in the yield of 81%. HPLC R_t = 15.8 min, purity: 99%; ^{1}H NMR (300 MHz, $CDCl_3$) δ 8.42 (d, J = 5.1 Hz, 1H), 7.97 – 7.94 (m, 3H), 7.64 (d, J = 8.1 Hz, 2H), 7.47 (d, J = 1.6 Hz, 1H), 7.41 (s, 1H), 7.40 – 7.32 (m, 5H), 7.18– 7.12 (m, 3H), 2.29 (s, 3H), 2.09 (s, 3H) ppm; ^{13}C NMR (75 MHz, $CDCl_3$) δ 167.2, 165.3, 160.0, 142.3, 151.6, 142.3, 137.1, 136.0, 131.4, 128.9, 127.0, 117.4, 113.9, 111.6, 21.3, 16.0 ppm; HRMS (ESI) calcd for $[C_{25}H_{21}N_3O_2+H]^+$ 396.1634, found 396.1713.

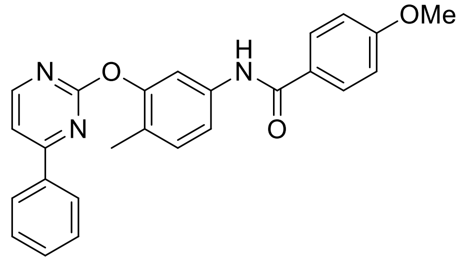

B5

4-methoxy-N-(4-methyl-3-((4-phenylpyrimidin-2-yl)oxy)phenyl)benzamide: A round bottom flask was charged with 0.28 g **8** **(4-methyl-3-((4-phenylpyrimidin-2-yl)oxy)aniline)**, 0.16 g of 4-methoxybenzoic acid and 10 mL DCM. 0.15 g HOBt and 0.21 g of EDCI were added to the solution at room temperature. After stirring for 2 hours, 20 mL distilled water was added and the mixture was extracted with 20 mL DCM for two times. The organic layers were combined and washed with water and brine. The solvent was removed under reduced pressure and the residue was purified through flash chromatography on silica gel with DCM:MeOH = 150:1(v:v) as eluent. 0.35 g of **B5** was obtained in the yield of 86%. HPLC R_t = 14.1 min, purity: 99%; ^{1}H NMR (300 MHz, $CDCl_3$) δ 8.43 (d, J = 5.2 Hz, 1H), 8.37 (s, 1H), 8.00 (d, J = 7.7Hz, 2H), 7.76 (d, J = 8.7Hz, 2H), 7.55 (s, 1H), 7.49 – 7.34 (m, 5H), 7.17 (d, J = 8.2 Hz, 1H), 6.81 (d, J = 8.7 Hz, 3H), 3.75 (s, 3H), 2.13 (s, 3H) ppm; ^{13}C NMR (75 MHz, $CDCl_3$) δ 167.1, 165.5, 165.3, 159.9, 151.5, 137.4, 135.9, 131.4, 131.3, 129.0, 128.9, 127.3, 127.0, 126.3, 117.6, 114.1, 113.8, 111.6, 55.4, 16.0 ppm; HRMS (ESI) calcd for $[C_{25}H_{21}N_3O_3+H]^+$ 412.1583, found

412.1662.

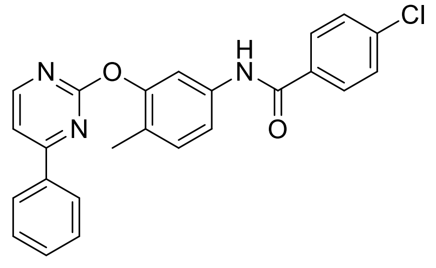

B6

4-chloro-N-(4-methyl-3-((4-phenylpyrimidin-2-yl)oxy)phenyl)benzamide: A round bottom flask was charged with 0.28 g **8** **(4-methyl-3-((4-phenylpyrimidin-2-yl)oxy)aniline)**, 0.16 g of 4-chlorobenzoic acid and 10 mL DCM. 0.15 g HOBt and 0.21 g of EDCI were added to the solution at room temperature. After stirring for 2 hours, 20 mL distilled water was added and the mixture was extracted with 20 mL DCM for two times. The organic layers were combined and washed with water and brine. The solvent was removed under reduced pressure and the residue was purified through flash chromatography on silica gel with DCM:MeOH = 150:1(v:v) as eluent. 0.37 g of **B6** was obtained in the yield of 89%. HPLC R_t = 11.7 min, purity: 97%; ^{1}H NMR (300 MHz, $CDCl_3$) δ 8.53 (d, J = 5.2 Hz, 1H), 8.04 (dd, J = 7.3, 1.5Hz, 2H), 7.88 (s, 1H), 7.77 (d, J = 8.5 Hz, 2H), 7.53 – 7.41 (m, 8H), 7.29 – 7.26 (m, 1H), 2.19 (s, 3H) ppm; ^{13}C NMR (75 MHz, $CDCl_3$) δ 167.2, 165.2, 164.6, 159.9, 151.6, 138.0, 136.8, 135.9, 133.3, 131.4, 128.9, 128.5, 127.3, 127.0, 117.5, 114.1, 111.7, 16.0 ppm; HRMS (ESI) calcd for $[C_{24}H_{18}N_3O_2Cl+H]^+$ 416.1088, found 416.1665.

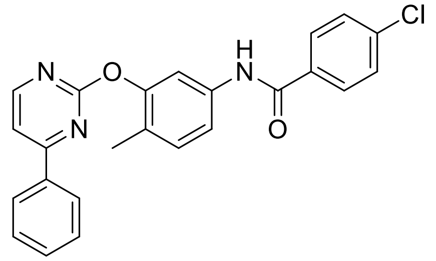

B7

3-chloro-N-(4-methyl-3-((4-phenylpyrimidin-2-yl)oxy)phenyl)benzamide: A round bottom flask was charged with 0.28 g **8** **(4-methyl-3-((4-phenylpyrimidin-2-yl)oxy)aniline)**, 0.16 g of 3-chlorobenzoic acid and 10 mL DCM. 0.15 g HOBt and 0.21 g of EDCI were added to the solution at room temperature. After stirring for 2 hours, 20 mL distilled water was added and the mixture was extracted with 20 mL DCM for two times. The organic layers were combined and washed with water and brine. The solvent was removed under reduced pressure and the residue was purified through flash chromatography on silica gel with DCM:MeOH = 150:1(v:v) as eluent. 0.35 g of **B7** was obtained in the yield of 84%. HPLC R_t = 13.6 min, purity: 99%; ^{1}H NMR (300 MHz, $CDCl_3$) δ 8.91 (s, 1H), 8.39 (d, J = 5.2 Hz, 1H), 7.96 (d, J = 6.6 Hz, 2H), 7.71 (s, 1H), 7.61 – 7.48 (m, 3H), 7.43 – 7.34 (m, 5H), 7.18 (d, J = 8.0 Hz, 1H), 7.13 (d, J = 8.0 Hz, 1H), 2.08 (s, 3H) ppm; ^{13}C NMR (75 MHz, $CDCl_3$) δ 167.2, 165.1, 164.9, 159.8, 151.4, 137.0, 136.6, 135.8, 134.5, 131.5, 131.3, 129.8, 127.5, 127.3, 126.8, 125.4, 117.9, 114.4, 111.8, 16.0 ppm;

HRMS (ESI) calcd for $[C_{24}H_{18}N_3O_2Cl+H]^+$ 416.1088, found 416.1165.

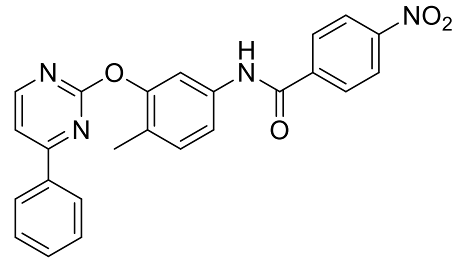

B8

N-(4-methyl-3-((4-phenylpyrimidin-2-yl)oxy)phenyl)-4-nitrobenzamide: A round bottom flask was charged with 0.28 g **8** **(4-methyl-3-((4-phenylpyrimidin-2-yl)oxy)aniline)**, 0.16 g of 4-nitrobenzoic acid and 10 mL DCM. 0.15 g HOBt and 0.21 g of EDCI were added to the solution at room temperature. After stirring for 2 hours, 20 mL distilled water was added and the mixture was extracted with 20 mL DCM for two times. The organic layers were combined and washed with water and brine. The solvent was removed under reduced pressure and the residue was purified through flash chromatography on silica gel with DCM:MeOH = 100:1(v:v) as eluent. 0.33 g of **B8** was obtained in the yield of 77%. HPLC R_t = 11.8 min, purity: 99%; ^{1}H NMR (300 MHz, $CDCl_3$) δ 8.55 (d, J = 5.1 Hz, 1H), 8.33 (d, J = 8.7 Hz, 2H), 8.14 – 7.93 (m, 4H), 7.84 (s, 1H), 7.56 (s, 1H), 7.50 – 7.44 (m, 4H), 7.32 (d, J = 8.2 Hz, 1H), 2.22 (s, 3H) ppm; ^{13}C NMR (75 MHz, $CDCl_3$) δ 167.3, 165.1, 163.9, 159.9, 151.5, 149.5, 140.4, 136.4, 135.7, 131.5, 129.0, 128.4, 127.3, 123.8, 117.8, 114.3, 111.8, 16.0 ppm; HRMS (ESI) calcd for $[C_{24}H_{18}N_4O_4+H]^+$ 427.1328, found 427.1403.

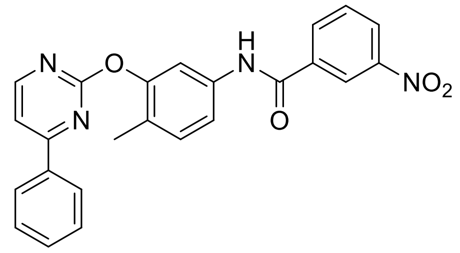

B9

N-(4-methyl-3-((4-phenylpyrimidin-2-yl)oxy)phenyl)-3-nitrobenzamide: A round bottom flask was charged with 0.28 g **8** **(4-methyl-3-((4-phenylpyrimidin-2-yl)oxy)aniline)**, 0.16 g of 3-nitrobenzoic acid and 10 mL DCM. 0.15 g HOBt and 0.21 g of EDCI were added to the solution at room temperature. After stirring for 2 hours, 20 mL distilled water was added and the mixture was extracted with 20 mL DCM for two times. The organic layers were combined and washed with water and brine. The solvent was removed under reduced pressure and the residue was purified through flash chromatography on silica gel with DCM:MeOH = 100:1(v:v) as eluent. 0.31 g of **B9** was obtained in the yield of 73%. HPLC R_t = 12.6 min, purity: 99%; ^{1}H NMR (300 MHz, $CDCl_3$) δ 8.65 (t, J = 1.7 Hz, 1H), 8.58 (s, 1H), 8.49 (d, J = 5.2 Hz, 1H), 8.35 – 8.24 (m, 1H), 8.19 (d, J = 7.9 Hz, 1H), 7.99 (dd, J = 7.9, 1.6 Hz, 2H), 7.59 (t, J = 8.0 Hz, 1H), 7.55 – 7.49 (m, 2H), 7.45 – 7.35 (m, 4H), 7.24 (dd, J = 9.7, 5.3 Hz, 1H), 2.14 (s, 3H). ppm; ^{13}C NMR (75 MHz, $CDCl_3$) δ 167.3, 165.1, 163.5, 159.8, 151.4, 148.0, 136.5, 135.7, 133.5, 131.5, 129.9,

128.9, 127.3, 126.2, 122.0, 117.8, 114.4, 111.8, 16.0 ppm; HRMS (ESI) calcd for $[C_{24}H_{18}N_4O_4+H]^+$ 427.1328, found 427.1407.

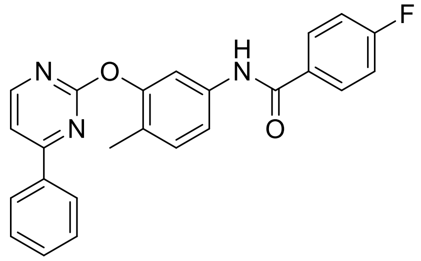

B10

4-fluoro-N-(4-methyl-3-((4-phenylpyrimidin-2-yl)oxy)phenyl)benzamide: A round bottom flask was charged with 0.28 g **8** **(4-methyl-3-((4-phenylpyrimidin-2-yl)oxy)aniline)**, 0.15 g of 4-fluorobenzoic acid and 10 mL DCM. 0.15 g HOBt and 0.21 g of EDCI were added to the solution at room temperature. After stirring for 2 hours, 20 mL distilled water was added and the mixture was extracted with 20 mL DCM for two times. The organic layers were combined and washed with water and brine. The solvent was removed under reduced pressure and the residue was purified through flash chromatography on silica gel with DCM:MeOH = 100:1(v:v) as eluent. 0.32 g of **B10** was obtained in the yield of 80%. HPLC R_t = 10.6 min, purity: 99%; ^{1}H NMR (300 MHz, $CDCl_3$) δ 8.52 (d, J = 5.2 Hz, 1H), 8.04 (dd, J = 7.5, 1.7 Hz, 2H), 7.91 (s, 1H), 7.84 (dd, J = 8.7, 5.3 Hz, 2H), 7.58 – 7.37 (m, 6H), 7.27 (d, J = 6.3 Hz, 1H), 7.12 (t, J = 8.6 Hz, 2H), 2.19 (s, 3H) ppm; ^{13}C NMR (75 MHz, $CDCl_3$) δ 167.2, 166.5, 160.0, 151.6, 136.8, 136.0, 131.4, 129.4, 129.3, 128.9, 127.3, 127.0, 117.4, 115.9, 115.6, 114.1, 111.6, 16.0 ppm; HRMS (ESI) calcd for $[C_{24}H_{18}N_3O_2F+H]^+$ 400.1383, found 400.1463.

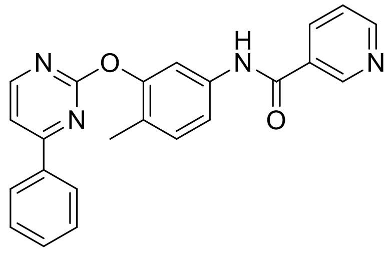

B11

N-(4-methyl-3-((4-phenylpyrimidin-2-yl)oxy)phenyl)nicotinamide: A round bottom flask was charged with 0.28 g **8** **(4-methyl-3-((4-phenylpyrimidin-2-yl)oxy)aniline)**, 0.13 g of nicotinic acid and 10 mL DCM. 0.15 g HOBt and 0.21 g of EDCI were added to the solution at room temperature. After stirring for 2 hours, 20 mL distilled water was added and the mixture was extracted with 20 mL DCM for two times. The organic layers were combined and washed with water and brine. The solvent was removed under reduced pressure and the residue was purified through flash chromatography on silica gel with DCM:MeOH = 50:1(v:v) as eluent. 0.34 g of **B11** was obtained in the yield of 89%. HPLC R_t = 10.9 min, purity: 99%; ^{1}H NMR (300 MHz, $CDCl_3$) δ 9.38 (s, 1H), 8.55 (d, J = 4.3 Hz, 2H), 8.43 (d, J = 5.1 Hz, 1H), 7.98 (d, J = 6.9 Hz, 2H), 7.61 – 7.50 (m, 4H), 7.46 – 7.36 (m, 4H), 7.19 (d, J = 8.2 Hz, 1H), 2.13 (s, 3H) ppm; ^{13}C NMR (75 MHz, $CDCl_3$) δ 167.2, 165.1, 164.2, 159.9, 151.5, 150.2, 142.2, 136.7, 135.7, 131.5,

131.4, 128.9, 127.3, 121.3, 117.9, 114.4, 111.8, 16.0 ppm; HRMS (ESI) calcd for $[C_{23}H_{18}N_4O_2+H]^+$ 383.1430, found 383.1506.

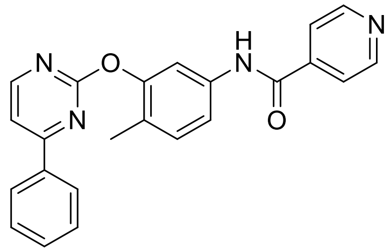

B12

N-(4-methyl-3-((4-phenylpyrimidin-2-yl)oxy)phenyl)isonicotinamide: A round bottom flask was charged with 0.28 g **8** **(4-methyl-3-((4-phenylpyrimidin-2-yl)oxy)aniline)**, 0.13 g of isonicotinic acid and 10 mL DCM. 0.15 g HOBt and 0.21 g of EDCI were added to the solution at room temperature. After stirring for 2 hours, 20 mL distilled water was added and the mixture was extracted with 20 mL DCM for two times. The organic layers were combined and washed with water and brine. The solvent was removed under reduced pressure and the residue was purified through flash chromatography on silica gel with DCM:MeOH = 50:1(v:v) as eluent. 0.34 g of **B12** was obtained in the yield of 89%. HPLC R_t = 11.7 min, purity: 99%; ^{1}H NMR (300 MHz, $CDCl_3$) δ 9.00 (d, J = 1.5 Hz, 1H), 8.91 (s, 1H), 8.63 (dd, J = 4.7, 1.3 Hz, 1H), 8.45 (d, J = 5.2 Hz, 1H), 8.12 (d, J = 8.0 Hz, 2H), 7.61 – 7.50 (m, 4H), 7.51 (s, 2H), 7.48 – 7.38 (m, 4H), 7.30 – 7.28 (m, 1H), 7.21 (d, J = 8.8 Hz, 1H), 2.14 (s, 3H) ppm; ^{13}C NMR (75 MHz, $CDCl_3$) δ 167.2, 165.1, 164.0, 159.8, 152.2, 151.5, 148.0, 136.8, 135.8, 135.5, 131.4, 130.8, 128.9, 127.3, 123.6, 117.8, 114.3, 111.8, 16.0 ppm; HRMS (ESI) calcd for $[C_{23}H_{18}N_4O_2+H]^+$ 383.1430, found 383.1507.

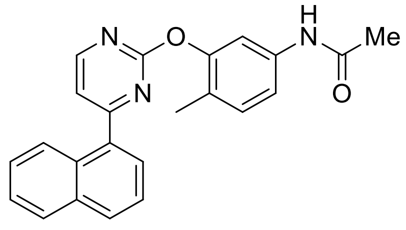

C1

N-(4-methyl-3-((4-(naphthalen-1-yl)pyrimidin-2-yl)oxy)phenyl)acetamide: A round bottom flask was charged with 0.32 g **8** **(4-methyl-3-((4-(naphthalen-1-yl)pyrimidin-2-yl)oxy)aniline)**, 0.07 g of acetic acid and 10 mL DCM. 0.15 g HOBt and 0.21 g of EDCI were added to the solution at room temperature. After stirring for 2 hours, 20 mL distilled water was added and the mixture was extracted with 20 mL DCM for two times. The organic layers were combined and washed with water and brine. The solvent was removed under reduced pressure and the residue was purified through flash chromatography on silica gel with DCM:MeOH = 200:1(v:v) as eluent. 0.37 g of **C1** was obtained in the yield of 96%. HPLC R_t = 20.7 min, purity: 99%; ^{1}H NMR (300 MHz, $CDCl_3$) δ 8.49 (d, J = 5.1 Hz, 1H), 8.22 – 8.08 (m, 1H), 7.90 – 7.71 (m, 3H), 7.60 (d, J = 6.6 Hz, 1H), 7.50 – 7.34 (m, 4H), 7.23 (d, J = 5.1 Hz, 1H), 7.13 – 7.05 (m, 2H), 2.10 (s, 3H), 1.92 (s, 3H) ppm; ^{13}C NMR (75 MHz, $CDCl_3$) δ 169.8, 168.4, 165.1, 159.4, 151.4, 137.3, 135.0, 133.9,

131.3, 130.8, 130.4, 128.6, 128.3, 127.1, 126.3, 126.0, 125.2, 125.0, 117.0, 116.8, 113.6, 24.4, 16.0 ppm; HRMS (ESI) calcd for [$C_{23}H_{19}N_3O_2$+H]+ 370.1477, found 370.1556.

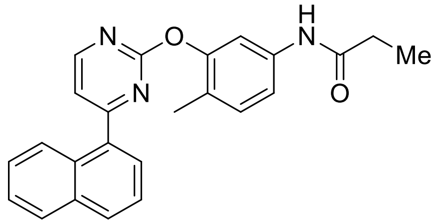

C2

N-(4-methyl-3-((4-(naphthalen-1-yl)pyrimidin-2-yl)oxy)phenyl)propionamide: A round bottom flask was charged with 0.32 g **8 (4-methyl-3-((4-(naphthalen-1-yl)pyrimidin-2-yl)oxy)aniline)**, 0.08 g of acetic acid and 10 mL DCM. 0.15 g HOBt and 0.21 g of EDCI were added to the solution at room temperature. After stirring for 2 hours, 20 mL distilled water was added and the mixture was extracted with 20 mL DCM for two times. The organic layers were combined and washed with water and brine. The solvent was removed under reduced pressure and the residue was purified through flash chromatography on silica gel with DCM:MeOH = 200:1(v:v) as eluent. 0.36 g of **C2** was obtained in the yield of 94%. HPLC R_t = 19.1 min, purity: 99%; ^{1}H NMR (300 MHz, $CDCl_3$) δ 8.48 (d, J = 5.0 Hz, 1H), 8.23 – 8.10 (m, 1H), 7.91 – 7.69 (m, 3H), 7.59 (d, J = 7.0 Hz, 1H), 7.52 – 7.33 (m, 4H), 7.20 (t, J = 7.1 Hz, 1H), 7.12 (d, J = 7.9 Hz, 1H), 7.05 (d, J = 8.1 Hz, 1H), 2.21 – 2.12 (m, 2H), 2.10 (s, 3H), 1.04 (t, J = 7.5 Hz, 3H) ppm; ^{13}C NMR (75 MHz, $CDCl_3$) δ 172.2, 169.8, 165.1, 159.4, 151.4, 137.4, 135.0, 133.9, 131.2, 130.8, 130.4, 128.6, 128.3, 127.1, 126.3, 125.8, 125.2, 125.1, 116.9, 116.8, 113.6, 30.4, 16.0, 9.6 ppm; HRMS (ESI) calcd for [$C_{24}H_{21}N_3O_2$+H]+ 384.1634, found 384.1712.

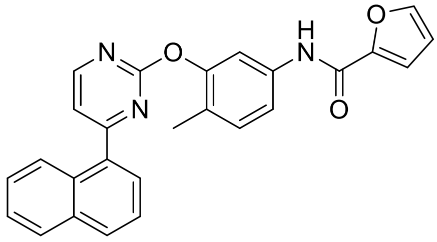

C3

N-(4-methyl-3-((4-(naphthalen-1-yl)pyrimidin-2-yl)oxy)phenyl)furan-2-carboxamide: A round bottom flask was charged with 0.32 g **8 (4-methyl-3-((4-(naphthalen-1-yl)pyrimidin-2-yl)oxy)aniline)**, 0.12 g of furan-2-carboxylic acid and 10 mL DCM. 0.15 g HOBt and 0.21 g of EDCI were added to the solution at room temperature. After stirring for 2 hours, 20 mL distilled water was added and the mixture was extracted with 20 mL DCM for two times. The organic layers were combined and washed with water and brine. The solvent was removed under reduced pressure and the residue was purified through flash chromatography on silica gel with DCM:MeOH = 200:1(v:v) as eluent. 0.35 g of **C3** was obtained in the yield of 83%. HPLC R_t = 18.3 min, purity: 99%; ^{1}H NMR (300 MHz, $CDCl_3$) δ 8.62 (d, J = 5.1 Hz, 1H), 8.32 – 8.22 (m, 1H), 8.13 (s, 1H), 7.94 (d, J = 8.2 Hz, 1H), 7.89 (dd, J = 6.4, 3.0 Hz, 1H), 7.77 – 7.67 (m, 2H), 7.60 – 7.43 (m,

4H), 7.42 – 7.29 (m, 2H), 7.23 (dd, J = 11.5, 5.5 Hz, 2H), 6.52 (dd, J = 3.4, 1.7 Hz, 1H), 2.24 (s, 3H) ppm; ^{13}C NMR (75 MHz, $CDCl_3$) δ 169.7, 165.1, 159.6, 155.9, 151.7, 147.8, 144.2, 136.4, 135.0, 133.9, 131.4, 130.7, 130.4, 128.5, 128.3, 127.1, 126.7, 126.2, 125.2, 125.1, 117.0, 116.8, 115.3, 113.8, 112.6, 16.0 ppm; HRMS (ESI) calcd for $[C_{26}H_{19}N_3O_3+H]^+$ 422.1426, found 422.1504.

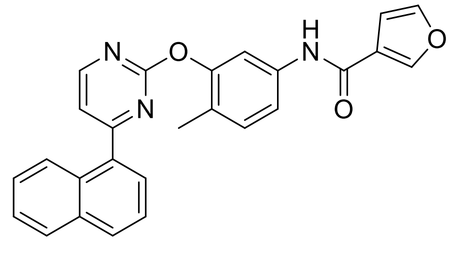

C4

N-(4-methyl-3-((4-(naphthalen-1-yl)pyrimidin-2-yl)oxy)phenyl)furan-3-carboxamide: A round bottom flask was charged with 0.32 g **8 (4-methyl-3-((4-(naphthalen-1-yl)pyrimidin-2-yl)oxy)aniline)**, 0.12 g of furan-3-carboxylic acid and 10 mL DCM. 0.15 g HOBt and 0.21 g of EDCI were added to the solution at room temperature. After stirring for 2 hours, 20 mL distilled water was added and the mixture was extracted with 20 mL DCM for two times. The organic layers were combined and washed with water and brine. The solvent was removed under reduced pressure and the residue was purified through flash chromatography on silica gel with DCM:MeOH = 200:1(v:v) as eluent. 0.38 g of **C4** was obtained in the yield of 90%. HPLC R_t = 20.3 min, purity: 99%; ^{1}H NMR (300 MHz, $CDCl_3$) δ 8.58 (d, J = 5.1 Hz, 1H), 8.29 – 8.18 (m, 1H), 7.97 (s, 1H), 7.93 (d, J = 8.2 Hz, 1H), 7.88 (dd, J = 6.1, 3.2 Hz, 1H), 7.78 (s, 1H), 7.69 (d, J = 7.1 Hz, 1H), 7.61 (s, 1H), 7.56 – 7.43 (m, 3H), 7.40 (s, 1H), 7.33 (dd, J = 11.0, 3.4 Hz, 2H), 7.27 – 7.16 (m, 1H), 6.68 (s, 1H), 2.21 (s, 3H) ppm; ^{13}C NMR (75 MHz, $CDCl_3$) δ 169.8, 165.1, 160.7, 159.5, 151.6, 145.1, 143.9, 136.7, 135.0, 133.9, 131.4, 130.8, 130.4, 128.6, 128.3, 127.1, 126.7, 126.25, 125.2, 125.1, 123.0, 117.4, 116.8, 114.1, 108.4, 16.0 ppm; HRMS (ESI) calcd for $[C_{26}H_{19}N_3O_3+H]^+$ 422.1426, found 422.1506.

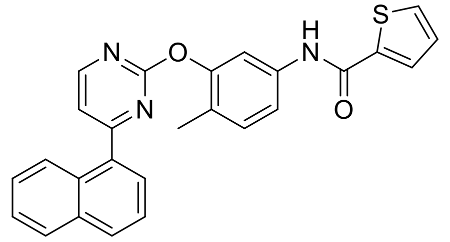

C5

N-(4-methyl-3-((4-(naphthalen-1-yl)pyrimidin-2-yl)oxy)phenyl)thiophene-2-carboxamide: A round bottom flask was charged with 0.32 g **8 (4-methyl-3-((4-(naphthalen-1-yl)pyrimidin-2-yl)oxy)aniline)**, 0.14 g of thiophene-2-carboxylic acid and 10 mL DCM. 0.15 g HOBt and 0.21 g of EDCI were added to the solution at room temperature. After stirring for 2 hours, 20 mL distilled water was added and the mixture was extracted with 20 mL DCM for two times. The organic layers were combined and washed with water and brine. The solvent was removed under reduced pressure and the residue was purified through flash

chromatography on silica gel with DCM:MeOH = 200:1(v:v) as eluent. 0.38 g of **C5** was obtained in the yield of 90%. HPLC R_t = 19.7 min, purity: 98%; ^{1}H NMR (300 MHz, $CDCl_3$) δ 8.61 (d, J = 5.1 Hz, 1H), 8.32 – 8.23 (m, 1H), 7.99 – 7.85 (m, 3H), 7.78 – 7.64 (m, 3H), 7.59 – 7.42 (m, 4H), 7.35 (dd, J = 7.3, 3.9 Hz, 3H), 7.25 (d, J = 7.8 Hz, 1H), 2.24 (s, 3H) ppm; ^{13}C NMR (75 MHz, $CDCl_3$) δ 169.7, 159.5, 136.8, 135.1, 133.9, 131.4, 130.7, 130.4, 128.6, 128.5, 128.3, 127.1, 126.9, 126.2, 126.1, 125.2, 125.1, 117.2, 116.8, 114.0, 16.0 ppm; HRMS (ESI) calcd for $[C_{26}H_{19}N_3O_2S+H]^+$ 438.1198, found 438.1276.

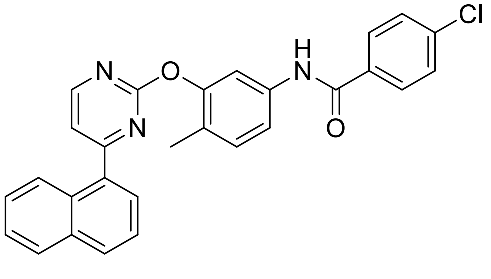

C6

4-chloro-N-(4-methyl-3-((4-(naphthalen-1-yl)pyrimidin-2-yl)oxy)phenyl)benzamide: A round bottom flask was charged with 0.32 g **8 (4-methyl-3-((4-(naphthalen-1-yl)pyrimidin-2-yl)oxy)aniline)**, 0.14 g of 4-chlorobenzoic acid and 10 mL DCM. 0.15 g HOBt and 0.21 g of EDCI were added to the solution at room temperature. After stirring for 2 hours, 20 mL distilled water was added and the mixture was extracted with 20 mL DCM for two times. The organic layers were combined and washed with water and brine. The solvent was removed under reduced pressure and the residue was purified through flash chromatography on silica gel with DCM:MeOH = 200:1(v:v) as eluent. 0.40 g of **C6** was obtained in the yield of 86%. HPLC R_t = 9.7 min, purity: 99%; ^{1}H NMR (300 MHz, $CDCl_3$) δ 8.57 – 8.44 (m, 2H), 8.21 – 8.12 (m, 1H), 7.90 – 7.76 (m, 2H), 7.67 – 7.55 (m, 4H), 7.43 (dd, J = 9.0, 5.6 Hz, 3H), 7.39 – 7.31 (m, 1H), 7.27 – 7.10 (m, 4H), 2.18 (s, 3H) ppm; ^{13}C NMR (75 MHz, $CDCl_3$) δ 169.7, 165.1, 164.9, 159.5, 151.5, 137.8, 137.0, 134.8, 133.8, 133.1, 131.4, 130.8, 130.3, 128.7, 128.6, 128.2, 127.1, 126.7, 126.2, 125.1, 125.0, 117.7, 116.9, 114.3, 16.0 ppm; HRMS (ESI) calcd for $[C_{28}H_{20}N_3O_2Cl+H]^+$ 466.1244, found 466.1313.

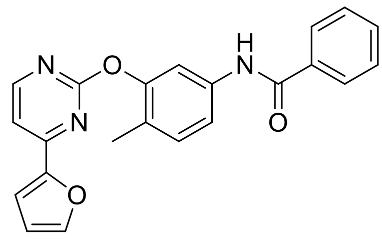

D1

N-(3-((4-(furan-2-yl)pyrimidin-2-yl)oxy)-4-methylphenyl)benzamide: A round bottom flask was charged with 0.26 g **8 3-((4-(furan-2-yl)pyrimidin-2-yl)oxy)-4-methylaniline**, 0.13 g of benzoic acid and 10 mL DCM. 0.15 g HOBt and 0.21 g of EDCI were added to the solution at room temperature. After stirring for 2 hours, 20 mL distilled water was added and the mixture was extracted with 20 mL DCM for two times. The organic layers were combined and washed with water and brine. The solvent was removed under reduced

pressure and the residue was purified through flash chromatography on silica gel with DCM:MeOH = 100:1(v:v) as eluent. 0.31 g of **D1** was obtained in the yield of 83%. HPLC R_t = 15.7 min, purity: 99%; ^{1}H NMR (300 MHz, $CDCl_3$) δ 8.46 (d, J = 5.2 Hz, 1H), 7.99 (s, 1H), 7.82 (d, J = 7.5 Hz, 2H), 7.60 (s, 1H), 7.56 – 7.37 (m, 5H), 7.31 (t, J = 4.6 Hz, 2H), 7.25 (d, J = 8.8 Hz, 1H), 6.63 – 6.49 (m, 1H), 2.17 (s, 3H) ppm; ^{13}C NMR (75 MHz, $CDCl_3$) δ 166.1, 164.9, 159.7, 158.1, 151.3, 151.1, 145.5, 137.2, 134.8, 131.6, 131.2, 128.4, 127.8, 126.5, 117.7, 114.2, 113.5, 112.7, 109.7, 15.9 ppm; HRMS (ESI) calcd for $[C_{22}H_{17}N_3O_3+H]+$ 372.1270, found 372.1344.

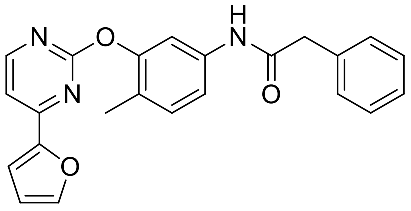

D2

N-(3-((4-(furan-2-yl)pyrimidin-2-yl)oxy)-4-methylphenyl)-2-phenylacetamide: A round bottom flask was charged with 0.26 g **8**
3-((4-(furan-2-yl)pyrimidin-2-yl)oxy)-4-methylaniline, 0.14 g of 2-phenylacetic acid and 10 mL DCM. 0.15 g HOBt and 0.21 g of EDCI were added to the solution at room temperature. After stirring for 2 hours, 20 mL distilled water was added and the mixture was extracted with 20 mL DCM for two times. The organic layers were combined and washed with water and brine. The solvent was removed under reduced pressure and the residue was purified through flash chromatography on silica gel with DCM:MeOH = 100:1(v:v) as eluent. 0.36 g of **D2** was obtained in the yield of 93%. HPLC R_t = 16.5 min, purity: 99%; ^{1}H NMR (300 MHz, $CDCl_3$) δ 8.40 (d, J = 5.2 Hz, 1H), 7.63 (s, 1H), 7.58 (dd, J = 1.5, 0.6 Hz, 1H), 7.39 – 7.19 (m, 10H), 7.14 (d, J = 8.2 Hz, 1H), 6.54 (dd, J = 3.5, 1.7 Hz, 1H), 3.64 (s, 2H), 2.12 (s, 3H) ppm; ^{13}C NMR (75 MHz, $CDCl_3$) δ 169.2, 164.9, 159.8, 158.2, 151.3, 151.2, 145.5, 137.9, 136.8, 134.5, 131.2, 129.5, 129.4, 129.1, 128.6, 127.5, 127.1, 126.6, 117.2, 113.6, 113.6, 112.7, 109.7, 44.6, 15.9 ppm; HRMS (ESI) calcd for $[C_{23}H_{19}N_3O_3+H]+$ 386.1426, found 386.1501.

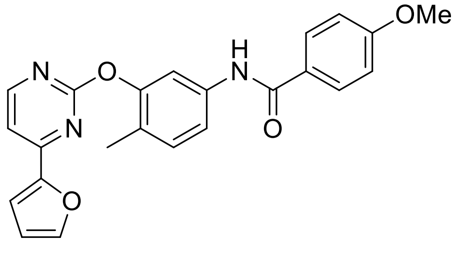

D3

N-(3-((4-(furan-2-yl)pyrimidin-2-yl)oxy)-4-methylphenyl)-4-methoxybenzamide: A round bottom flask was charged with 0.26 g **8**
3-((4-(furan-2-yl)pyrimidin-2-yl)oxy)-4-methylaniline, 0.16 g of 4-methoxybenzoic acid and 10 mL DCM. 0.15 g HOBt and 0.21 g of EDCI were added to the solution at room temperature. After stirring for 2 hours, 20 mL distilled water was added and the mixture was extracted with 20 mL DCM for two times. The organic layers were combined and washed with water and brine. The solvent was removed under reduced

pressure and the residue was purified through flash chromatography on silica gel with DCM:MeOH = 100:1(v:v) as eluent. 0.37 g of **D3** was obtained in the yield of 92%. HPLC R_t = 16.9 min, purity: 99%; ^1H NMR (300 MHz, $CDCl_3$) δ 8.43 (d, J = 5.2 Hz, 1H), 8.12 – 7.98 (m, 1H), 7.87 – 7.70 (m, 2H), 7.58 (dd, J = 1.6, 0.6 Hz, 1H), 7.50 (d, J = 2.1 Hz, 1H), 7.46 (dd, J = 8.1, 2.2 Hz, 1H), 7.32 – 7.25 (m, 2H), 7.21 (d, J = 8.2 Hz, 1H), 6.92 – 6.85 (m, 2H), 6.55 (dd, J = 3.5, 1.8 Hz, 1H), 3.82 (s, 3H), 2.15 (s, 3H) ppm; ^{13}C NMR (75 MHz, $CDCl_3$) δ 165.2, 165.0, 162.4, 159.9, 158.2, 151.43, 151.2, 145.4, 137.2, 131.3, 128.9, 127.1, 126.4, 117.5, 113.9, 113.8, 113.5, 112.7, 109.7, 29.7, 16.0 ppm; HRMS (ESI) calcd for $[C_{23}H_{19}N_3O_4+H]+$ 402.1376, found 402.1451.

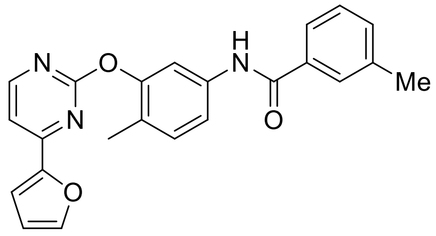

D4

N-(3-((4-(furan-2-yl)pyrimidin-2-yl)oxy)-4-methylphenyl)-3-methylbenzamide: A round bottom flask was charged with 0.26 g **8 3-((4-(furan-2-yl)pyrimidin-2-yl)oxy)-4-methylaniline**, 0.14 g of 3-methylbenzoic acid and 10 mL DCM. 0.15 g HOBt and 0.21 g of EDCI were added to the solution at room temperature. After stirring for 2 hours, 20 mL distilled water was added and the mixture was extracted with 20 mL DCM for two times. The organic layers were combined and washed with water and brine. The solvent was removed under reduced pressure and the residue was purified through flash chromatography on silica gel with DCM:MeOH = 100:1(v:v) as eluent. 0.36 g of **D4** was obtained in the yield of 93%. HPLC R_t = 15.2 min, purity: 96%; ^1H NMR (300 MHz, $CDCl_3$) δ 8.57 (s, 1H), 8.37 (d, J = 5.2 Hz, 1H), 7.62 – 7.44 (m, 5H), 7.32 – 7.08 (m, 5H), 6.49 (dd, J = 3.5, 1.7 Hz, 1H), 2.25 (s, 3H), 2.08 (s, 3H) ppm; ^{13}C NMR (75 MHz, $CDCl_3$) δ 166.3, 164.9, 159.8, 158.2, 151.3, 151.1, 145.5, 138.3, 138.1, 137.3, 134.8, 133.9, 132.4, 131.3, 130.5, 128.4, 128.2, 127.9, 127.2, 126.4, 124.2, 117.7, 114.2, 113.6, 112.7, 109.7, 21.3, 15.9 ppm; HRMS (ESI) calcd for $[C_{23}H_{19}N_3O_3+H]+$ 386.1426, found 386.1499.

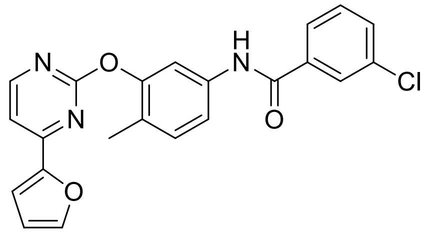

D5

3-chloro-N-(3-((4-(furan-2-yl)pyrimidin-2-yl)oxy)-4-methylphenyl)benzamide: A round bottom flask was charged with 0.26 g **8 3-((4-(furan-2-yl)pyrimidin-2-yl)oxy)-4-methylaniline**, 0.14 g of 3-chlorobenzoic acid and 10 mL DCM. 0.15 g HOBt and 0.21 g of EDCI were added to the solution at room temperature. After stirring for 2 hours, 20 mL distilled water was added and the mixture was extracted with 20 mL DCM for two times. The organic layers were combined and washed with water and brine. The solvent was removed under reduced pressure and the residue was purified through flash chromatography on silica gel with

DCM:MeOH = 100:1(v:v) as eluent. 0.38 g of **D5** was obtained in the yield of 94%. HPLC R_t = 14.7 min, purity: 99%; ^{1}H NMR (300 MHz, $CDCl_3$) δ 8.62 (s, 1H), 8.38 (d, J = 5.2 Hz, 1H), 7.74 (t, J = 1.7 Hz, 1H), 7.63 (d, J = 7.8 Hz, 1H), 7.60 – 7.53 (m, 1H), 7.50 – 7.44 (m, 2H), 7.37 (ddd, J = 7.9, 1.9, 1.0 Hz, 1H), 7.31 – 7.20 (m, 3H), 7.20 – 7.08 (m, 1H), 6.52 (dd, J = 3.5, 1.7 Hz, 1H), 2.08 (s, 3H) ppm; ^{13}C NMR (75 MHz, $CDCl_3$) δ 164.9, 164.7, 163.5, 159.7, 158.2, 156.7, 151.3, 151.1, 148.0, 145.5, 140.7, 136.9, 136.7, 134.6, 133.8, 131.6, 131.3, 129.8, 127.5, 126.8, 125.9, 125.3, 123.8, 117.8, 117.2, 114.2, 114.0, 113.6, 112.7, 110.0, 109.8, 101.2, 15.9 ppm; HRMS (ESI) calcd for $[C_{22}H_{16}N_3O_3Cl+H]^+$ 406.0880, found 406.0954.

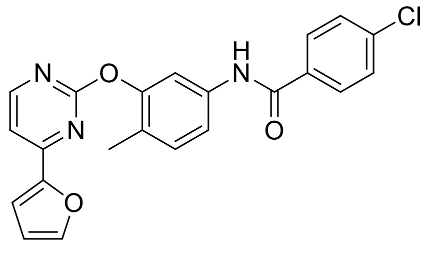

D6

4-chloro-N-(3-((4-(furan-2-yl)pyrimidin-2-yl)oxy)-4-methylphenyl)benzamide: A round bottom flask was charged with 0.26 g **8**
3-((4-(furan-2-yl)pyrimidin-2-yl)oxy)-4-methylaniline, 0.14 g of 4-chlorobenzoic acid and 10 mL DCM. 0.15 g HOBt and 0.21 g of EDCI were added to the solution at room temperature. After stirring for 2 hours, 20 mL distilled water was added and the mixture was extracted with 20 mL DCM for two times. The organic layers were combined and washed with water and brine. The solvent was removed under reduced pressure and the residue was purified through flash chromatography on silica gel with DCM:MeOH = 100:1(v:v) as eluent. 0.32 g of **D6** was obtained in the yield of 81%. HPLC R_t = 15.3 min, purity: 99%; ^{1}H NMR (300 MHz, $CDCl_3$) δ 8.60 (s, 1H), 8.37 (d, J = 5.1 Hz, 1H), 7.70 (d, J = 8.4 Hz, 2H), 7.56 (s, 1H), 7.46 (d, J = 7.1 Hz, 2H), 7.37 – 7.19 (m, 4H), 7.15 (d, J = 8.4 Hz, 1H), 6.53 (d, J = 1.6 Hz, 1H), 2.10 (s, 3H) ppm; ^{13}C NMR (75 MHz, $CDCl_3$) δ 165.0, 164.9, 159.7, 158.2, 151.3, 151.1, 145.5, 137.8, 137.0, 133.2, 131.3, 128.7, 128.7, 126.8, 117.8, 114.3, 113.6, 112.7, 109.8, 15.9 ppm; HRMS (ESI) calcd for $[C_{22}H_{16}N_3O_3Cl+H]^+$ 406.0880, found 406.0951.

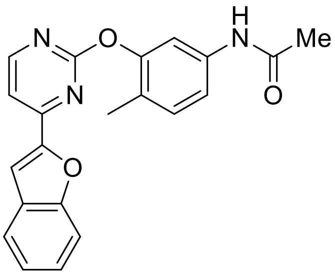

E1

N-(3-((4-(benzofuran-2-yl)pyrimidin-2-yl)oxy)-4-methylphenyl)acetamide: A round bottom flask was charged with 0.32 g **8**
3-((4-(benzofuran-2-yl)pyrimidin-2-yl)oxy)-4-methylaniline, 0.07 g of acetic acid and 10 mL DCM. 0.15 g HOBt and 0.21 g of EDCI were added to the solution at room temperature. After stirring for 2 hours, 20 mL distilled water was added and the mixture was extracted with 20 mL DCM for two times. The organic layers were

combined and washed with water and brine. The solvent was removed under reduced pressure and the residue was purified through flash chromatography on silica gel with DCM:MeOH = 100:1(v:v) as eluent. 0.33 g of **E1** was obtained in the yield of 92%. HPLC R_t = 13.3 min, purity: 99%; ^{1}H NMR (300 MHz, $CDCl_3$) δ 8.54 (d, J = 5.1 Hz, 1H), 7.88 (s, 1H), 7.70 – 7.58 (m, 2H), 7.53 (dd, J = 9.5, 6.8 Hz, 2H), 7.46 – 7.23 (m, 5H), 7.19 (d, J = 8.2 Hz, 1H), 2.15 (s, 3H), 2.07 (s, 3H) ppm; ^{13}C NMR (75 MHz, $CDCl_3$) δ 168.4, 165.1, 160.3, 158.6, 155.8, 152.3, 151.3, 137.2, 131.3, 128.1, 126.8, 126.2, 123.7, 122.5, 117.2, 113.6, 111.8, 110.8, 109.4, 24.4, 15.9 ppm; HRMS (ESI) calcd for $[C_{21}H_{17}N_3O_3+H]^+$ 360.1270, found 360.1362.

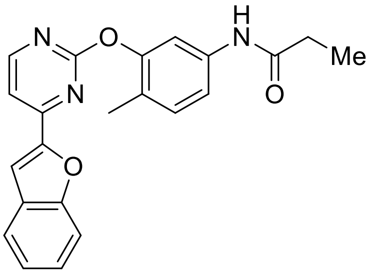

E2

N-(3-((4-(benzofuran-2-yl)pyrimidin-2-yl)oxy)-4-methylphenyl)propionamide: A round bottom flask was charged with 0.32 g **8** **3-((4-(benzofuran-2-yl)pyrimidin-2-yl)oxy)-4-methylaniline**, 0.08 g of propionic acid and 10 mL DCM. 0.15 g HOBt and 0.21 g of EDCI were added to the solution at room temperature. After stirring for 2 hours, 20 mL distilled water was added and the mixture was extracted with 20 mL DCM for two times. The organic layers were combined and washed with water and brine. The solvent was removed under reduced pressure and the residue was purified through flash chromatography on silica gel with DCM:MeOH = 100:1(v:v) as eluent. 0.32 g of **E2** was obtained in the yield of 86%. HPLC R_t = 12.5 min, purity: 99%; ^{1}H NMR (300 MHz, $CDCl_3$) δ 8.45 (d, J = 5.1 Hz, 1H), 7.56 (d, J = 8.0 Hz, 2H), 7.52 (s, 1H), 7.46 (d, J = 8.3 Hz, 1H), 7.42 (d, J = 5.1 Hz, 1H), 7.37 – 7.22 (m, 3H), 7.18 (t, J = 7.5 Hz, 1H), 7.10 (d, J = 8.2 Hz, 1H), 2.23 (q, J = 7.5 Hz, 2H), 2.07 (s, 3H), 1.09 (t, J = 7.5 Hz, 3H) ppm; ^{13}C NMR (75 MHz, $CDCl_3$) δ 165.1, 160.3, 158.5, 155.8, 152.4, 151.4, 137.2, 131.3, 128.1, 126.7, 126.07, 123.7, 122.5, 117.1, 115.0, 113.6, 111.8, 110.8, 109.4, 30.6, 15.9, 9.6 ppm; HRMS (ESI) calcd for $[C_{22}H_{19}N_3O_3+H]^+$ 374.1426, found 374.1497.

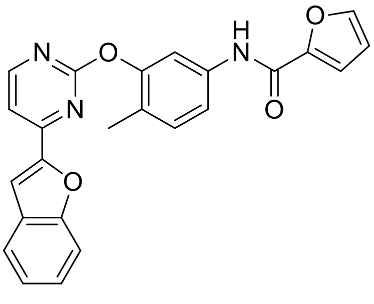

E3

N-(3-((4-(benzofuran-2-yl)pyrimidin-2-yl)oxy)-4-methylphenyl)furan-2-carboxamide: A round bottom flask was charged with 0.32 g **8** **3-((4-(benzofuran-2-yl)pyrimidin-2-yl)oxy)-4-methylaniline**, 0.12 g of

furan-2-carboxylic acid and 10 mL DCM. 0.15 g HOBt and 0.21 g of EDCI were added to the solution at room temperature. After stirring for 2 hours, 20 mL distilled water was added and the mixture was extracted with 20 mL DCM for two times. The organic layers were combined and washed with water and brine. The solvent was removed under reduced pressure and the residue was purified through flash chromatography on silica gel with DCM:MeOH = 100:1(v:v) as eluent. 0.36 g of **E4** was obtained in the yield of 87%. HPLC R_t = 12.8 min, purity: 96%; ^{1}H NMR (300 MHz, $CDCl_3$) δ 8.49 (d, J = 5.1 Hz, 1H), 8.08 (s, 1H), 7.61 – 7.52 (m, 2H), 7.52 – 7.35 (m, 5H), 7.35 – 7.25 (m, 1H), 7.18 (dd, J = 9.0, 5.7 Hz, 2H), 7.11 (d, J = 3.3 Hz, 1H), 6.42 (dd, J = 3.4, 1.7 Hz, 1H), 2.11 (s, 3H) ppm; ^{13}C NMR (75 MHz, $CDCl_3$) δ 165.1, 160.5, 158.5, 156.0, 155.8, 152.5, 151.6, 147.8, 144.2, 136.4, 131.4, 128.2, 126.7, 126.7, 123.6, 122.5, 117.2, 115.2, 113.8, 112.6, 111.8, 110.7, 109.3, 16.0 ppm; HRMS (ESI) calcd for $[C_{24}H_{17}N_3O_4+H]^+$ 412.1219, found 412.1291.

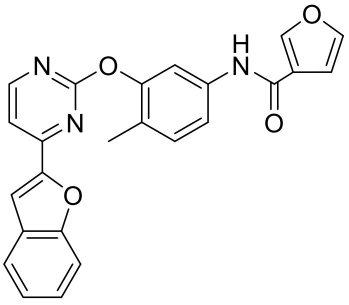

E4

N-(3-((4-(benzofuran-2-yl)pyrimidin-2-yl)oxy)-4-methylphenyl)furan-3-carboxamide: A round bottom flask was charged with 0.32 g **8 3-((4-(benzofuran-2-yl)pyrimidin-2-yl)oxy)-4-methylaniline**, 0.12 g of furan-3-carboxylic acid and 10 mL DCM. 0.15 g HOBt and 0.21 g of EDCI were added to the solution at room temperature. After stirring for 2 hours, 20 mL distilled water was added and the mixture was extracted with 20 mL DCM for two times. The organic layers were combined and washed with water and brine. The solvent was removed under reduced pressure and the residue was purified through flash chromatography on silica gel with DCM:MeOH = 100:1(v:v) as eluent. 0.33 g of **E5** was obtained in the yield of 80%. HPLC R_t = 12.0 min, purity: 99%; ^{1}H NMR (300 MHz, $CDCl_3$) δ 8.42 (d, J = 5.1 Hz, 1H), 8.08 (s, 1H), 7.90 (s, 1H), 7.57 – 7.46 (m, 2H), 7.46 – 7.33 (m, 4H), 7.33 – 7.24 (m, 2H), 7.16 (t, J = 7.5 Hz, 1H), 7.09 (d, J = 7.9 Hz, 1H), 6.63 (d, J = 0.9 Hz, 1H), 2.05 (s, 3H) ppm; ^{13}C NMR (75 MHz, $CDCl_3$) δ 165.0, 160.9, 160.4, 158.5, 155.8, 152.3, 151.4, 145.2, 143.8, 136.8, 131.4, 128.1, 126.8, 126.6, 123.7, 123.0, 122.5, 117.8, 114.2, 111.8, 110.8, 109.4, 108.5, 15.9 ppm; HRMS (ESI) calcd for $[C_{24}H_{17}N_3O_4+H]^+$ 412.1219, found 412.1289.

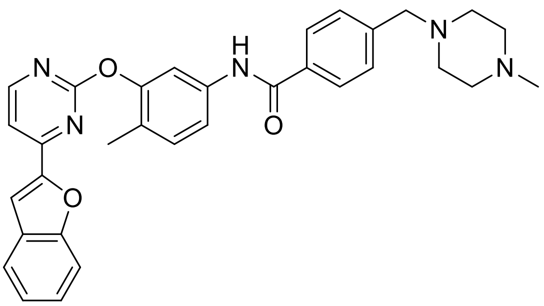

E5

N-(3-((4-(benzofuran-2-yl)pyrimidin-2-yl)oxy)-4-methylphenyl)-4-((4-methylpipe razin-1-yl)methyl)benzamide: A round bottom flask was charged with 0.32 g **8 3-((4-(benzofuran-2-yl)pyrimidin-2-yl)oxy)-4-methylaniline**, 0.50 g of 3-(4-methylpiperazin-1-yl)-2-oxopropanoic acid and 10 mL DCM. 0.15 g HOBt and 0.21 g of EDCI were added to the solution at room temperature. After stirring for 2 hours, 20 mL distilled water was added and the mixture was extracted with 20 mL DCM for two times. The organic layers were combined and washed with water and brine. The solvent was removed under reduced pressure and the residue was purified through flash chromatography on silica gel with DCM:MeOH = 10:1(v:v) as eluent. 0.25 g of **G2** was obtained in the yield of 47%. HPLC R_t = 6.8 min, purity: 99%; ^{1}H NMR (300 MHz, $CDCl_3$) δ 8.58 (d, J = 5.1 Hz, 1H), 7.87 (s, 1H), 7.79 (d, J = 8.1 Hz, 2H), 7.72 – 7.60 (m, 2H), 7.55 (dd, J = 6.3, 4.6 Hz, 3H), 7.51 – 7.33 (m, 4H), 7.33 – 7.21 (m, 3H), 3.55 (s, 2H), 2.46 (s, 8H), 2.29 (s, 3H), 2.21 (s, 3H) ppm; ^{13}C NMR (75 MHz, $CDCl_3$) δ 167.6, 165.0, 160.5, 158.5, 155.8, 152.4, 151.5, 136.5, 131.4, 128.1, 126.8, 126.7, 125.6, 124.4, 123.7, 122.5, 118.7, 116.9, 113.4, 111.8, 110.8, 110.4, 109.3, 61.5, 54.3, 51.8, 44.7, 16.0 ppm; HRMS (ESI) calcd for $[C_{32}H_{31}N_5O_3+H]^+$ 534.2427, found 534.2520.

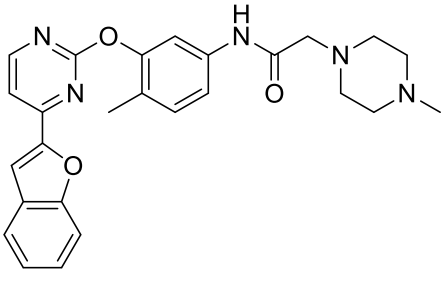

E6

N-(3-((4-(benzofuran-2-yl)pyrimidin-2-yl)oxy)-4-methylphenyl)-2-(4-methylpiper azin-1-yl)acetamide: A round bottom flask was charged with 0.32 g **8 3-((4-(benzofuran-2-yl)pyrimidin-2-yl)oxy)-4-methylaniline**, 0.30 g of 3-(4-methylpiperazin-1-yl)-2-oxopropanoic acid and 10 mL DCM. 0.15 g HOBt and 0.21 g of EDCI were added to the solution at room temperature. After stirring for 2 hours, 20 mL distilled water was added and the mixture was extracted with 20 mL DCM for two times. The organic layers were combined and washed with water and brine. The solvent was removed under reduced pressure and the residue was purified through flash chromatography on silica gel with DCM:MeOH = 10:1(v:v) as eluent. 0.29 g of **G2** was obtained in the yield of 63%. HPLC R_t = 7.9 min, purity: 97%; ^{1}H NMR (300 MHz, $CDCl_3$) δ 9.14 (s, 1H), 8.57 (d, J = 5.1 Hz, 1H), 7.66 (d, J = 7.7 Hz, 1H), 7.63 (d, J = 0.8 Hz, 1H), 7.59 – 7.54 (m, 1H), 7.53 (d, J = 5.1 Hz, 1H), 7.48 (d, J

= 2.0 Hz, 1H), 7.46 – 7.35 (m, 2H), 7.32 – 7.21 (m, 2H), 3.11 (s, 2H), 2.64 (s, 4H), 2.48 (s, 4H), 2.29 (s, 3H), 2.18 (s, 3H) ppm; ^{13}C NMR (75 MHz, $CDCl_3$) δ 164.4, 164.1, 159.5, 157.5, 154.8, 151.5, 150.5, 141.7, 136.0, 132.7, 130.4, 128.3, 127.2, 126.0, 125.7, 122.6, 121.5, 116.4, 112.9, 110.8, 109.7, 108.3, 61.5, 54.1, 52.1, 45.0, 15.0 ppm; HRMS (ESI) calcd for $[C_{26}H_{27}N_5O_3+H]+$ 458.2114, found 458.1998.

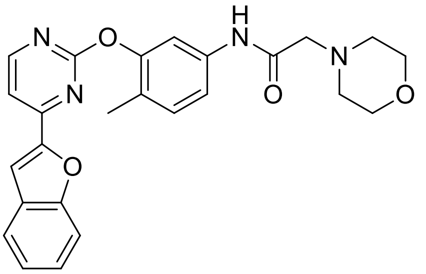

E7

N-(3-((4-(benzofuran-2-yl)pyrimidin-2-yl)oxy)-4-methylphenyl)-2-morpholinoacetamide: A round bottom flask was charged with 0.32 g **8 3-((4-(benzofuran-2-yl)pyrimidin-2-yl)oxy)-4-methylaniline**, 0.30 g of 2-morpholinoacetic acid and 10 mL DCM. 0.15 g HOBt and 0.21 g of EDCI were added to the solution at room temperature. After stirring for 2 hours, 20 mL distilled water was added and the mixture was extracted with 20 mL DCM for two times. The organic layers were combined and washed with water and brine. The solvent was removed under reduced pressure and the residue was purified through flash chromatography on silica gel with DCM:MeOH = 10:1(v:v) as eluent. 0.23 g of **G3** was obtained in the yield of 51%. HPLC R_t = 8.1 min, purity: 99%; ^{1}H NMR (300 MHz, $CDCl_3$) δ 8.99 (s, 1H), 8.49 (d, J = 5.1 Hz, 1H), 7.62 – 7.52 (m, 2H), 7.52 – 7.38 (m, 3H), 7.32 (dd, J = 7.9, 6.7 Hz, 2H), 7.26 – 7.13 (m, 2H), 3.72 – 3.59 (m, 4H), 3.03 (s, 2H), 2.57 – 2.44 (m, 4H), 2.10 (s, 3H) ppm; ^{13}C NMR (75 MHz, $CDCl_3$) δ 167.8, 165.1, 160.5, 158.5, 155.8, 152.4, 151.5, 136.5, 131.4, 128.1, 126.7, 126.6, 123.7, 122.4, 116.8, 113.2, 111.5, 110.7, 109.3, 67.0, 62.4, 53.8, 16.0 ppm; HRMS (ESI) calcd for $[C_{25}H_{24}N_4O_4+H]+$ 445.1798, found 445.1875.

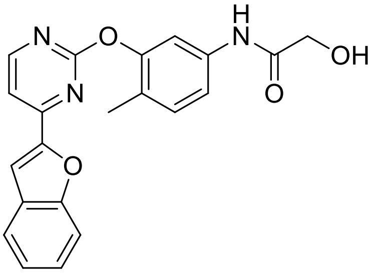

E8

N-(3-((4-(benzofuran-2-yl)pyrimidin-2-yl)oxy)-4-methylphenyl)-2-hydroxyacetamide: A round bottom flask was charged with 0.32 g **8 3-((4-(benzofuran-2-yl)pyrimidin-2-yl)oxy)-4-methylaniline**, 0.30 g of 2-hydroxyacetic acid and 10 mL DCM. 0.15 g HOBt and 0.21 g of EDCI were added to the solution at room temperature. After stirring for 2 hours, 20 mL distilled water was added and the mixture was extracted with 20 mL DCM for two times. The organic layers were combined and washed with water and brine. The solvent was removed under reduced pressure and the residue was purified through flash chromatography on silica gel with DCM:MeOH = 10:1(v:v) as eluent. 0.12 g of **G4**

was obtained in the yield of 31%. HPLC R_t = 5.9 min, purity: 99%; ^{1}H NMR (300 MHz, $CDCl_3$) δ 9.73 (s, 1H), 8.72 (d, J = 5.1 Hz, 1H), 7.81 (d, J = 9.1 Hz, 2H), 7.76 – 7.67 (m, 2H), 7.63 (s, 1H), 7.50 (dd, J = 15.7, 8.3 Hz, 2H), 7.35 (t, J = 7.5 Hz, 1H), 7.26 (d, J = 8.3 Hz, 1H), 5.65 (t, J = 5.9 Hz, 1H), 3.98 (d, J = 5.9 Hz, 2H), 3.33 (s, 8H), 2.07 (s, 3H) ppm; ^{13}C NMR (75 MHz, $CDCl_3$) δ 171.3, 165.0, 161.7, 157.9, 155.6, 152.4, 151.4, 138.1, 131.3, 128.1, 127.5, 125.1, 124.3, 123.2, 117.1, 113.5, 112.2, 111.6, 109.9, 62.3, 15.9 ppm; HRMS (ESI) calcd for $[C_{21}H_{17}N_3O_4+H]^+$ 376.1219, found 376.1298.

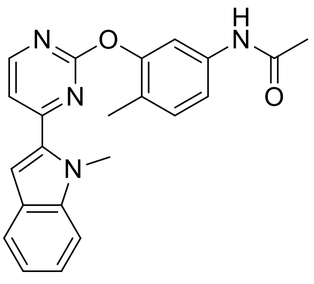

F1

N-(4-methyl-3-((4-(1-methyl-1H-indol-2-yl)pyrimidin-2-yl)oxy)phenyl)acetamide: A round bottom flask was charged with 0.33 g **8 4-methyl-3-((4-(1-methyl-1H-indol-2-yl)pyrimidin-2-yl)oxy)aniline**, 0.07 g of acetic acid and 10 mL DCM. 0.15 g HOBt and 0.21 g of EDCI were added to the solution at room temperature. After stirring for 2 hours, 20 mL distilled water was added and the mixture was extracted with 20 mL DCM for two times. The organic layers were combined and washed with water and brine. The solvent was removed under reduced pressure and the residue was purified through flash chromatography on silica gel with DCM:MeOH = 50:1(v:v) as eluent. 0.31 g of **F1** was obtained in the yield of 84%. HPLC R_t = 11.7 min, purity: 99%; ^{1}H NMR (300 MHz, DMSO-*d6*) δ 8.52 (d, J = 5.2 Hz, 1H), 8.40 (s, 1H), 7.86-7.97 (m, 1H), 7.70-7.47 (m, 4H), 7.31 (d, J = 1.8 Hz, 1H), 7.24 (t, J = 7.4 Hz, 1H), 6.99 (t, J = 7.4 Hz, 1H), 3.89 (s, 3H), 2.10 (s, 3H), 2.06 (s, 3H) ppm; ^{13}C NMR (75 MHz, DMSO-*d6*) δ 168.7, 165.3, 164.6, 159.1, 151.9, 138.9, 138.0, 134.0, 131.3, 126.0, 125.1, 122.8, 122.7, 121.4, 116.2, 113.4, 112.1, 110.8, 110.7, 33.6, 24.4, 16.0 ppm; HRMS (ESI) calcd for $[C_{22}H_{20}N_4O_2+H]^+$ 373.1586, found 373.1660.

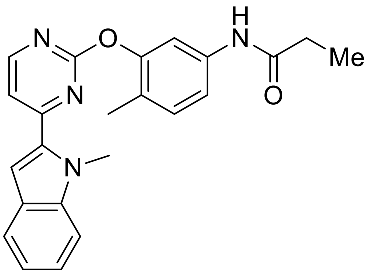

F2

N-(4-methyl-3-((4-(1-methyl-1H-indol-2-yl)pyrimidin-2-yl)oxy)phenyl)propionamide: A round bottom flask was charged with 0.33 g **8 4-methyl-3-((4-(1-methyl-1H-indol-2-yl)pyrimidin-2-yl)oxy)aniline**, 0.08 g of propionic acid and 10 mL DCM. 0.15 g HOBt and 0.21 g of EDCI were added to the solution at room temperature. After stirring for 2 hours, 20 mL distilled water was added and the mixture was extracted with 20 mL DCM for two times. The organic

layers were combined and washed with water and brine. The solvent was removed under reduced pressure and the residue was purified through flash chromatography on silica gel with DCM:MeOH = 50:1(v:v) as eluent. 0.33 g of **F1** was obtained in the yield of 85%. HPLC R_t = 12.1 min, purity: 99%; ^{1}H NMR (300 MHz, DMSO-*d6*) δ 9.91 (s, 1H), 8.44 (d, J = 5.4 Hz, 1H), 8.38 (s, 1H), 7.82 (d, J = 8.0 Hz, 1H), 7.59 – 7.39 (m, 4H), 7.28 (d, J = 8.3 Hz, 1H), 7.20 (t, J = 7.2 Hz, 1H), 6.96 (t, J = 7.3 Hz, 1H), 3.85 (s, 3H), 3.33 (s, 5H), 2.29 (q, J = 7.5 Hz, 2H), 2.06 (s, 3H), 1.06 (t, J = 7.5 Hz, 3H) ppm; ^{13}C NMR (75 MHz, DMSO-*d6*) δ 171.9, 164.8, 164.1, 158.6, 151.5, 138.5, 137.6, 133.5, 130.8, 125.5, 124.5, 122.3, 122.2, 120.9, 115.7, 112.9, 110.4, 110.3, 33.1, 29.5, 15.5, 9.6 ppm; HRMS (ESI) calcd for $[C_{23}H_{22}N_4O_2+H]^+$ 387.1743, found 387.1822.

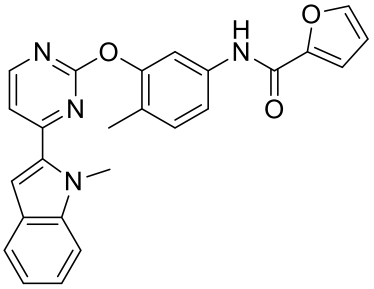

F3

N-(4-methyl-3-((4-(1-methyl-1H-indol-2-yl)pyrimidin-2-yl)oxy)phenyl)furan-2-carboxamide: A round bottom flask was charged with 0.33 g **8 4-methyl-3-((4-(1-methyl-1H-indol-2-yl)pyrimidin-2-yl)oxy)aniline**, 0.12 g of furan-2-carboxylic acid and 10 mL DCM. 0.15 g HOBt and 0.21 g of EDCI were added to the solution at room temperature. After stirring for 2 hours, 20 mL distilled water was added and the mixture was extracted with 20 mL DCM for two times. The organic layers were combined and washed with water and brine. The solvent was removed under reduced pressure and the residue was purified through flash chromatography on silica gel with DCM:MeOH = 50:1(v:v) as eluent. 0.37 g of **F4** was obtained in the yield of 97%. HPLC R_t = 11.8 min, purity: 99%; ^{1}H NMR (300 MHz, DMSO-*d6*) δ 10.33 (s, 1H), 8.47 (d, J = 5.4 Hz, 1H), 8.38 (s, 1H), 8.00 – 7.91 (m, 2H), 7.88 (d, J = 8.0 Hz, 1H), 7.79 – 7.67 (m, 2H), 7.61 – 7.44 (m, 5H), 7.36 (d, J = 8.2 Hz, 1H), 7.19 (dd, J = 11.3, 4.0 Hz, 1H), 6.99 (t, J = 7.6 Hz, 1H), 3.85 (s, 3H), 2.12 (s, 3H) ppm; ^{13}C NMR (75 MHz, DMSO-*d6*) δ 164.8, 164.1, 158.6, 156.1, 151.4, 147.4, 145.6, 137.7, 137.6, 133.5, 130.8, 125.6, 125.5, 122.3, 122.2, 121.0, 117.0, 114.6, 114.1, 112.1, 111.6, 110.4, 110.3, 33.0, 15.5 ppm; HRMS (ESI) calcd for $[C_{25}H_{20}N_4O_3+H]^+$ 425.1535, found 425.1609.

Cell culture

Liver cancer cell lines of Hep3B, MHCC97-L, QGY-7703, SMMC-7721 and SNU368 were obtained from HD Biosciences (China) Co., Ltd. Hep3B cells were cultured in DMEM medium and all the other cell lines in RPMI 1640 medium. All media contained 10% fetal bovine serum (FBS) (Bioind, US origin) and all cells were incubated in a humidified atmosphere of 5 % CO_2 at 37 °C. All the cell lines were

authenticated by using short tandem repeat (STR) matching analysis. No mycoplasma contamination was detected.

CellTiter-Glo luminescent cell viability assay

For experiments hepatocellular carcinoma cells were seeded at 4000 cells per well in 96-well plates. After overnight incubation, solution of compounds in DMSO at various concentrations were added to the cells and further incubated for 120 h. Cell viability was measured with the CellTiter-Glo Luminescent Cell Viability Assay (Promega) according to the manufacturer's instructions. Luminescence was measured using an EnVision Multilabel Plate Reader (Perkin-Elmer). The cytotoxicity of the compounds was expressed as the percentage of luminescence relative to that of untreated cells. The concentration of compound producing 50% of cell proliferation (IC_{50}) was calculated by nonlinear regression analysis of the response-concentration (log) curve, using the Graph-Pad Prism program package (Graph Pad Software; San Diego, CA). Results are expressed as the means of two dependent experiments performed in triplicate.

Supplementary Table S1 PDGFR kinase inhibition of 2-phenyloxypyrimidine derivatives

Compound ID	Inhibition effect (%)[a]		IC_{50} (μM)[b]		Selectivity index[c]
	PDGFRα	PDGFRβ	PDGFRα	PDGFRβ	
A1	39.11	21.40	N.D.[d]	N.D.	N.D.
A2	65.72	40.73	N.D.	N.D.	N.D.
A3	45.45	45.50	N.D.	N.D.	N.D.
A4	66.71	43.74	N.D.	N.D.	N.D.
A5	45.20	33.70	N.D.	N.D.	N.D.
A6	47.73	35.96	N.D.	N.D.	N.D.
A7	62.93	40.23	N.D.	N.D.	N.D.
A8	55.65	43.24	N.D.	N.D.	N.D.
A9	23.63	23.41	N.D.	N.D.	N.D.
A10	40.63	30.94	N.D.	N.D.	N.D.
A11	42.91	6.84	N.D.	N.D.	N.D.
A12	43.17	31.94	N.D.	N.D.	N.D.
B1	2.32	12.87	N.D.	N.D.	N.D.
B2	21.09	17.63	N.D.	N.D.	N.D.
B3	23.12	26.17	N.D.	N.D.	N.D.
B4	35.04	24.42	N.D.	N.D.	N.D.
B5	1.81	19.39	N.D.	N.D.	N.D.
B6	12.21	18.64	N.D.	N.D.	N.D.
B7	31.49	16.63	N.D.	N.D.	N.D.
B8	26.17	37.71	N.D.	N.D.	N.D.
B9	21.34	19.14	N.D.	N.D.	N.D.
B10	19.57	15.63	N.D.	N.D.	N.D.
B11	32.00	5.08	N.D.	N.D.	N.D.
B12	25.66	20.65	N.D.	N.D.	N.D.
C1	33.27	1.32	N.D.	N.D.	N.D.
C2	33.78	2.07	N.D.	N.D.	N.D.
C3	46.46	11.61	N.D.	N.D.	N.D.
C4	13.73	2.83	N.D.	N.D.	N.D.
C5	16.27	5.08	N.D.	N.D.	N.D.
C6	24.14	-0.94	N.D.	N.D.	N.D.
D1	13.74	28.93	N.D.	N.D.	N.D.
D2	36.82	46.75	N.D.	N.D.	N.D.
D3	22.61	37.72	N.D.	N.D.	N.D.
D4	45.70	46.00	N.D.	N.D.	N.D.
D5	51.79	43.49	N.D.	N.D.	N.D.
D6	33.02	37.47	N.D.	N.D.	N.D.
E1	1.56	1.82	N.D.	N.D.	N.D.
E2	19.06	10.10	N.D.	N.D.	N.D.
E3	-2.00	3.83	N.D.	N.D.	N.D.

E4	27.18	7.35	N.D.	N.D.	N.D.
**E5**	**86.12**	**66.41**	**0.40**	**0.93**	**2.32**
E6	-1.24	-0.44	N.D.	N.D.	N.D.
E7	18.56	19.39	N.D.	N.D.	N.D.
E8	26.16	14.62	N.D.	N.D.	N.D.
F1	6.37	2.32	N.D.	N.D.	N.D.
F2	12.21	-4.46	N.D.	N.D.	N.D.
F3	24.14	25.67	N.D.	N.D.	N.D.

[a] Inhibition effect (%): kinase inhibition activities of compounds at 1 μM.

[b] IC_{50}: 50% inhibitory concentration (averages of two separate experiments).

[c] Selectivity index=IC_{50} (PDGFRβ)/ IC_{50} (PDGFRα).

[d] N.D. = not determined.

Supplementary Table S2 Anti-proliferative activity of 2-phenyloxypyrimidine derivatives on HCC cell lines determined by CellTiter-Glo luminescent cell viability assay (IC_{50}, μM)

Compound ID	BEL7404	Hep3B	MHCC97-L	QGY-7703	SMMC-7721	SNU368
A1	10.8	15.8	15.1	24.5	13.6	85.7
A2	39.3	39.5	>200	59.8	84.3	112.2
A3	32.8	21.2	22.3	47.0	25.1	51.4
A4	12.2	17.5	24.5	39.8	17.5	54.0
A5	15.6	23.3	31.3	40.5	23.5	49.5
A6	9.5	10.7	16.2	21.9	12.3	24.0
A7	32.8	18.9	51.5	57.8	27.2	45.9
A8	20.1	14.3	34.6	24.6	10.1	25.7
A9	23.8	28.4	53.2	48.4	26.8	51.5
A10	28.2	20.1	35.7	43.4	21.1	38.7
A11	74.2	105.4	89.3	252.0	79.7	94.6
A12	133.7	130.3	281.3	170.4	112.6	286.6
B1	10.5	2.7	4.9	5.8	2.7	17.7
B2	14.4	13.0	16.9	20.6	11.7	24.8
B3	3.7	4.6	6.1	7.3	6.0	12.8
B4	9.5	6.4	7.0	10.2	6.2	12.9
B5	7.9	9.2	9.8	14.9	8.4	15.8
B6	13.4	7.9	10.5	15.4	9.7	15.0
B7	18.3	15.5	>200	25.8	17.0	14.0
B8	9.9	9.0	14.0	14.4	7.5	12.0
B9	12.9	83.2	>200	19.2	13.8	16.8
B10	8.1	5.8	6.3	10.9	3.9	12.6
B11	21.6	44.2	53.5	81.1	20.2	18.0
B12	13.4	17.7	44.0	27.6	8.8	18.0
C1	36.2	32.0	108.6	91.6	21.0	43.0
C2	48.2	36.3	110.7	119.5	29.5	55.9
C3	39.7	33.5	91.0	70.7	27.2	37.9
C4	8.8	22.9	96.4	14.9	12.0	15.8
C5	20.8	17.0	28.0	35.2	16.8	28.4
C6	6.7	19.7	>200	19.2	14.3	17.2
D1	48.7	11.4	1.6	2.5	1.5	37.3
D2	32.8	28.3	26.4	42.2	21.6	43.8
D3	17.6	9.7	8.5	15.7	7.9	22.2
D4	4.5	6.0	3.4	7.5	3.7	23.2
D5	12.4	11.2	14.7	22.5	12.4	24.0
D6	10.9	7.3	24.6	25.8	8.2	12.7
E1	10.1	86.8	5.7	0.3	0.3	8.5
E2	8.2	74.6	5.5	0.4	0.2	13.2

E3	1.2	125.0	9.6	0.7	0.7	1.3
E4	5.2	15.8	0.0	0.0	0.4	11.4
**E5**	**0.8**	**2.6**	**10.0**	**15.1**	**1.0**	**8.9**
E6	38.1	11.7	16.7	29.5	27.7	31.6
E7	5.9	9.0	59.1	10.3	4.6	14.7
E8	2.8	3.8	13.6	3.3	2.5	6.3
F1	37.1	31.0	48.4	195.4	34.5	29.3
F2	19.0	33.8	31.8	51.9	27.9	29.1
F3	15.6	41.8	>200	97.7	18.4	26.5

Supplementary Figure S1 Original gel images for Manuscript Fig. 5C

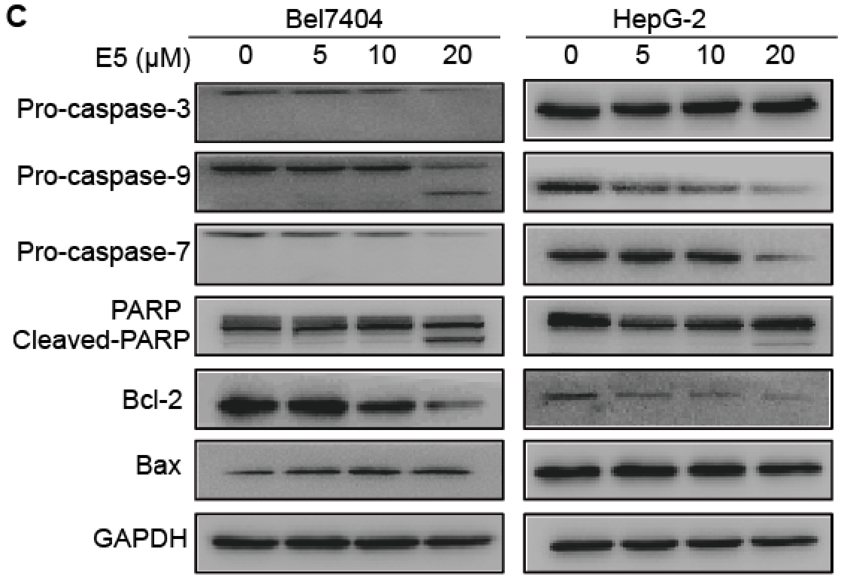

<u>Bel7404</u>

Procaspase-3:

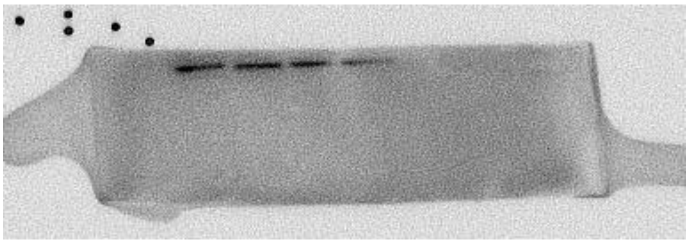

Procaspase-9:

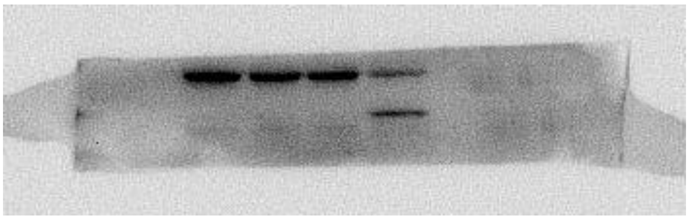

Procapase-7:

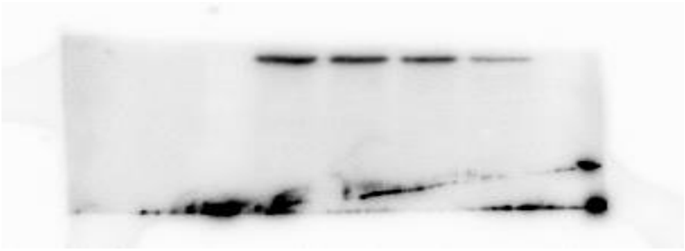

PARP:

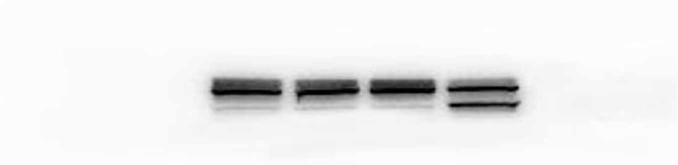

Bcl-2:

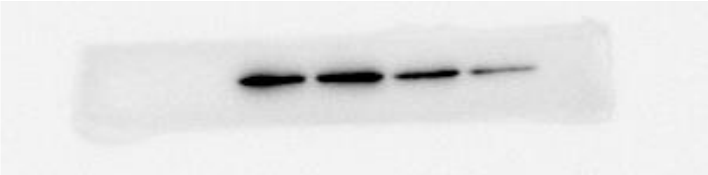

Bax：

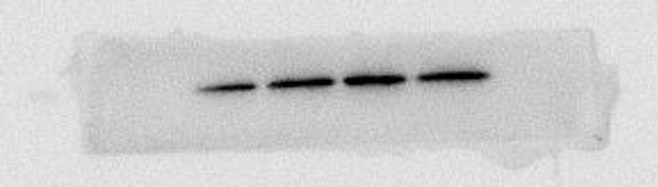

GAPDH:

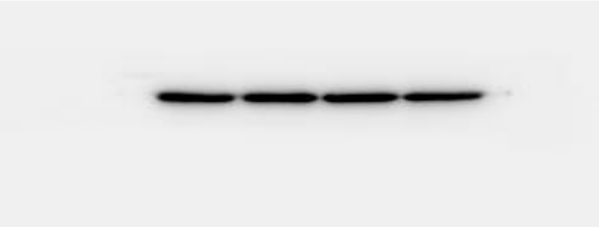

<u>HepG-2</u>

Procaspase-3:

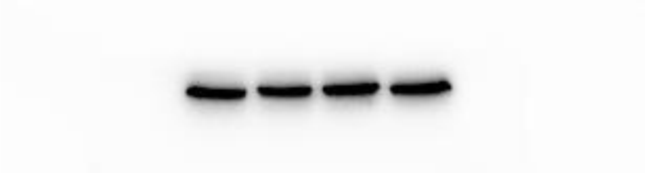

Procaspase-9:

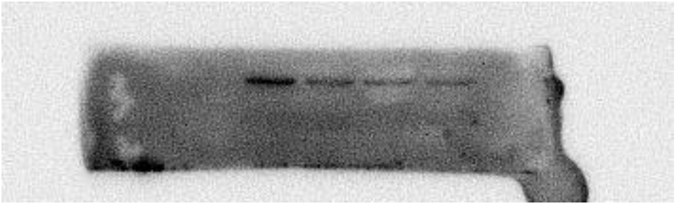

Procapase-7:

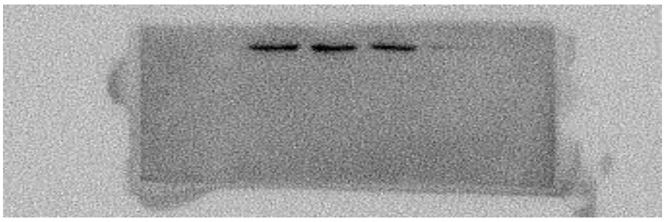

PARP:

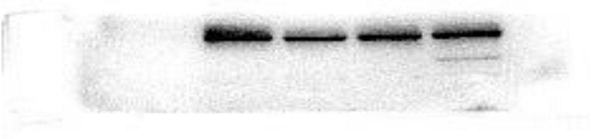

Bcl-2:

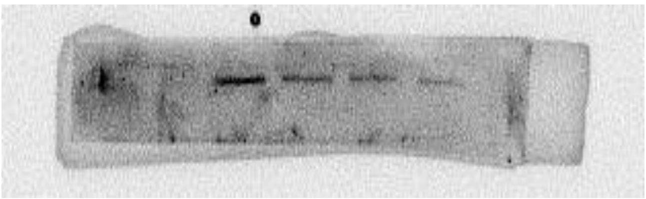

Bax：

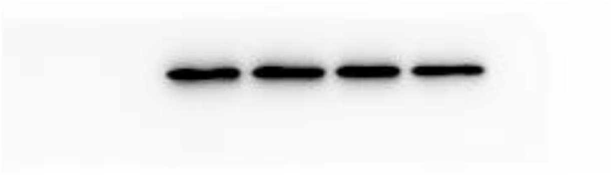

GAPDH:

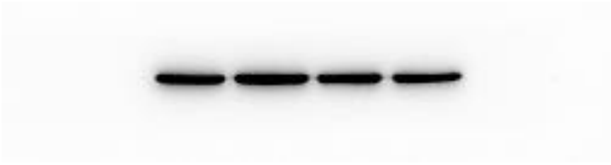

Supplementary Figure S2 Original gel images for Manuscript Fig. 6B

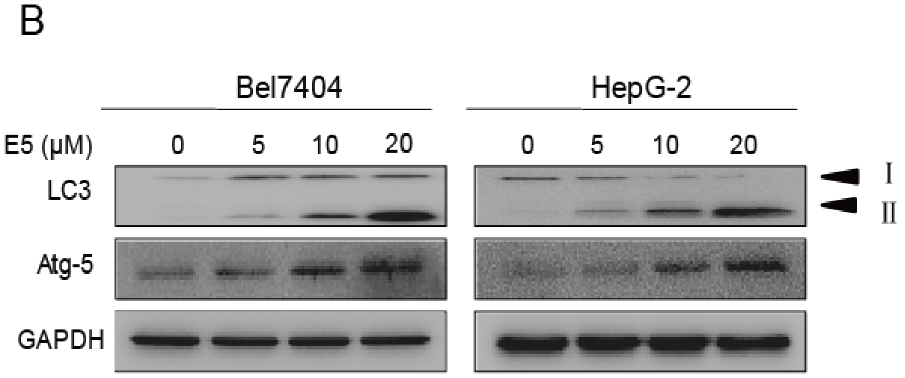

Bel7404

LC3:

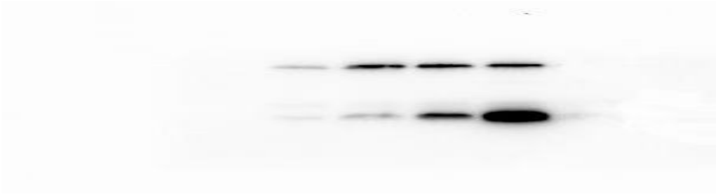

Atg-5:

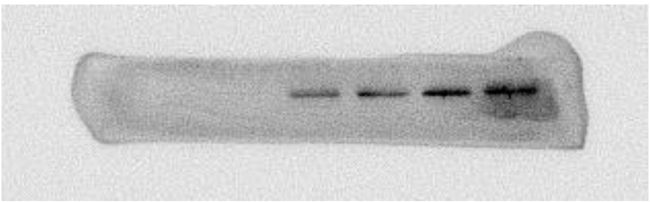

GAPDH:

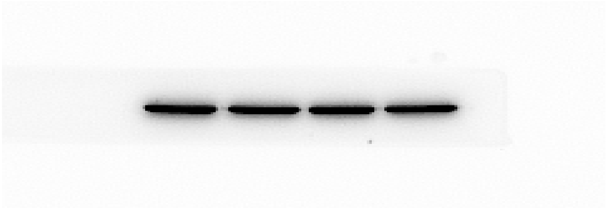

HepG-2

LC3:

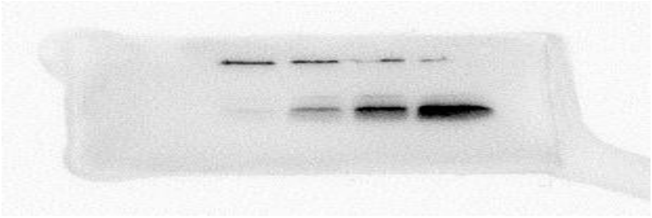

Atg-5:

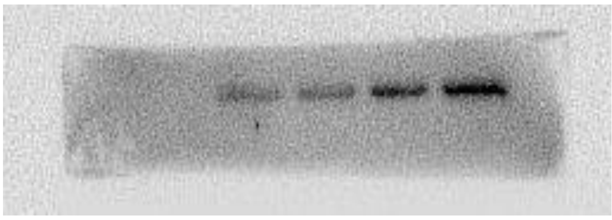

GAPDH:

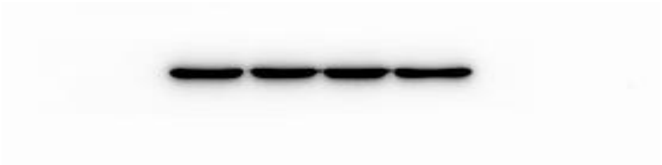

Supplementary Figure S3 Original gel images for Manuscript Fig. 6C

C

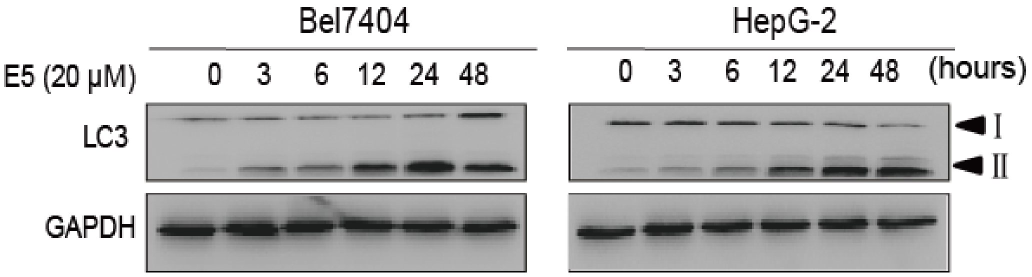

<u>Bel7404</u>

LC3：

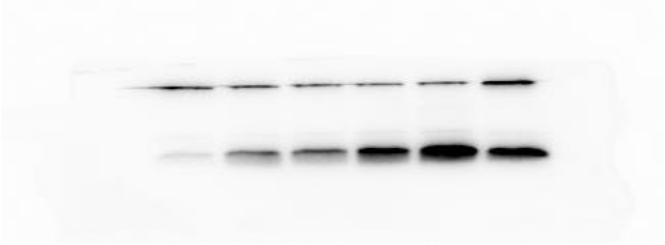

GAPDH:

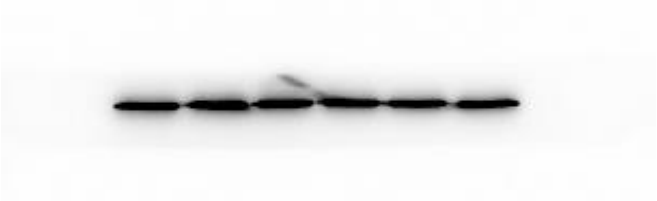

<u>HepG-2</u>

LC3:

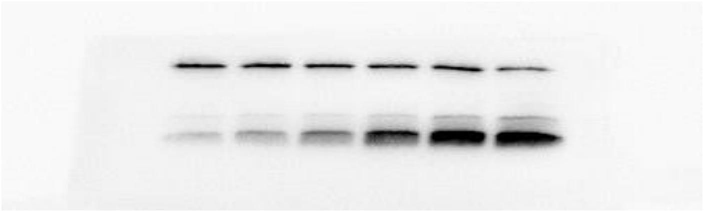

GAPDH:

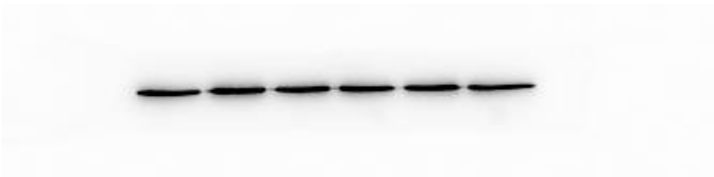

Supplementary Figure S4 Original gel images for Manuscript Fig. 6D

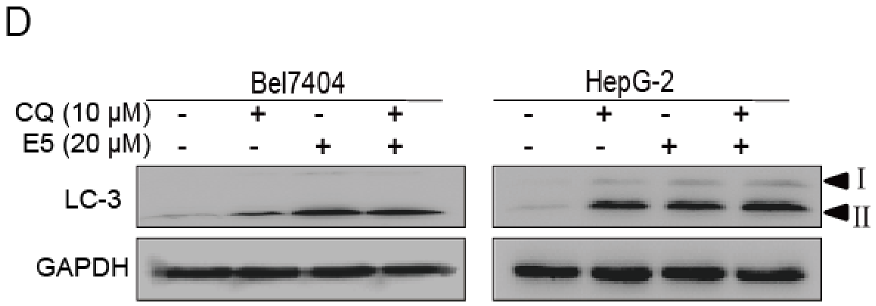

Bel7404

LC3：

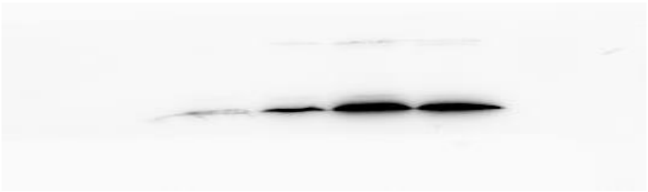

GAPDH:

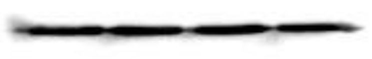

HepG-2

LC3：

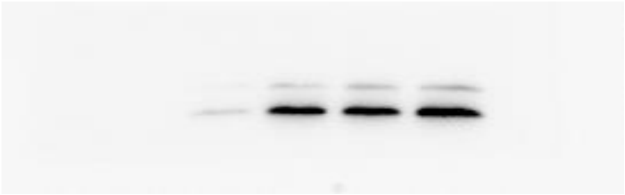

GAPDH:

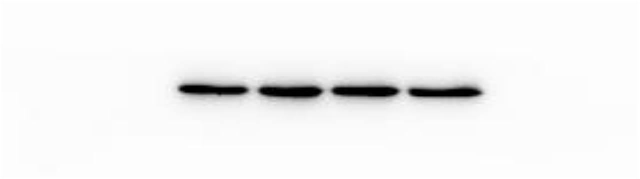

Supplementary Figure S5 Original gel images for Manuscript Fig. 6E

E

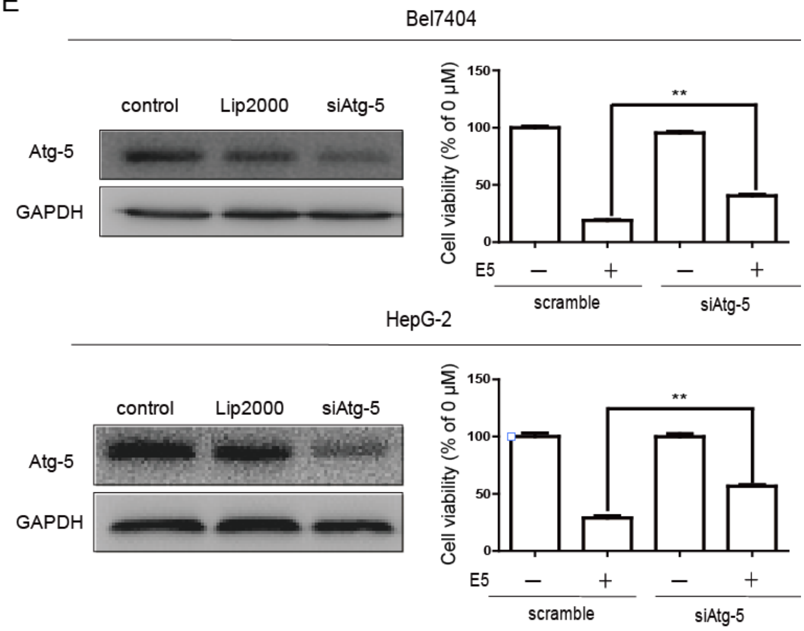

<u>Bel7404</u>

Atg-5:

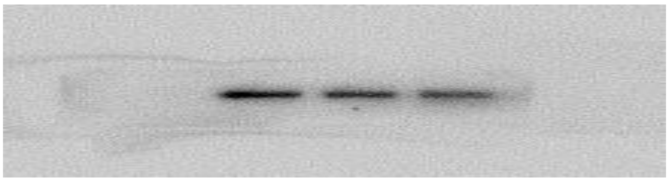

GAPDH:

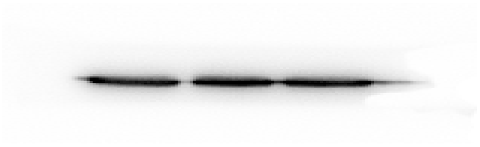

<u>HepG-2</u>

Atg-5:

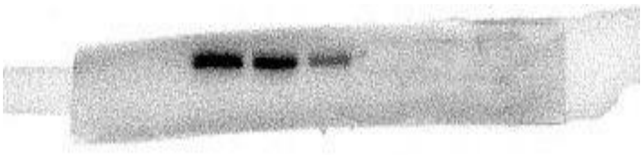

GAPDH:

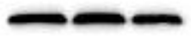

Supplementary Figure S6 Original gel images for Manuscript Fig. 7A

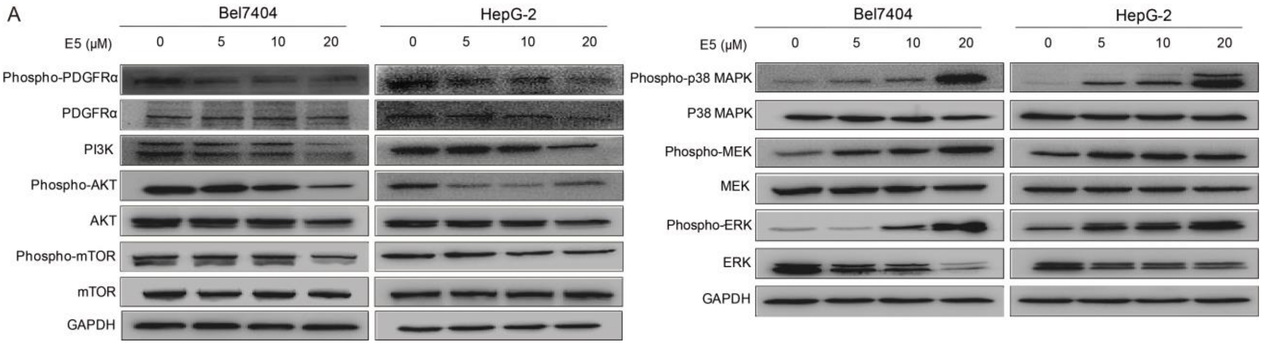

Bel7404

Phospho-PDGFRα:

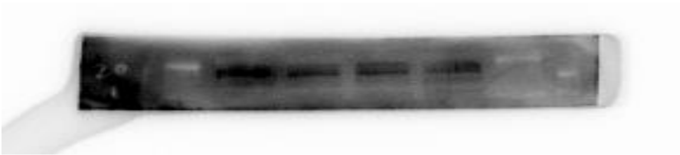

PDGFRα:

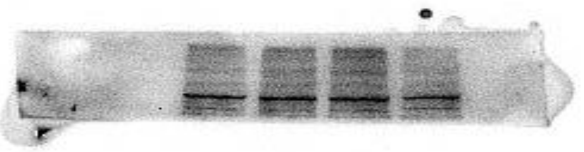

PI3K:

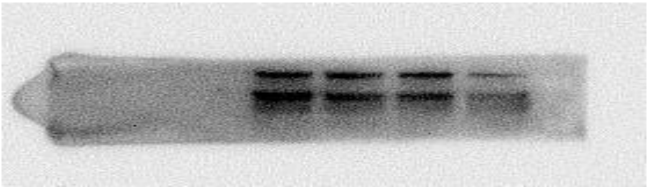

Phospho-AKT:

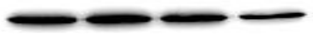

AKT:

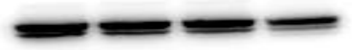

Phospho-mTOR:

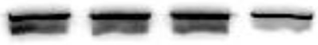

mTOR:

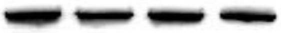

Phospho-p38 MAPK:

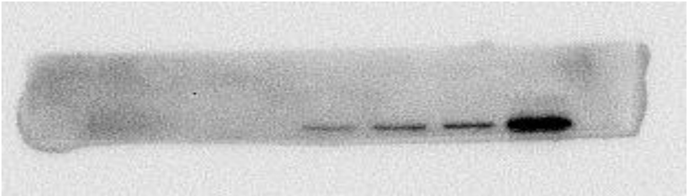

p38 MAPK:

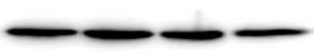

Phospho-MEK:

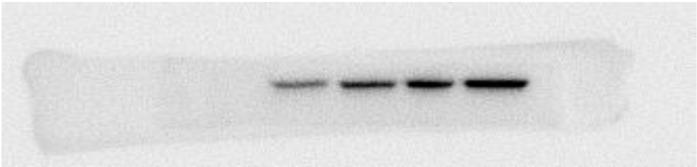

MEK:

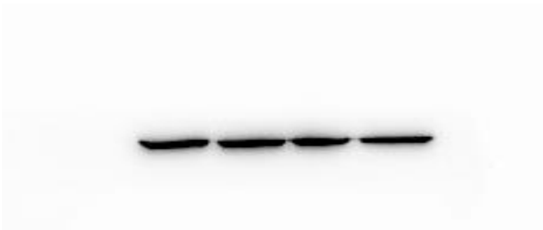

Phospho-ERK:

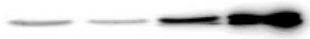

ERK:

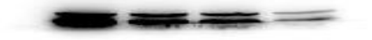

GAPDH:

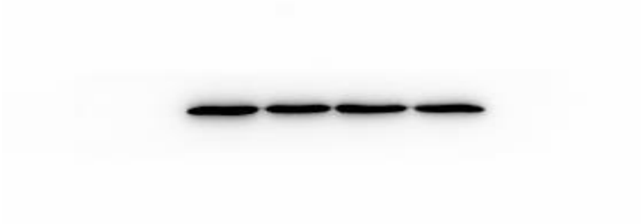

<u>HepG-2</u>

Phospho-PDGFRα:

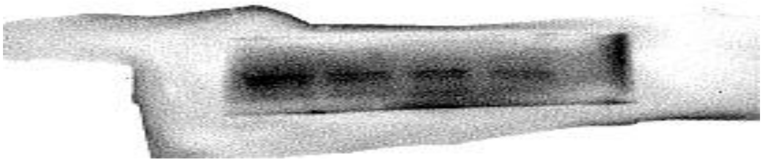

PDGFRα:

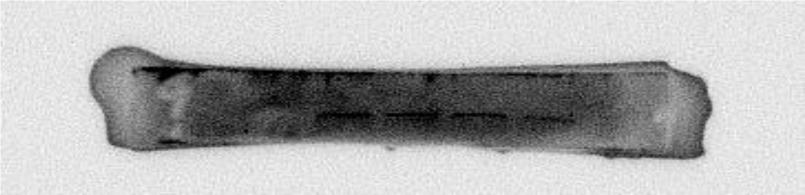

PI3K:

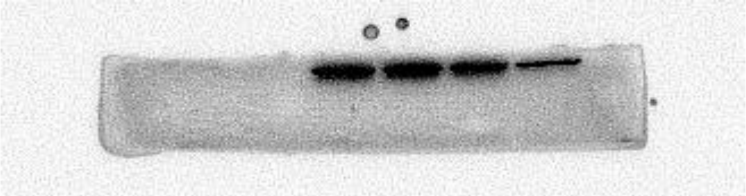

Phospho-AKT:

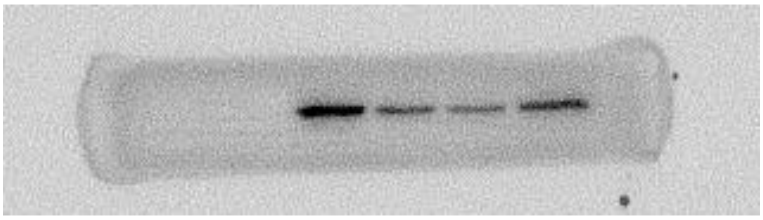

AKT:

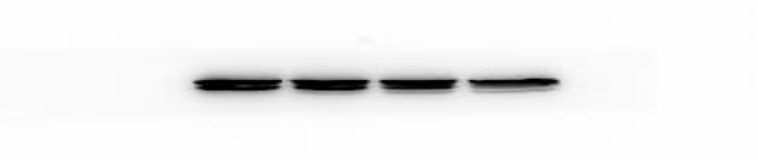

Phospho-mTOR:

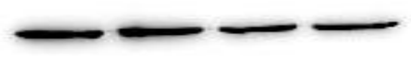

mTOR:

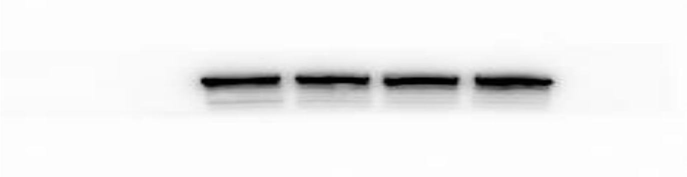

Phospho-p38 MAPK:

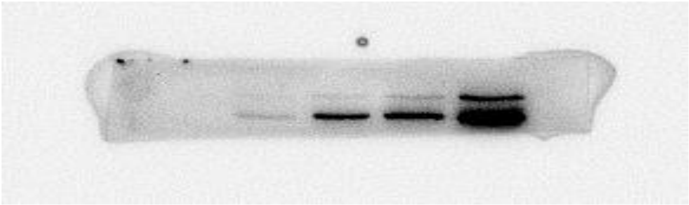

p38 MAPK:

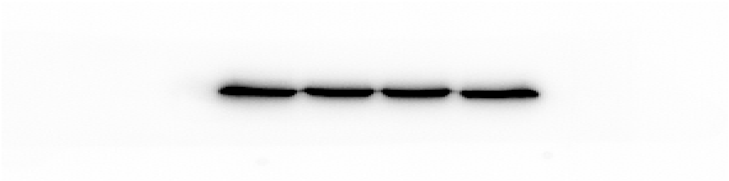

Phospho-MEK:

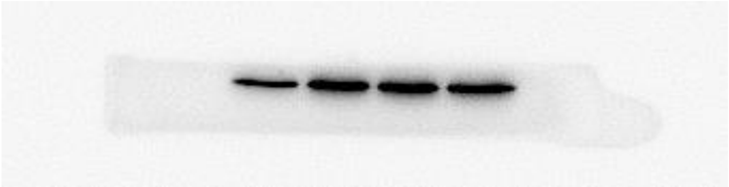

MEK:

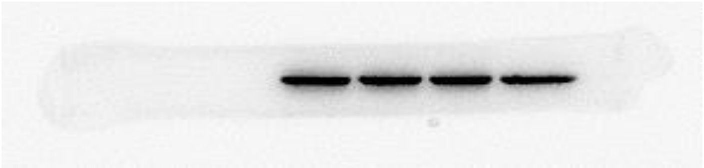

Phospho-ERK:

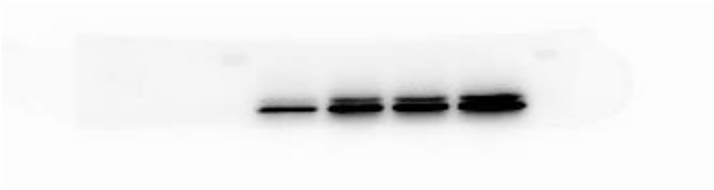

ERK:

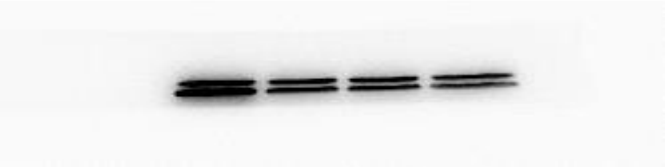

GAPDH:

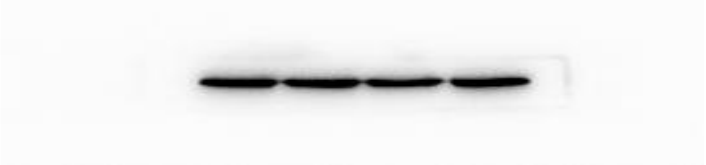

Supplementary Figure S7 Original gel images for Manuscript Fig. 7B

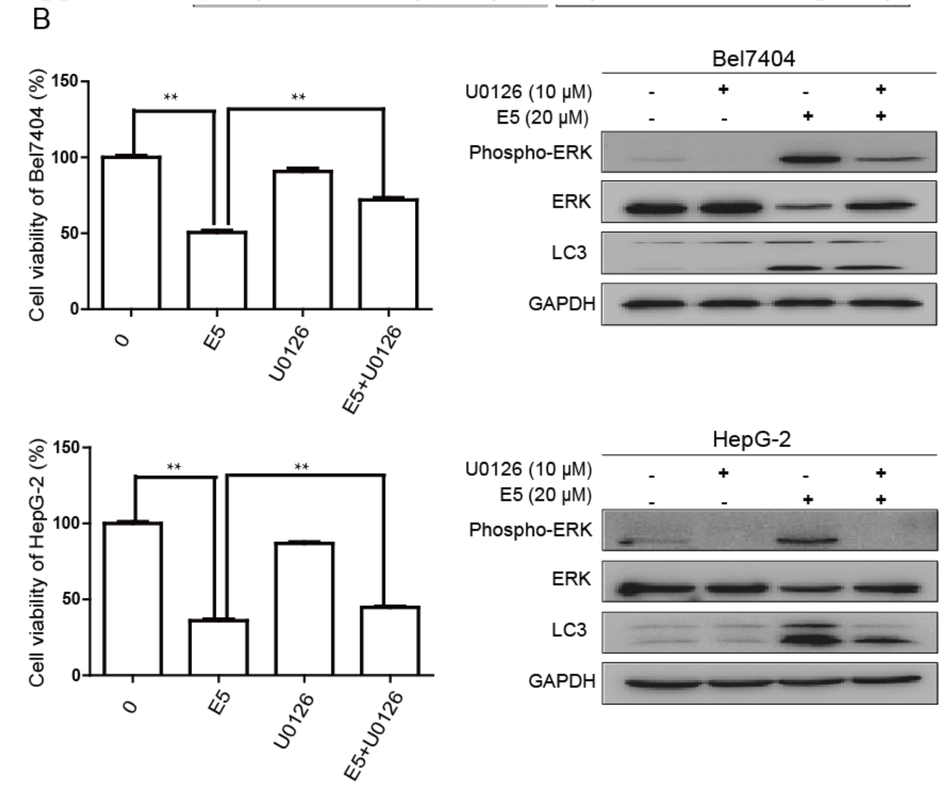

Bel7404

Phospho-ERK:

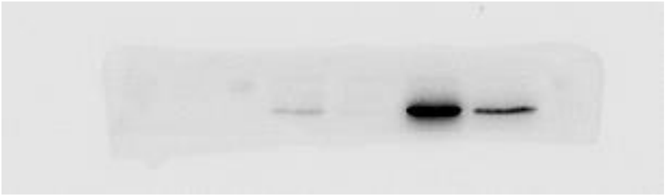

ERK:

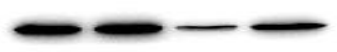

LC3 :

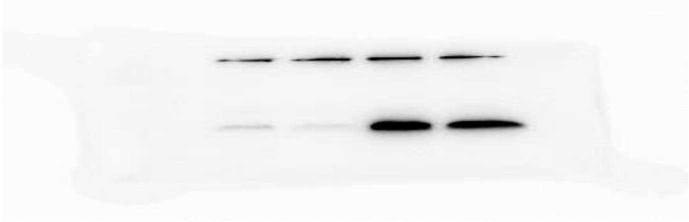

GAPDH:

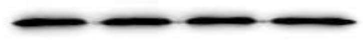

HepG-2

Phospho-ERK:

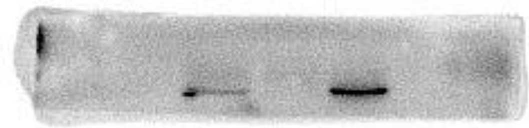

ERK:

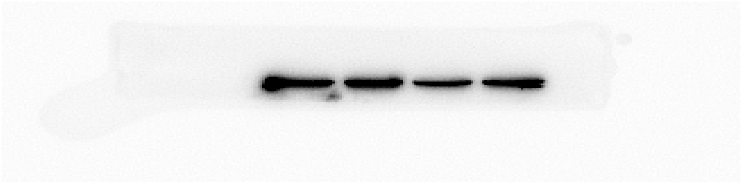

LC3 :

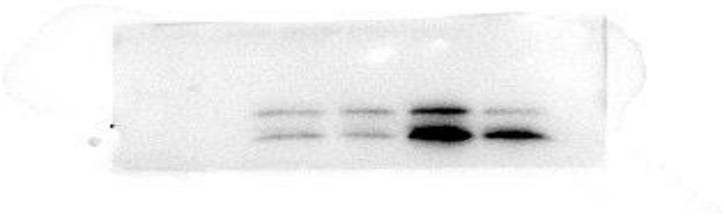

GAPDH:

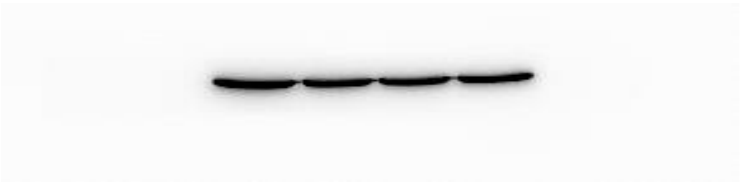

A1

SP1308303-2-C C13-NMR CDCL3 303K AV-300

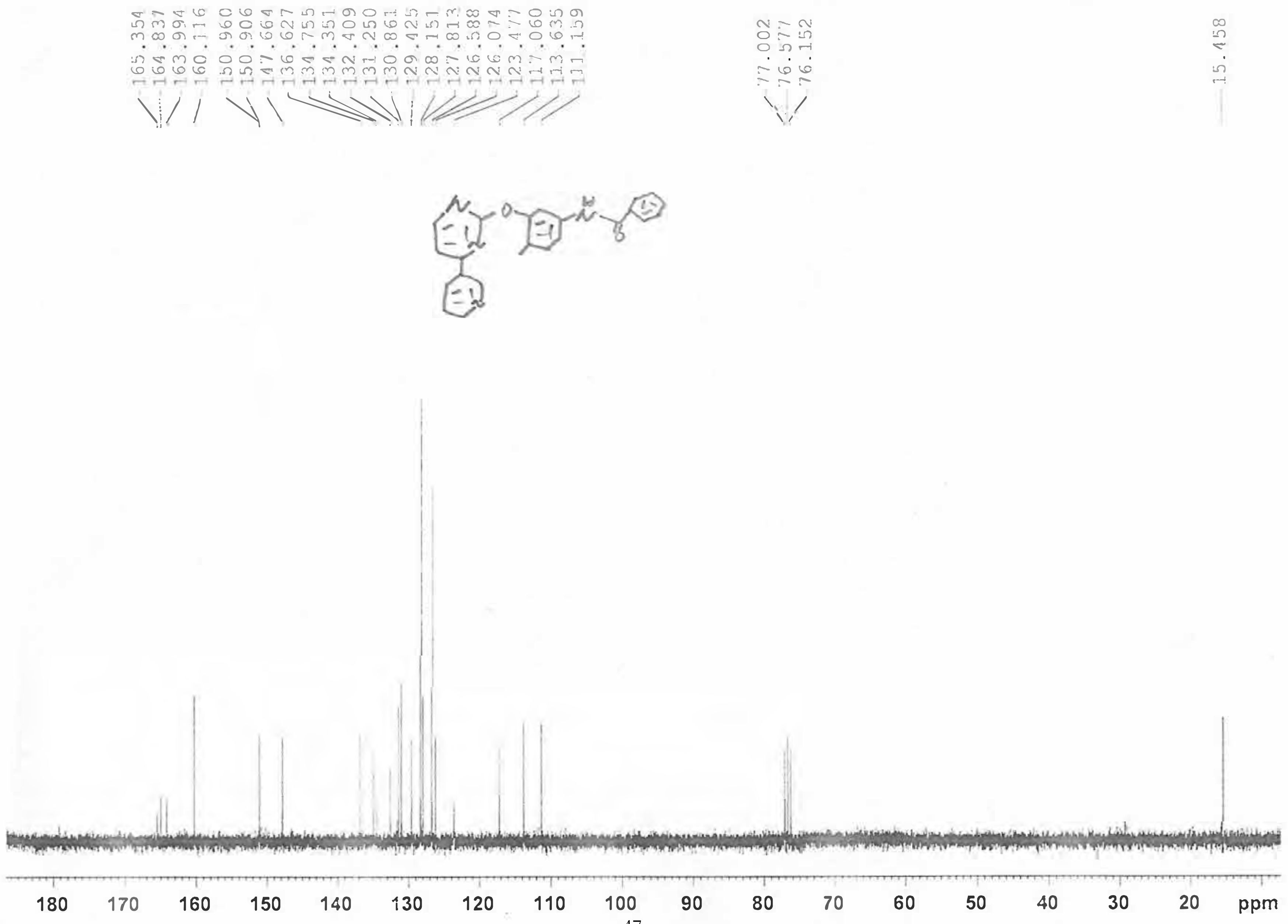

A1

SP121031-2 1H-NMR CDCl3 303K AV-300

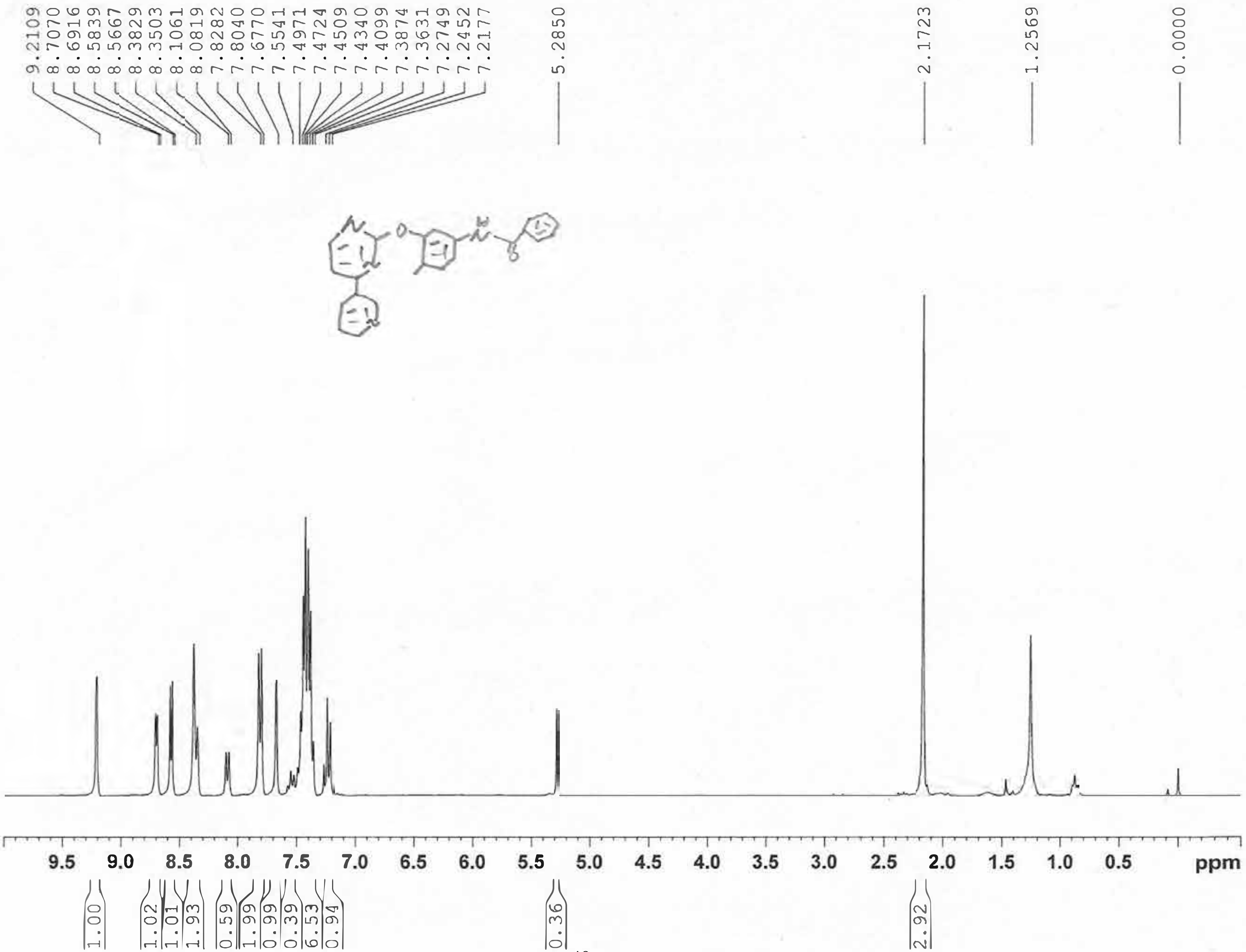

**Sample Name**		**Position**	p2d6	**Instrument Name**	Instrument 1
**User Name**	QTOF-PC\QTOF	**Inj Vol**	0.2	**InjPosition**	
**Sample Type**	Sample	**IRM Calibration Status**	Success	**Data Filename**	A1-p.d
**ACQ Method**	20110418-MSonly-p.m	**Comment**		**Acquired Time**	3/12/2018 2:50:16 PM

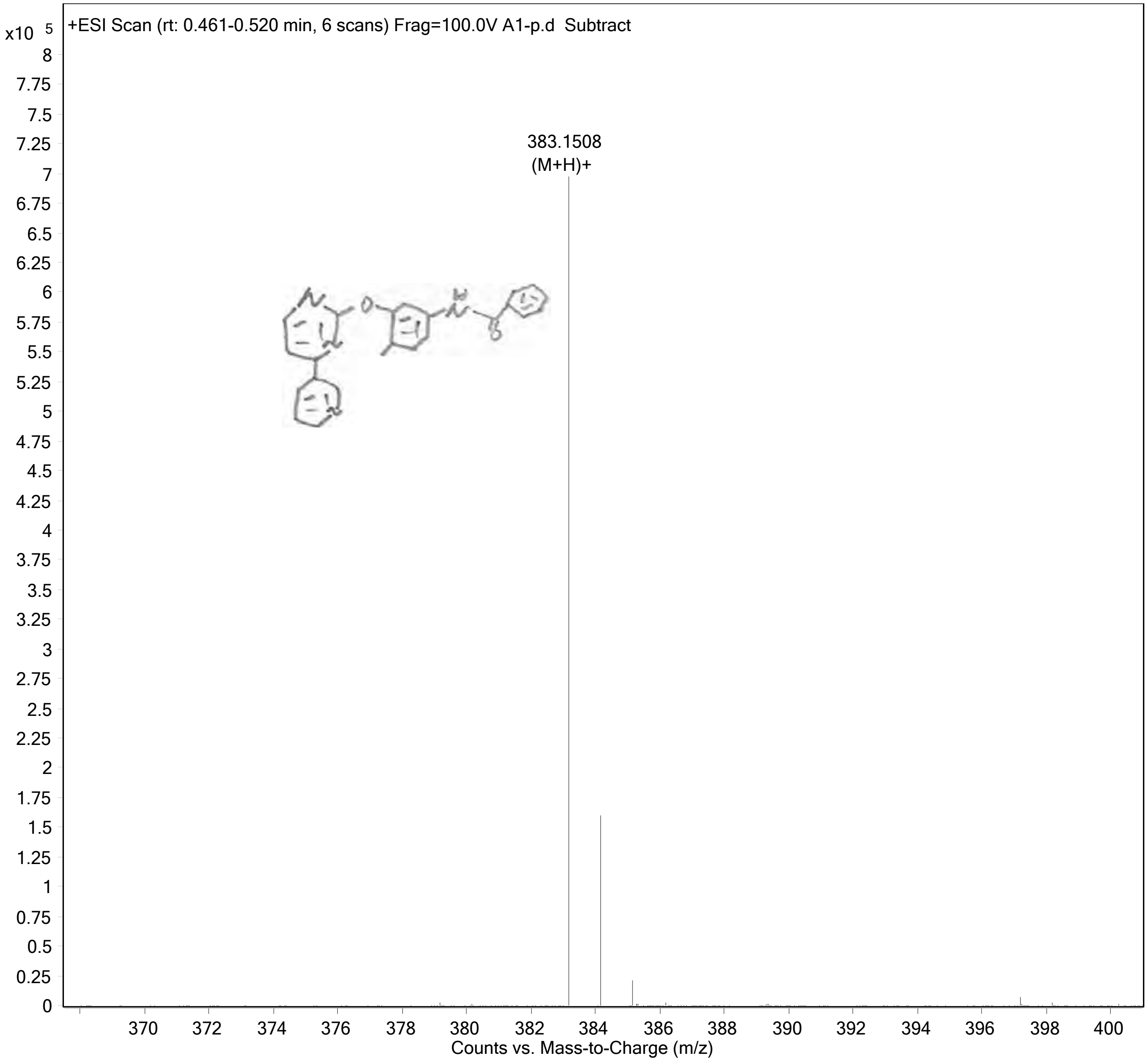

A 2

SP121126 CDCl3 303K C13-NMR AV300

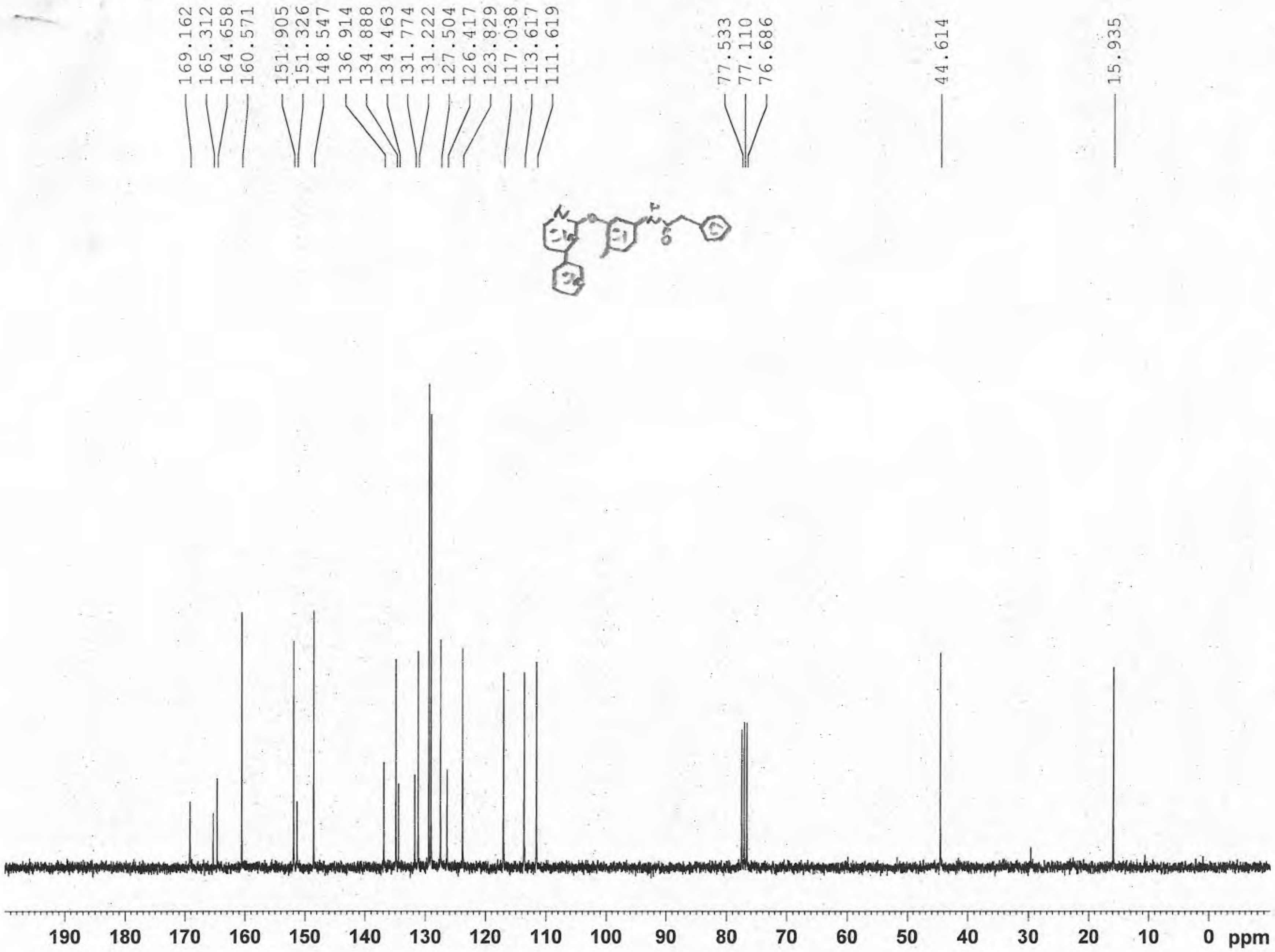

A2

SP121109-3 1H-NMR CDCl3 303K AV-300

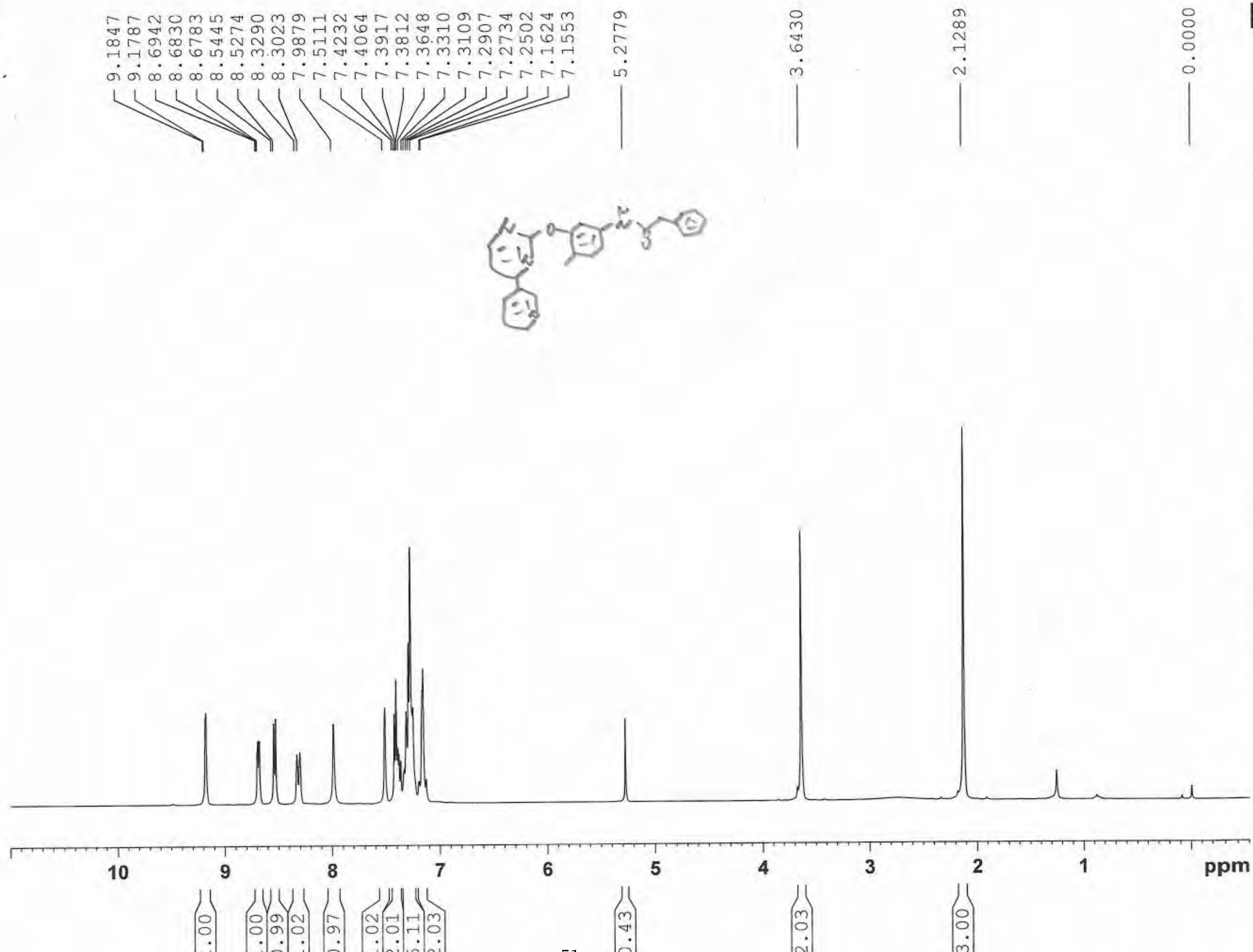

**Sample Name**		**Position**	p2d7	**Instrument Name**	Instrument 1
**User Name**	QTOF-PC\QTOF	**Inj Vol**	0.2	**InjPosition**	
**Sample Type**	Sample	**IRM Calibration Status**	Success	**Data Filename**	A2-p.d
**ACQ Method**	20110418-MSonly-p.m	**Comment**		**Acquired Time**	3/12/2018 2:52:45 PM

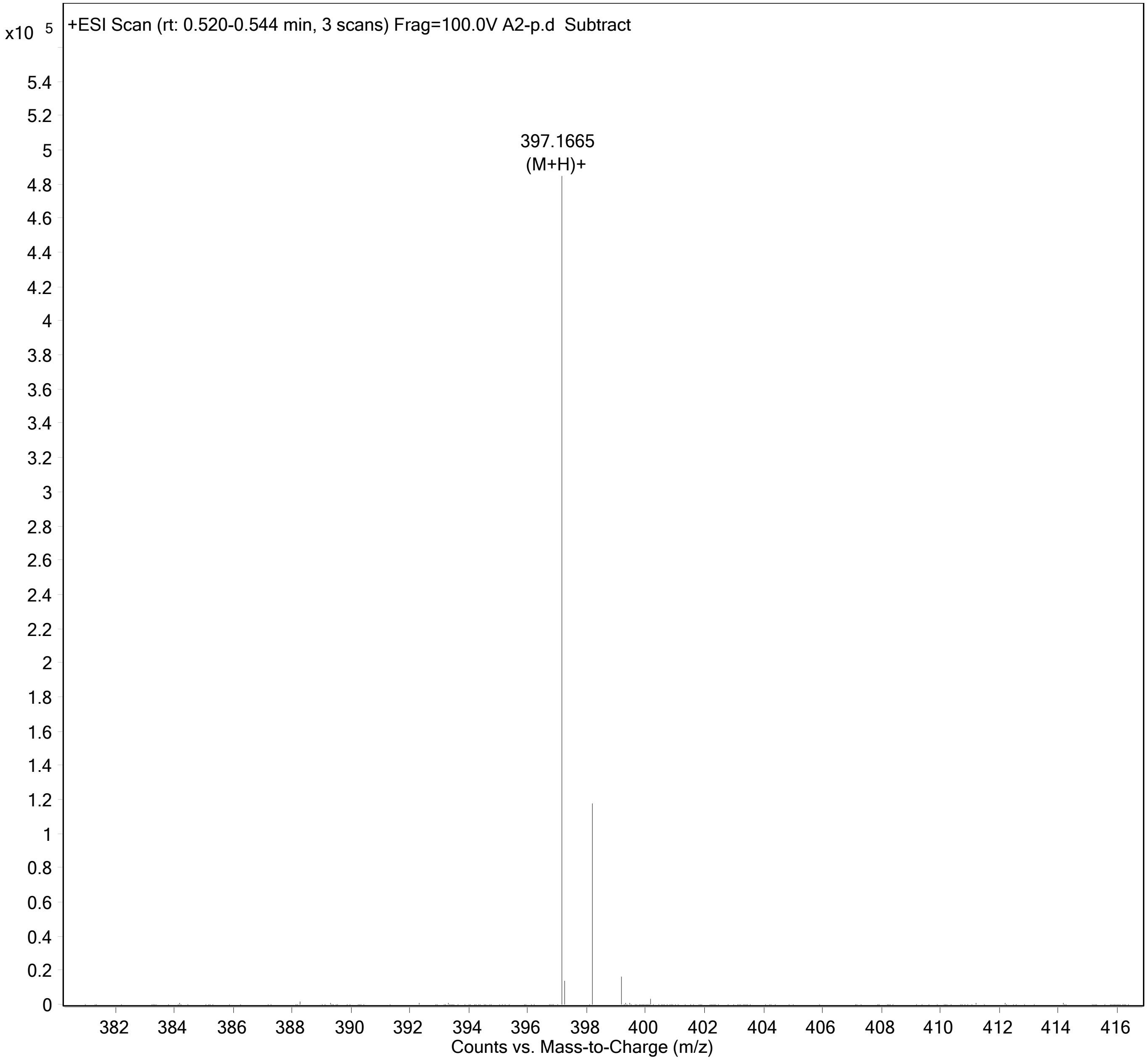

A3

SP121128-2 CDCl3 303K C13-NMR AV300

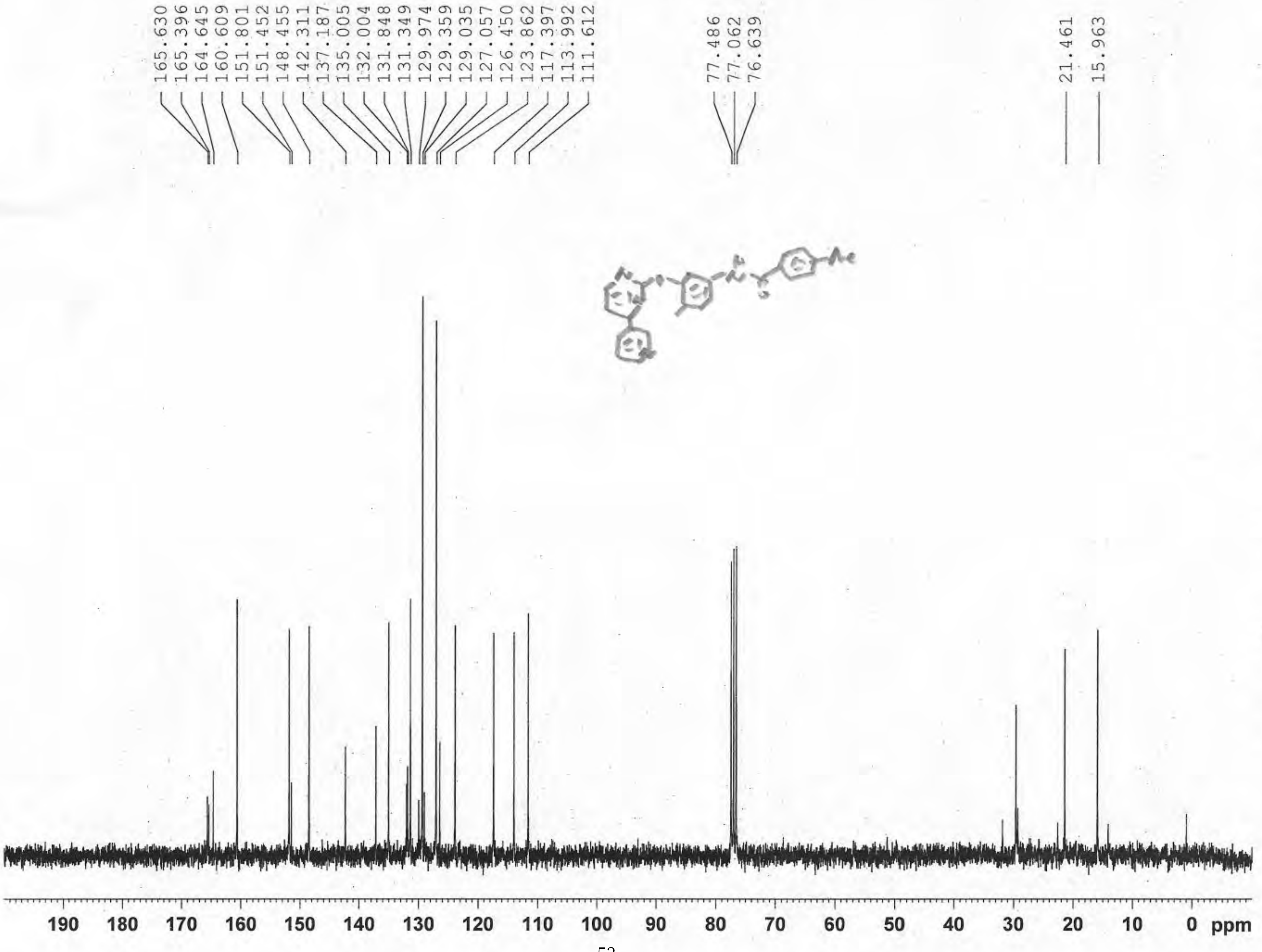

A3

SP121107-1 1H-NMR CDCl3 303K AV-300

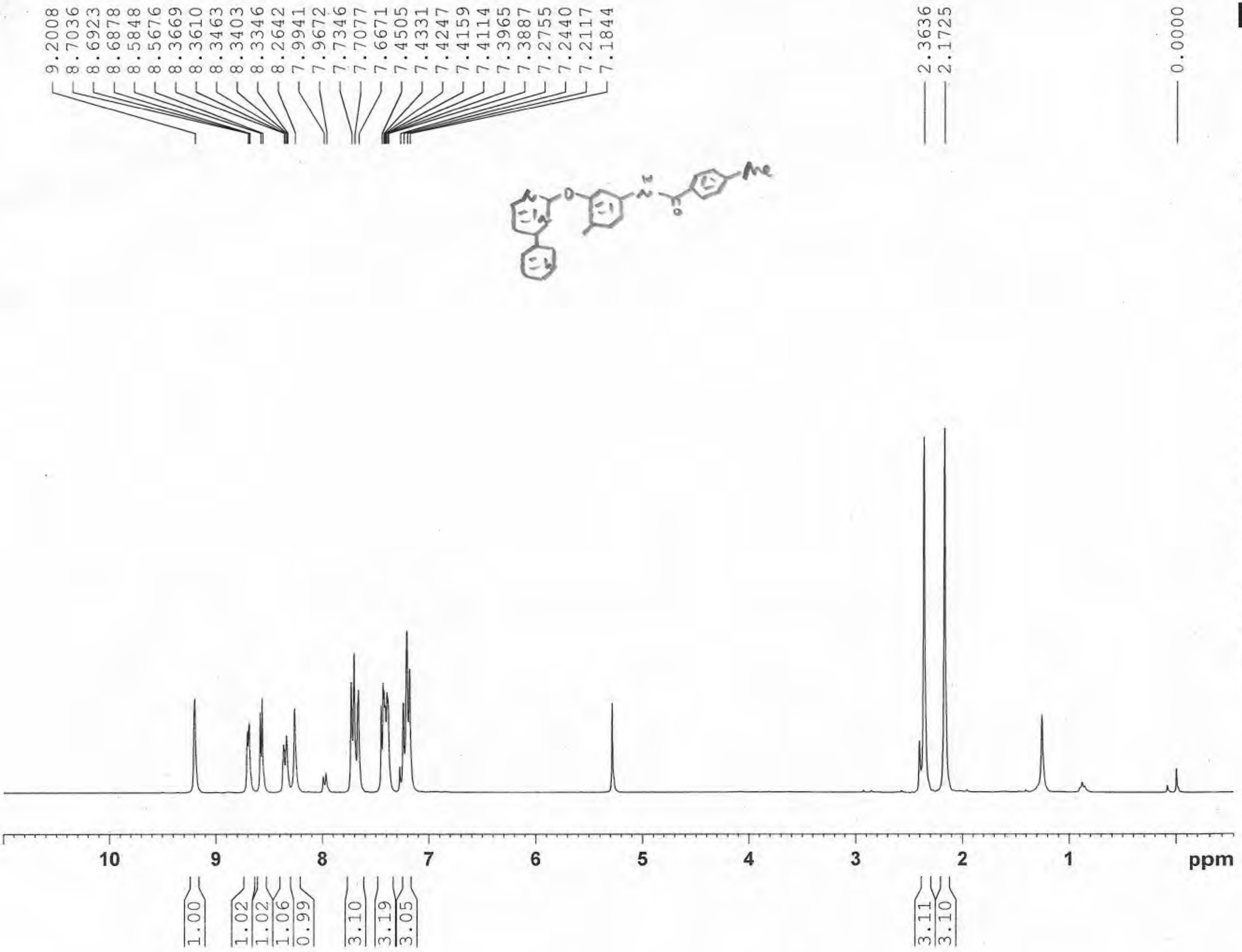

**Sample Name**		**Position**	p2d8	**Instrument Name**	Instrument 1
**User Name**	QTOF-PC\QTOF	**Inj Vol**	0.1	**InjPosition**	
**Sample Type**	Sample	**IRM Calibration Status**	Success	**Data Filename**	A3-p.d
**ACQ Method**	20110418-MSonly-p.m	**Comment**		**Acquired Time**	3/12/2018 2:54:56 PM

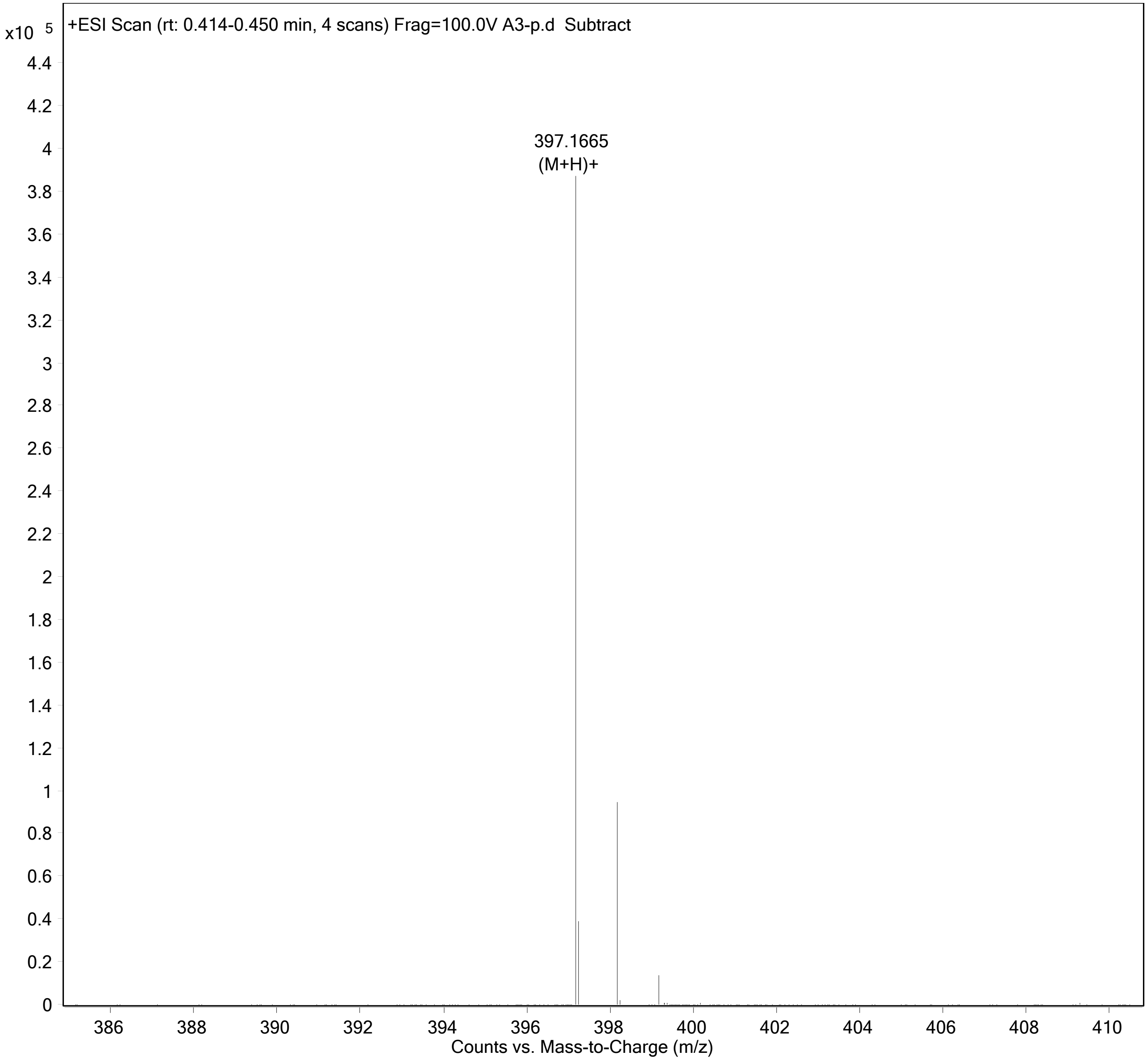

A 4

SP121212-1 CDCl3 303K C13-NMR AV300

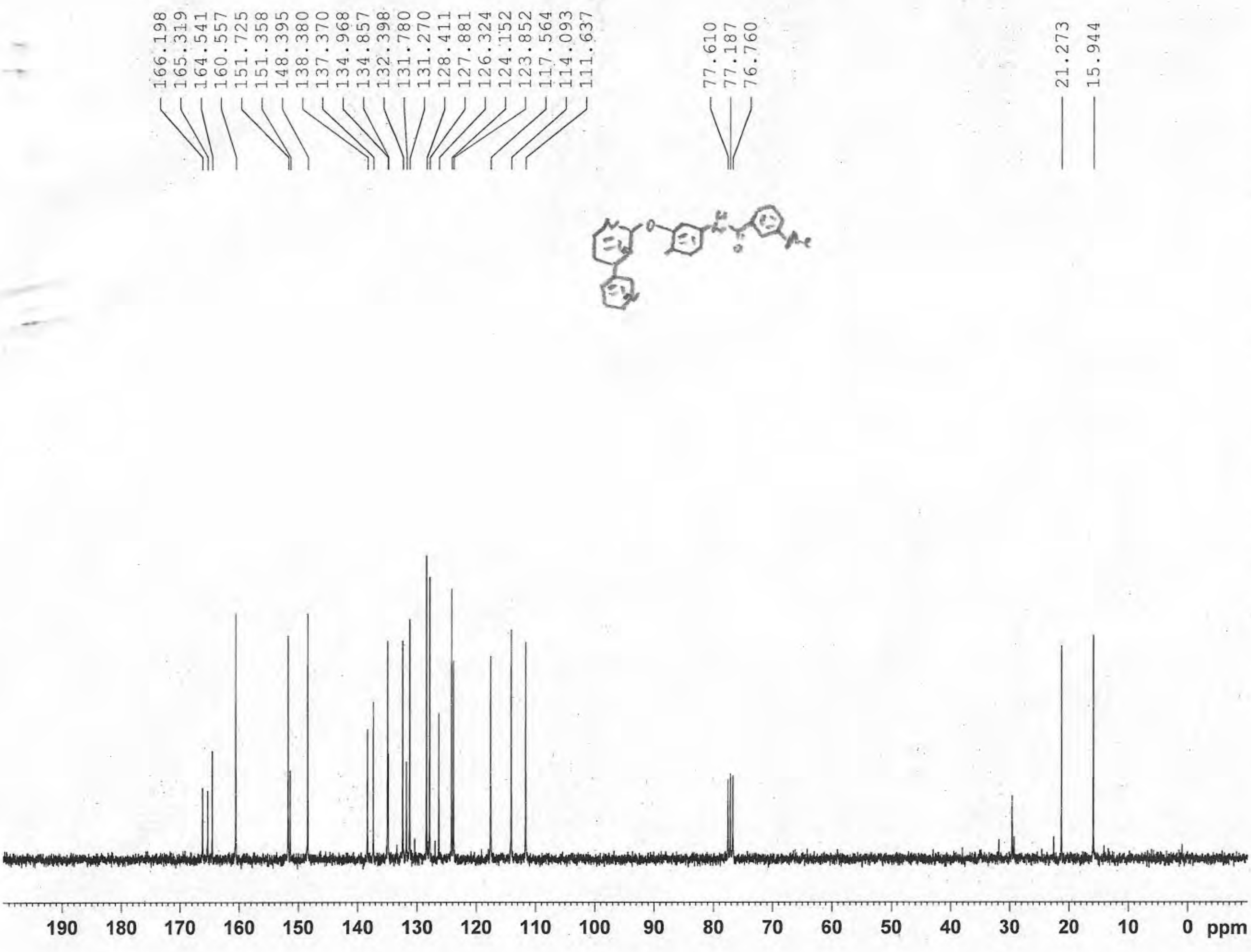

A4

SP121212-1 1H-NMR CDCl3 303K AV-300

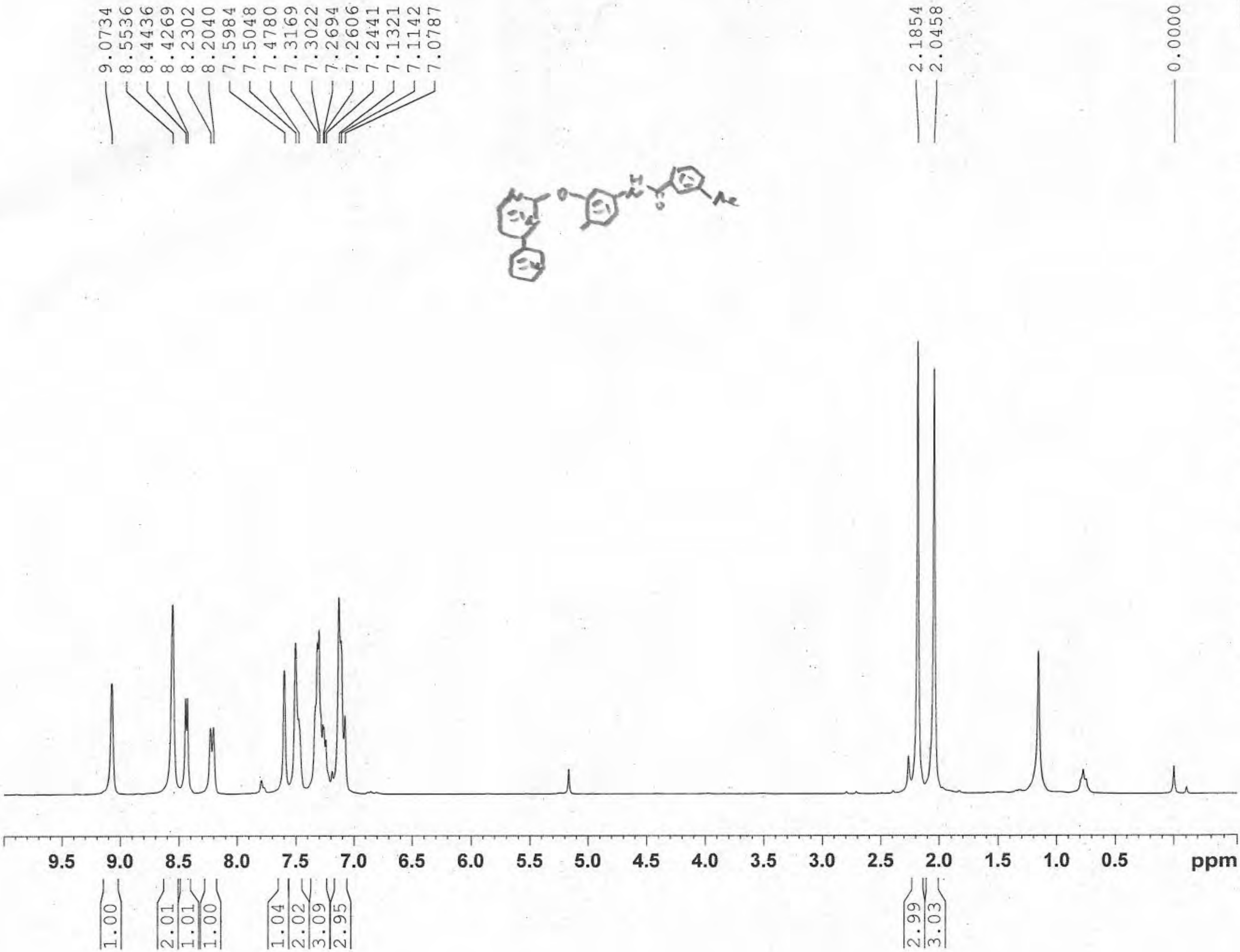

**Sample Name**		**Position**	p2d9	**Instrument Name**	Instrument 1
**User Name**	QTOF-PC\QTOF	**Inj Vol**	0.1	**InjPosition**	
**Sample Type**	Sample	**IRM Calibration Status**	Success	**Data Filename**	A4-p.d
**ACQ Method**	20110418-MSonly-p.m	**Comment**		**Acquired Time**	3/12/2018 2:57:46 PM

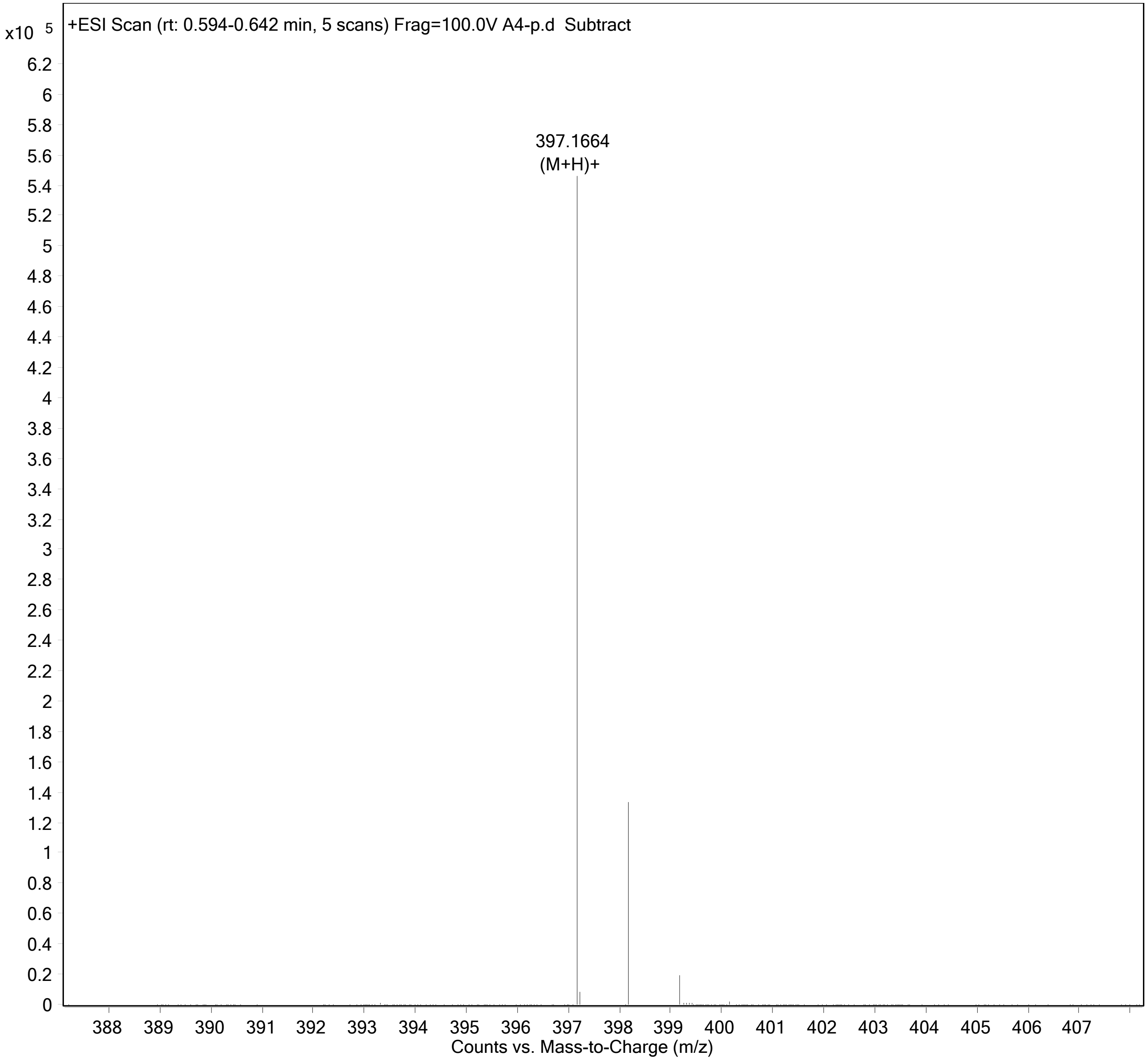

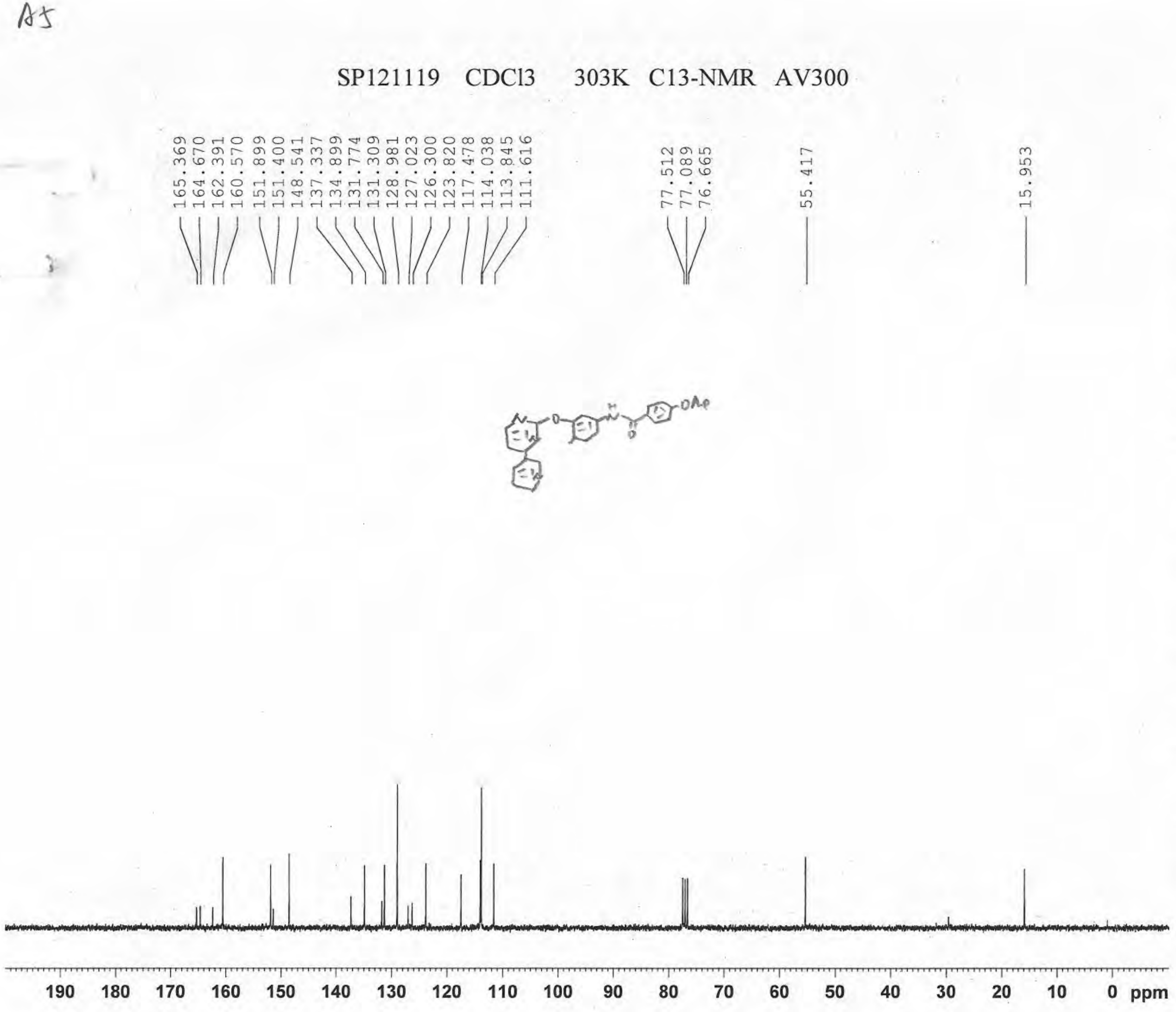

SP121119 CDCl3 303K C13-NMR AV300
165.369
164.670
162.391
160.570
151.899
151.400
148.541
137.337
134.899
131.774
131.309
128.981
127.023
126.300
123.820
117.478
114.038
113.845
111.616
77.512
77.089
76.665
55.417
15.953
190 180 170 160 150 140 130 120 110 100 90 80 70 60 50 40 30 20 10 0 ppm

SP121105-2 1H-NMR CDCl3 303K AV-300

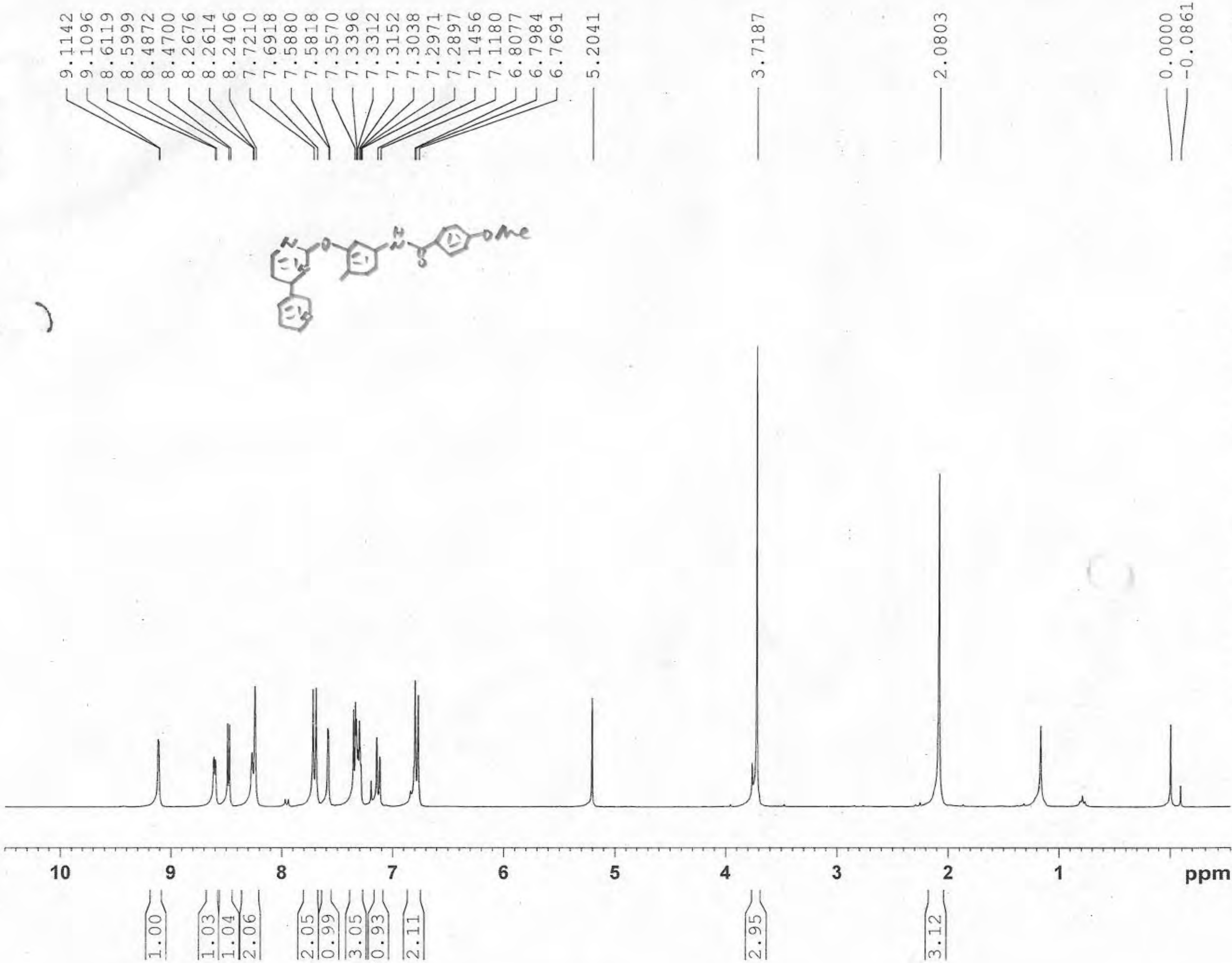

**Sample Name**		**Position**	p2E1	**Instrument Name**	Instrument 1
**User Name**	QTOF-PC\QTOF	**Inj Vol**	0.1	**InjPosition**	
**Sample Type**	Sample	**IRM Calibration Status**	Success	**Data Filename**	A5-p.d
**ACQ Method**	20110418-MSonly-p.m	**Comment**		**Acquired Time**	3/12/2018 3:00:14 PM

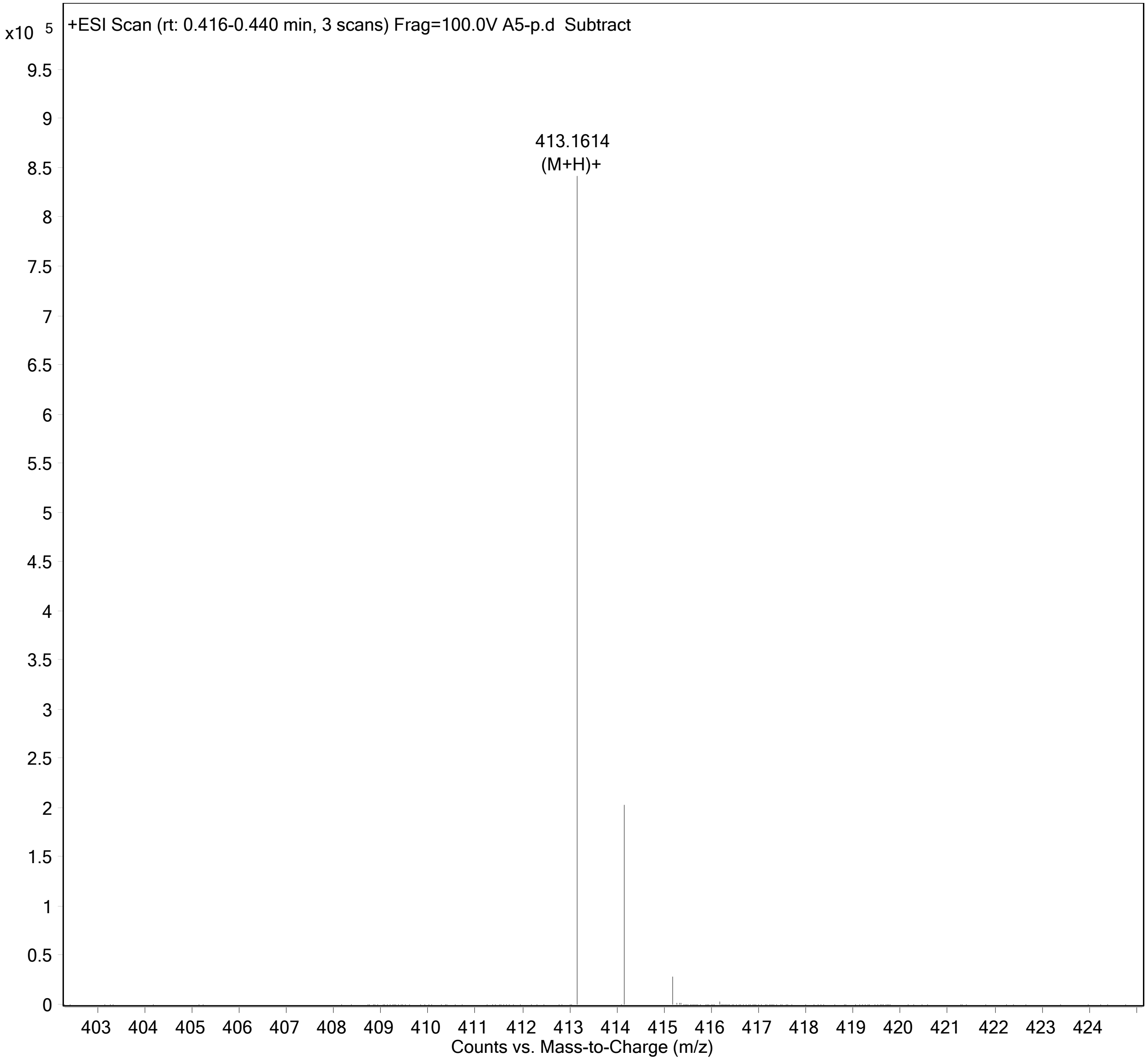

A6.

SP121205-1 CDCl3 303K C13-NMR AV300

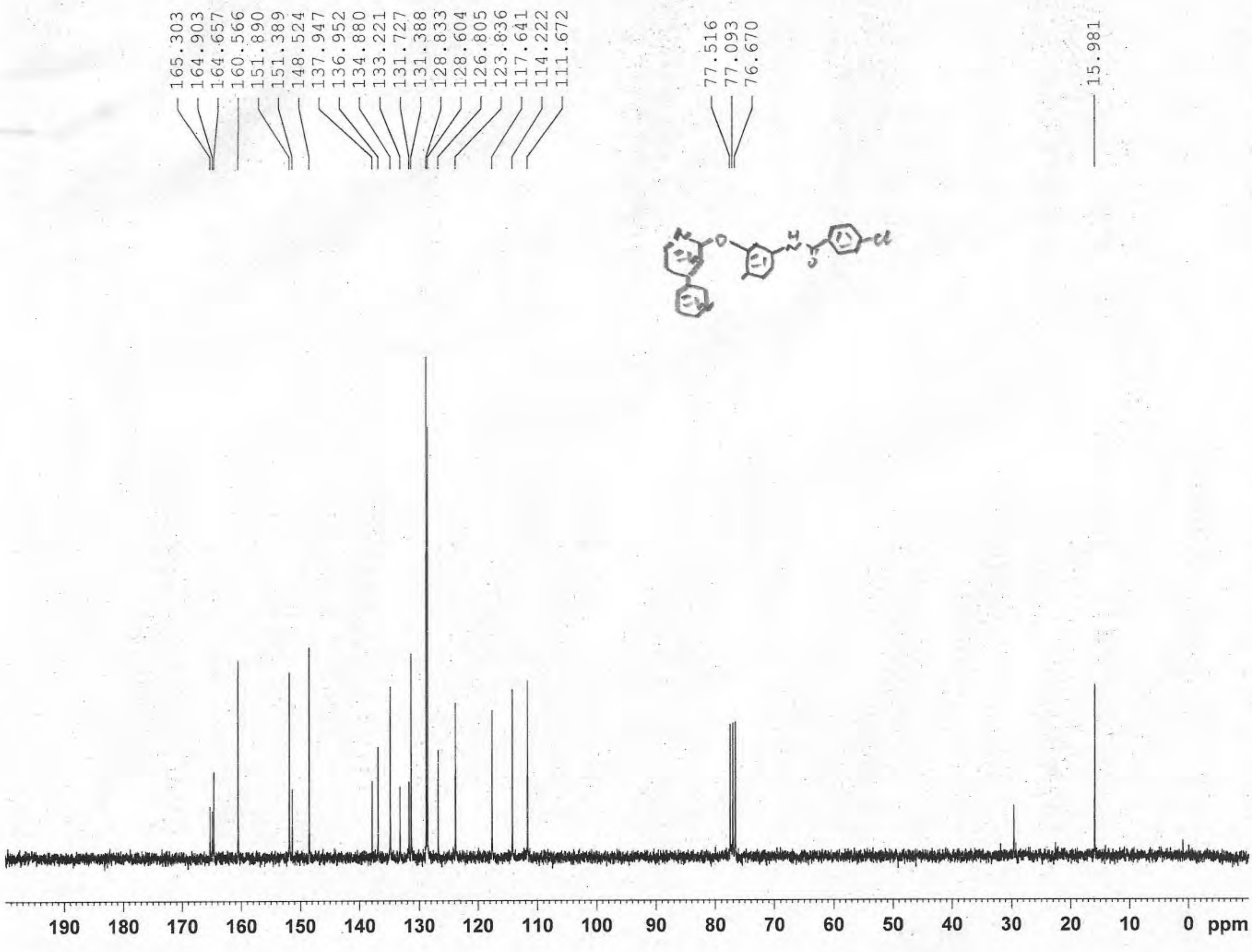

A6

SP121114-3 1HNMR CDCl3 303K AV-300

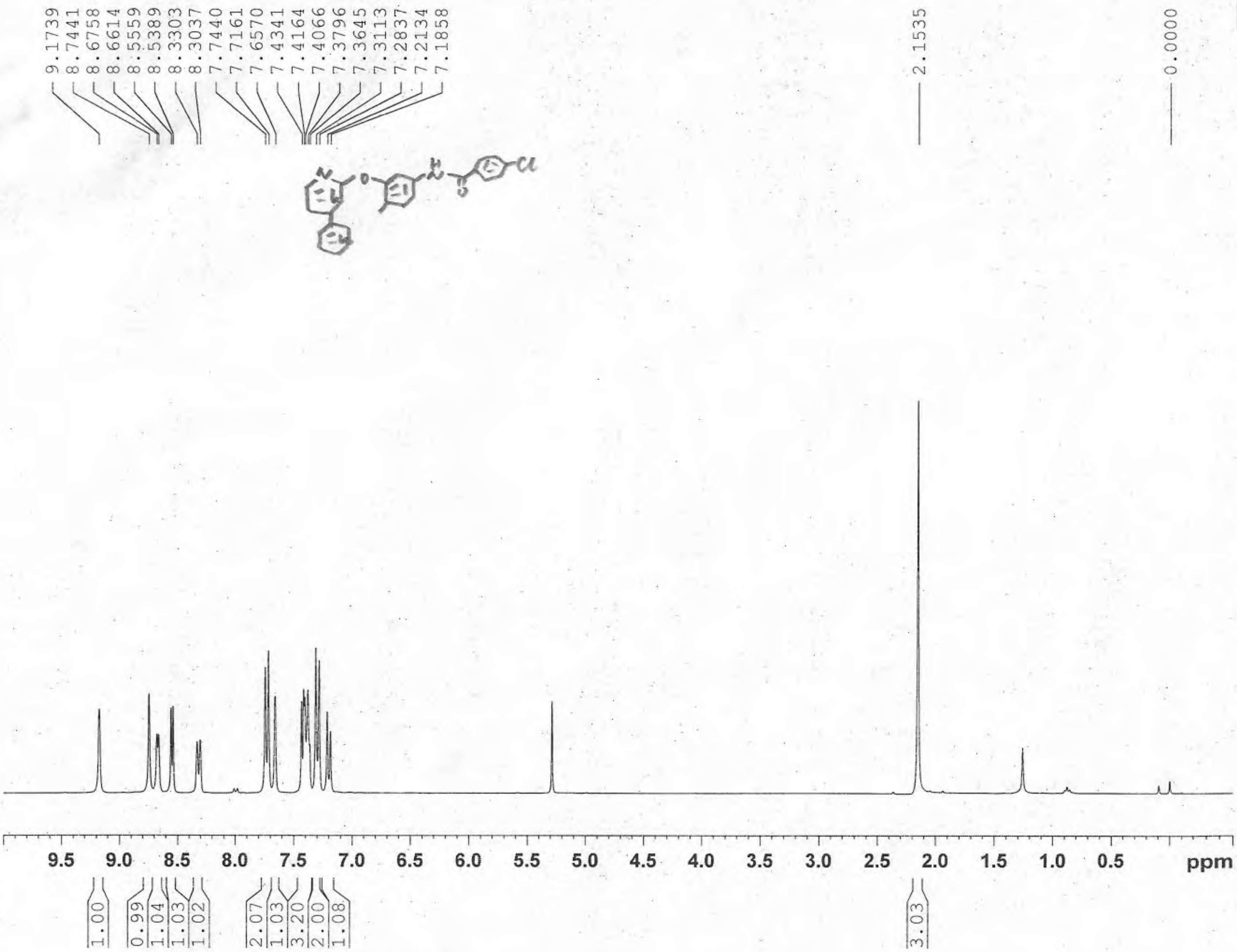

**Sample Name**		**Position**	p2E2	**Instrument Name**	Instrument 1
**User Name**	QTOF-PC\QTOF	**Inj Vol**	0.1	**InjPosition**	
**Sample Type**	Sample	**IRM Calibration Status**	Success	**Data Filename**	A6-p.d
**ACQ Method**	20110418-MSonly-p.m	**Comment**		**Acquired Time**	3/12/2018 3:02:19 PM

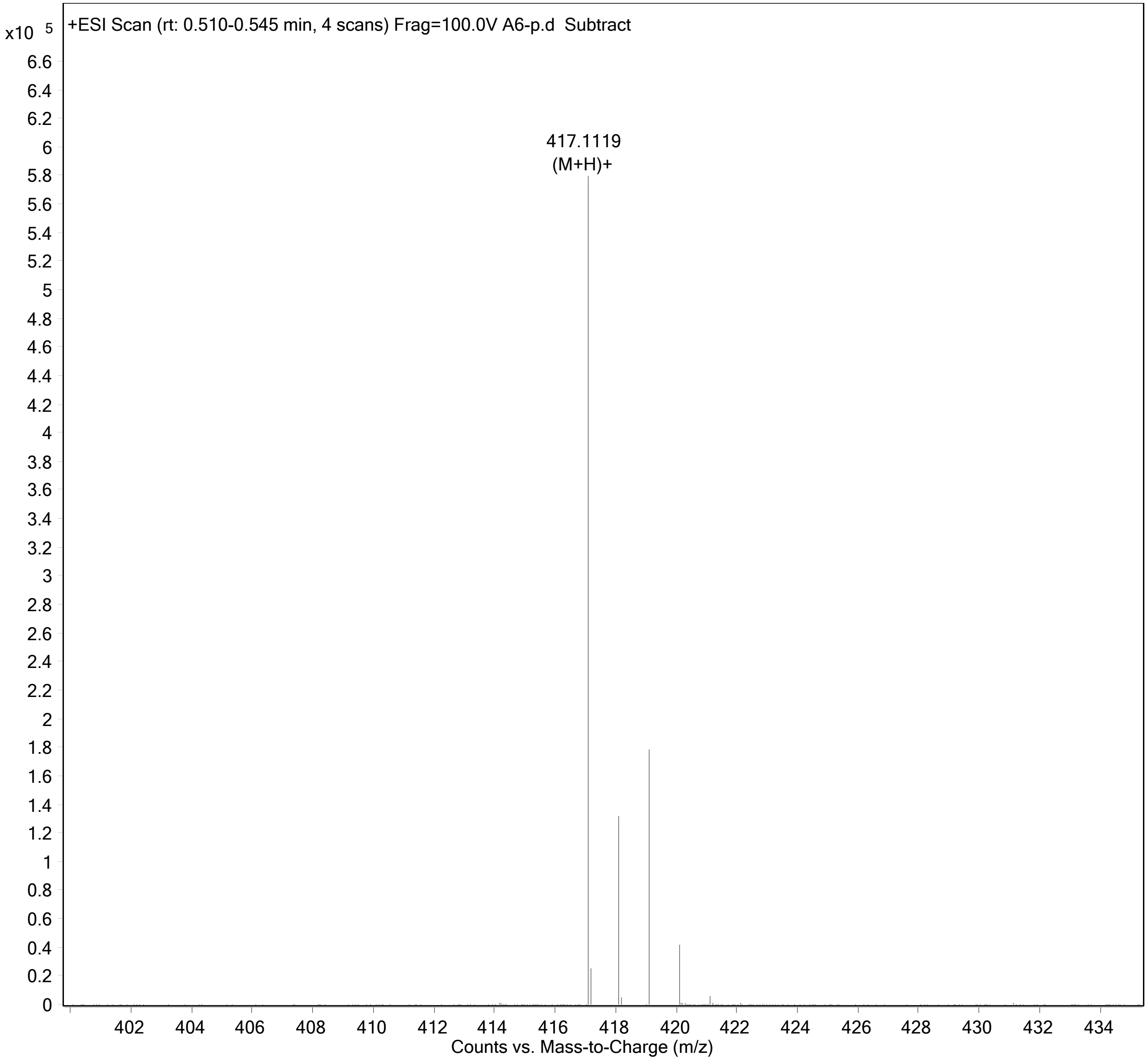

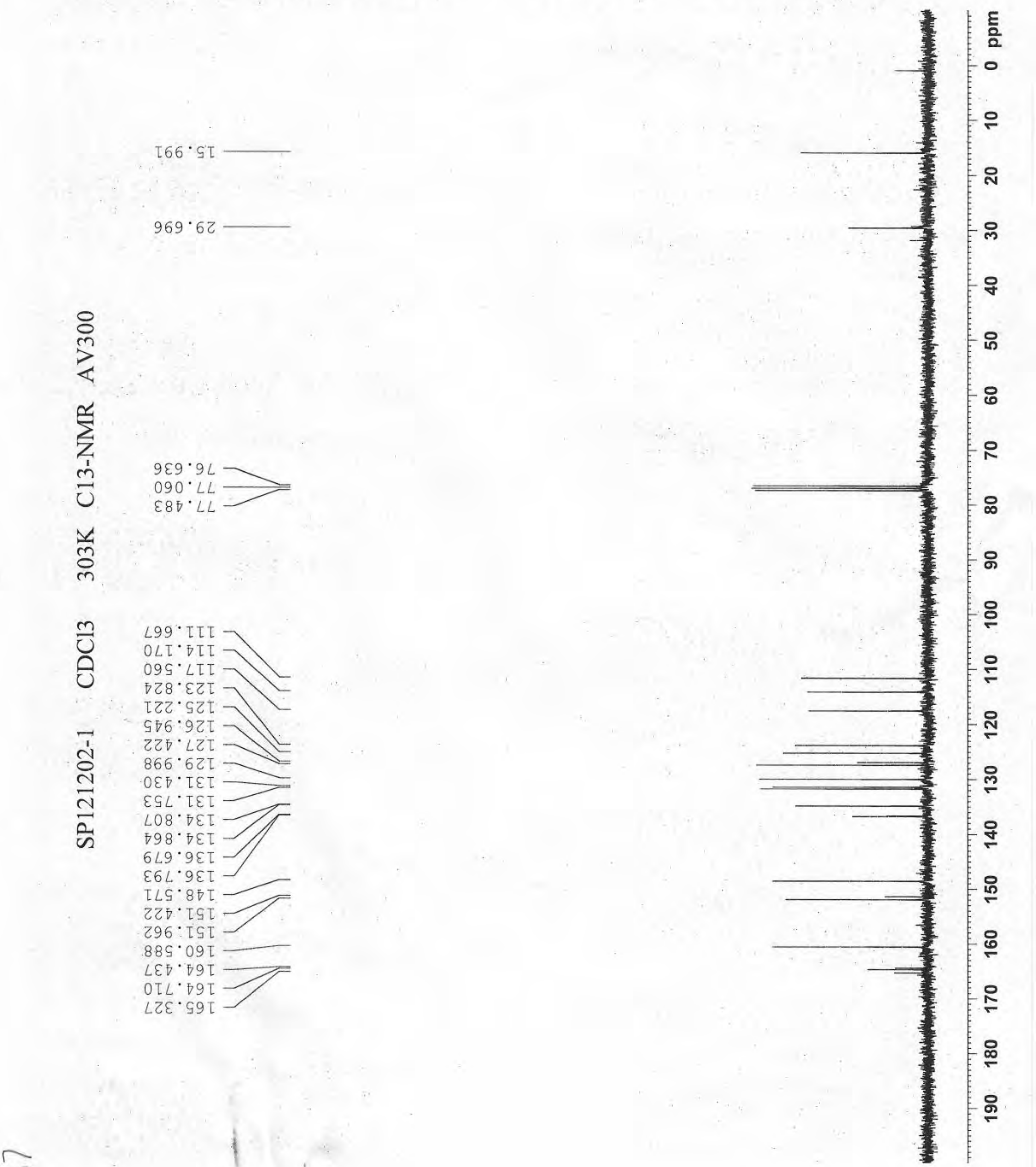
A7
SP121202-1 CDCl3 303K C13-NMR AV300
165.327
164.710
164.437
160.588
151.962
151.422
148.571
136.793
136.679
134.864
134.807
131.753
131.430
129.998
127.422
126.945
125.221
123.824
117.560
114.170
111.667
77.483
77.060
76.636
29.696
15.991
190 180 170 160 150 140 130 120 110 100 90 80 70 60 50 40 30 20 10 0 ppm

A7

SP121114-2 1HNMR CDCl3 303K AV-300

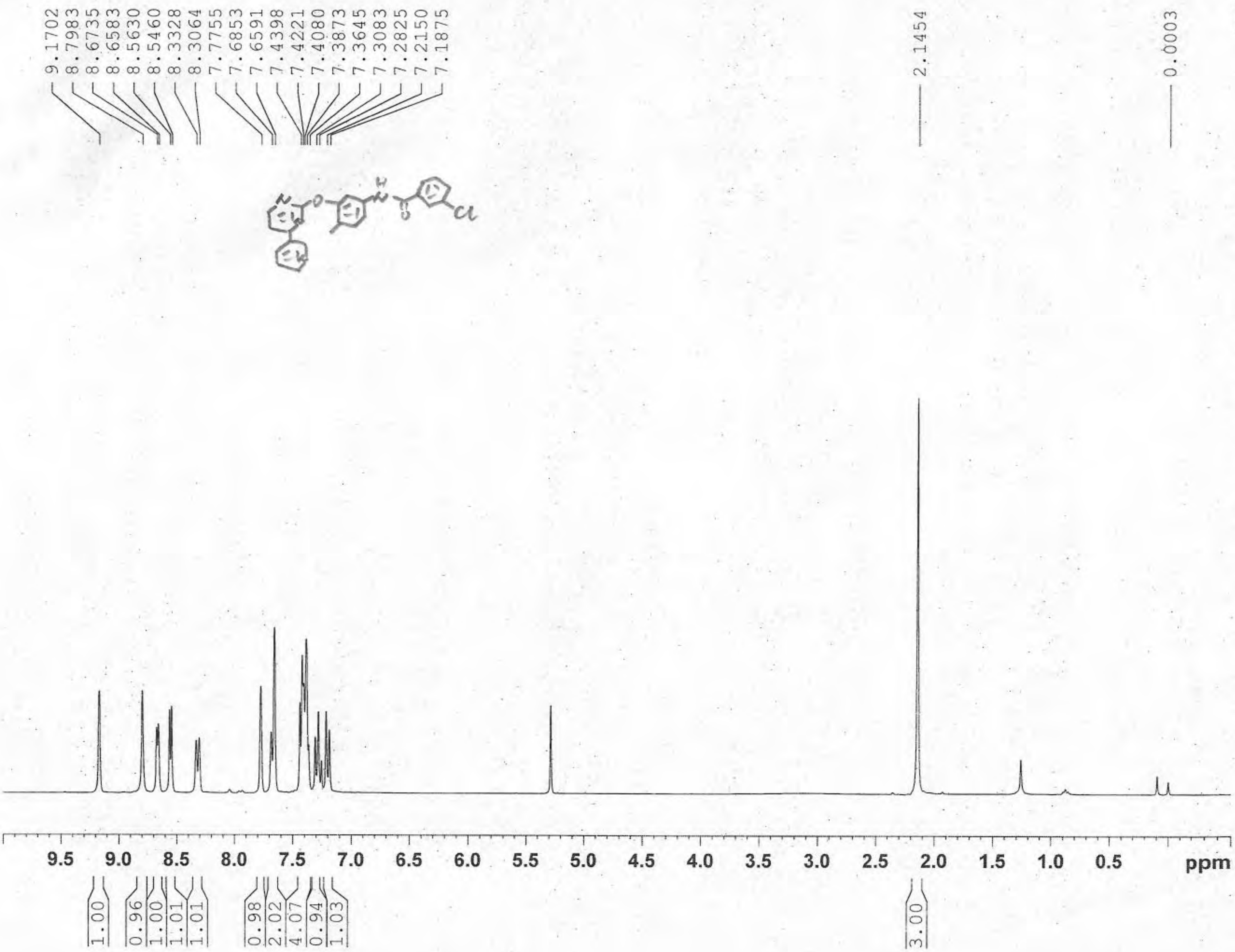

**Sample Name**		**Position**	p2E3	**Instrument Name**	Instrument 1
**User Name**	QTOF-PC\QTOF	**Inj Vol**	0.1	**InjPosition**	
**Sample Type**	Sample	**IRM Calibration Status**	Success	**Data Filename**	A7-p.d
**ACQ Method**	20110418-MSonly-p.m	**Comment**		**Acquired Time**	3/12/2018 3:04:19 PM

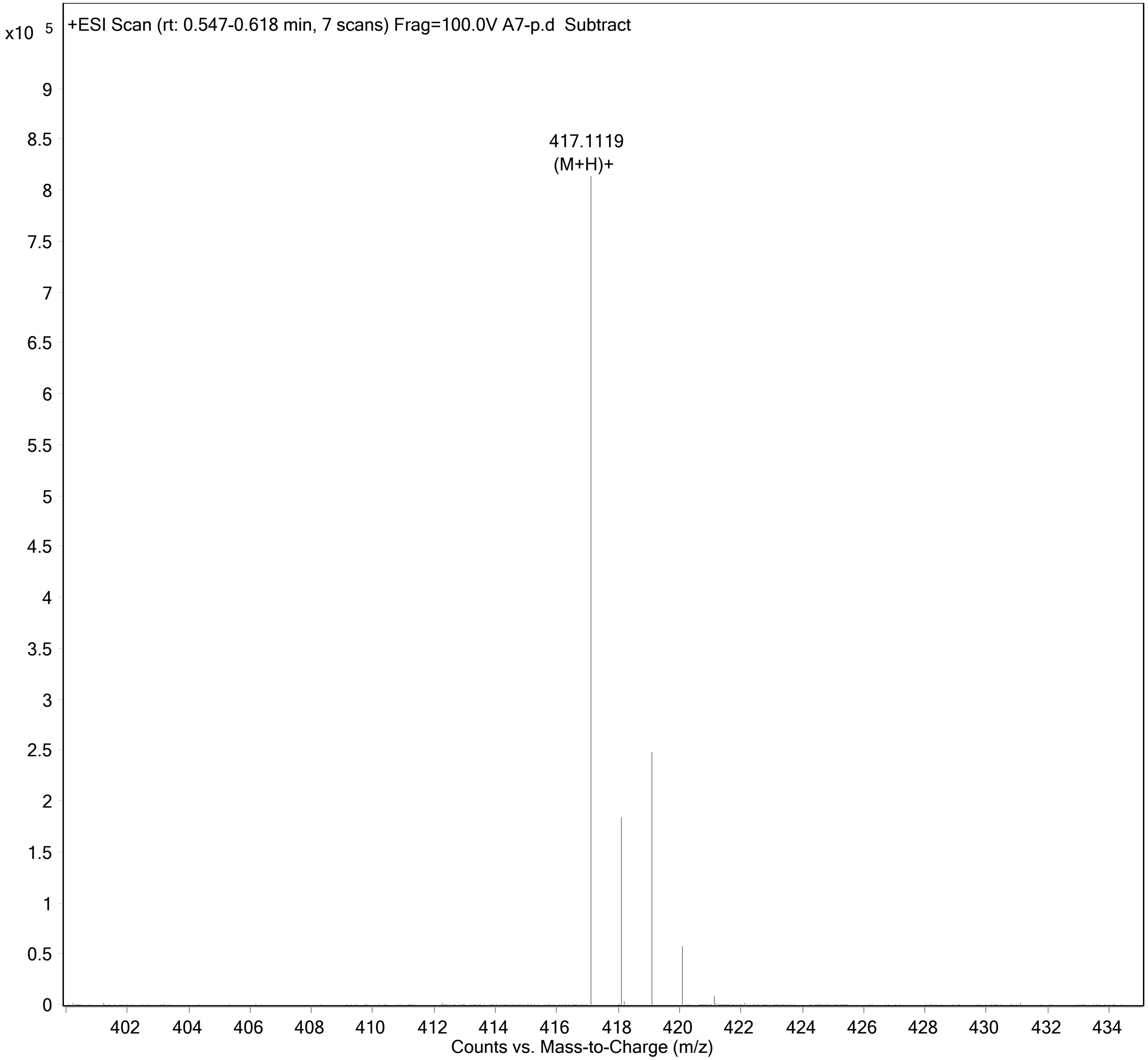

A8

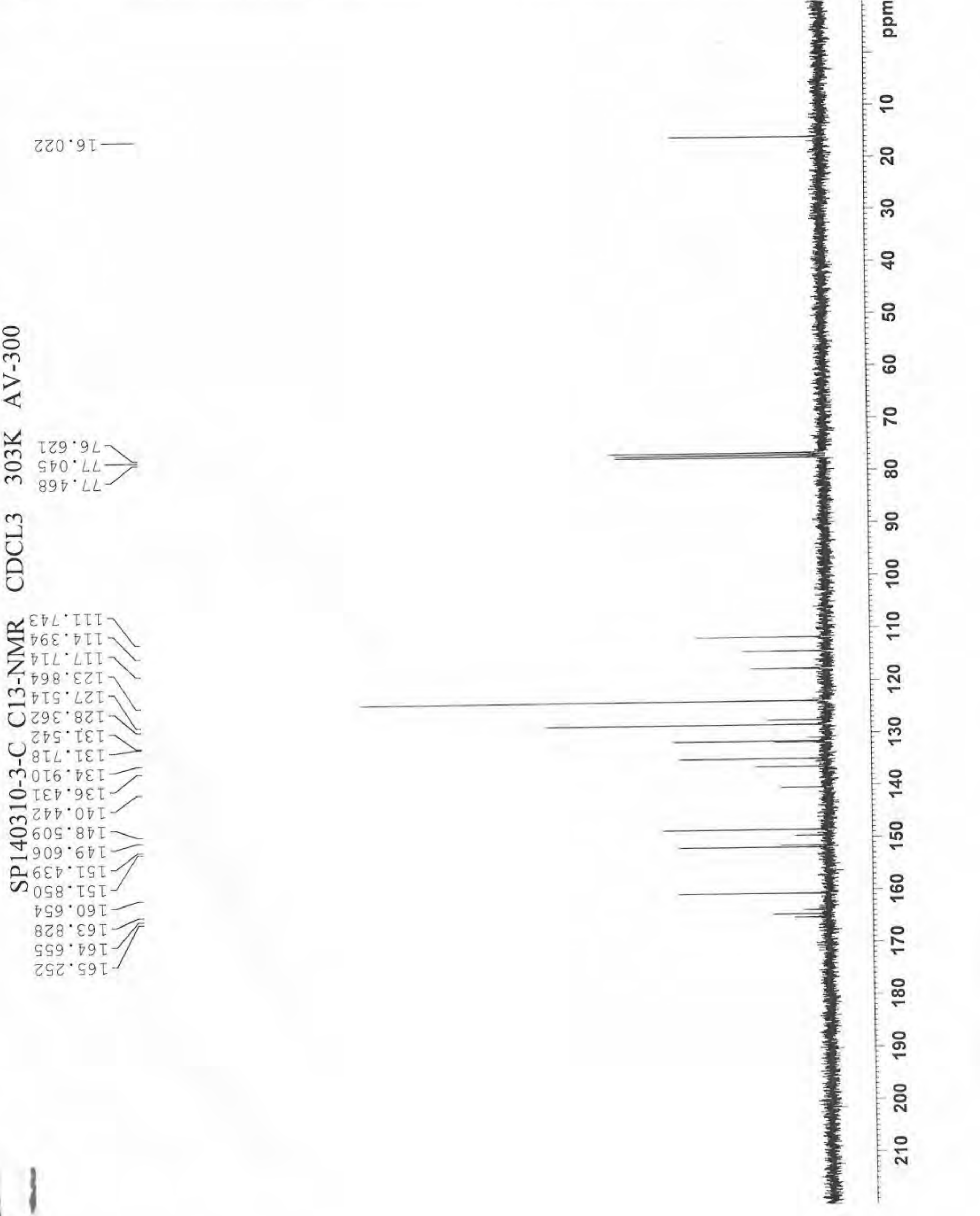

SP140310-3-C C13-NMR CDCL3 303K AV-300
165.252
164.655
163.828
160.654
151.850
151.439
149.606
148.509
140.442
136.431
134.910
131.718
131.542
128.362
127.514
123.864
117.714
114.394
111.743
77.468
77.045
76.621
16.022
ppm
10
20
30
40
50
60
70
80
90
100
110
120
130
140
150
160
170
180
190
200
210

A8

SP121109-1 1H-NMR CDCl3 303K AV-300

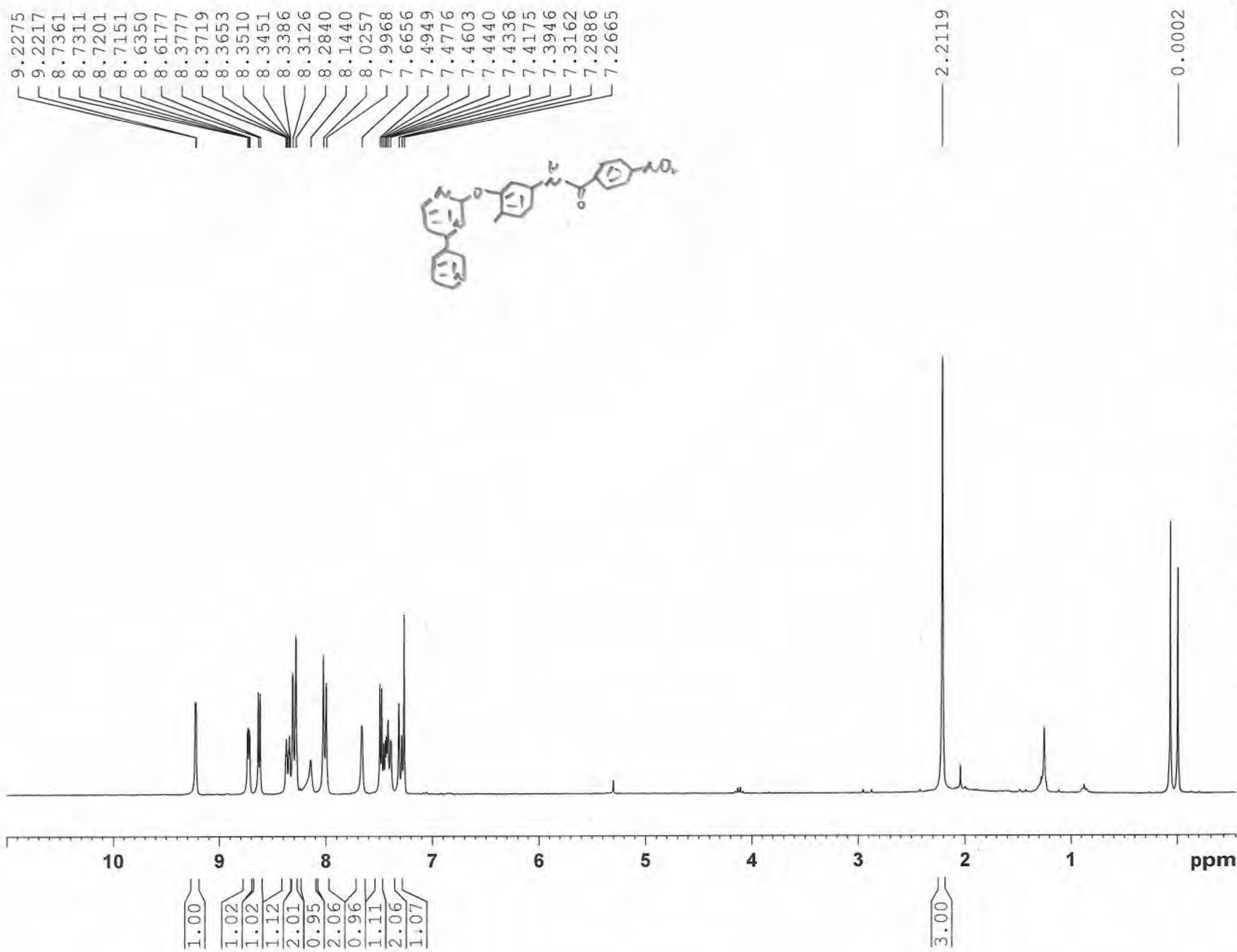

**Sample Name**		**Position**	p2E4	**Instrument Name**	Instrument 1
**User Name**	QTOF-PC\QTOF	**Inj Vol**	0.1	**InjPosition**	
**Sample Type**	Sample	**IRM Calibration Status**	Success	**Data Filename**	A8-p.d
**ACQ Method**	20110418-MSonly-p.m	**Comment**		**Acquired Time**	3/12/2018 3:07:26 PM

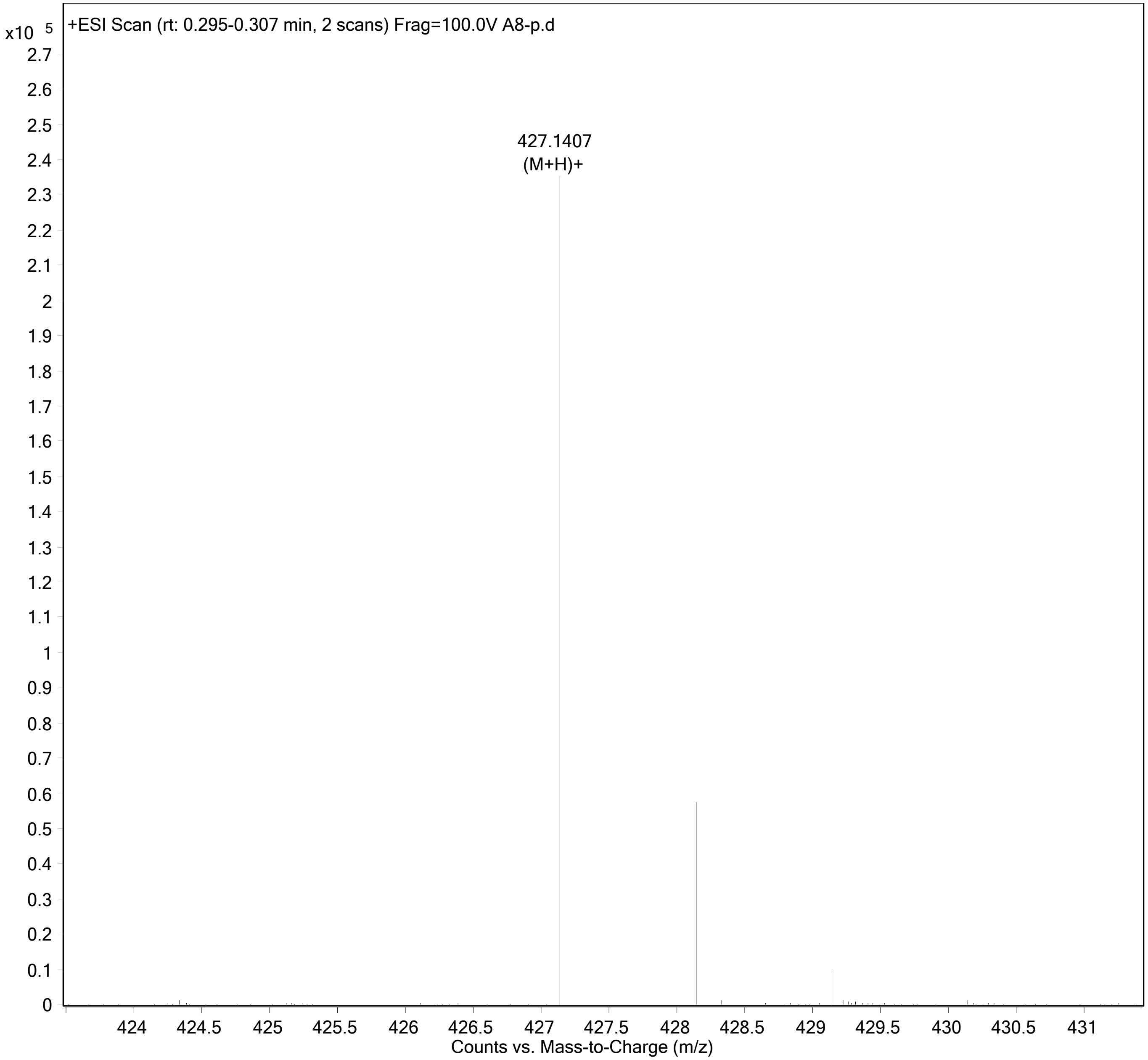

A9

SP121121-1 CDCl3 303K C13-NMR AV300

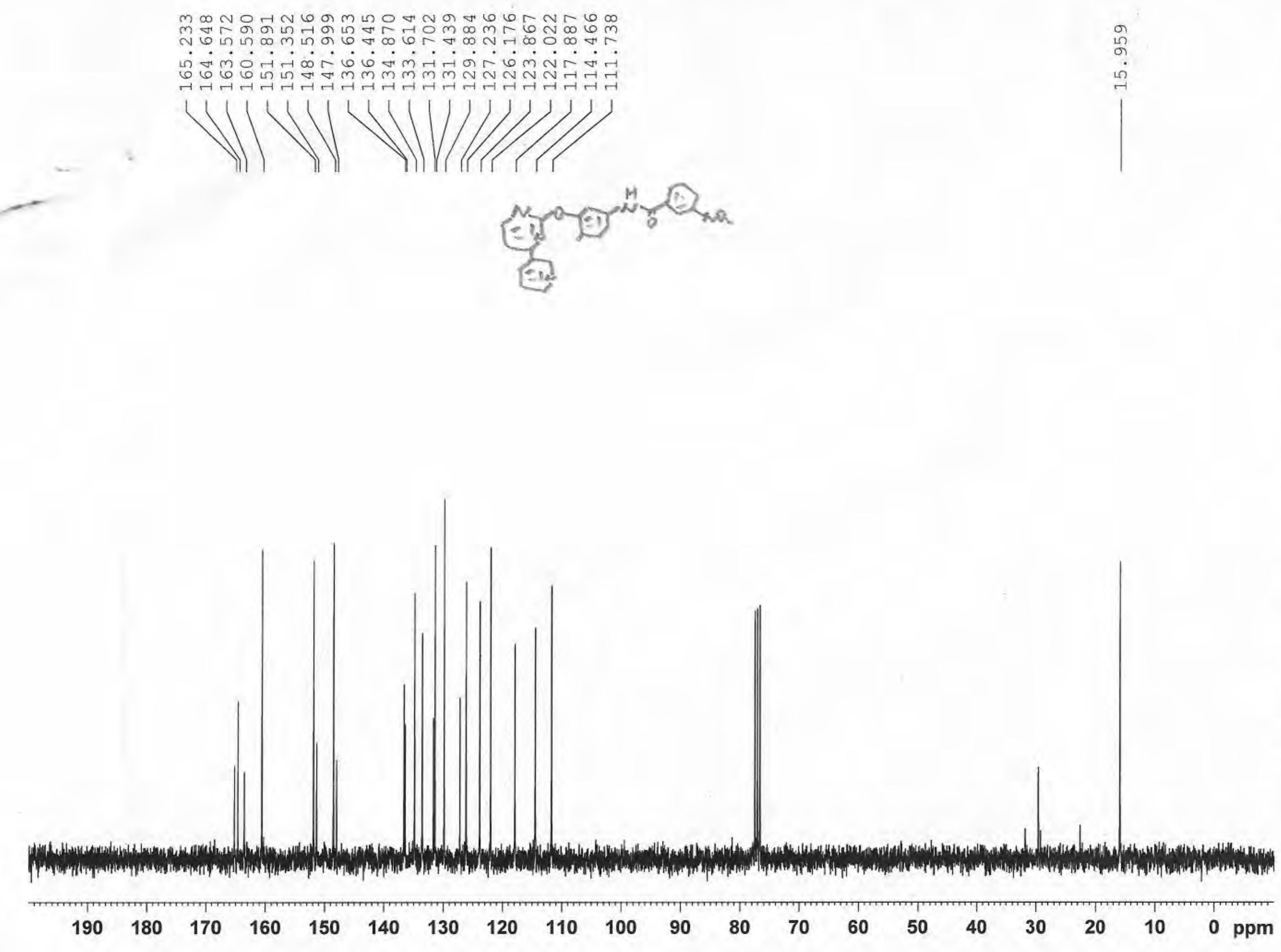

A 9

SP121109-2 1H-NMR CDCl3 303K AV-300

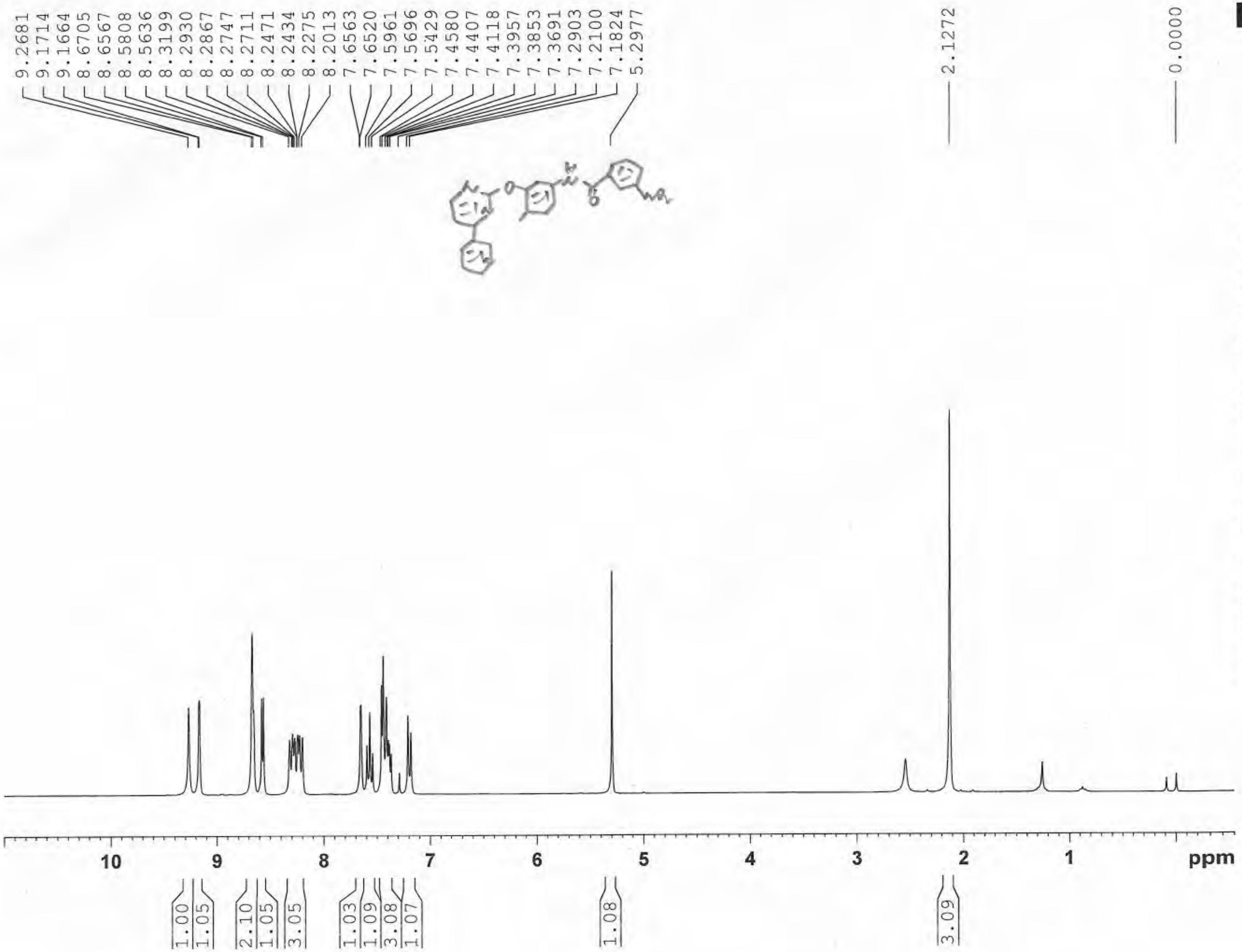

**Sample Name**		**Position**	p2E5	**Instrument Name**	Instrument 1
**User Name**	QTOF-PC\QTOF	**Inj Vol**	0.1	**InjPosition**	
**Sample Type**	Sample	**IRM Calibration Status**	Success	**Data Filename**	A9-p.d
**ACQ Method**	20110418-MSonly-p.m	**Comment**		**Acquired Time**	3/12/2018 3:09:32 PM

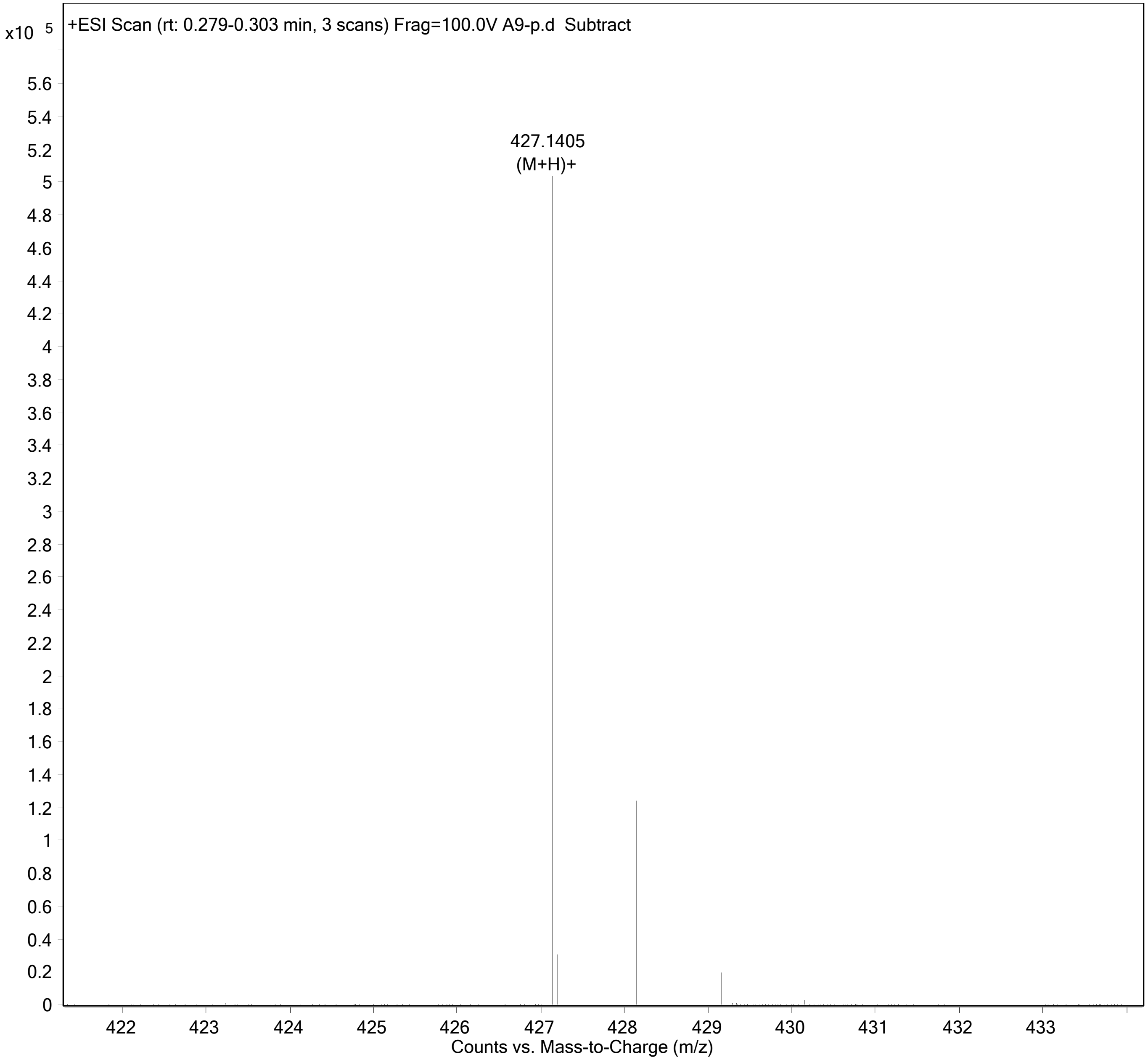

A 10

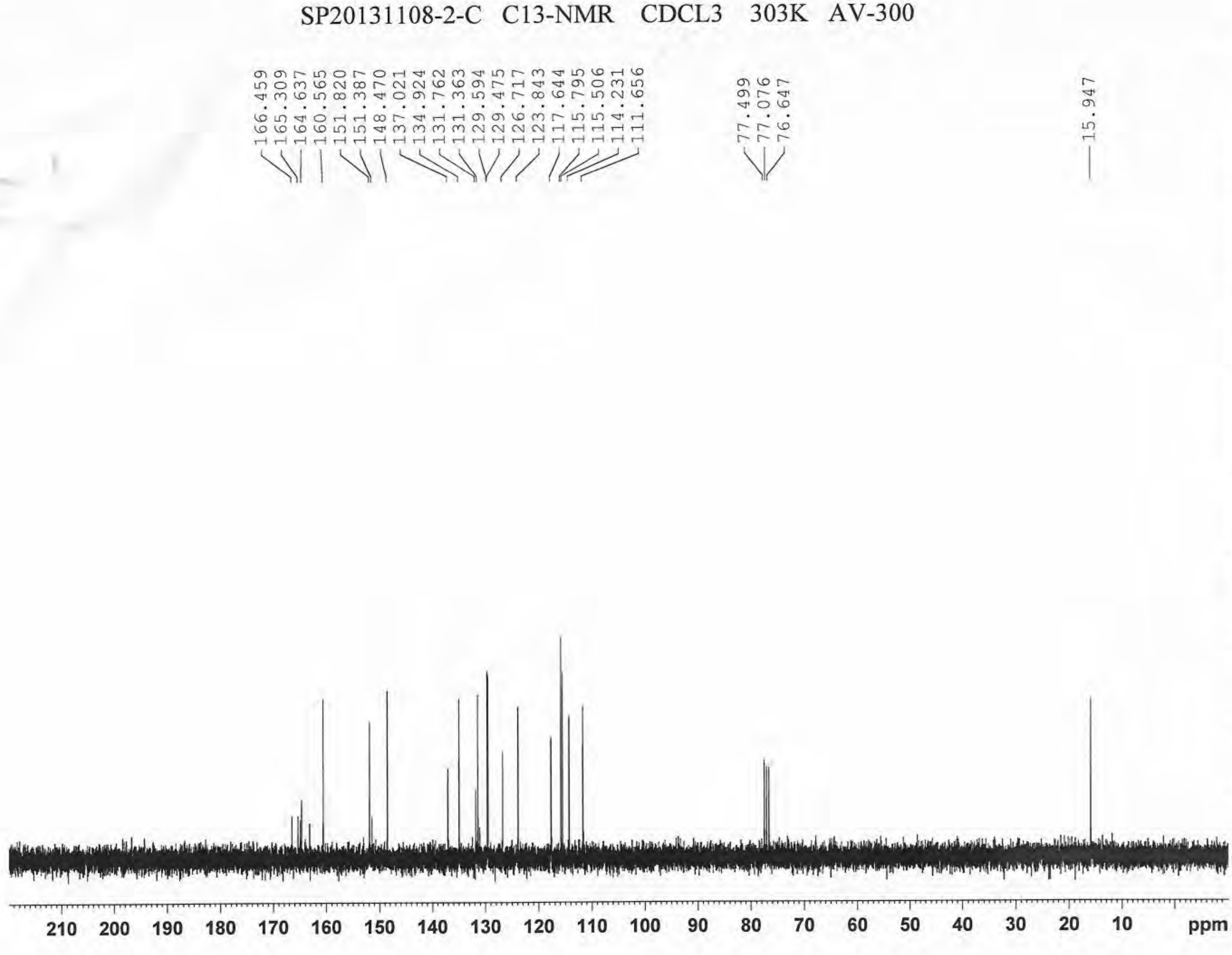
SP20131108-2-C C13-NMR CDCL3 303K AV-300
166.459
165.309
164.637
160.565
151.820
151.387
148.470
137.021
134.924
131.762
131.363
129.594
129.475
126.717
123.843
117.644
115.795
115.506
114.231
111.656
77.499
77.076
76.647
15.947
210 200 190 180 170 160 150 140 130 120 110 100 90 80 70 60 50 40 30 20 10 ppm

A10

SP20131108-2 CDCL3 1HNMR AV300

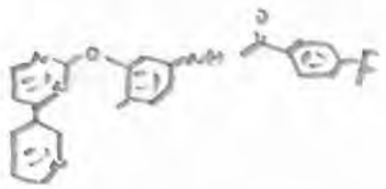

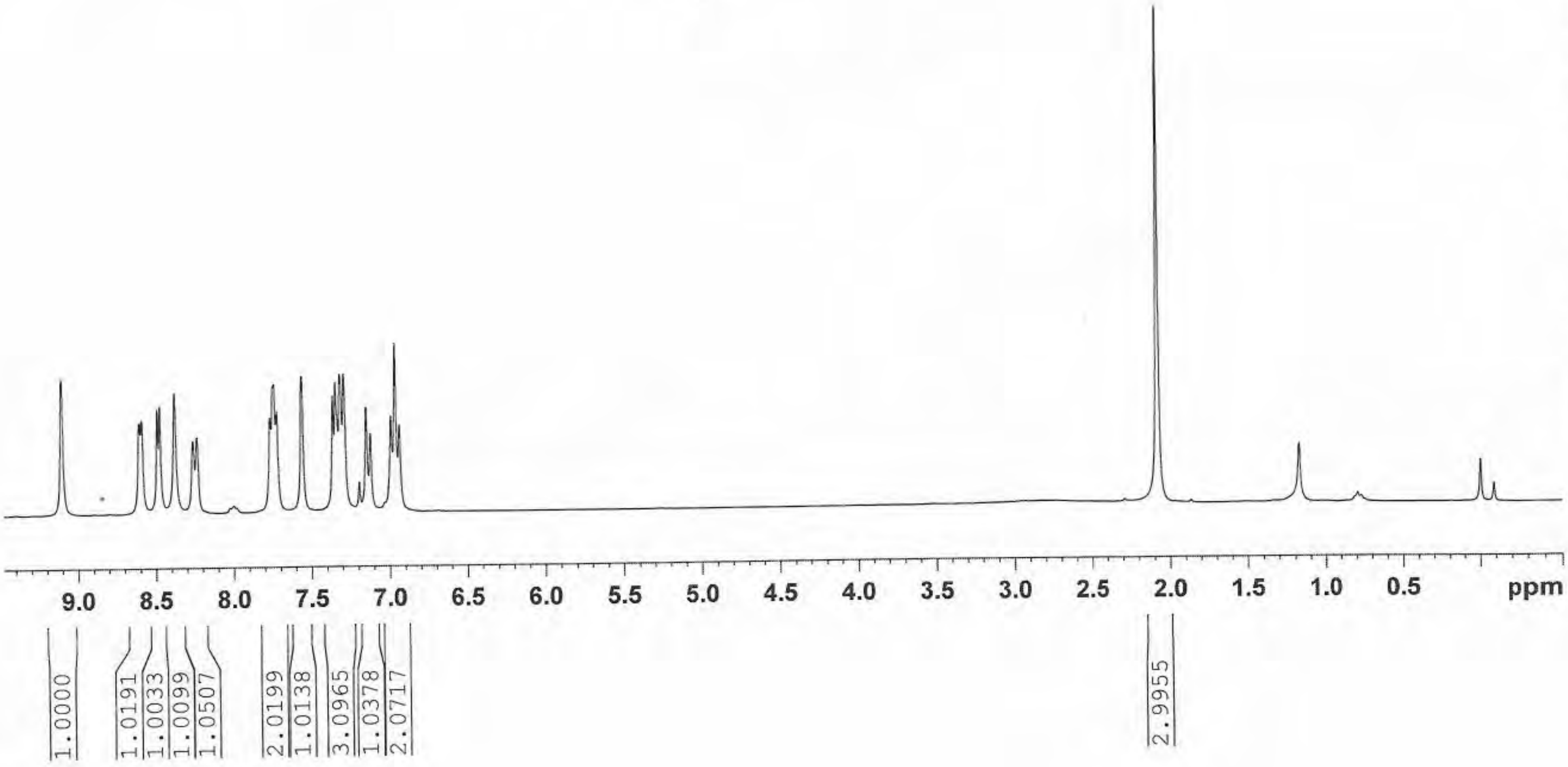

**Sample Name**		**Position**	p2E6	**Instrument Name**	Instrument 1
**User Name**	QTOF-PC\QTOF	**Inj Vol**	0.1	**InjPosition**	
**Sample Type**	Sample	**IRM Calibration Status**	Success	**Data Filename**	A10-p.d
**ACQ Method**	20110418-MSonly-p.m	**Comment**		**Acquired Time**	3/12/2018 3:16:43 PM

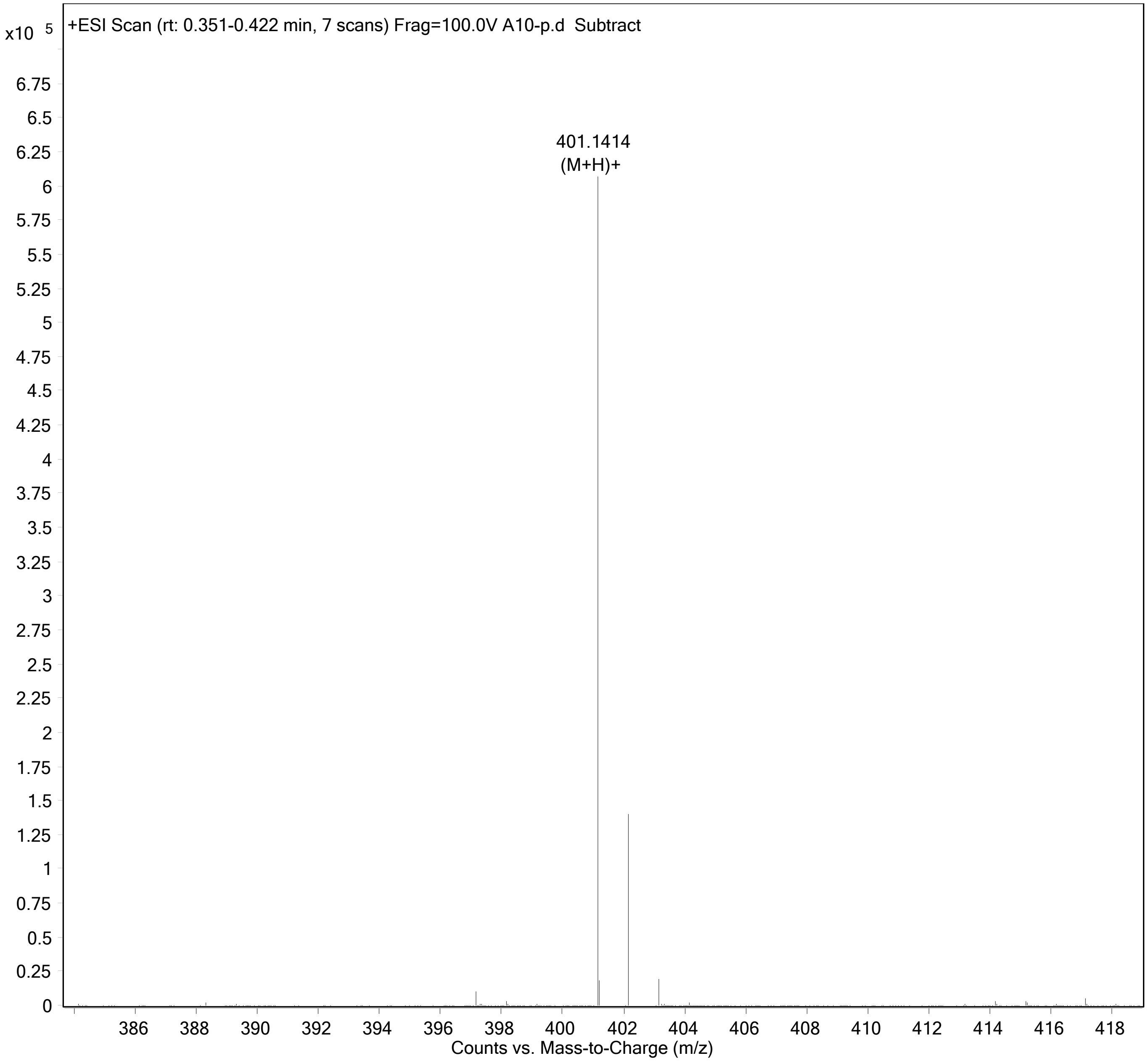

A11

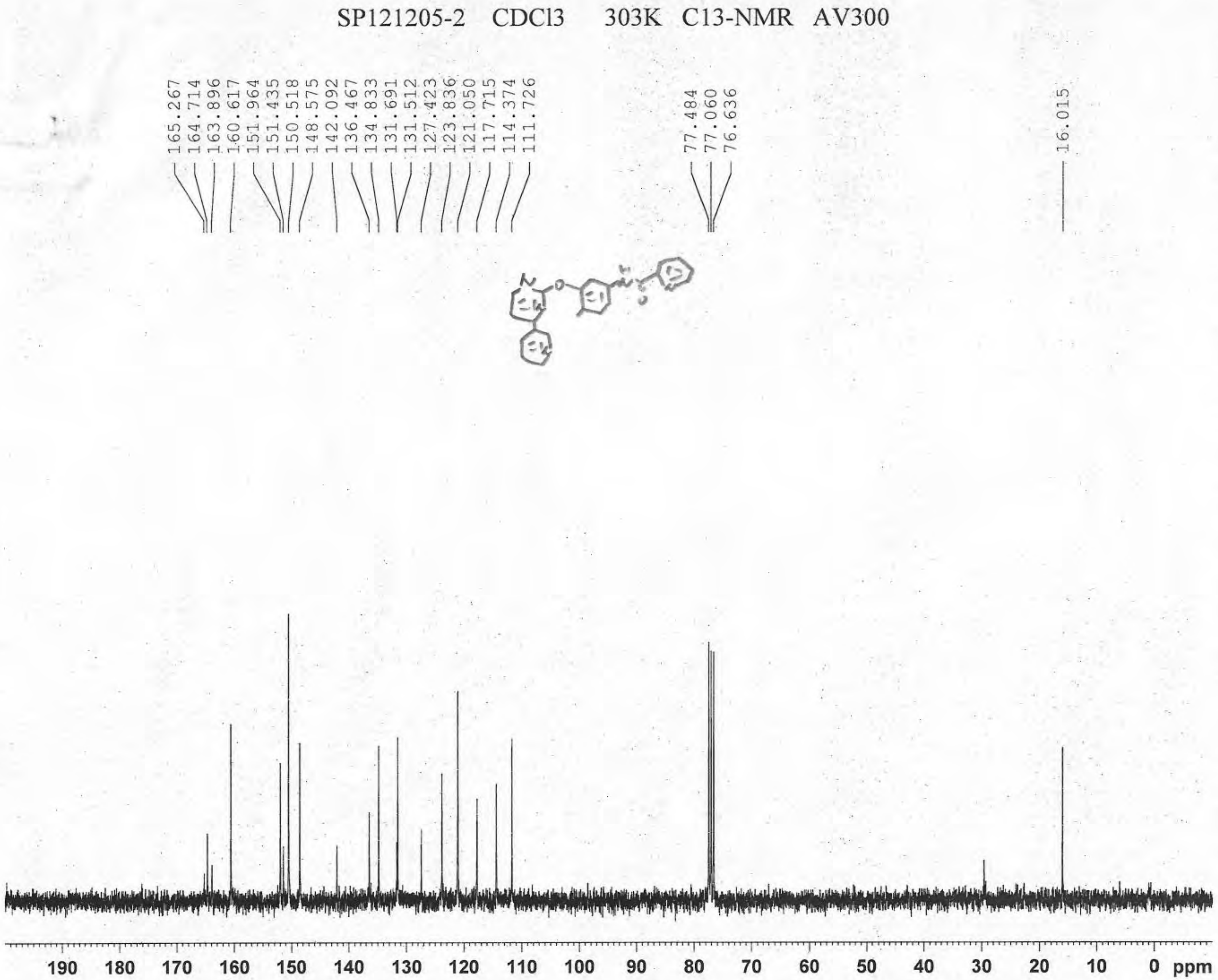

SP121205-2 CDCl3 303K C13-NMR AV300
165.267
164.714
163.896
160.617
151.964
151.435
150.518
148.575
142.092
136.467
134.833
131.691
131.512
127.423
123.836
121.050
117.715
114.374
111.726
77.484
77.060
76.636
16.015
190 180 170 160 150 140 130 120 110 100 90 80 70 60 50 40 30 20 10 0 ppm

A11

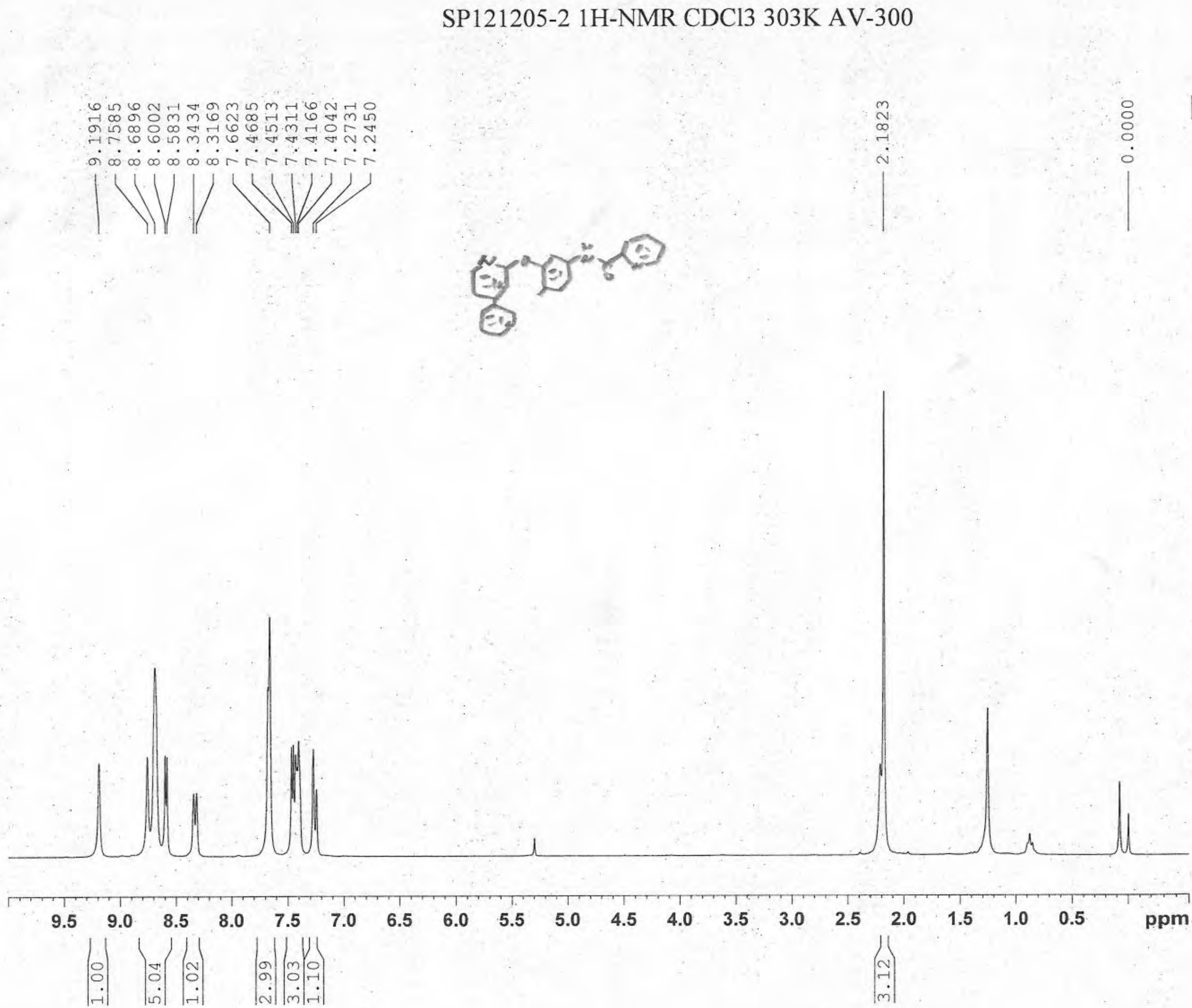
SP121205-2 1H-NMR CDCl3 303K AV-300
9.1916
8.7585
8.6896
8.6002
8.5831
8.3434
8.3169
7.6623
7.4685
7.4513
7.4311
7.4166
7.4042
7.2731
7.2450
2.1823
0.0000
9.5
9.0
8.5
8.0
7.5
7.0
6.5
6.0
5.5
5.0
4.5
4.0
3.5
3.0
2.5
2.0
1.5
1.0
0.5
ppm
1.00
5.04
1.02
2.99
3.03
1.10
3.12

**Sample Name**		**Position**	p2E7	**Instrument Name**	Instrument 1
**User Name**	QTOF-PC\QTOF	**Inj Vol**	0.1	**InjPosition**	
**Sample Type**	Sample	**IRM Calibration Status**	Success	**Data Filename**	A11-p.d
**ACQ Method**	20110418-MSonly-p.m	**Comment**		**Acquired Time**	3/12/2018 3:19:42 PM

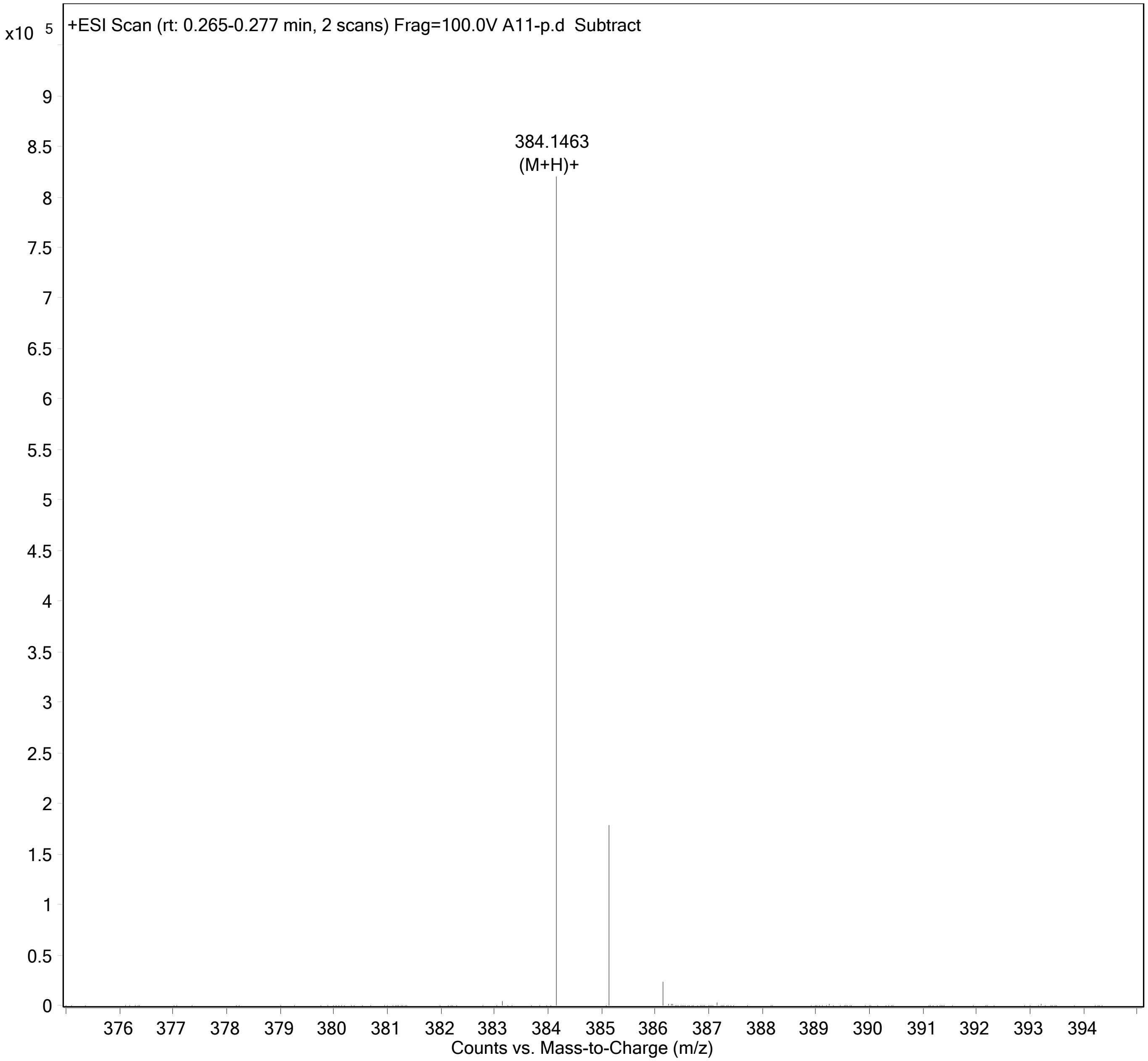

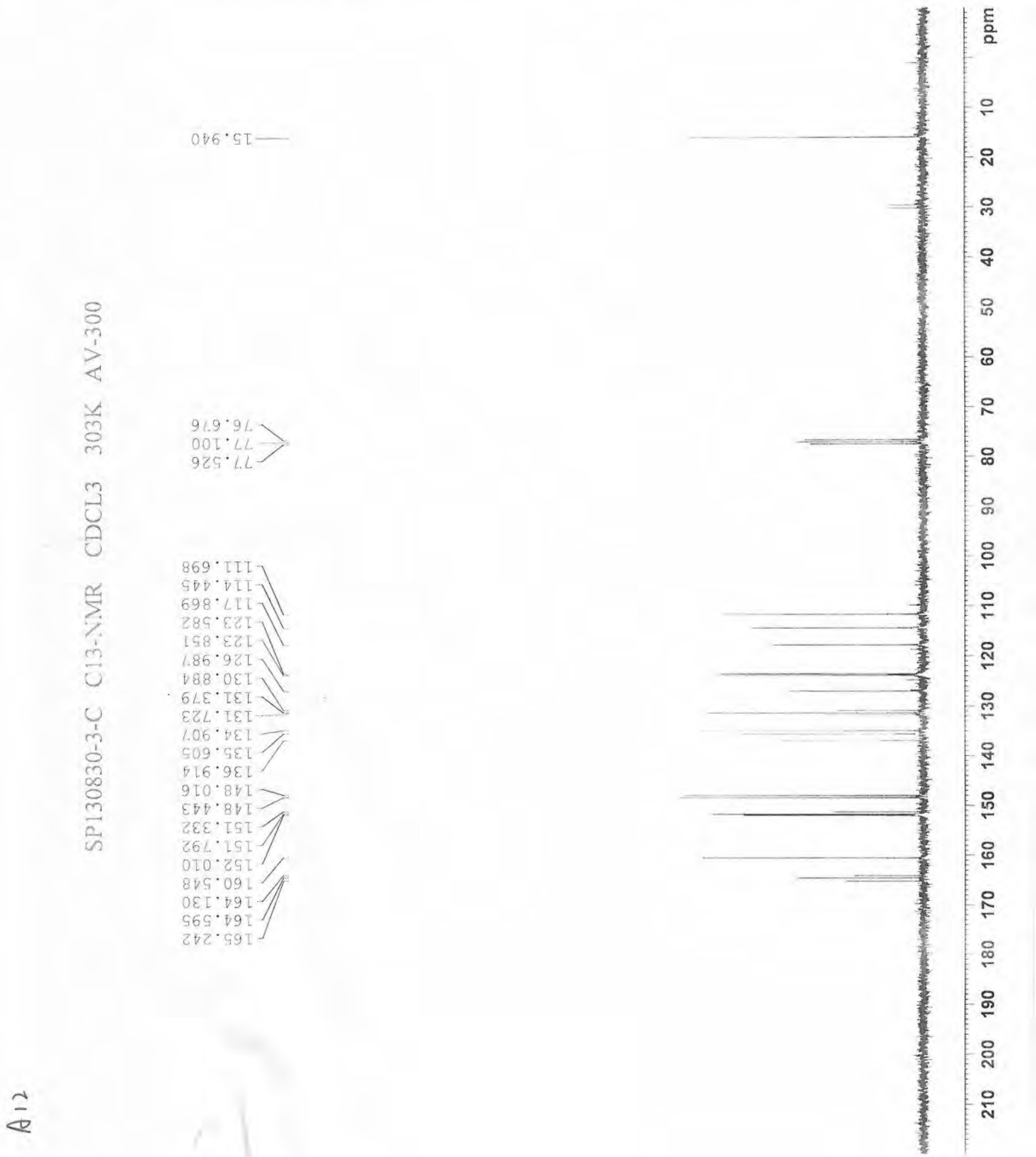
A12
SP130830-3-C C13-NMR CDCL3 303K AV-300
15.940
76.676
77.100
77.526
111.698
114.445
117.869
123.582
123.851
126.987
130.884
131.379
131.723
134.907
135.605
136.914
148.016
148.443
151.332
151.792
152.010
160.548
164.130
164.595
165.242
ppm
10
20
30
40
50
60
70
80
90
100
110
120
130
140
150
160
170
180
190
200
210

A12

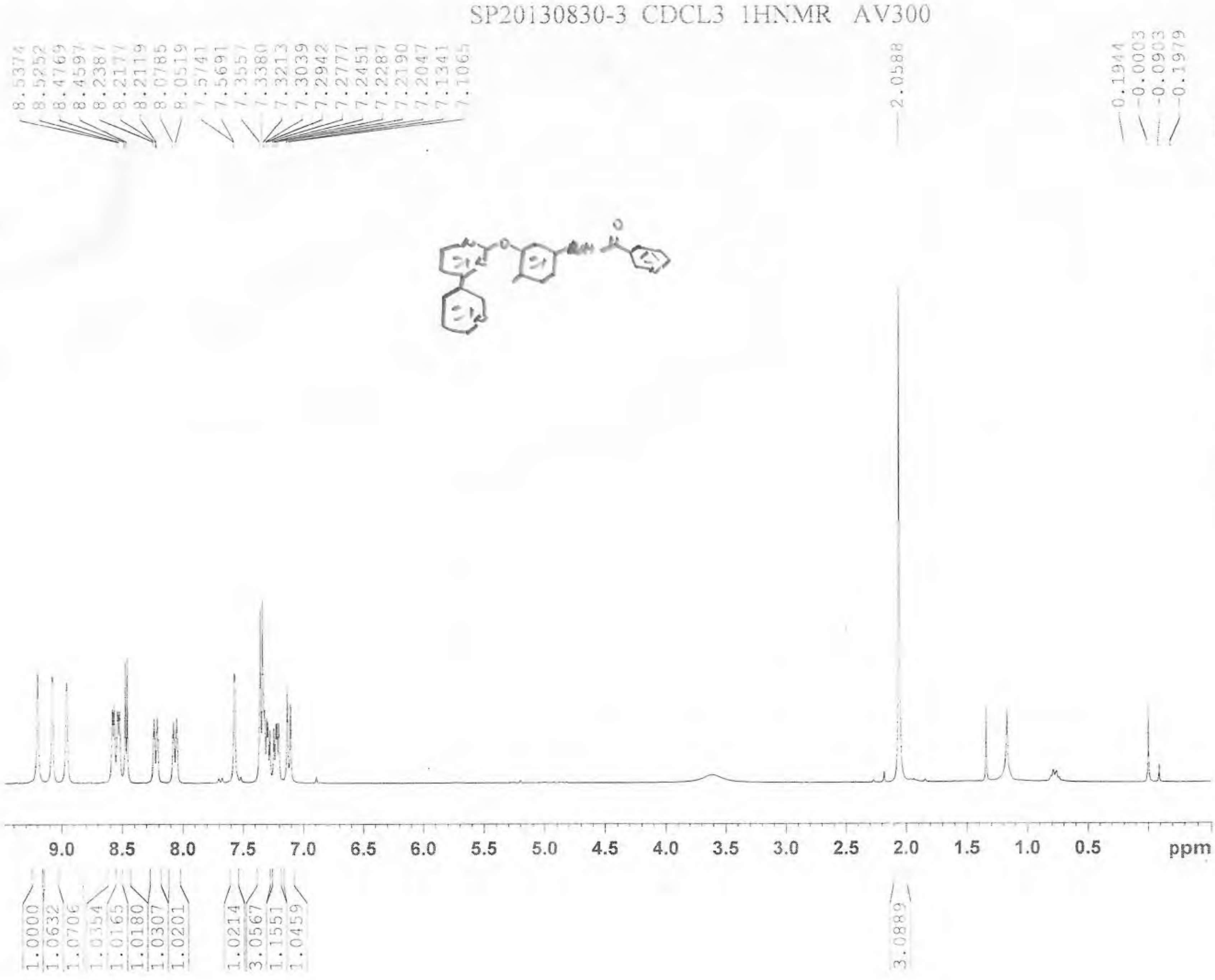

**Sample Name**		**Position**	p2E8	**Instrument Name**	Instrument 1
**User Name**	QTOF-PC\QTOF	**Inj Vol**	0.1	**InjPosition**	
**Sample Type**	Sample	**IRM Calibration Status**	Success	**Data Filename**	A12-p.d
**ACQ Method**	20110418-MSonly-p.m	**Comment**		**Acquired Time**	3/12/2018 3:24:41 PM

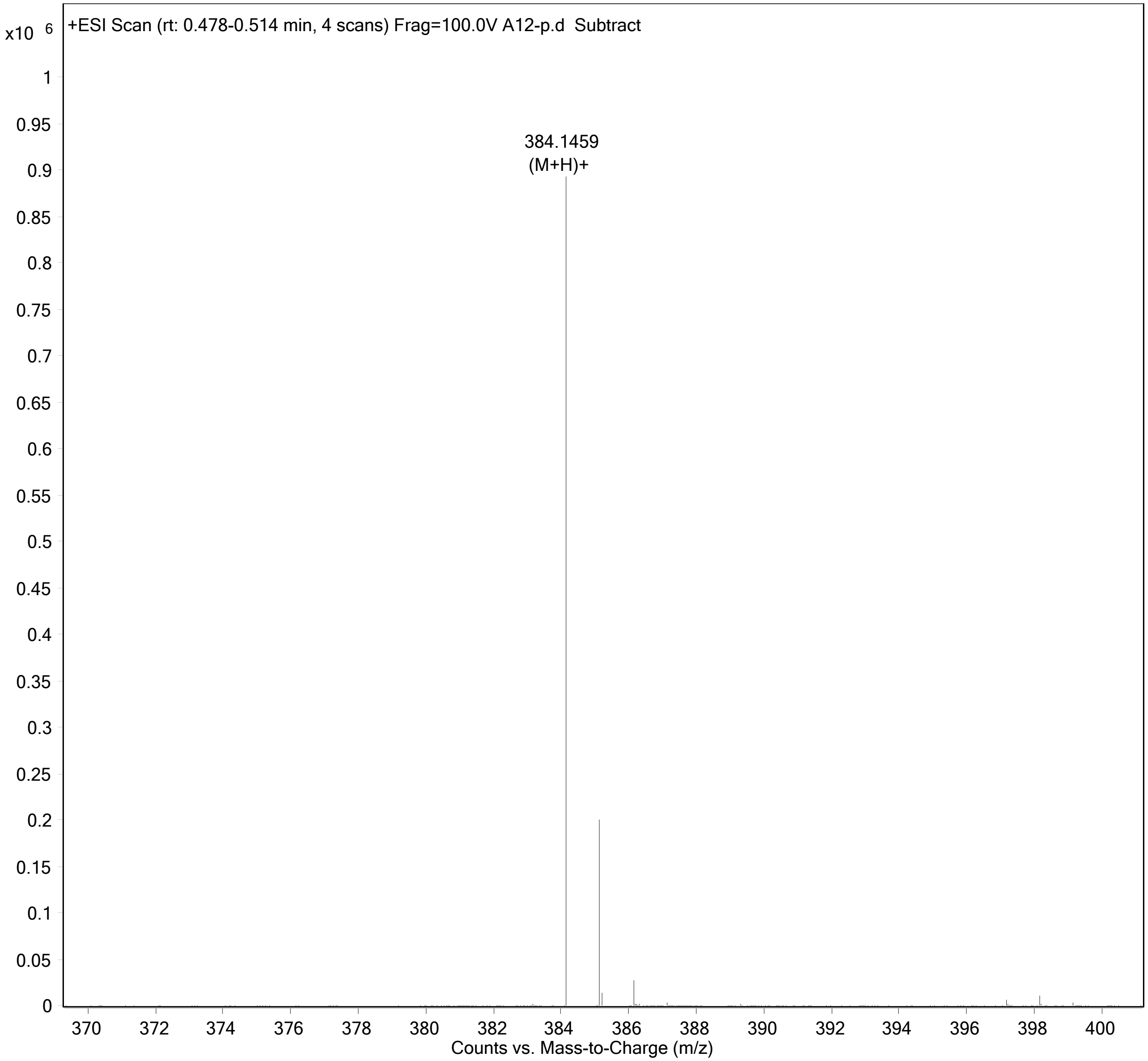

B1

SP121205-4 CDCl3 303K C13-NMR AV300

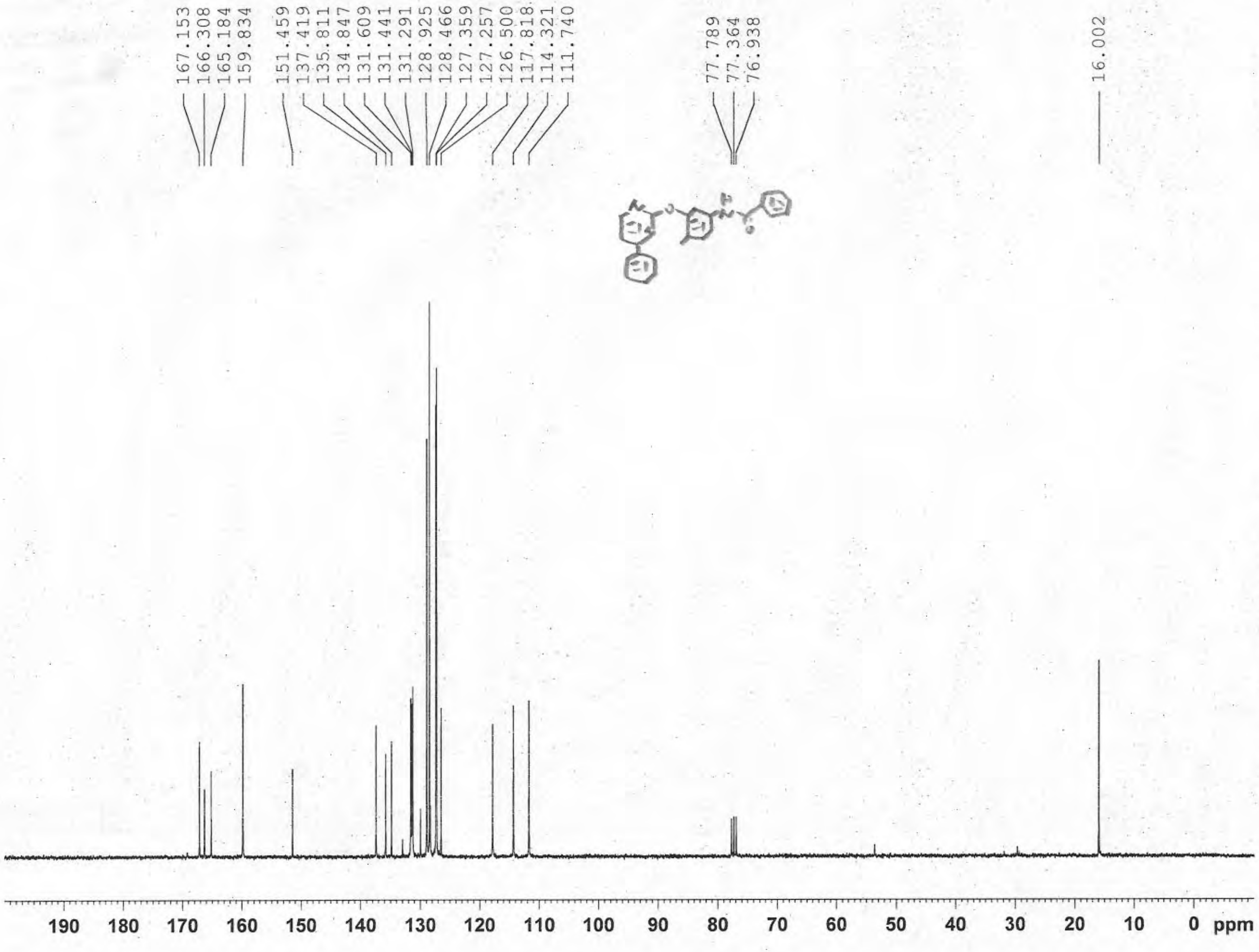

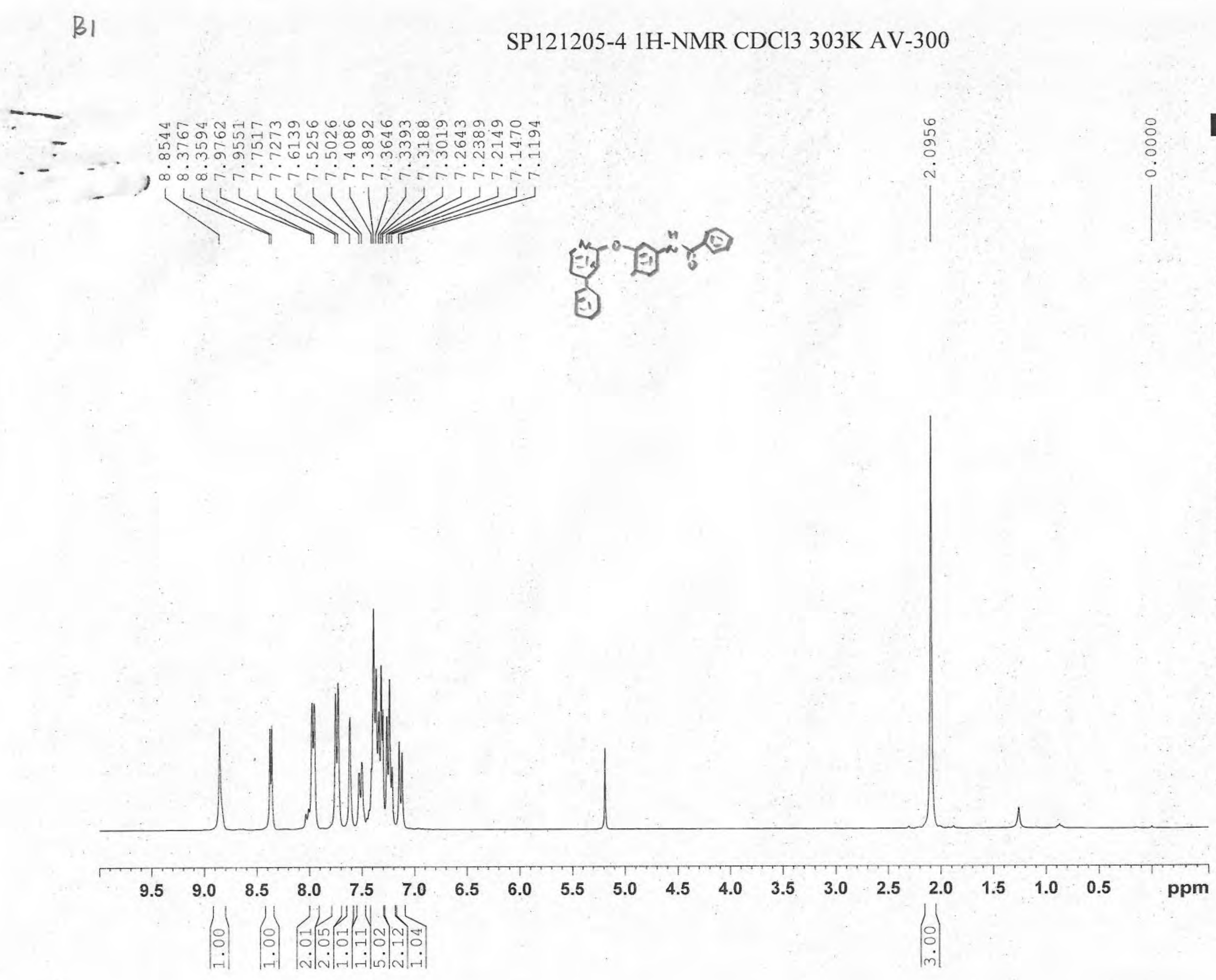
B1
SP121205-4 1H-NMR CDCl3 303K AV-300
8.8544
8.3767
8.3594
7.9762
7.9551
7.7517
7.7273
7.6139
7.5256
7.5026
7.4086
7.3892
7.3646
7.3393
7.3188
7.3019
7.2643
7.2389
7.2149
7.1470
7.1194
2.0956
0.0000
9.5 9.0 8.5 8.0 7.5 7.0 6.5 6.0 5.5 5.0 4.5 4.0 3.5 3.0 2.5 2.0 1.5 1.0 0.5 ppm
1.00
1.00
2.01
2.05
1.01
1.11
5.02
2.12
1.04
3.00

**Sample Name**		**Position**	p2E9	**Instrument Name**	Instrument 1
**User Name**	QTOF-PC\QTOF	**Inj Vol**	0.1	**InjPosition**	
**Sample Type**	Sample	**IRM Calibration Status**	Success	**Data Filename**	B1-p.d
**ACQ Method**	20110418-MSonly-p.m	**Comment**		**Acquired Time**	3/12/2018 3:27:08 PM

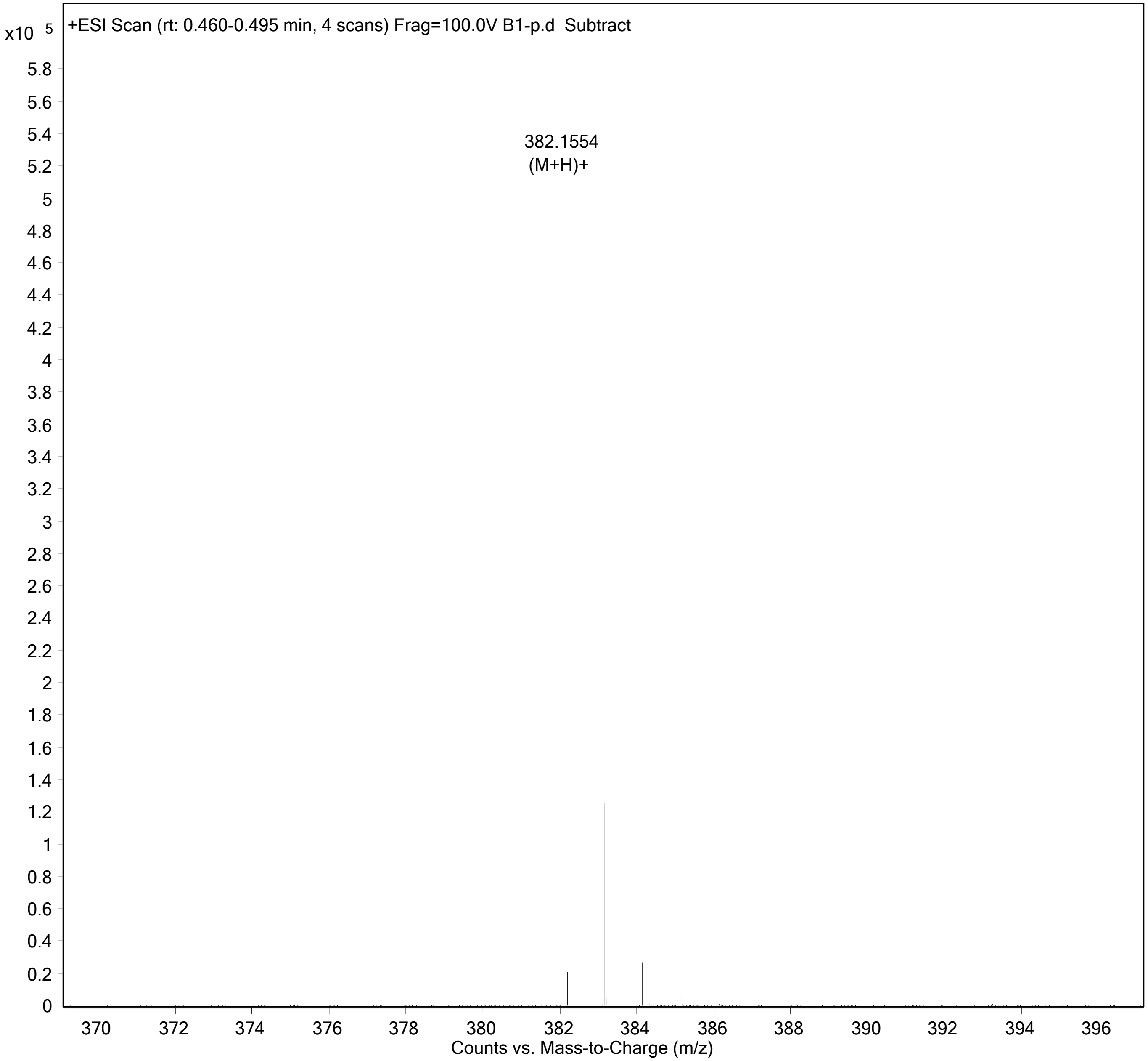

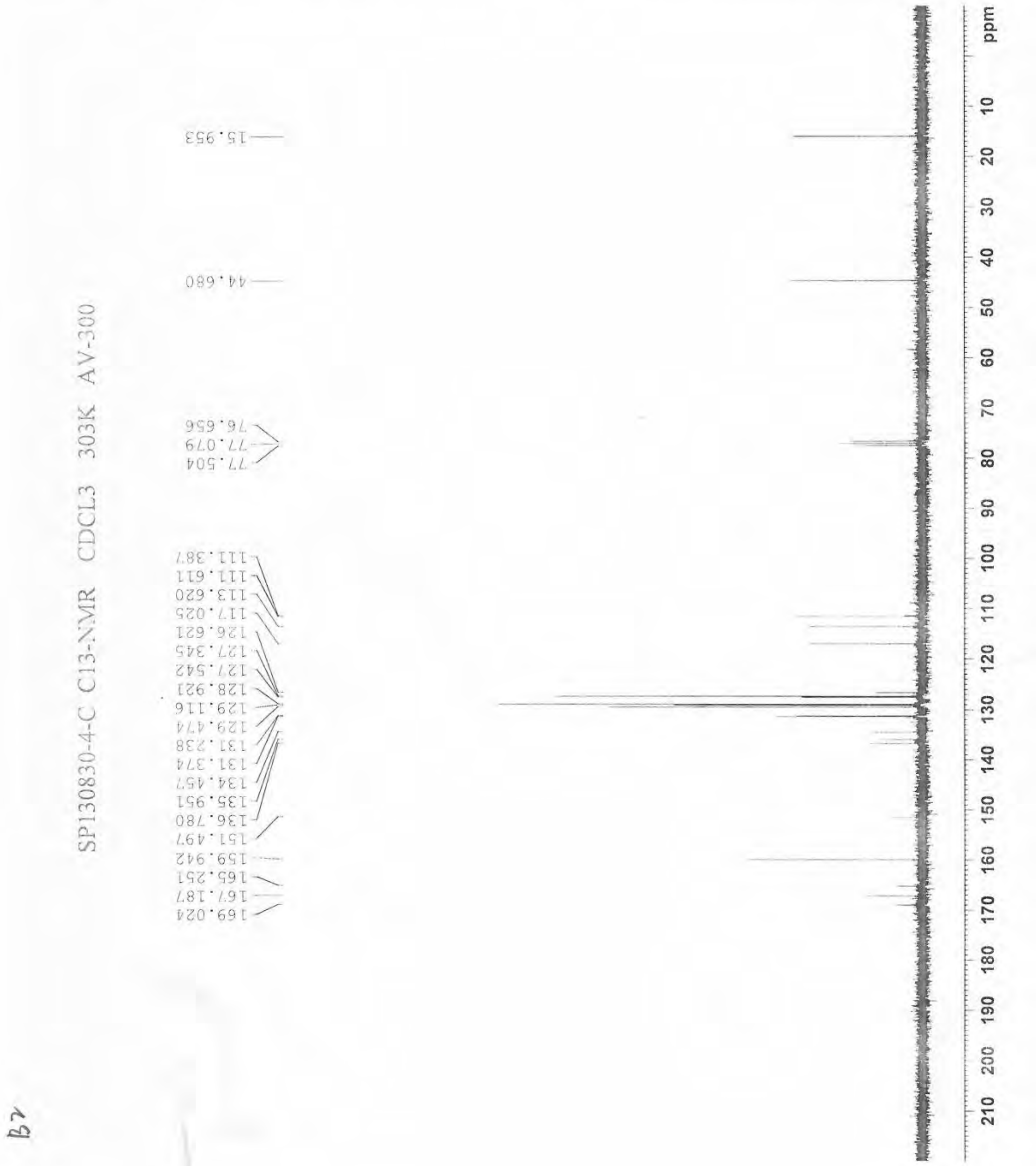
SP130830-4-C C13-NMR CDCL3 303K AV-300
15.953
44.680
76.656
77.079
77.504
111.387
111.611
113.620
117.025
126.621
127.345
127.542
128.921
129.116
129.474
131.238
131.374
134.457
135.951
136.780
151.497
159.942
165.251
167.187
169.024
ppm
10 20 30 40 50 60 70 80 90 100 110 120 130 140 150 160 170 180 190 200 210

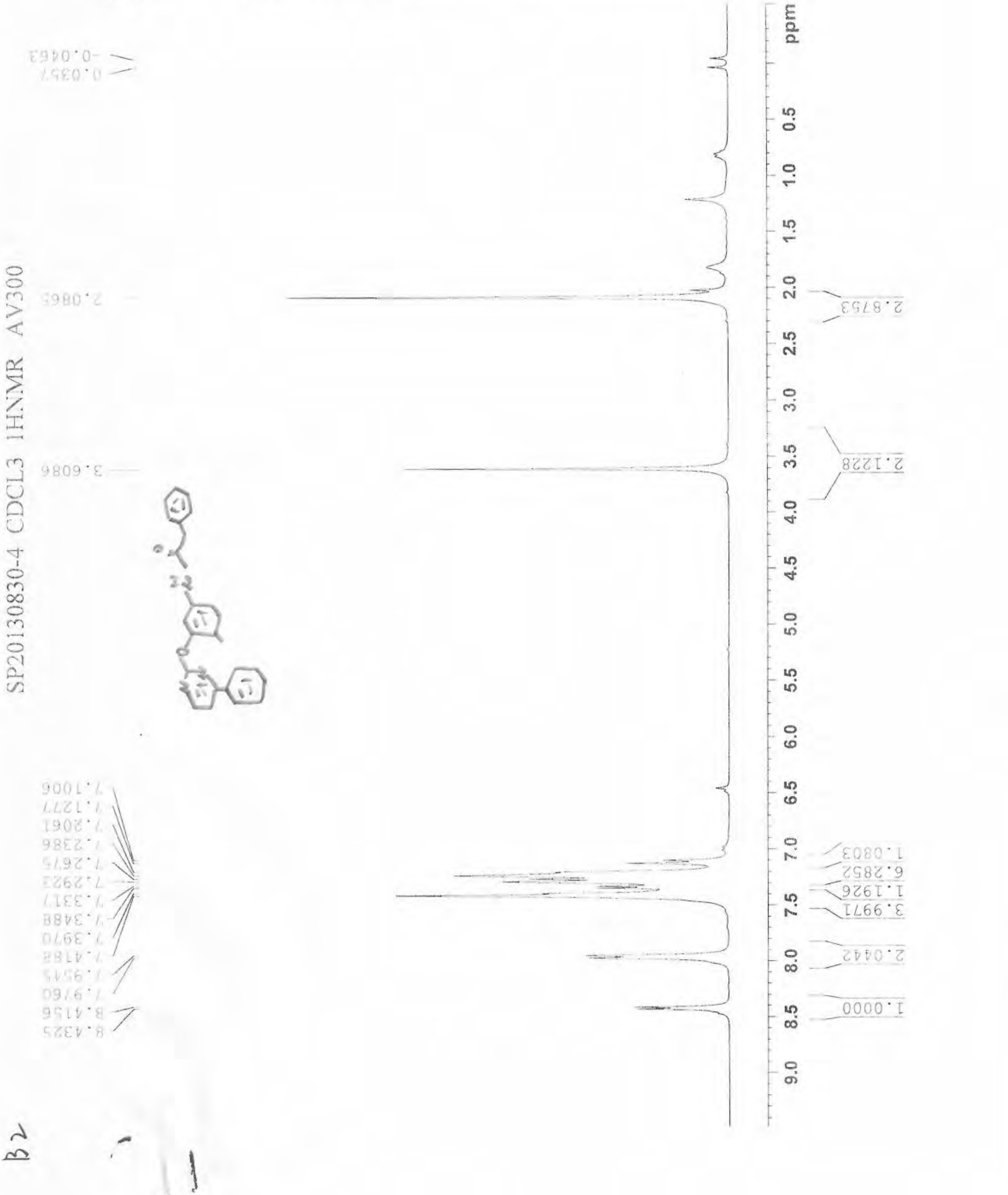
SP20130830-4 CDCL3 1HNMR AV300
8.4325
8.4156
7.9760
7.9545
7.4188
7.3970
7.3488
7.3317
7.2923
7.2675
7.2386
7.2061
7.1277
7.1006
3.6086
2.0865
0.0357
-0.0463
1.0000
2.0442
3.9971
1.1926
6.2852
1.0803
2.1228
2.8753
ppm

**Sample Name**		**Position**	p2F1	**Instrument Name**	Instrument 1
**User Name**	QTOF-PC\QTOF	**Inj Vol**	0.1	**InjPosition**	
**Sample Type**	Sample	**IRM Calibration Status**	Success	**Data Filename**	B2-p.d
**ACQ Method**	20110418-MSonly-p.m	**Comment**		**Acquired Time**	3/12/2018 3:29:48 PM

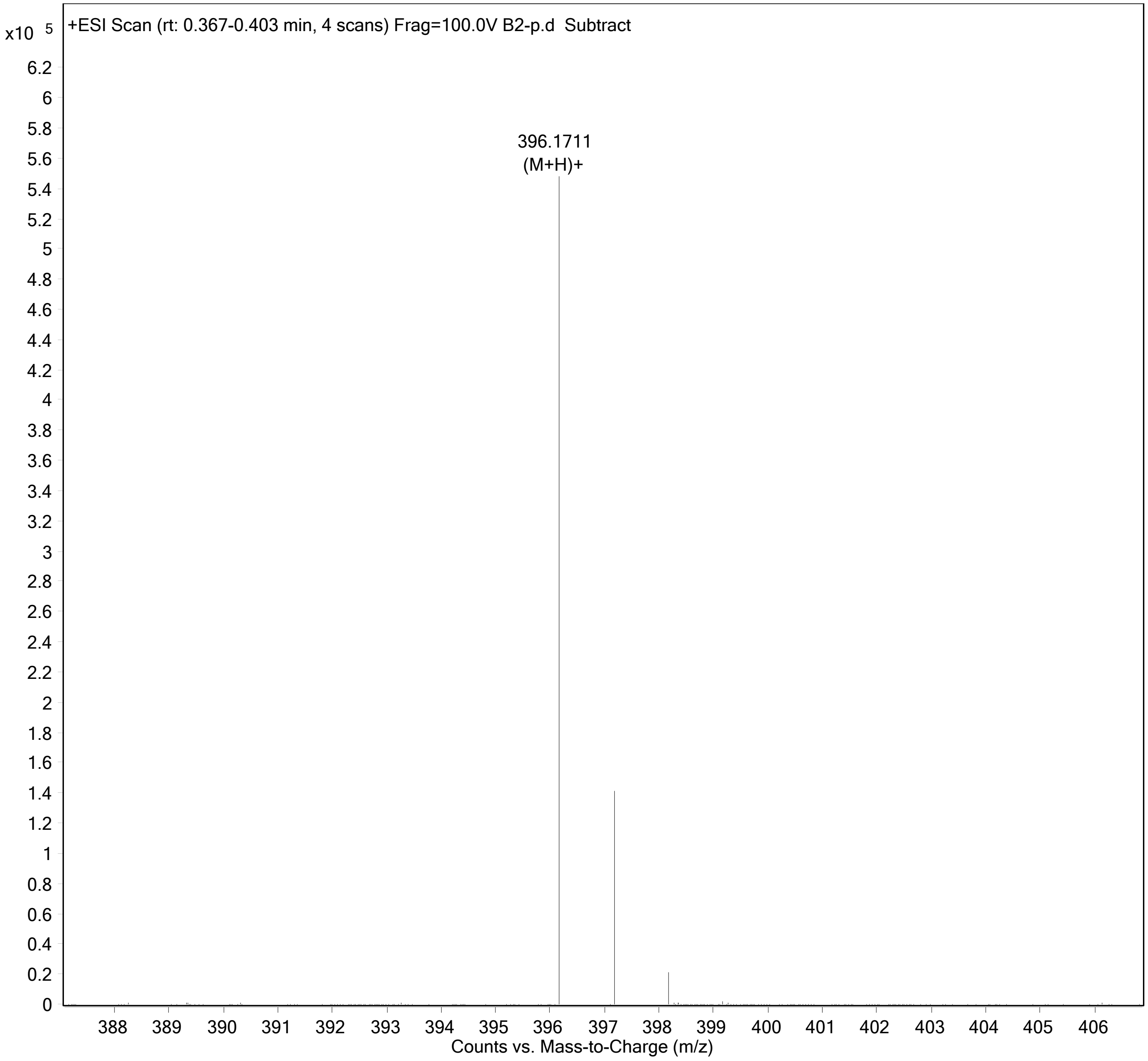

B3

SP121128-3 CDCl3 303K C13-NMR AV300

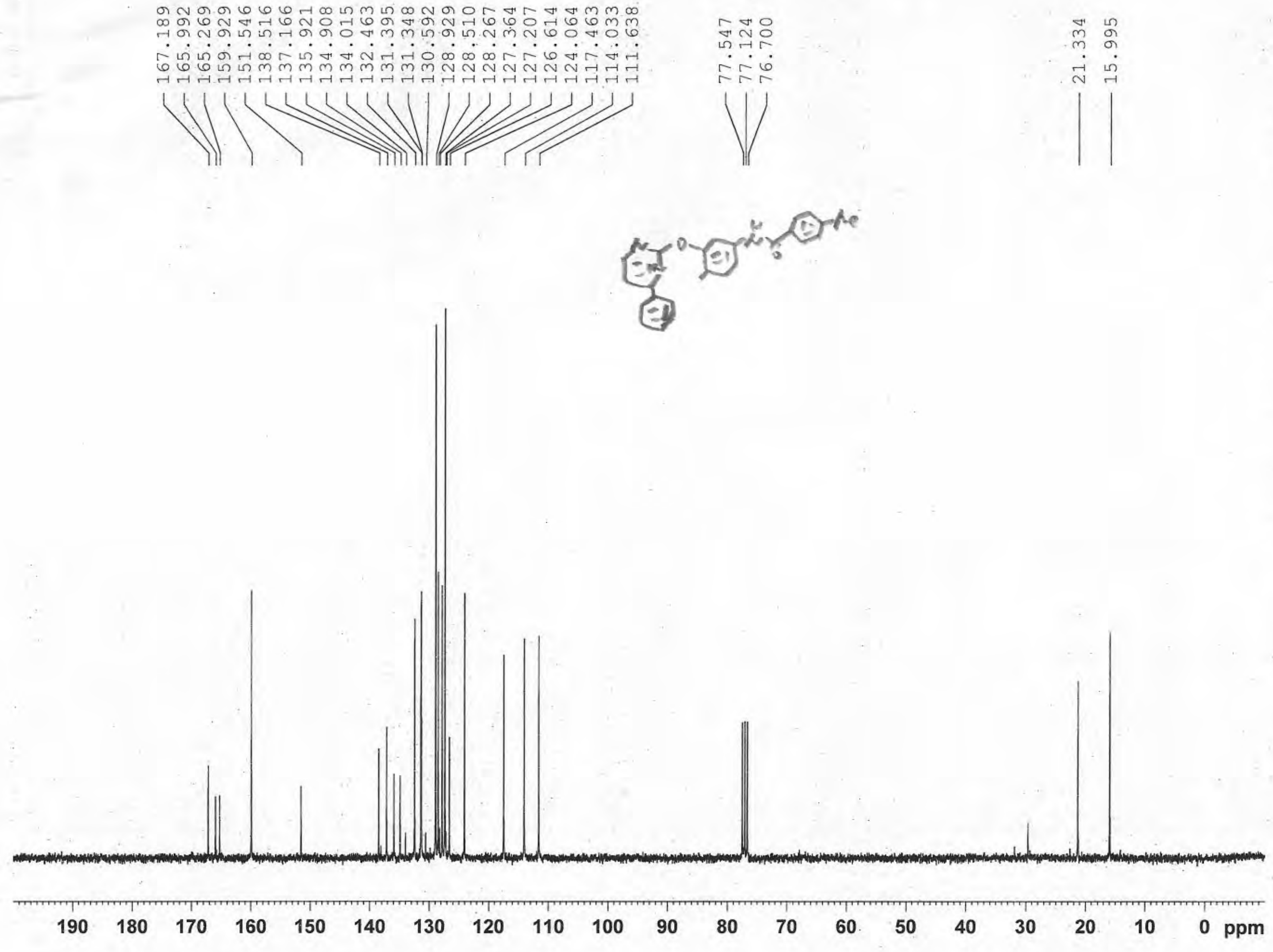

B 3

SP121128-3 1HNMR CDCl3 303K AV-300

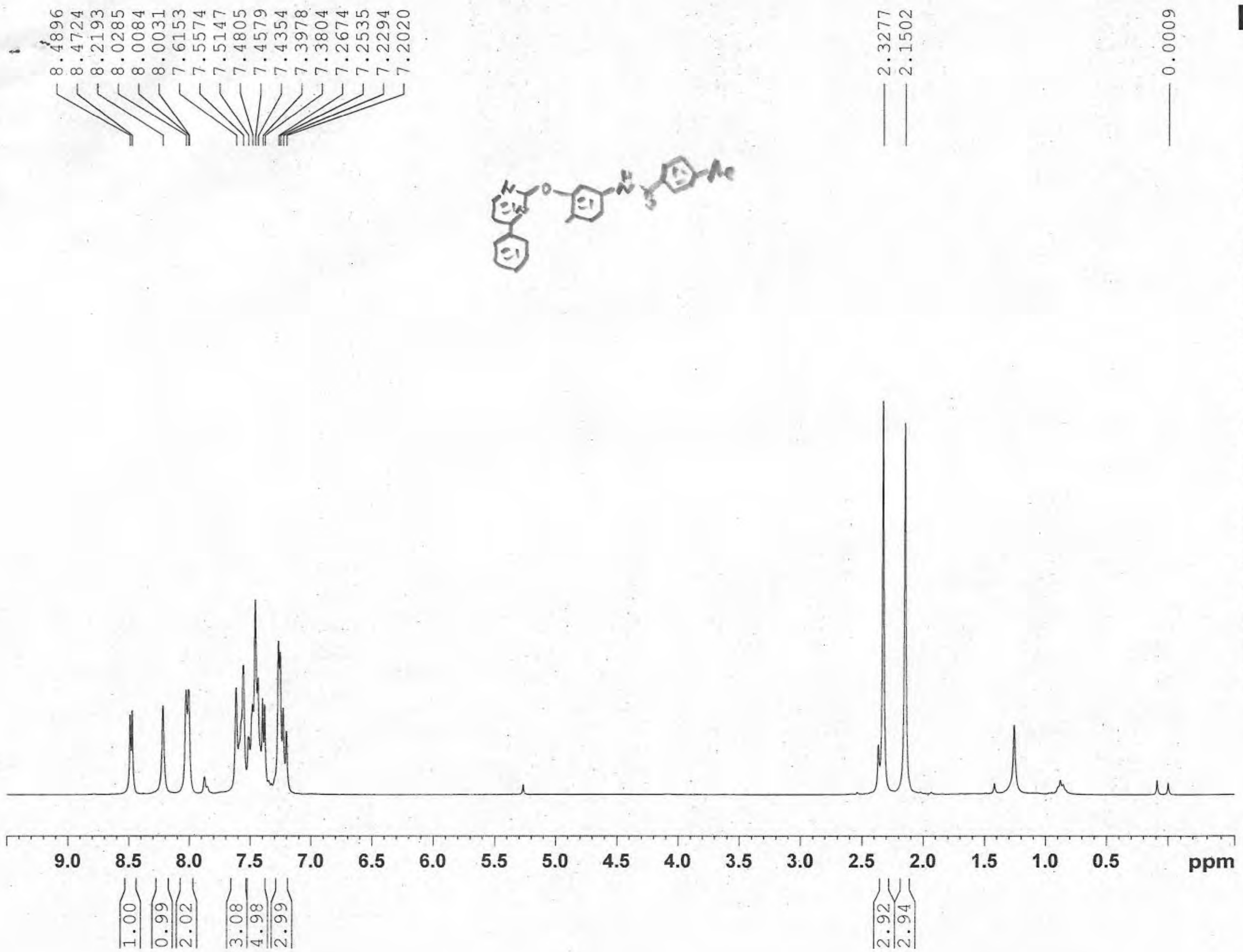

**Sample Name**		**Position**	p2F2	**Instrument Name**	Instrument 1
**User Name**	QTOF-PC\QTOF	**Inj Vol**	0.1	**InjPosition**	
**Sample Type**	Sample	**IRM Calibration Status**	Success	**Data Filename**	B3-p.d
**ACQ Method**	20110418-MSonly-p.m	**Comment**		**Acquired Time**	3/12/2018 3:32:10 PM

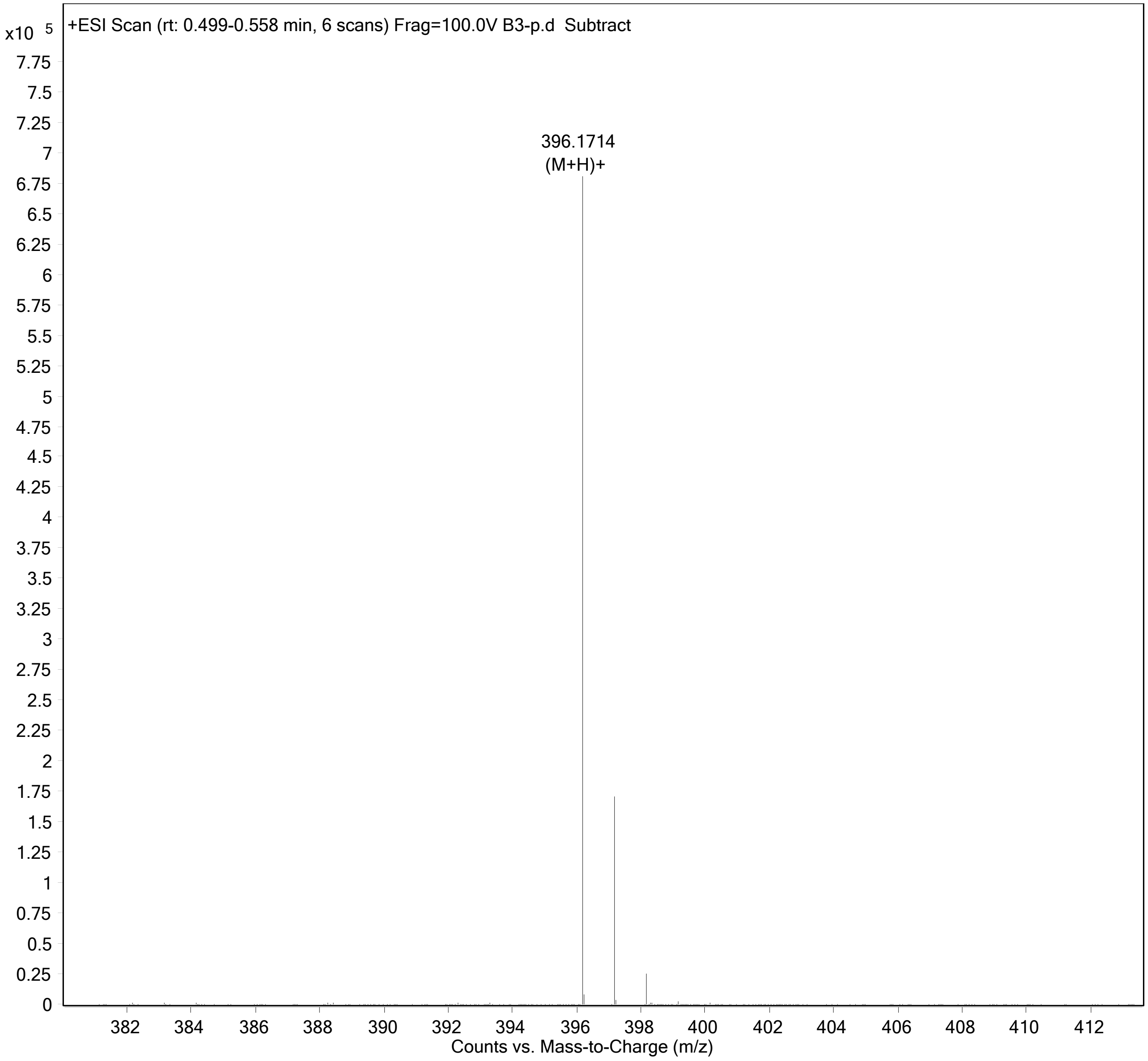

B4

SP121202-2 CDCl3 303K C13-NMR AV300

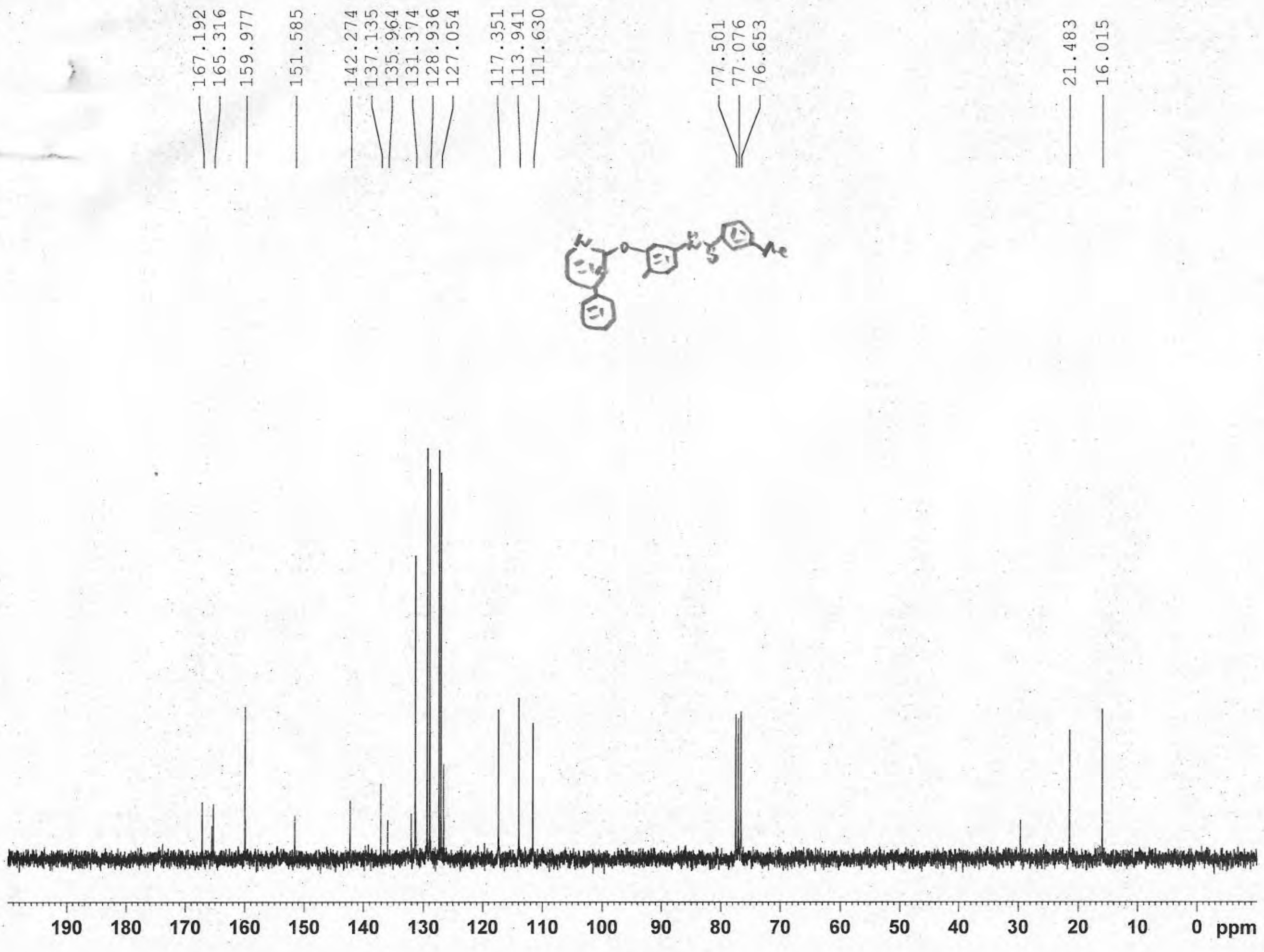

B4

SP121202-2 1HNMR CDCL3 303K AV-300

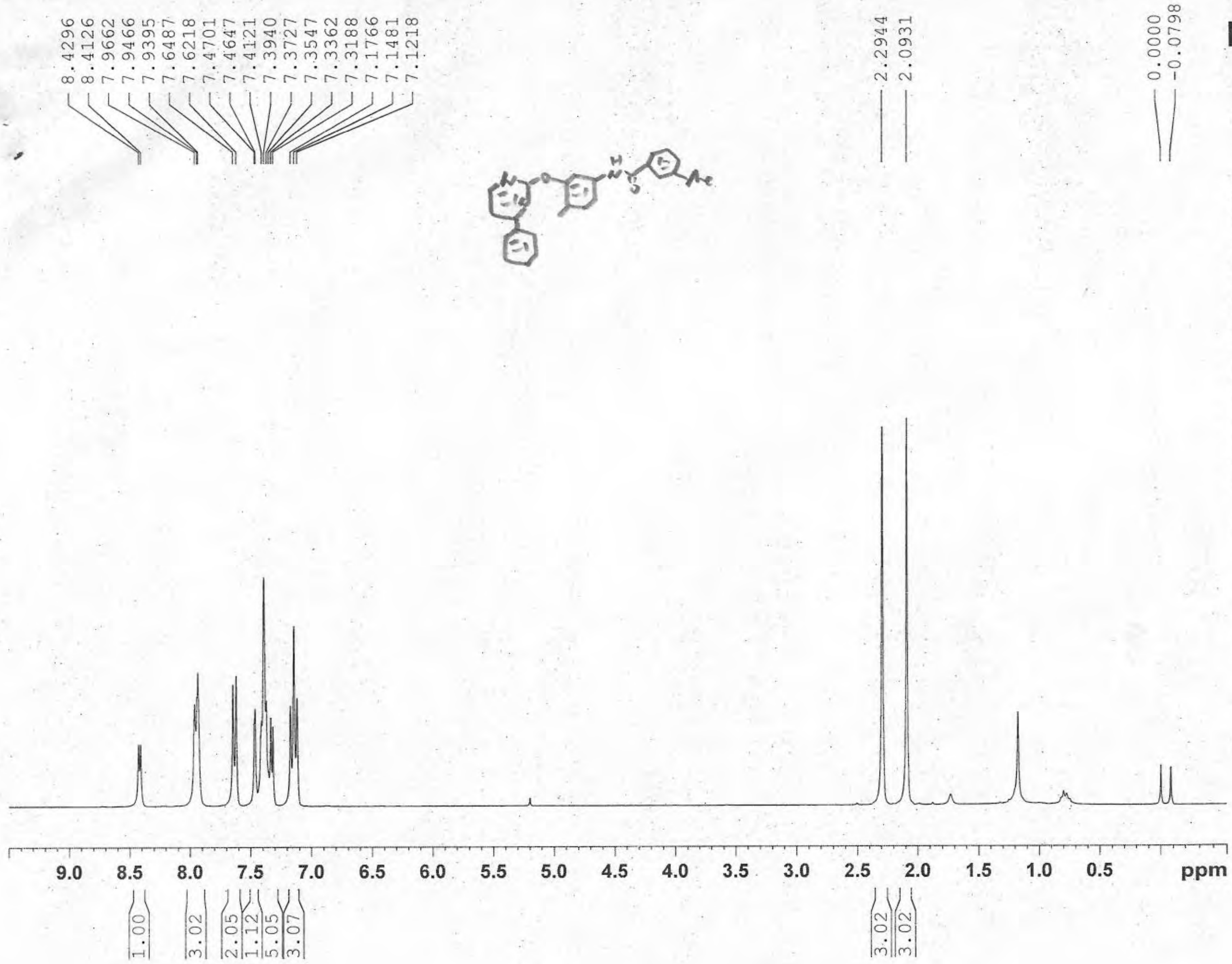

**Sample Name**		**Position**	p2F3	**Instrument Name**	Instrument 1
**User Name**	QTOF-PC\QTOF	**Inj Vol**	0.1	**InjPosition**	
**Sample Type**	Sample	**IRM Calibration Status**	Success	**Data Filename**	B4-p.d
**ACQ Method**	20110418-MSonly-p.m	**Comment**		**Acquired Time**	3/12/2018 3:34:31 PM

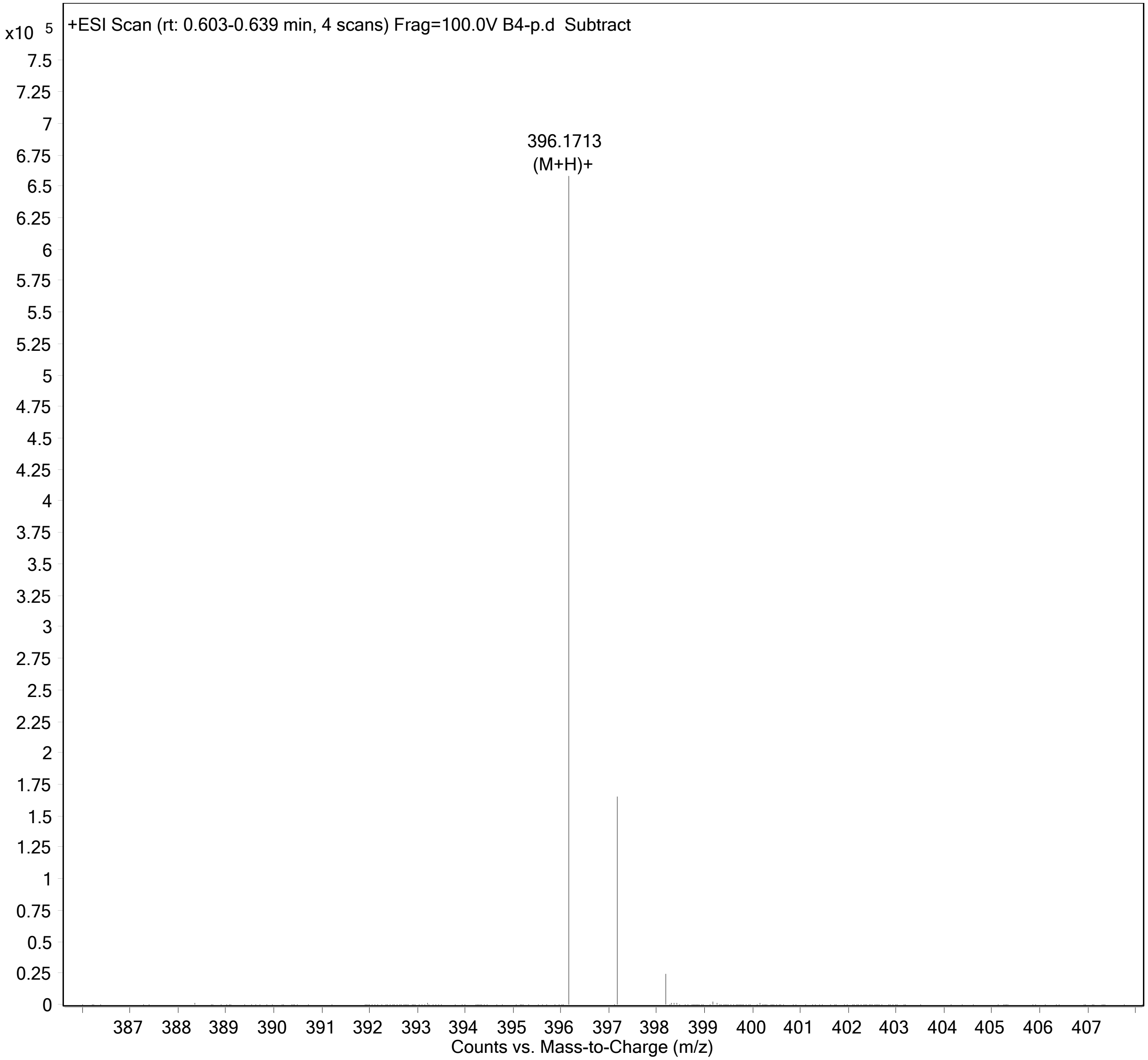

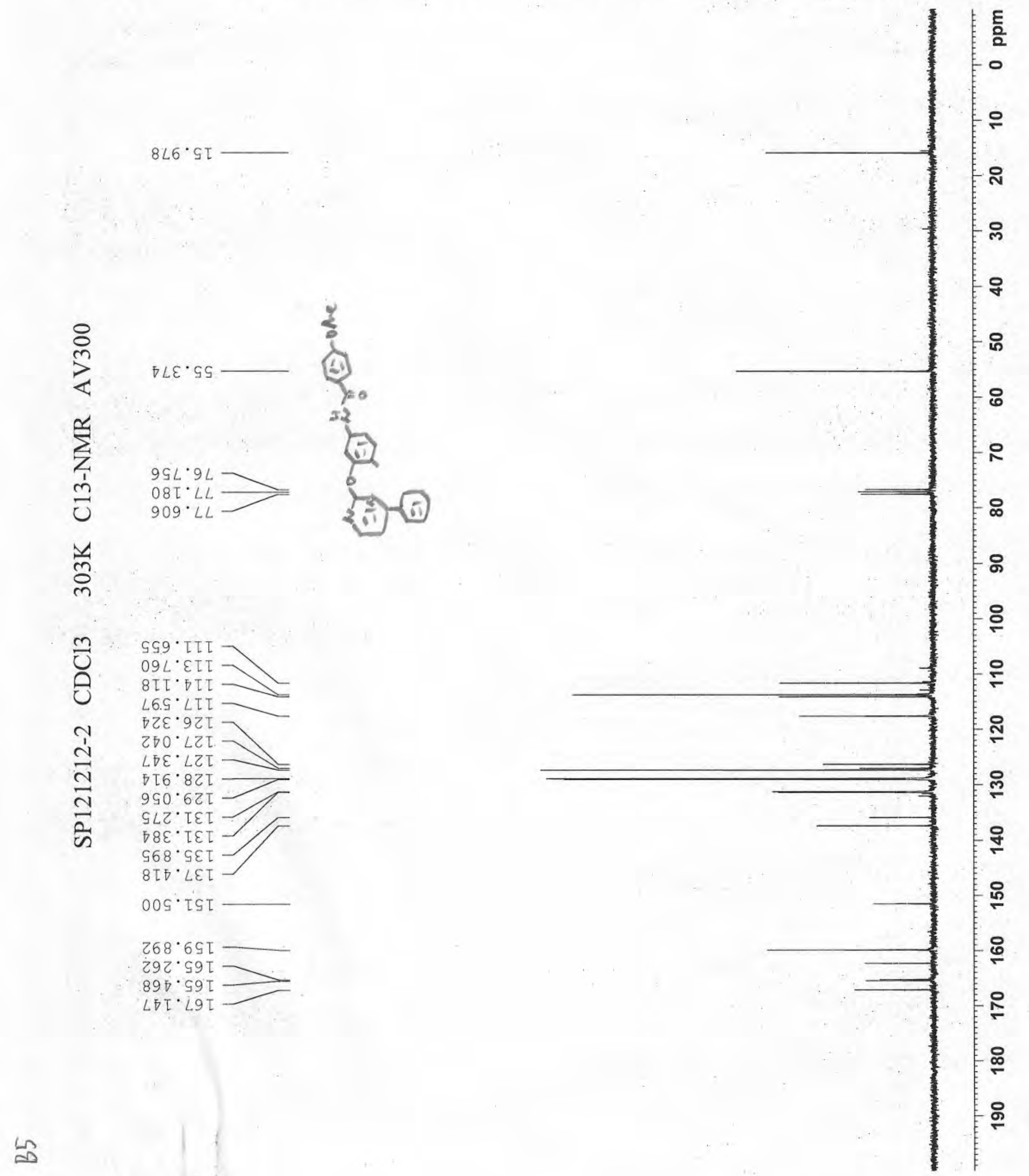
B5
SP121212-2 CDCl3 303K C13-NMR AV300
15.978
55.374
76.756
77.180
77.606
111.655
113.760
114.118
117.597
126.324
127.042
127.347
128.914
129.056
131.275
131.384
135.895
137.418
151.500
159.892
165.262
165.468
167.147
ppm

B5.

SP121212-2 1H-NMR CDCl3 303K AV-300

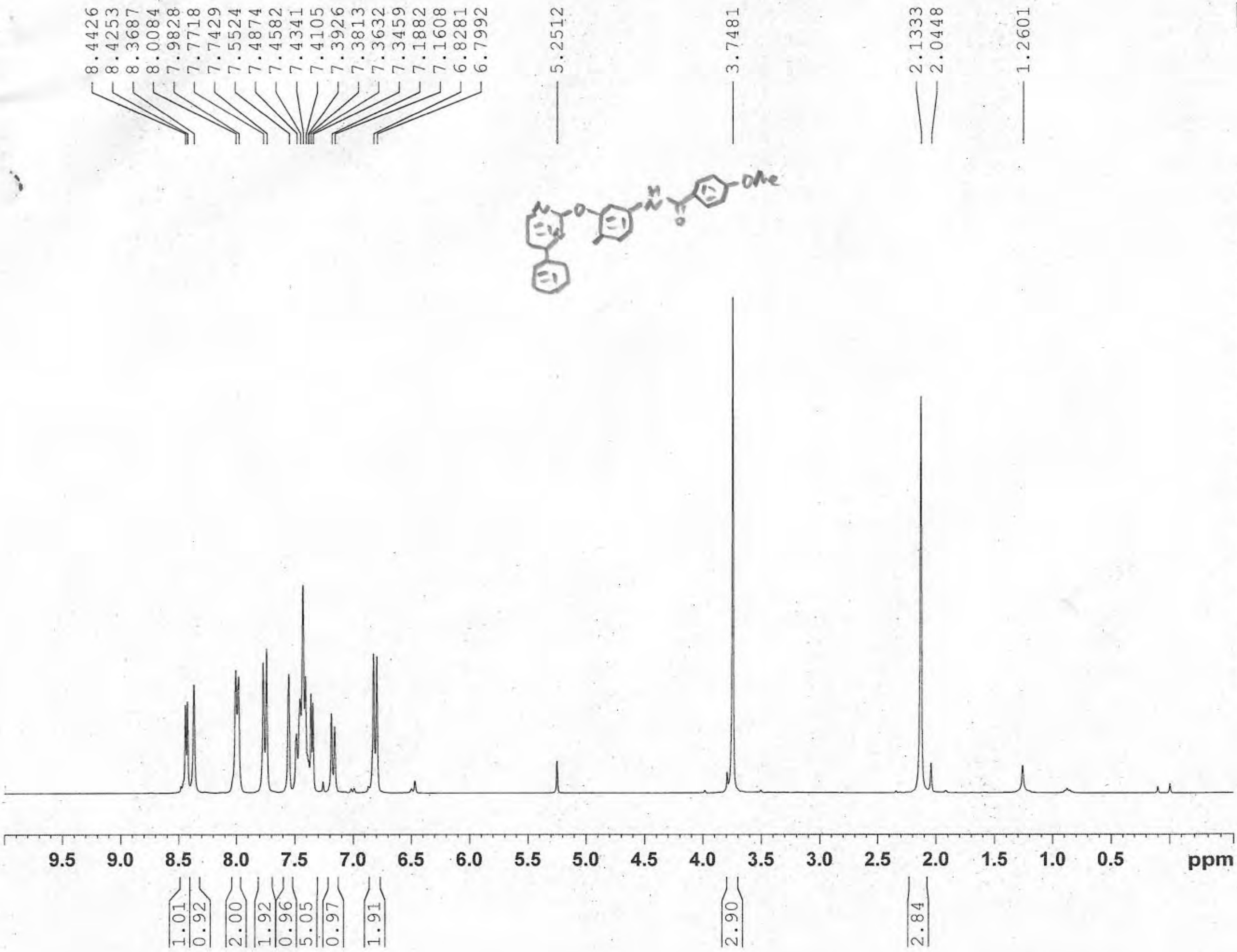

**Sample Name**		**Position**	p2F4	**Instrument Name**	Instrument 1
**User Name**	QTOF-PC\QTOF	**Inj Vol**	0.1	**InjPosition**	
**Sample Type**	Sample	**IRM Calibration Status**	Success	**Data Filename**	B5-p.d
**ACQ Method**	20110418-MSonly-p.m	**Comment**		**Acquired Time**	3/12/2018 3:36:31 PM

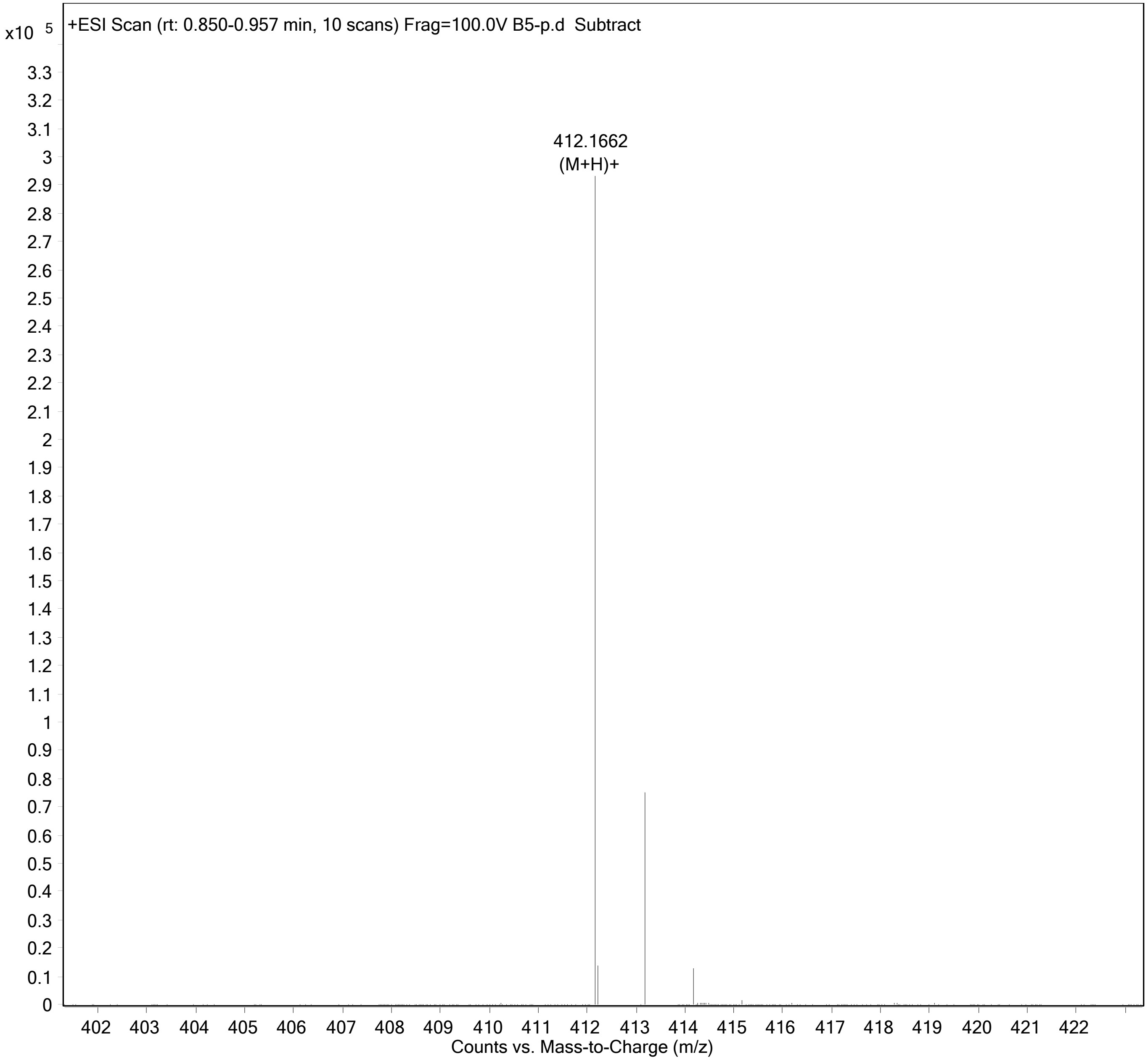

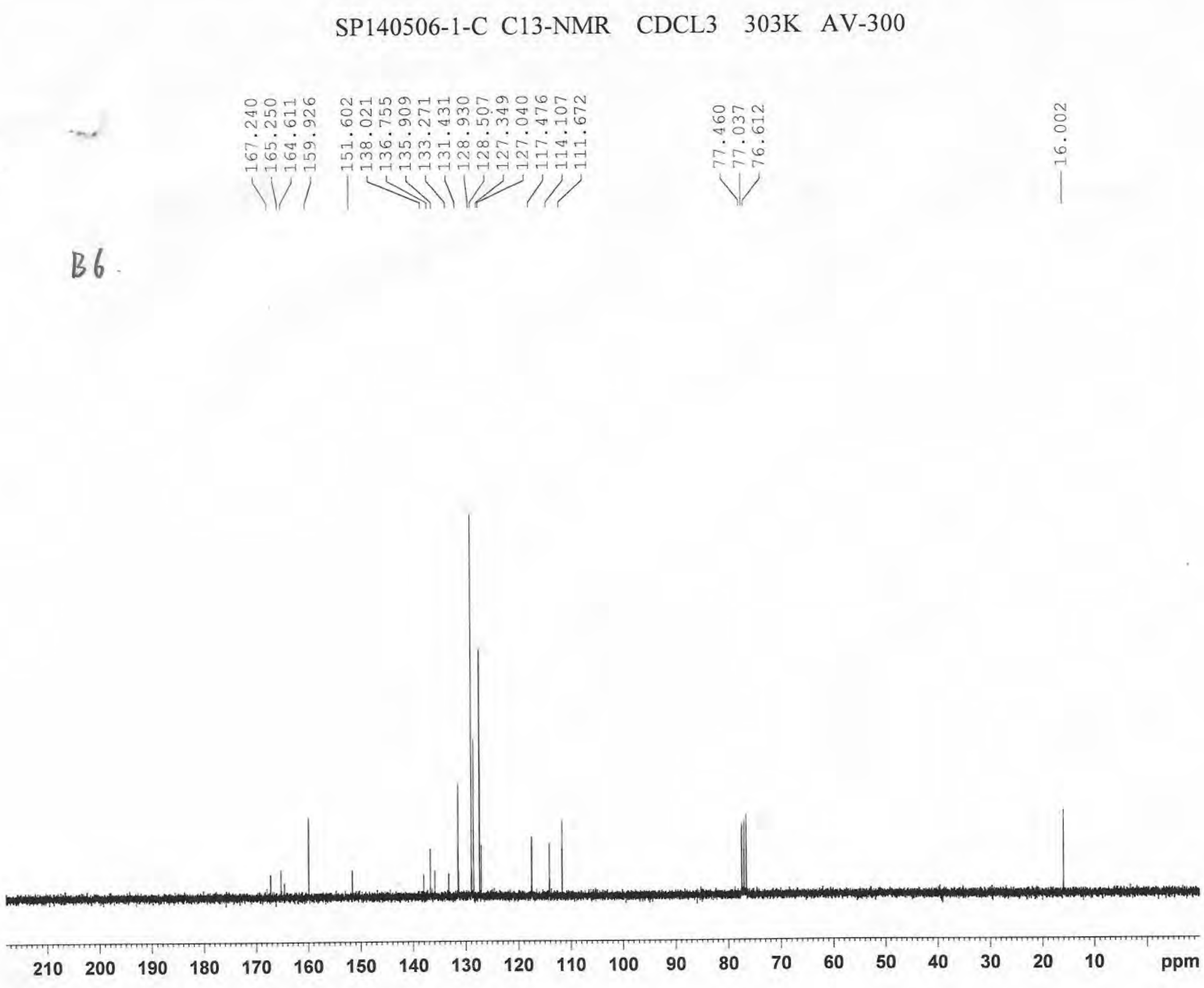
SP140506-1-C C13-NMR CDCL3 303K AV-300
167.240
165.250
164.611
159.926
151.602
138.021
136.755
135.909
133.271
131.431
128.930
128.507
127.349
127.040
117.476
114.107
111.672
77.460
77.037
76.612
16.002
B6.
210
200
190
180
170
160
150
140
130
120
110
100
90
80
70
60
50
40
30
20
10
ppm

B6

SP20131108-4 CDCL3 1HNMR AV300

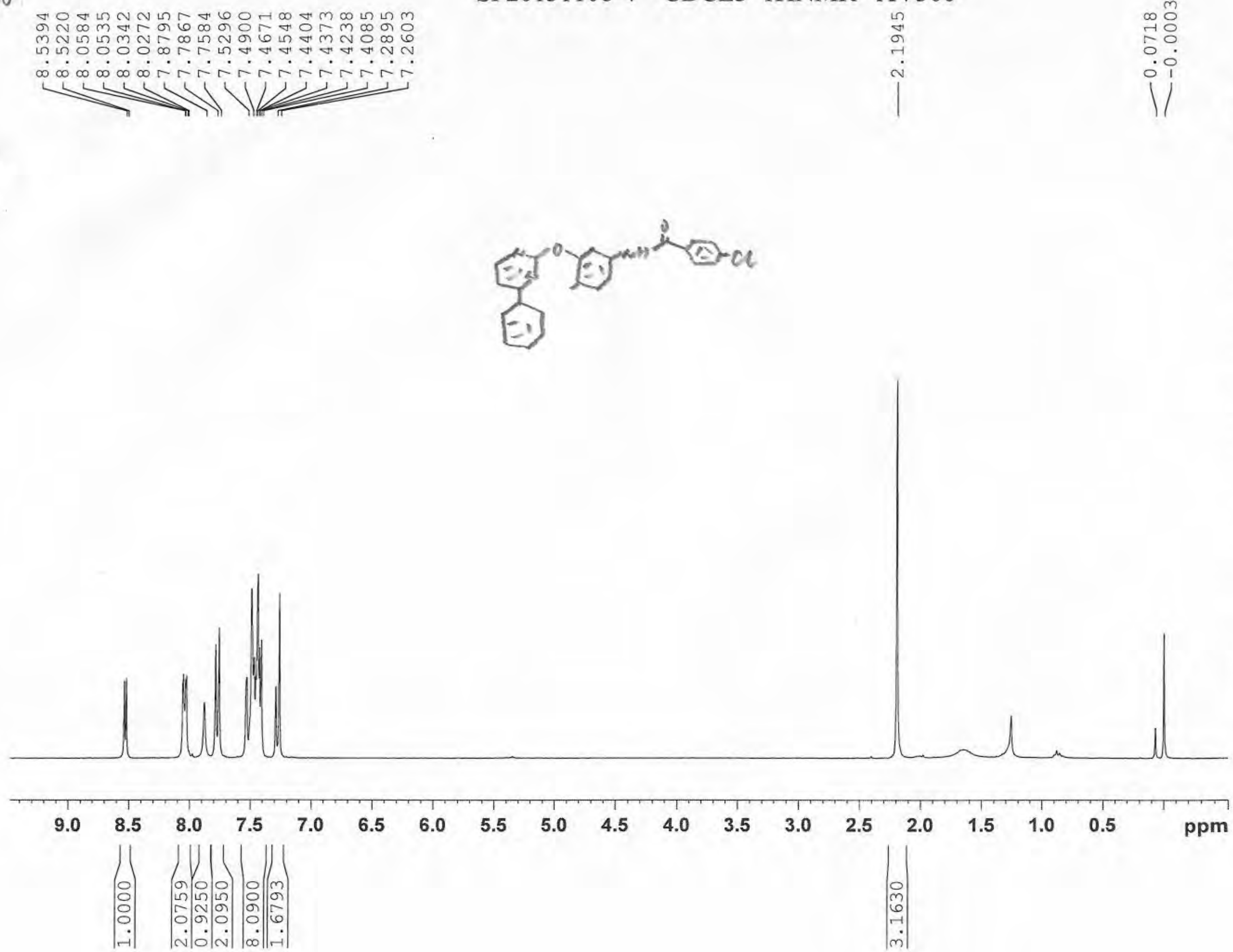

**Sample Name**		**Position**	p2F5	**Instrument Name**	Instrument 1
**User Name**	QTOF-PC\QTOF	**Inj Vol**	0.1	**InjPosition**	
**Sample Type**	Sample	**IRM Calibration Status**	Success	**Data Filename**	B6-p.d
**ACQ Method**	20110418-MSonly-p.m	**Comment**		**Acquired Time**	3/12/2018 3:39:16 PM

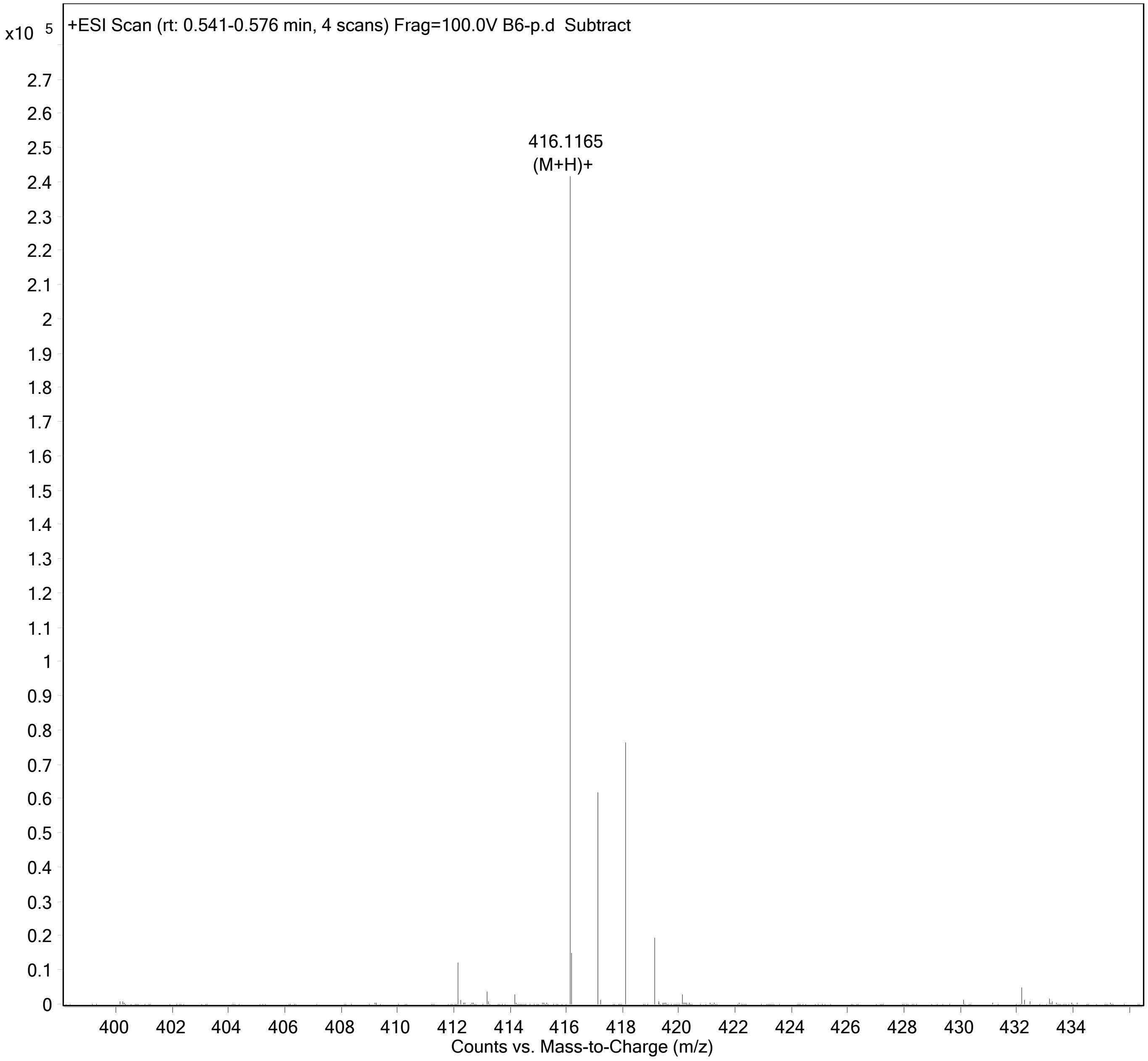

B7

SP121205-3 CDCl3 303K C13-NMR AV300

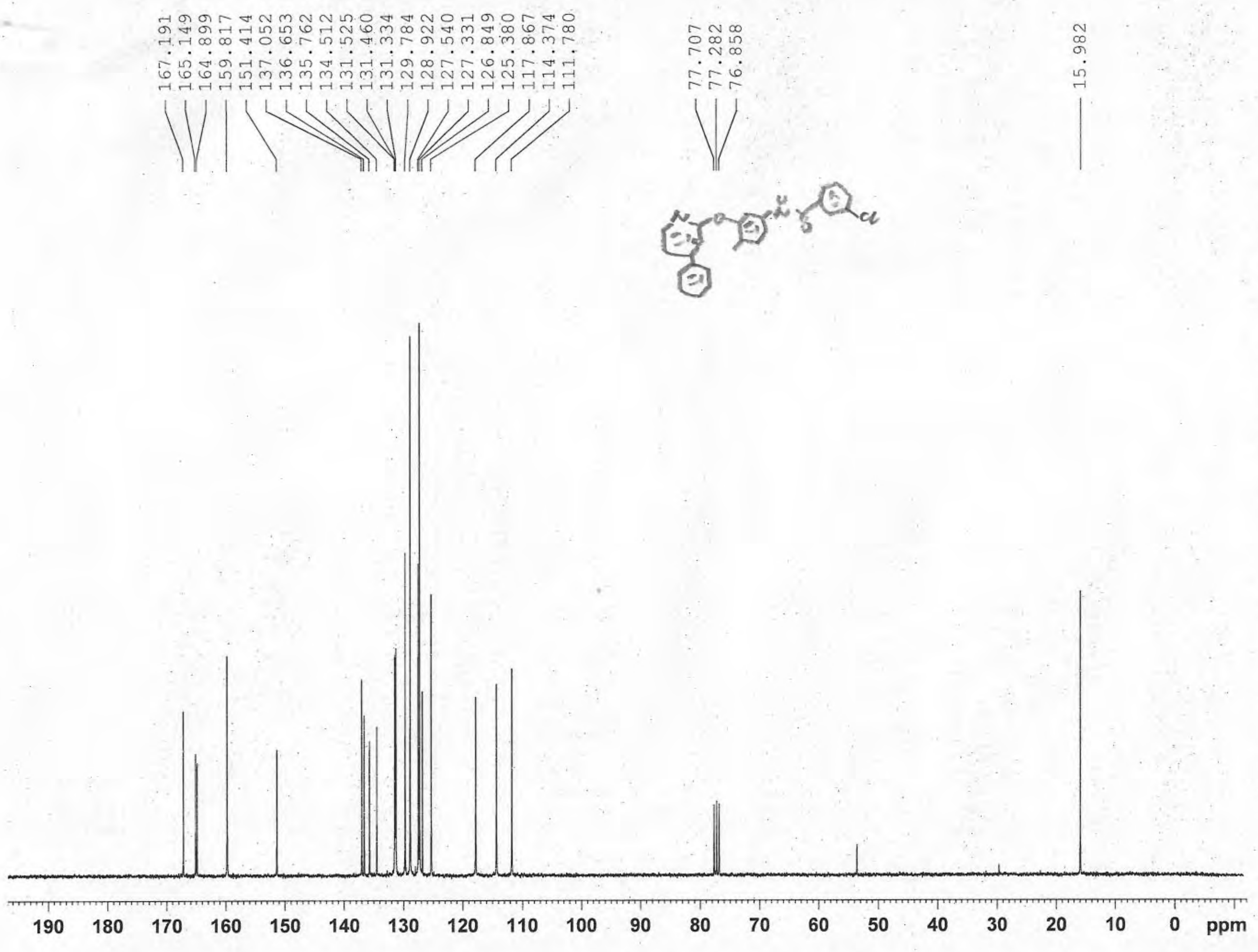

B7

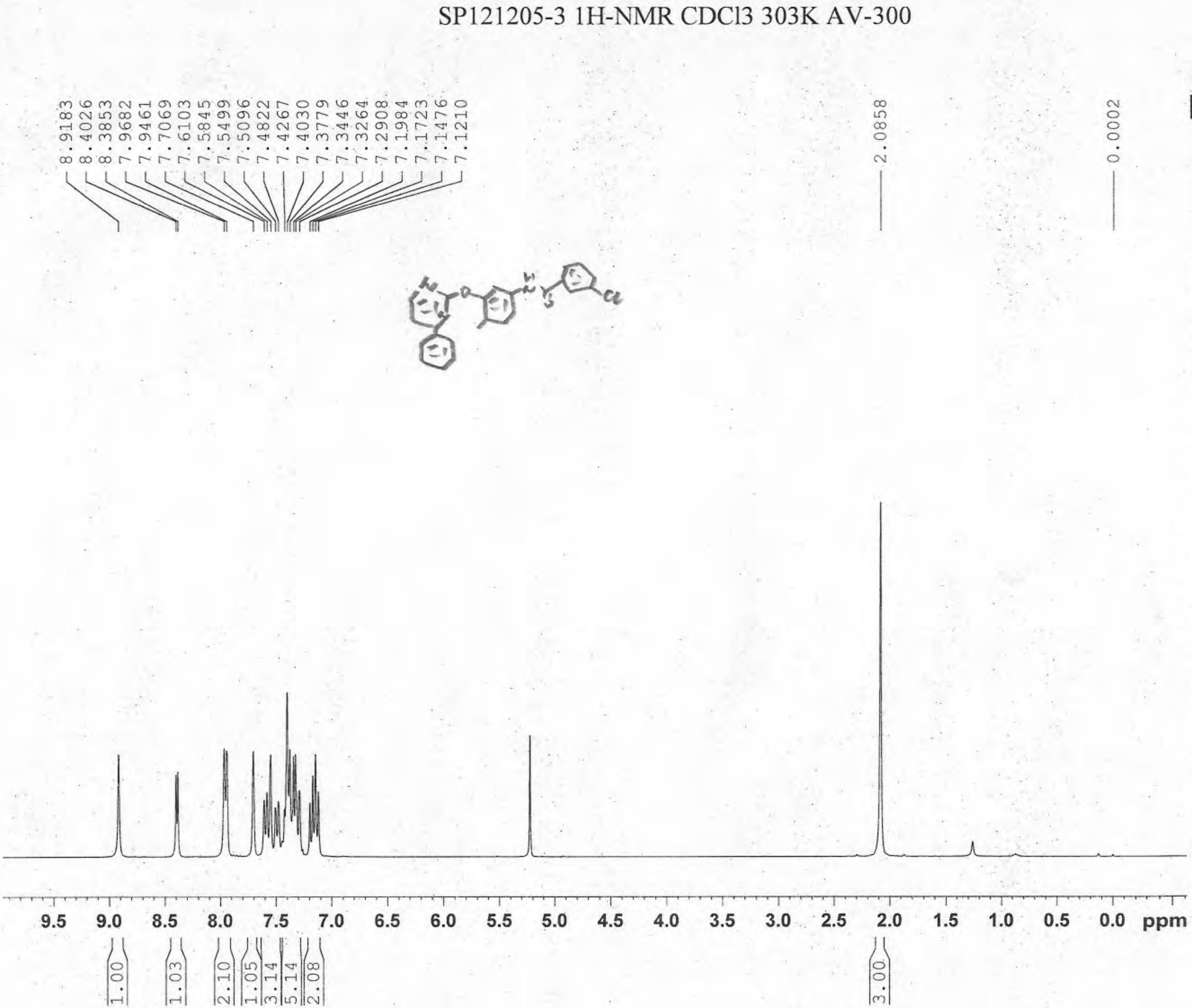
SP121205-3 1H-NMR CDCl3 303K AV-300
8.9183
8.4026
8.3853
7.9682
7.9461
7.7069
7.6103
7.5845
7.5499
7.5096
7.4822
7.4267
7.4030
7.3779
7.3446
7.3264
7.2908
7.1984
7.1723
7.1476
7.1210
2.0858
0.0002
9.5 9.0 8.5 8.0 7.5 7.0 6.5 6.0 5.5 5.0 4.5 4.0 3.5 3.0 2.5 2.0 1.5 1.0 0.5 0.0 ppm
1.00
1.03
2.10
1.05
3.14
5.14
2.08
3.00

**Sample Name**		**Position**	p2F6	**Instrument Name**	Instrument 1
**User Name**	QTOF-PC\QTOF	**Inj Vol**	0.1	**InjPosition**	
**Sample Type**	Sample	**IRM Calibration Status**	Success	**Data Filename**	B7-p.d
**ACQ Method**	20110418-MSonly-p.m	**Comment**		**Acquired Time**	3/12/2018 3:41:26 PM

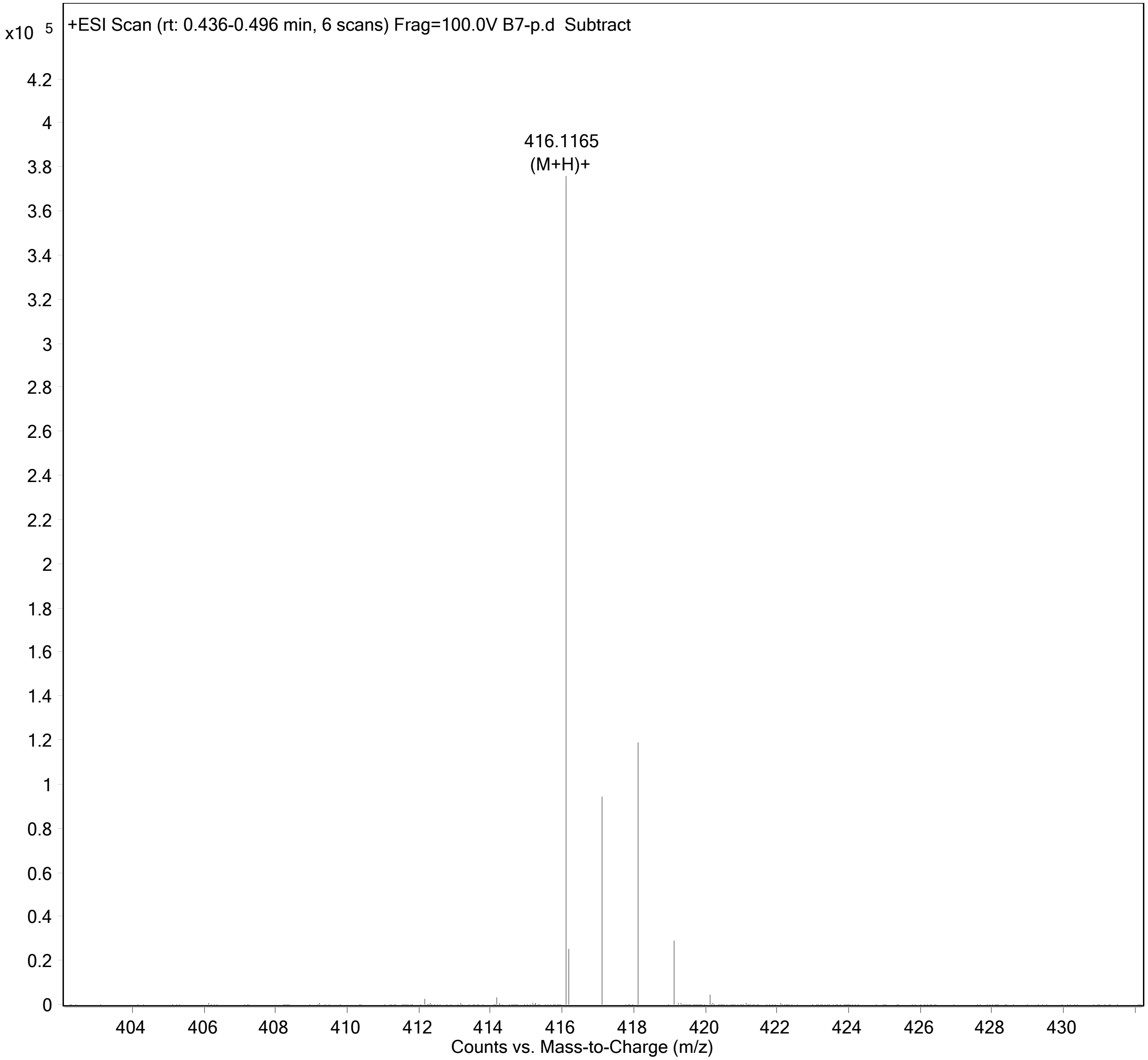

B8

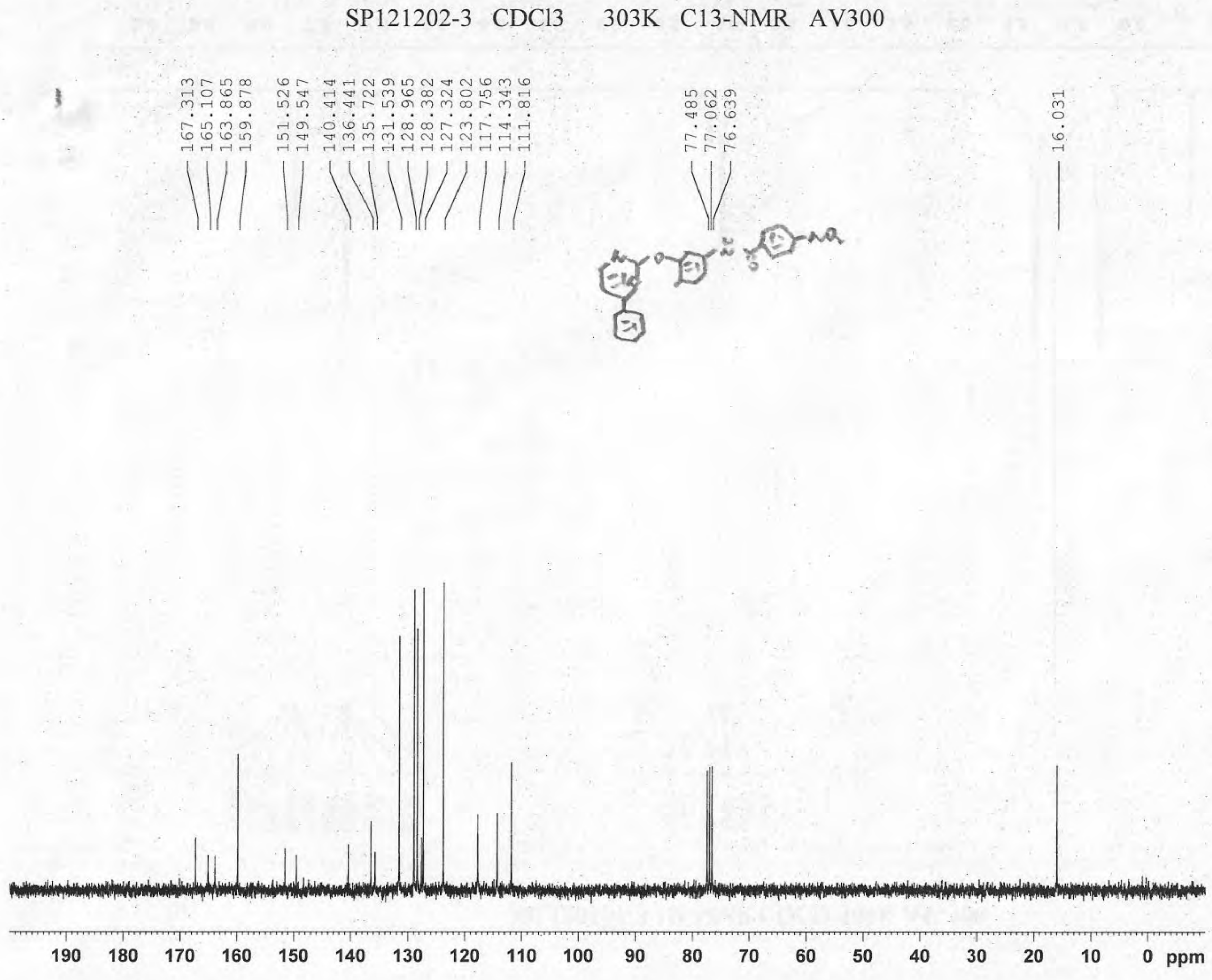
SP121202-3 CDCl3 303K C13-NMR AV300
167.313
165.107
163.865
159.878
151.526
149.547
140.414
136.441
135.722
131.539
128.965
128.382
127.324
123.802
117.756
114.343
111.816
77.485
77.062
76.639
16.031
190 180 170 160 150 140 130 120 110 100 90 80 70 60 50 40 30 20 10 0 ppm

B8

SP140506-2 CDCL3 1HNMR AV300

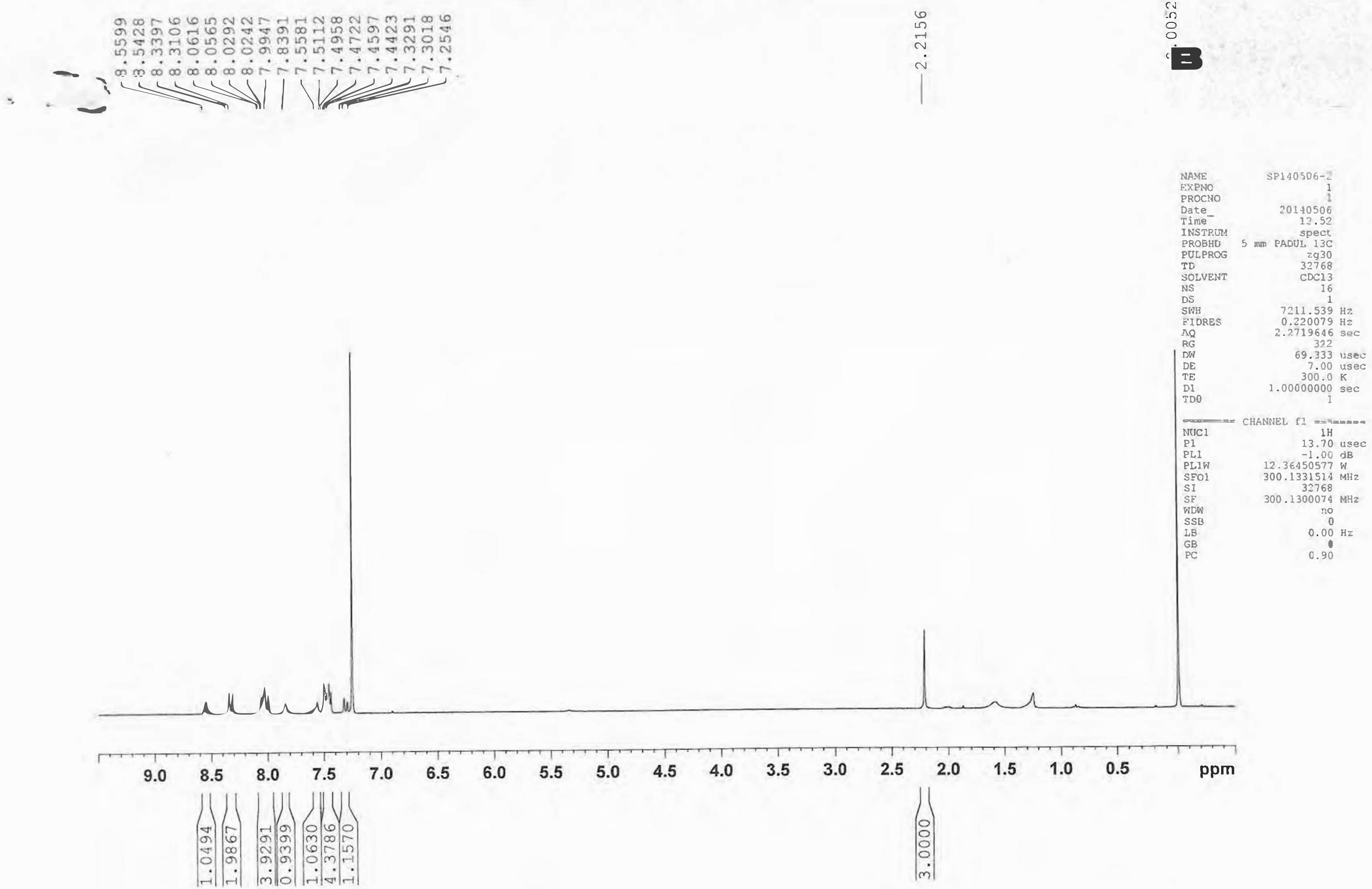

**Sample Name**		**Position**	p2F7	**Instrument Name**	Instrument 1
**User Name**	QTOF-PC\QTOF	**Inj Vol**	0.1	**InjPosition**	
**Sample Type**	Sample	**IRM Calibration Status**	Success	**Data Filename**	B8-p.d
**ACQ Method**	20110418-MSonly-p.m	**Comment**		**Acquired Time**	3/12/2018 3:43:49 PM

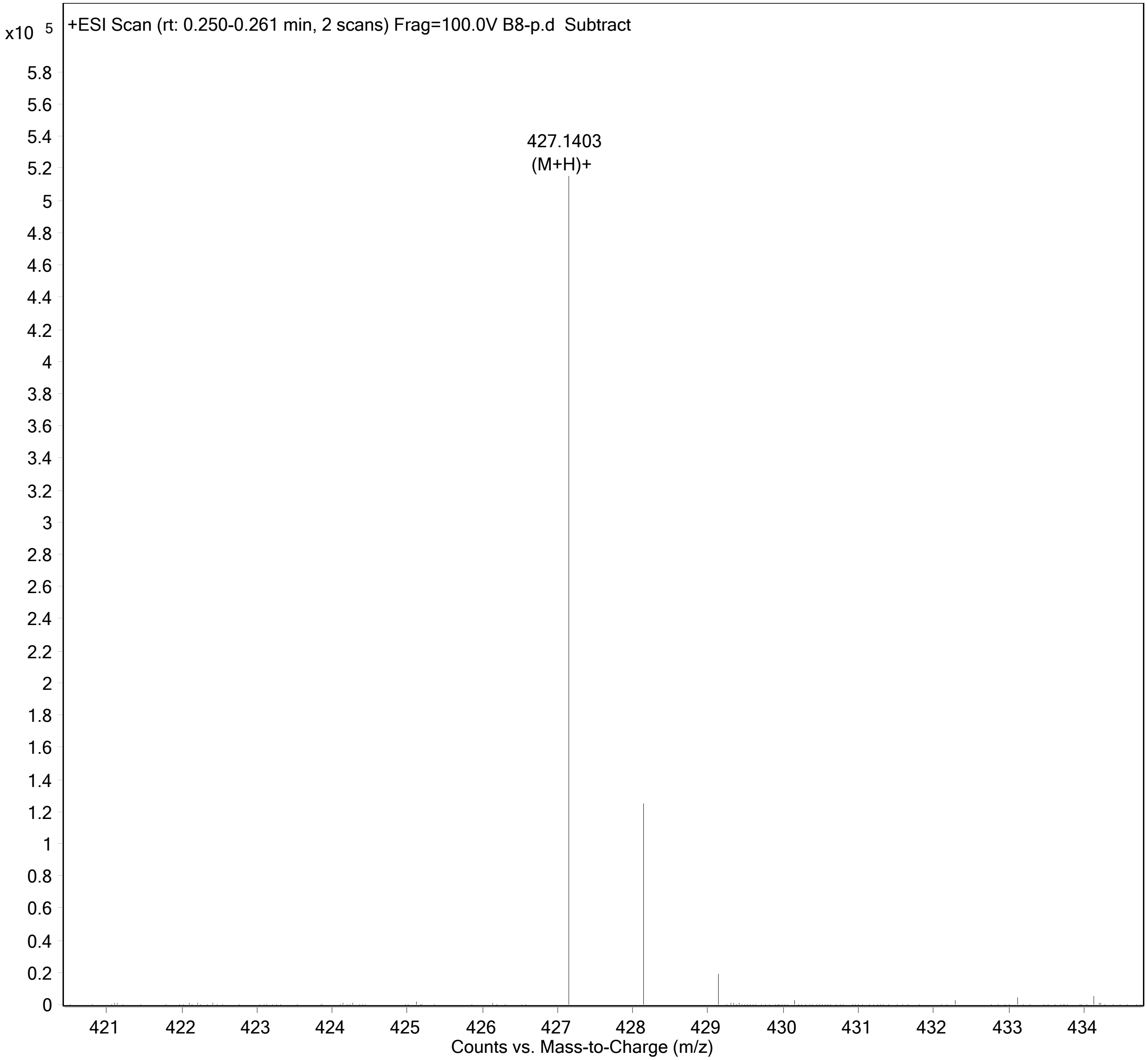

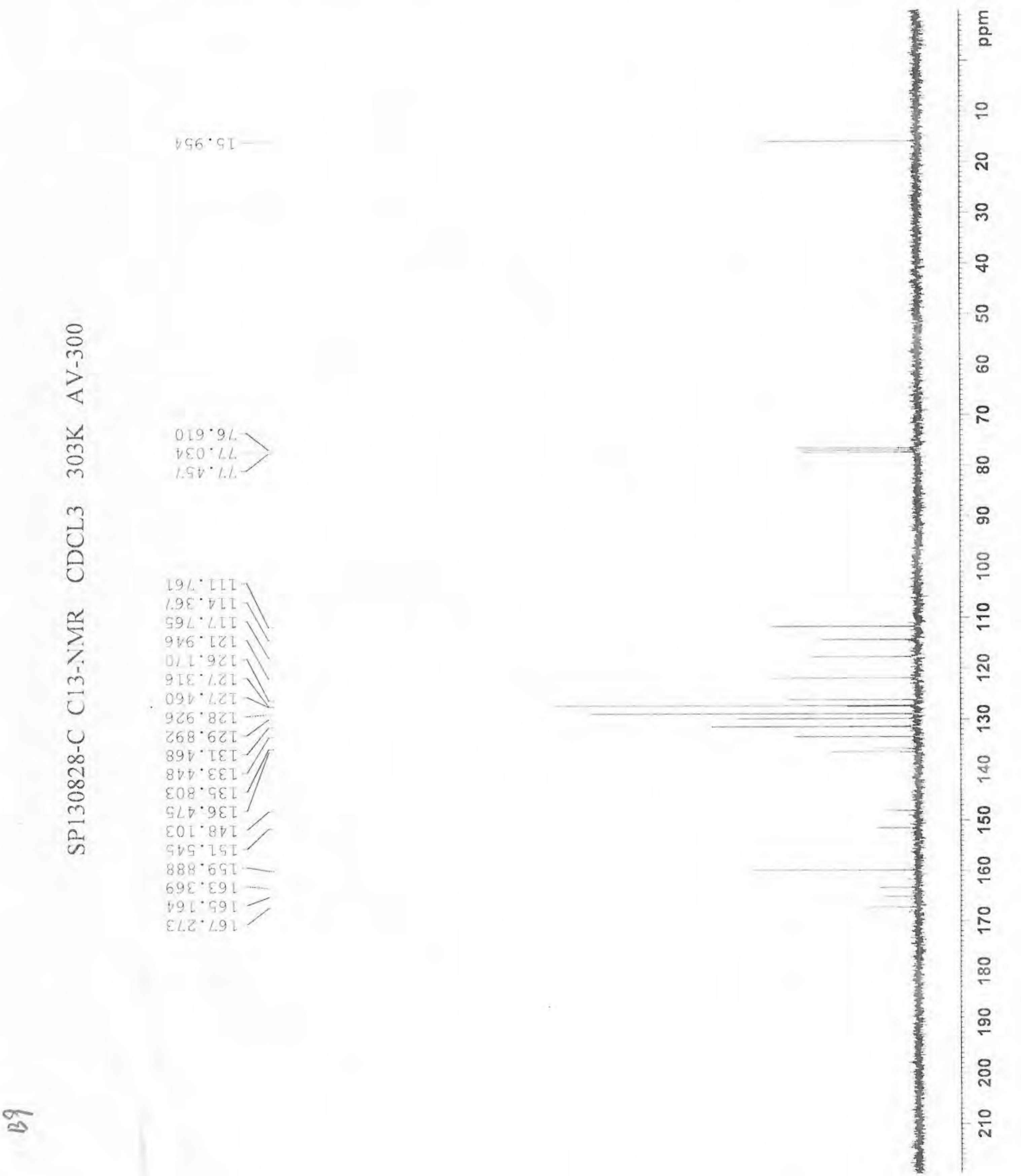
B9
SP130828-C C13-NMR CDCL3 303K AV-300
15.954
76.610
77.034
77.457
111.761
114.367
117.765
121.946
126.170
127.316
127.460
128.926
129.892
131.468
133.448
135.803
136.475
148.103
151.545
159.888
163.369
165.164
167.273
210 200 190 180 170 160 150 140 130 120 110 100 90 80 70 60 50 40 30 20 10 ppm

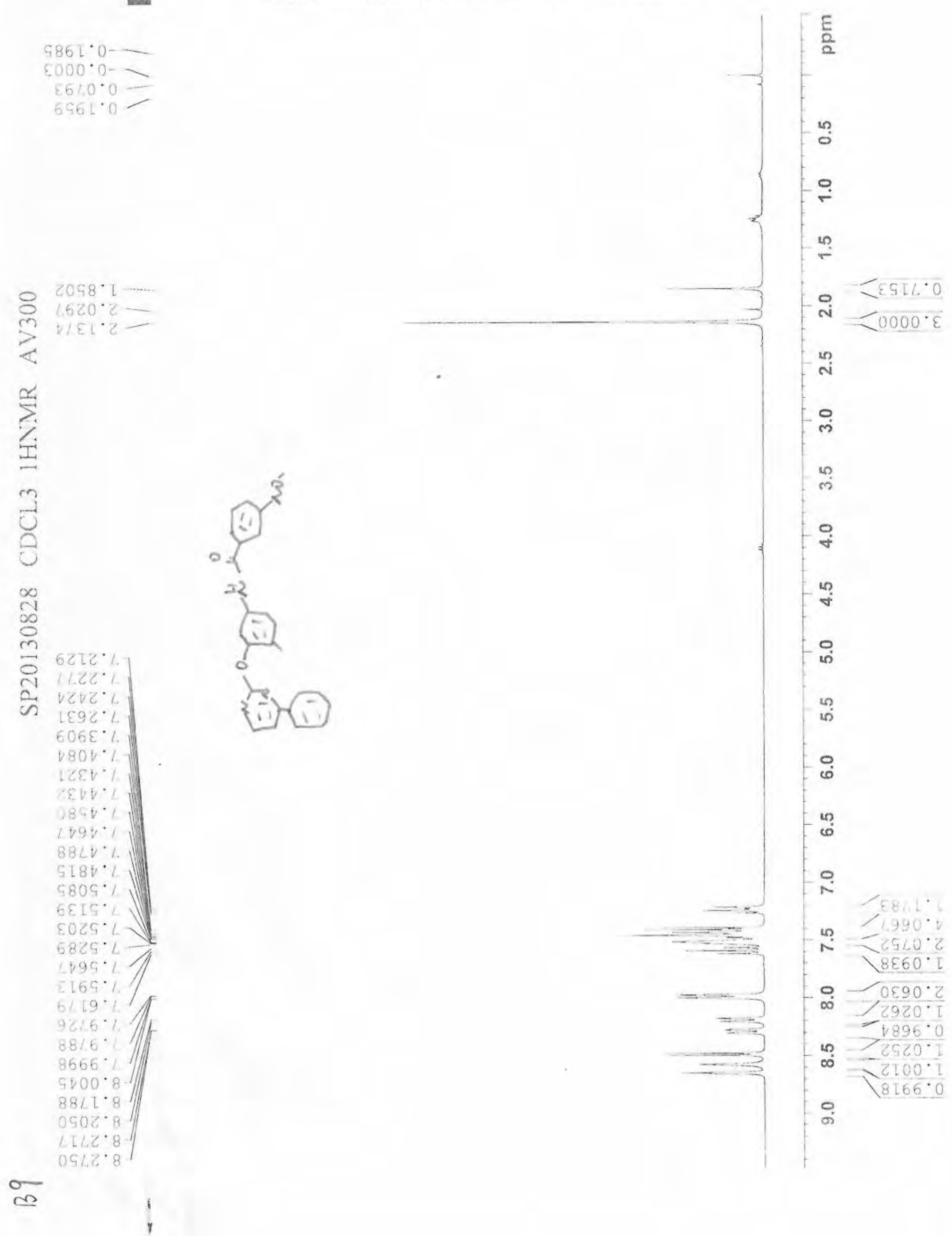
SP20130828 CDCL3 1HNMR AV300
8.2750
8.2717
8.2050
8.1788
8.0045
7.9998
7.9788
7.9726
7.6179
7.5913
7.5647
7.5289
7.5203
7.5139
7.5085
7.4815
7.4788
7.4647
7.4580
7.4432
7.4321
7.4084
7.3909
7.2631
7.2424
7.2277
7.2129
2.1374
2.0297
1.8502
0.1959
0.0793
-0.0003
-0.1985
0.7153
3.0000
1.1783
4.0667
2.0752
1.0938
2.0630
1.0262
0.9684
1.0252
1.0012
0.9918
ppm

**Sample Name**		**Position**	p2F8	**Instrument Name**	Instrument 1
**User Name**	QTOF-PC\QTOF	**Inj Vol**	0.1	**InjPosition**	
**Sample Type**	Sample	**IRM Calibration Status**	Success	**Data Filename**	B9-p.d
**ACQ Method**	20110418-MSonly-p.m	**Comment**		**Acquired Time**	3/12/2018 3:45:31 PM

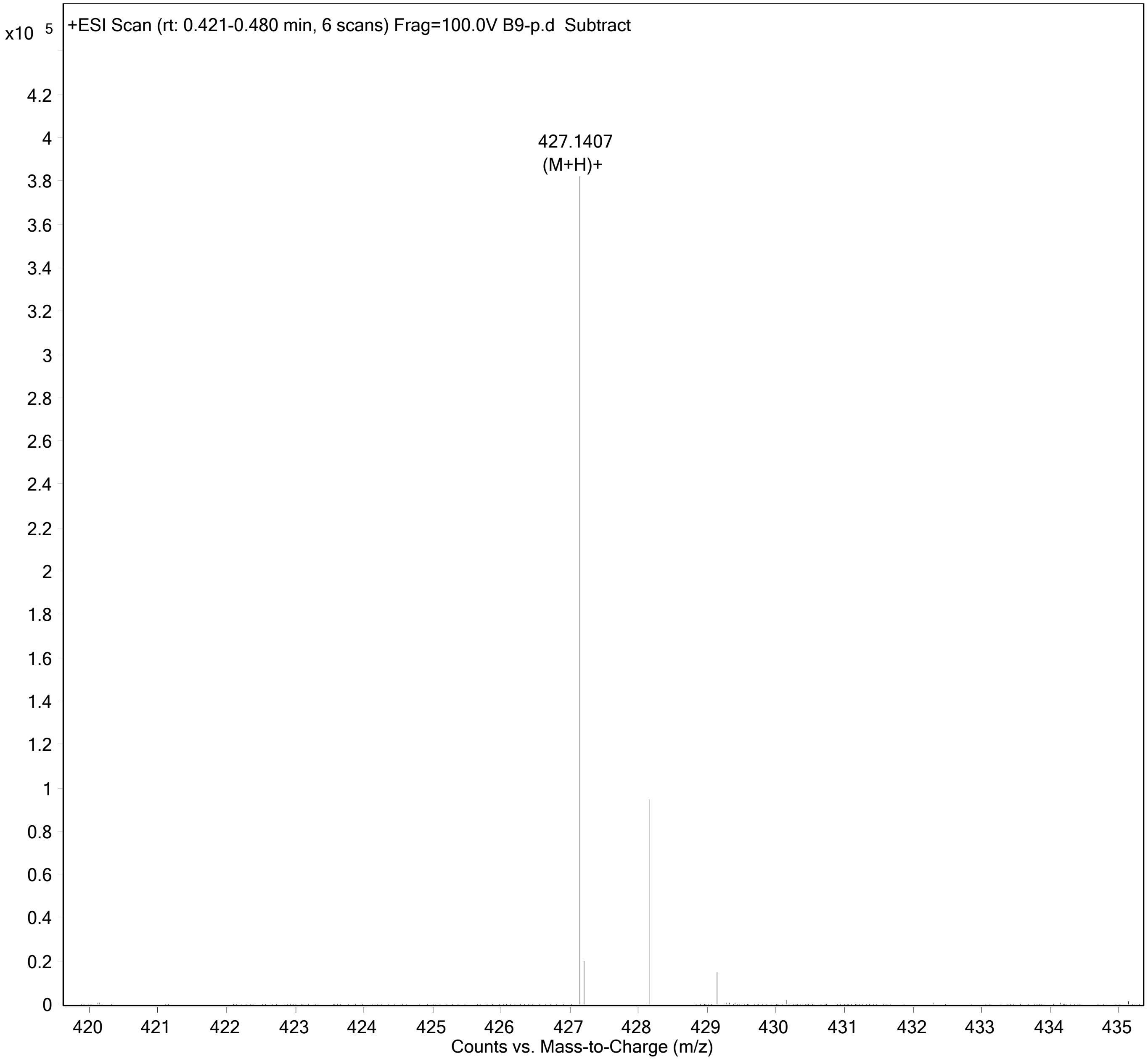

B10

SP20131108-3-C C13-NMR CDCL3 303K AV-300

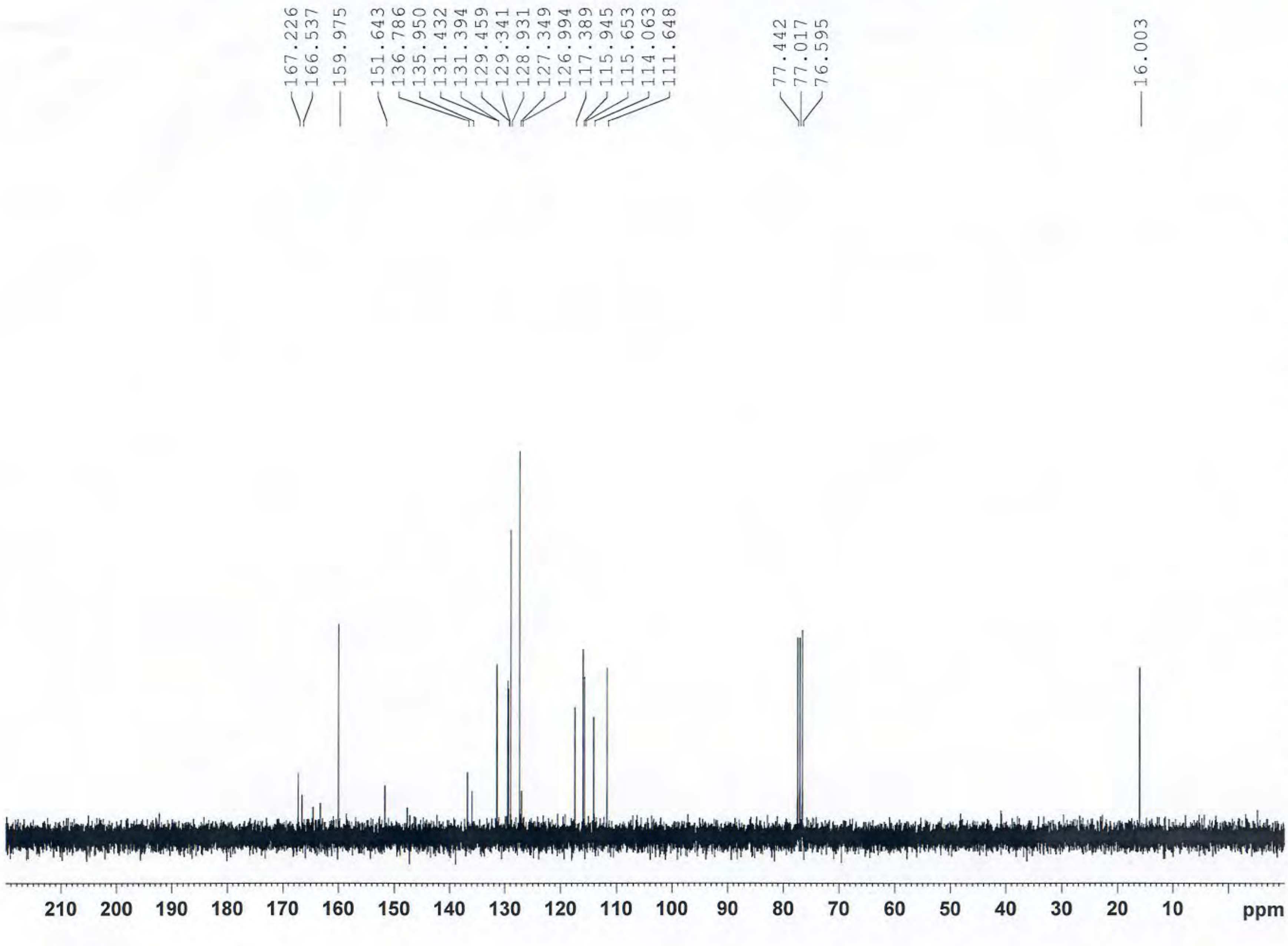

B10

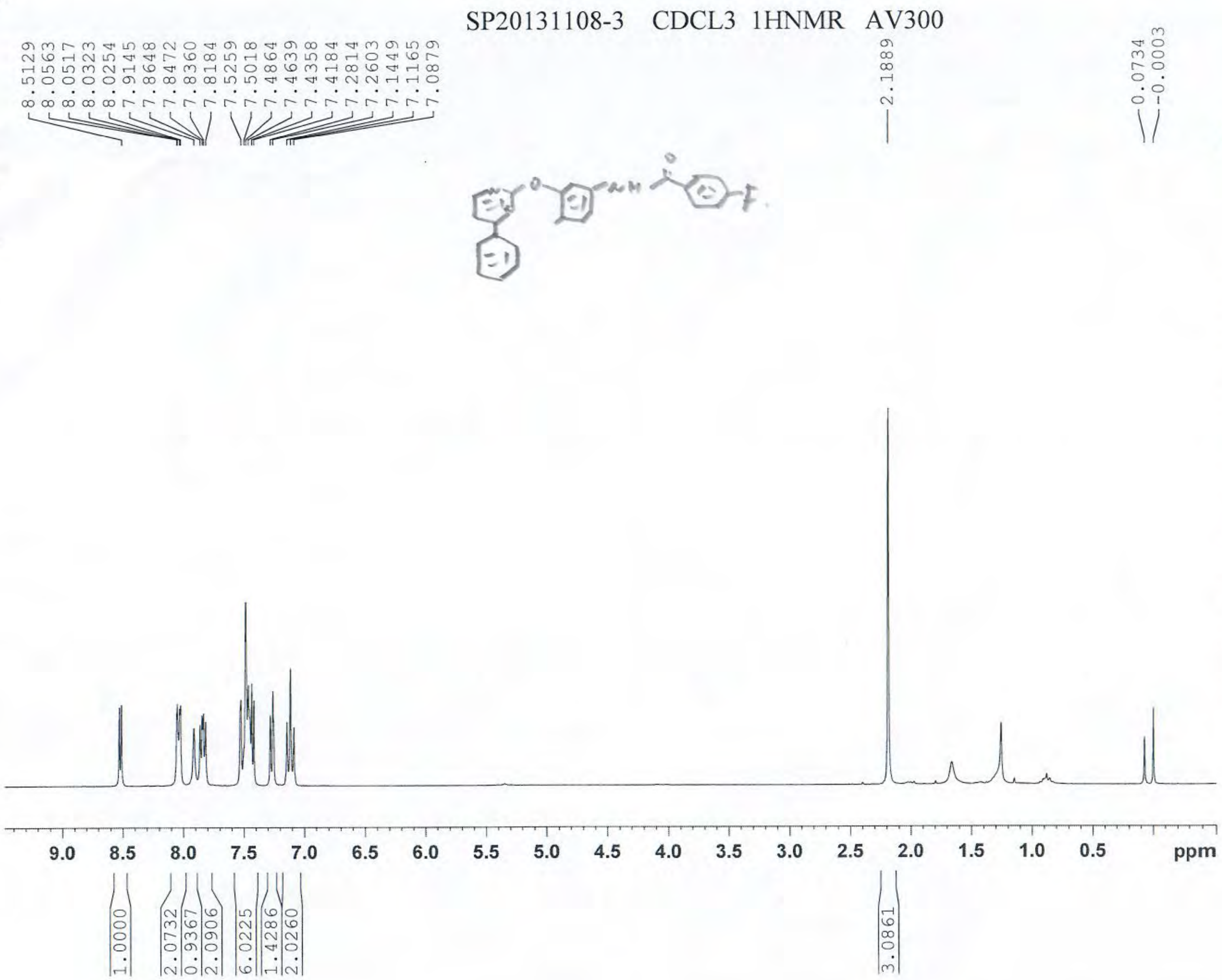
SP20131108-3 CDCL3 1HNMR AV300
8.5129
8.0563
8.0517
8.0323
8.0254
7.9145
7.8648
7.8472
7.8360
7.8184
7.5259
7.5018
7.4864
7.4639
7.4358
7.4184
7.2814
7.2603
7.1449
7.1165
7.0879
2.1889
0.0734
-0.0003
9.0
8.5
8.0
7.5
7.0
6.5
6.0
5.5
5.0
4.5
4.0
3.5
3.0
2.5
2.0
1.5
1.0
0.5
ppm
1.0000
2.0732
0.9367
2.0906
6.0225
1.4286
2.0260
3.0861

**Sample Name**		**Position**	p2F9	**Instrument Name**	Instrument 1
**User Name**	QTOF-PC\QTOF	**Inj Vol**	0.1	**InjPosition**	
**Sample Type**	Sample	**IRM Calibration Status**	Success	**Data Filename**	B10-p.d
**ACQ Method**	20110418-MSonly-p.m	**Comment**		**Acquired Time**	3/12/2018 3:53:13 PM

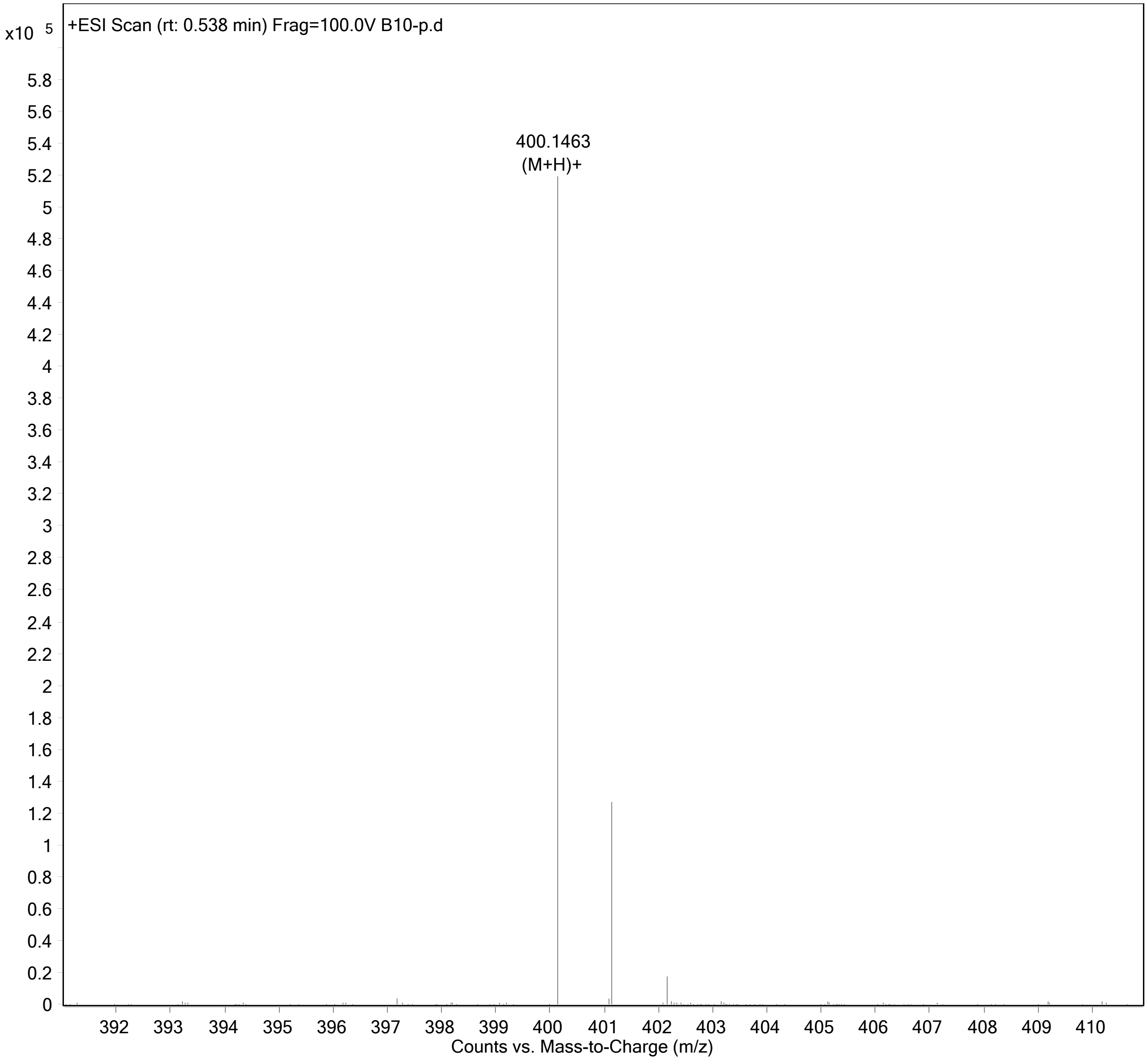

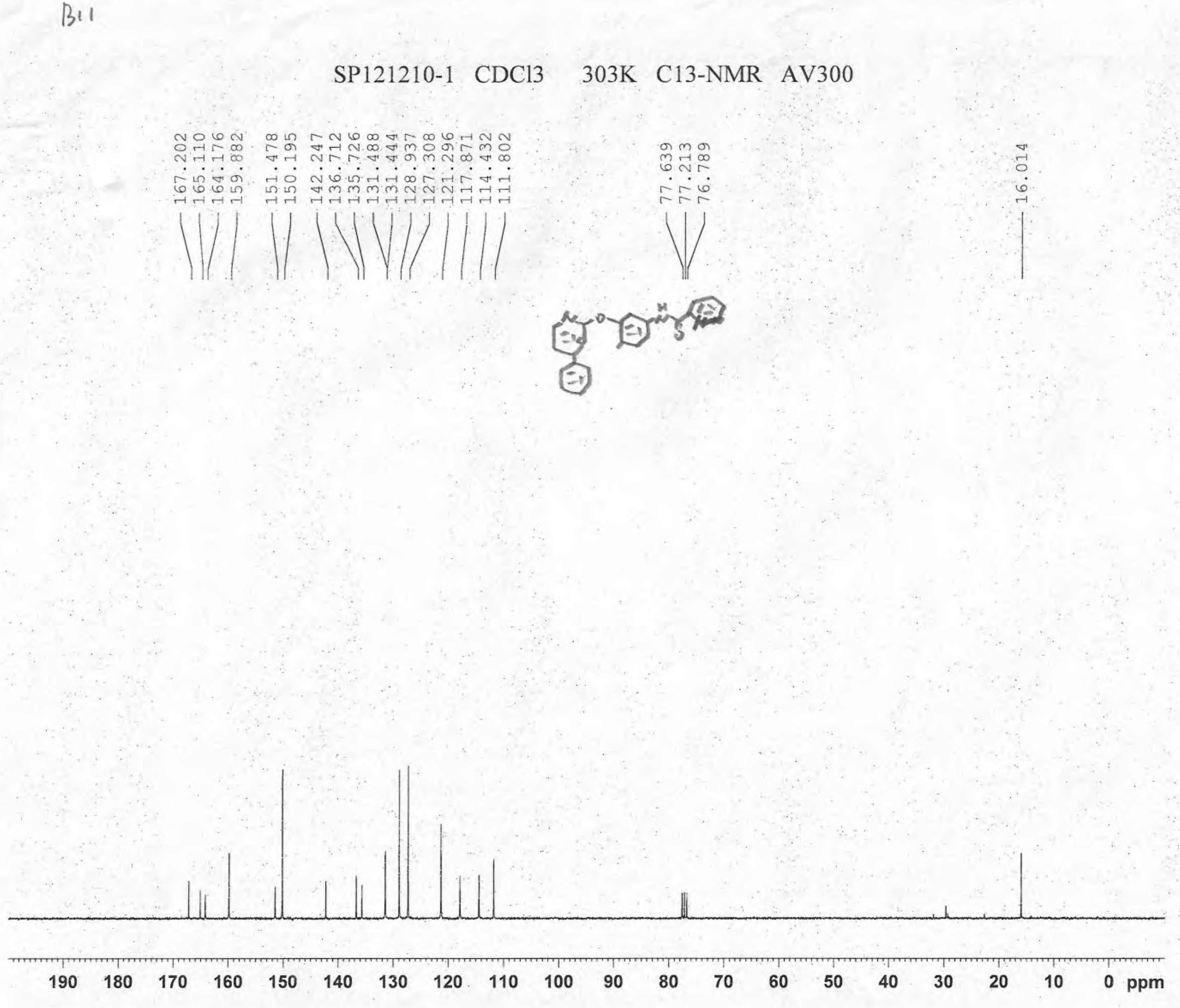
B11
SP121210-1 CDCl3 303K C13-NMR AV300
167.202
165.110
164.176
159.882
151.478
150.195
142.247
136.712
135.726
131.488
131.444
128.937
127.308
121.296
117.871
114.432
111.802
77.639
77.213
76.789
16.014
190 180 170 160 150 140 130 120 110 100 90 80 70 60 50 40 30 20 10 0 ppm

B11

SP121210-1 1H-NMR CDCl3 303K AV-300

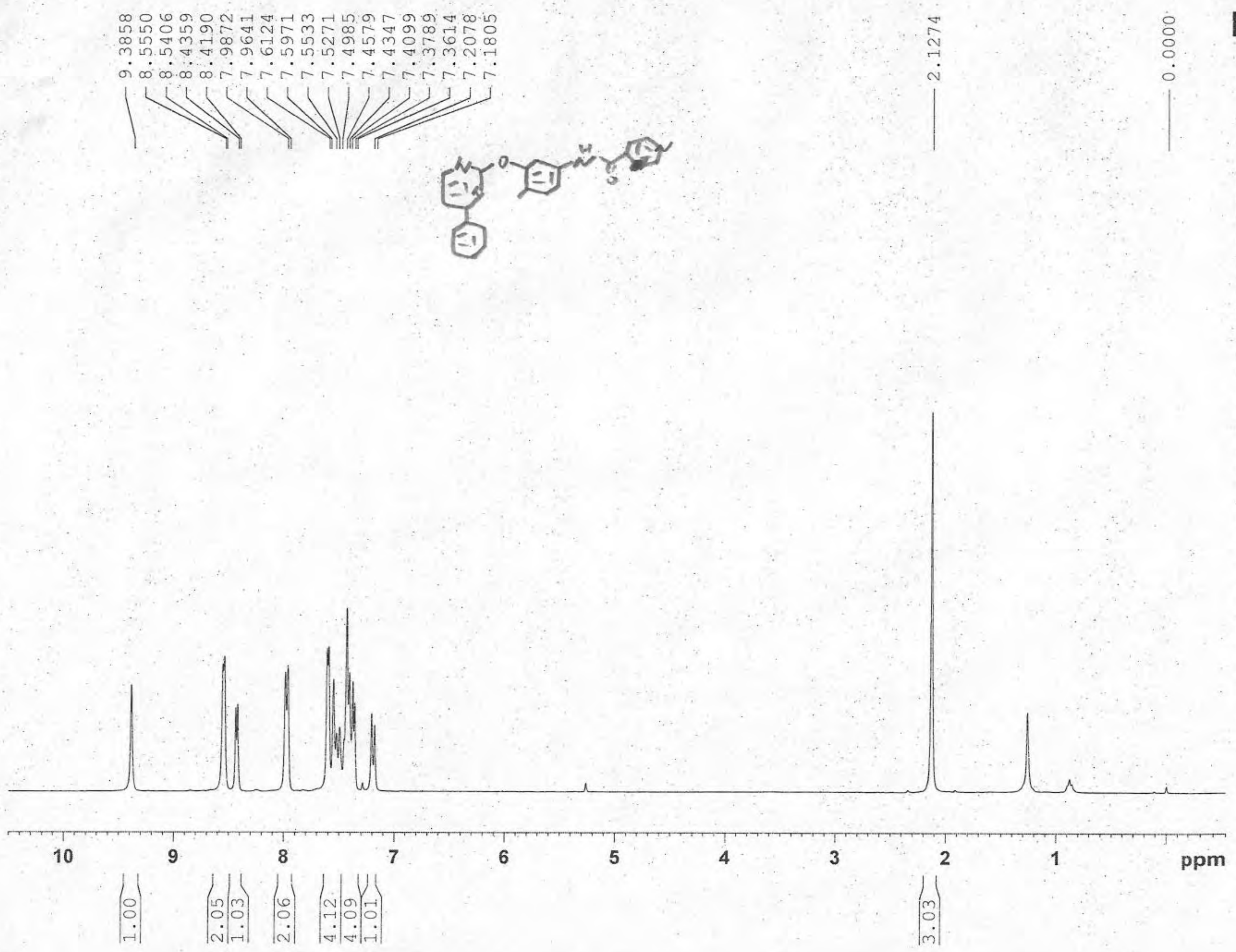

**Sample Name**		**Position**	p1A1	**Instrument Name**	Instrument 1
**User Name**	QTOF-PC\QTOF	**Inj Vol**	0.1	**InjPosition**	
**Sample Type**	Sample	**IRM Calibration Status**	Success	**Data Filename**	B11-p.d
**ACQ Method**	20110418-MSonly-p.m	**Comment**		**Acquired Time**	3/12/2018 3:50:54 PM

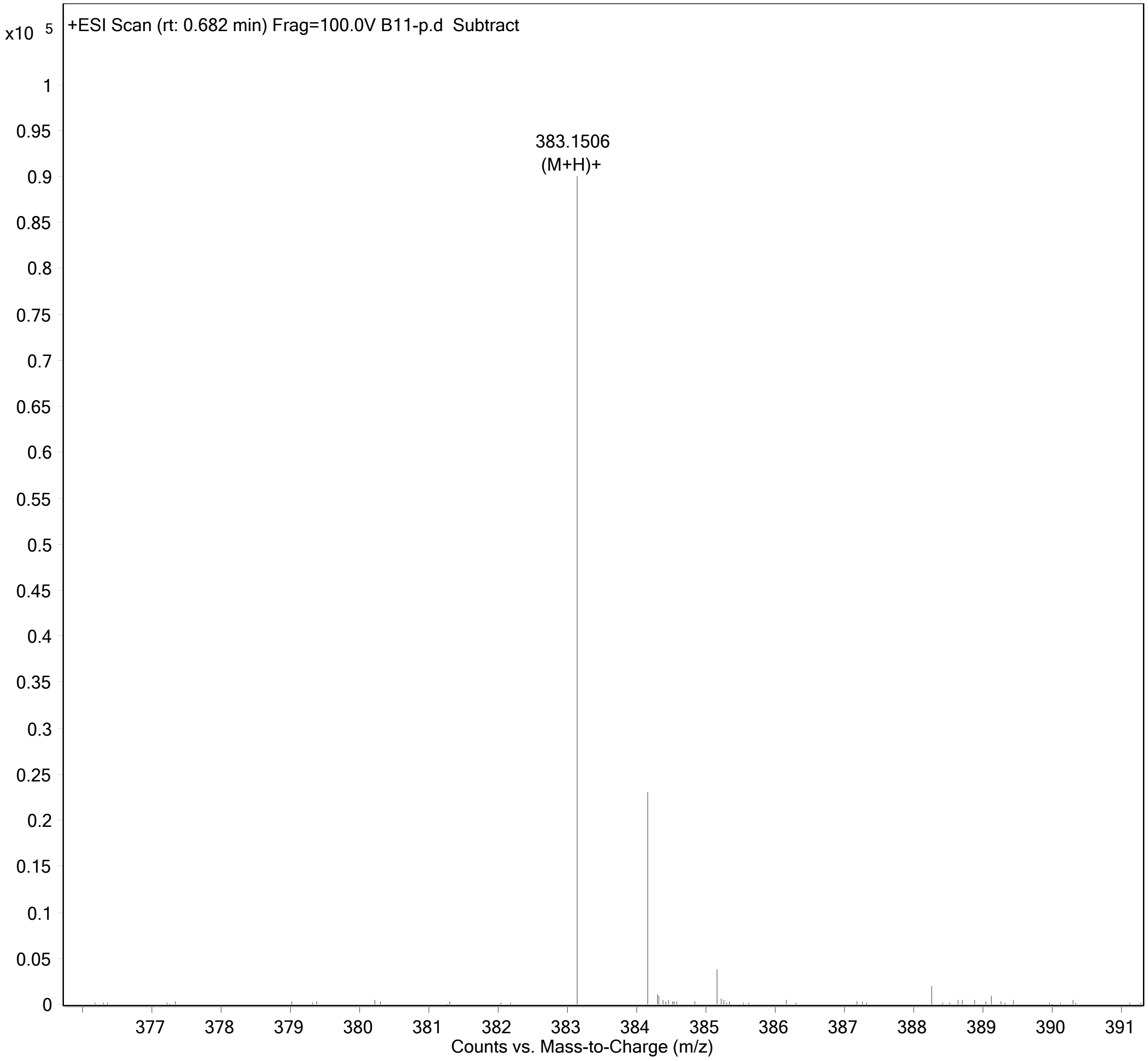

B12

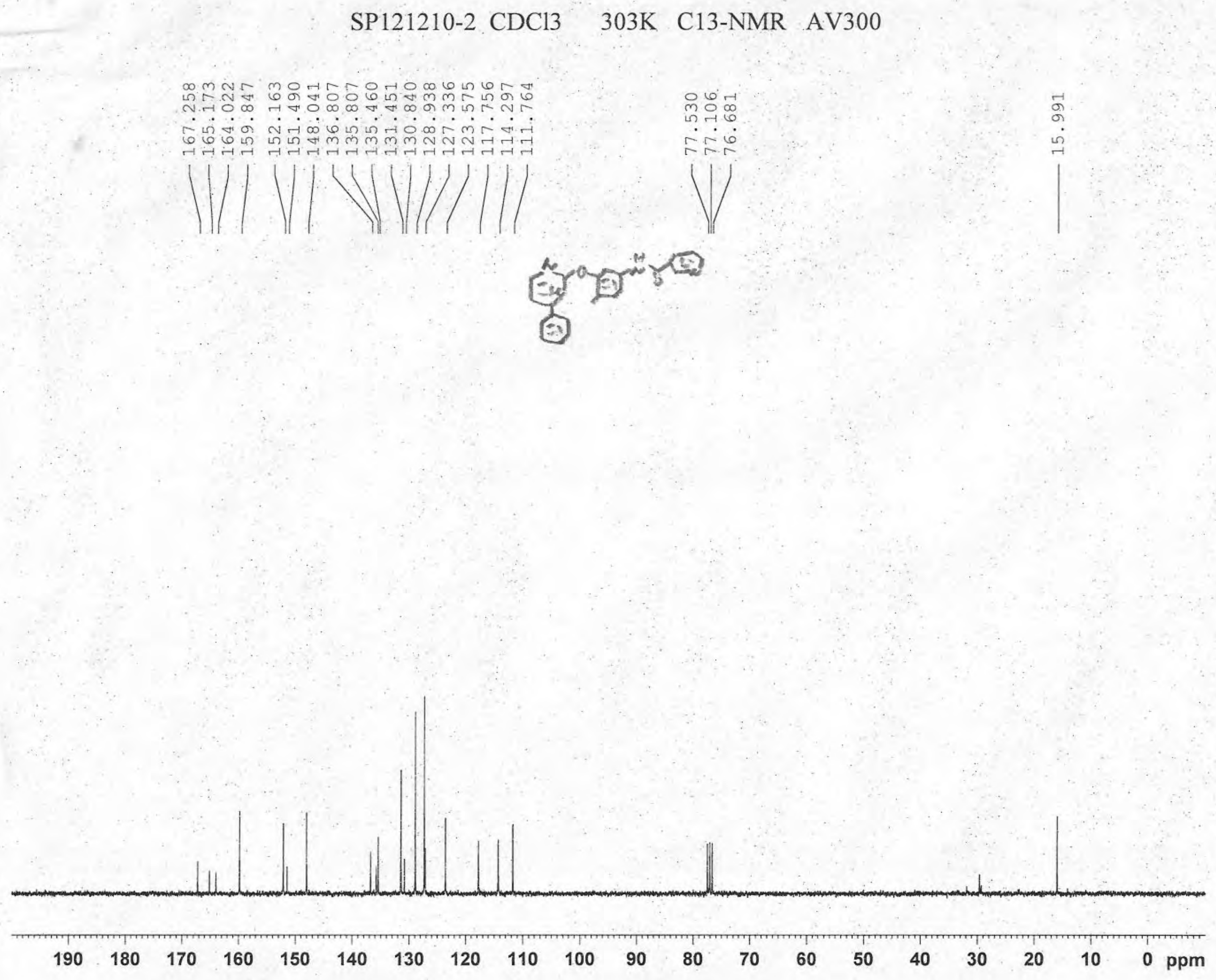

SP121210-2 CDCl3 303K C13-NMR AV300
167.258
165.173
164.022
159.847
152.163
151.490
148.041
136.807
135.807
135.460
131.451
130.840
128.938
127.336
123.575
117.756
114.297
111.764
77.530
77.106
76.681
15.991
190 180 170 160 150 140 130 120 110 100 90 80 70 60 50 40 30 20 10 0 ppm

B12

SP121210-2 1H-NMR CDCl3 303K AV-300

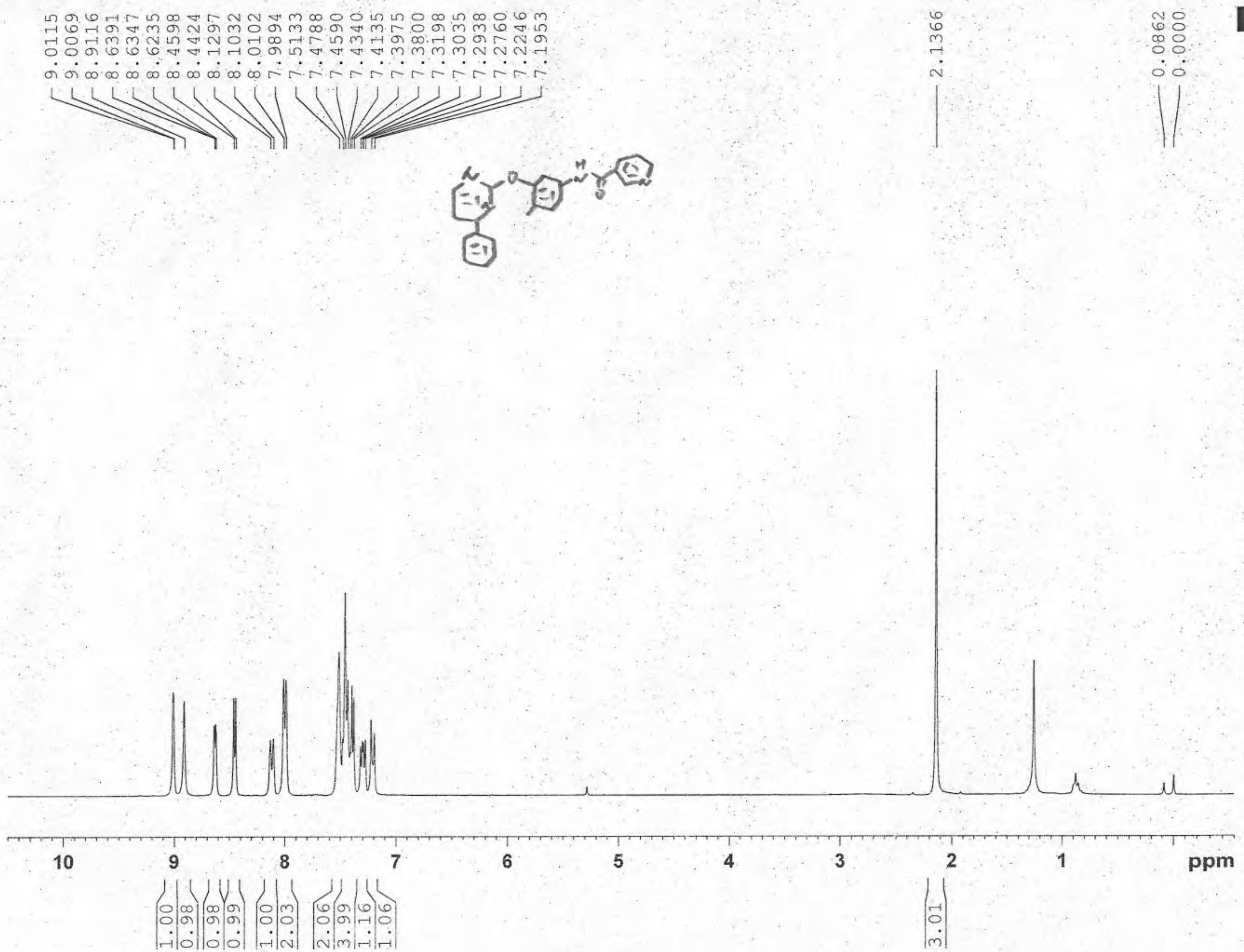

**Sample Name**		**Position**	p1A2	**Instrument Name**	Instrument 1
**User Name**	QTOF-PC\QTOF	**Inj Vol**	0.1	**InjPosition**	
**Sample Type**	Sample	**IRM Calibration Status**	Success	**Data Filename**	B12-p.d
**ACQ Method**	20110418-MSonly-p.m	**Comment**		**Acquired Time**	3/12/2018 3:55:07 PM

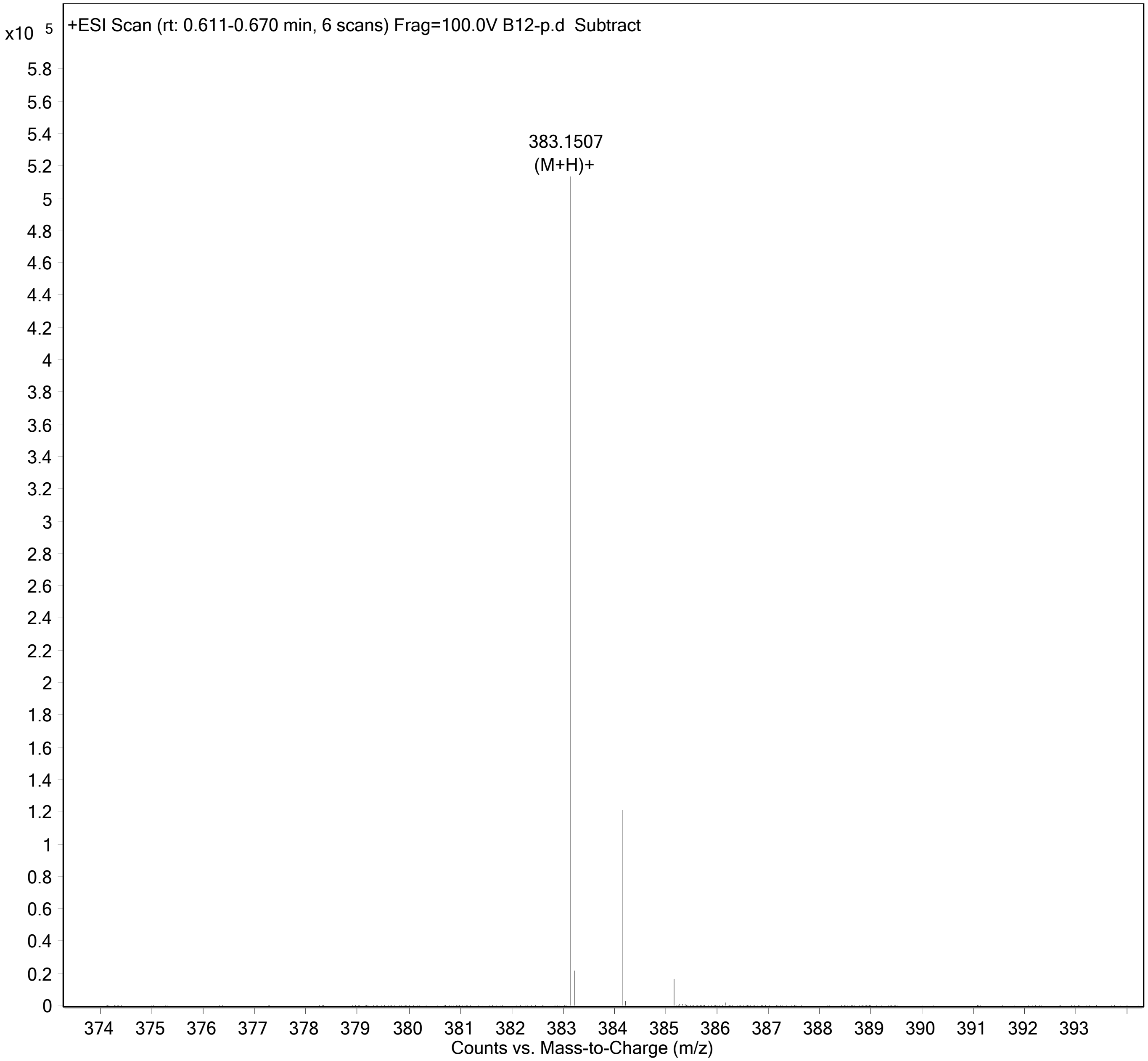

C1

SP130325-2-C C13-NMR CDCL3 303K AV-300

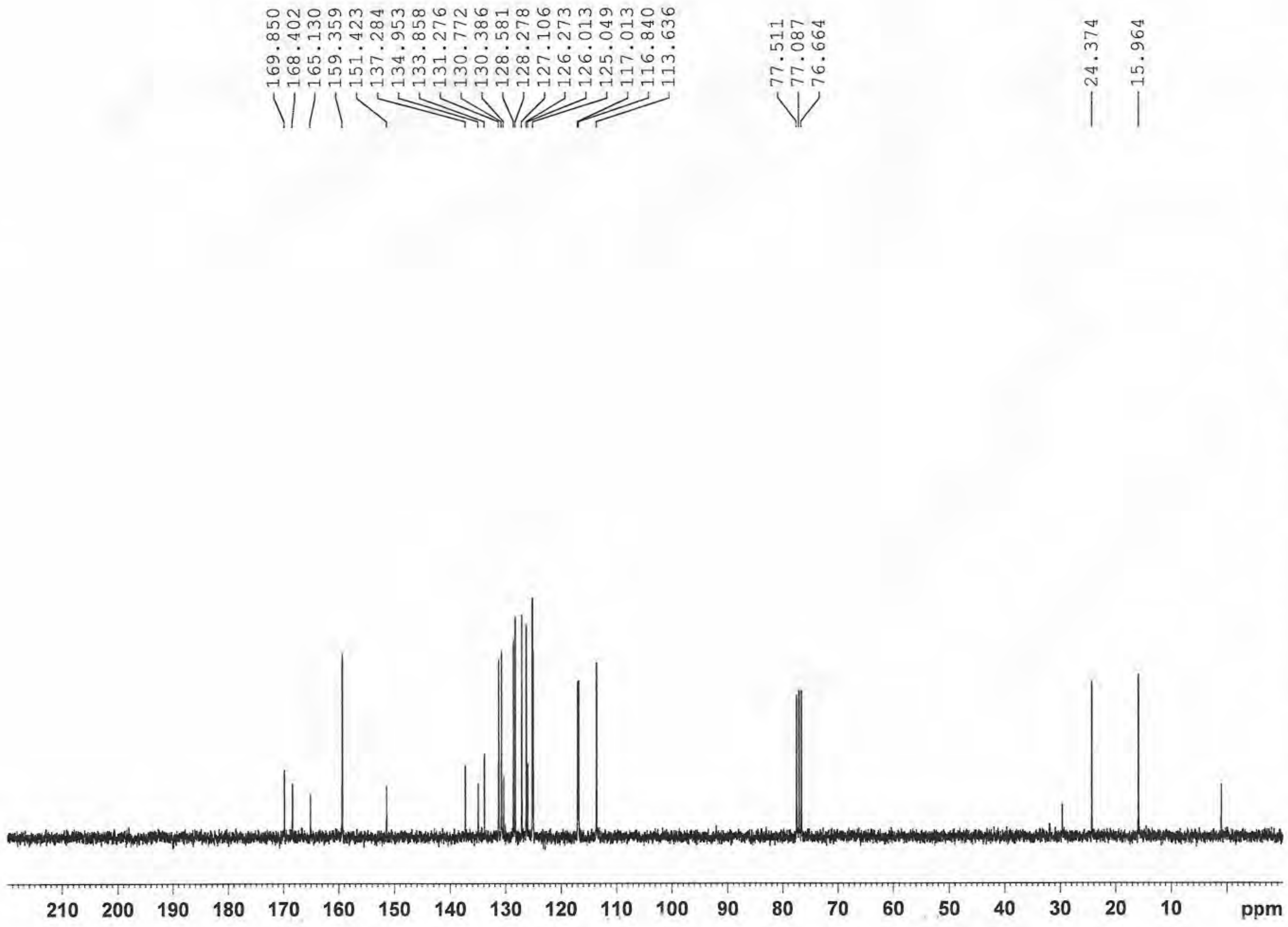

C1

SP2013-03-22-2 CDCL3 1HNMR AV300

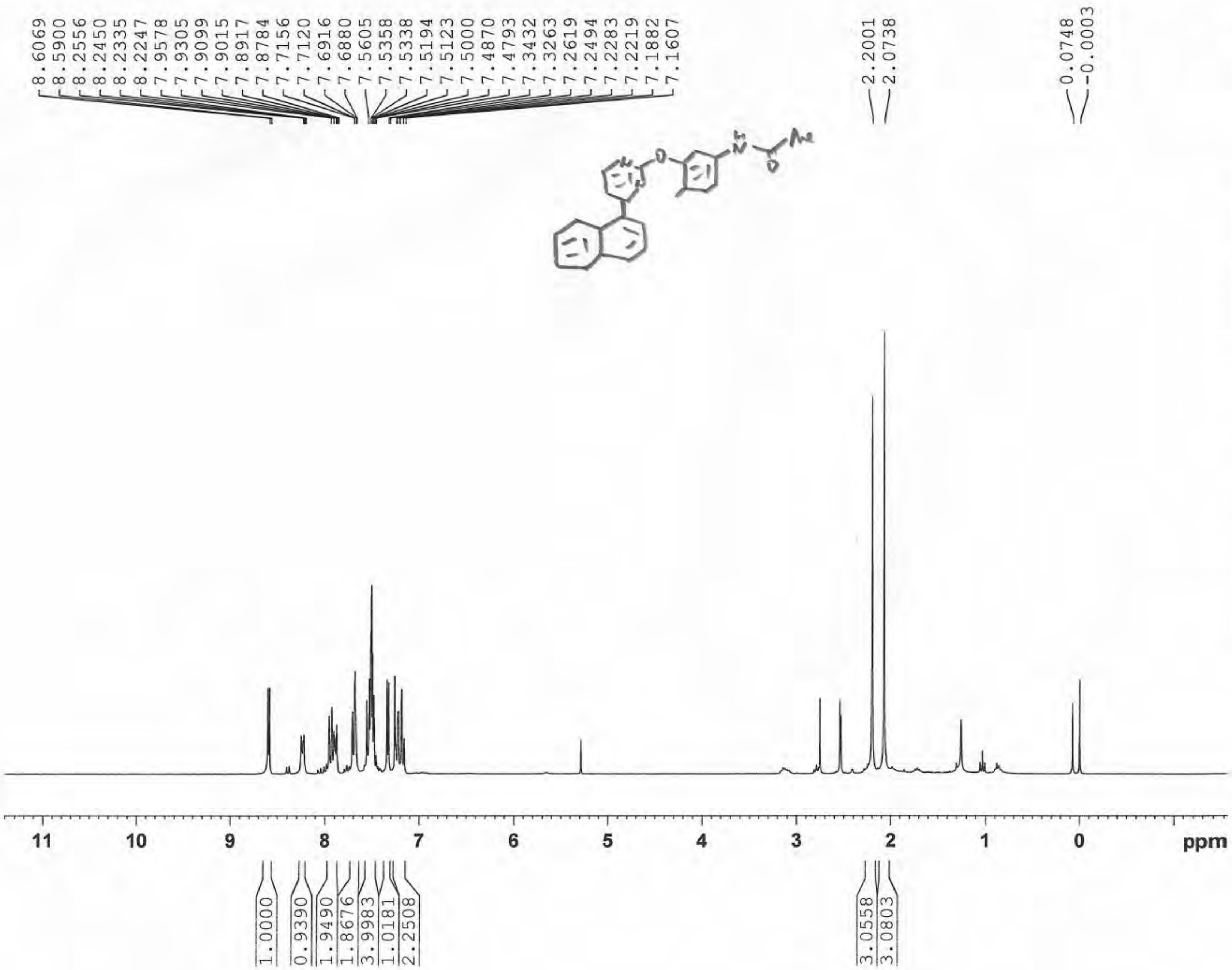

**Sample Name**		**Position**	p1A3	**Instrument Name**	Instrument 1
**User Name**	QTOF-PC\QTOF	**Inj Vol**	0.1	**InjPosition**	
**Sample Type**	Sample	**IRM Calibration Status**	Success	**Data Filename**	C1-p.d
**ACQ Method**	20110418-MSonly-p.m	**Comment**		**Acquired Time**	3/12/2018 3:57:44 PM

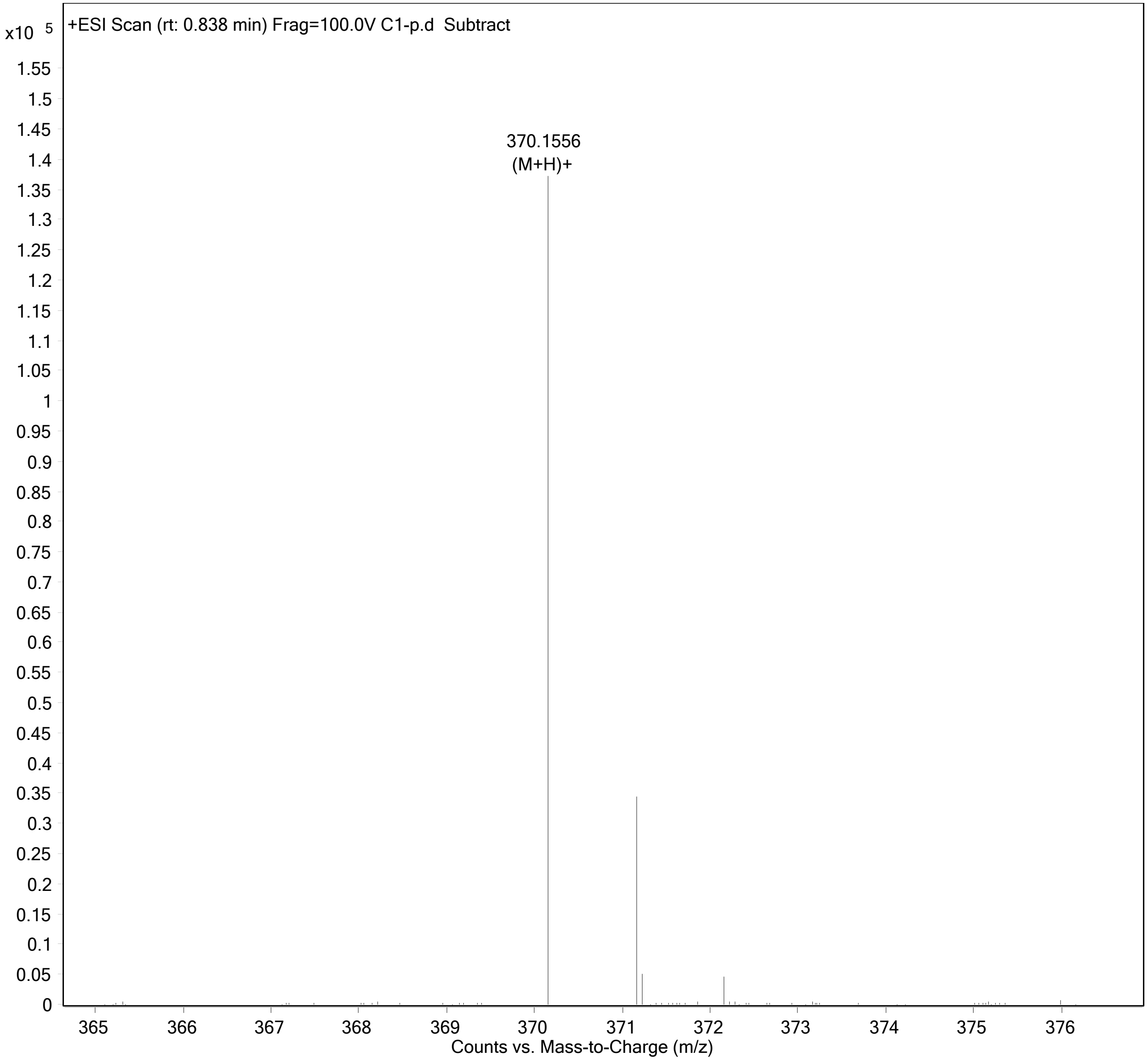

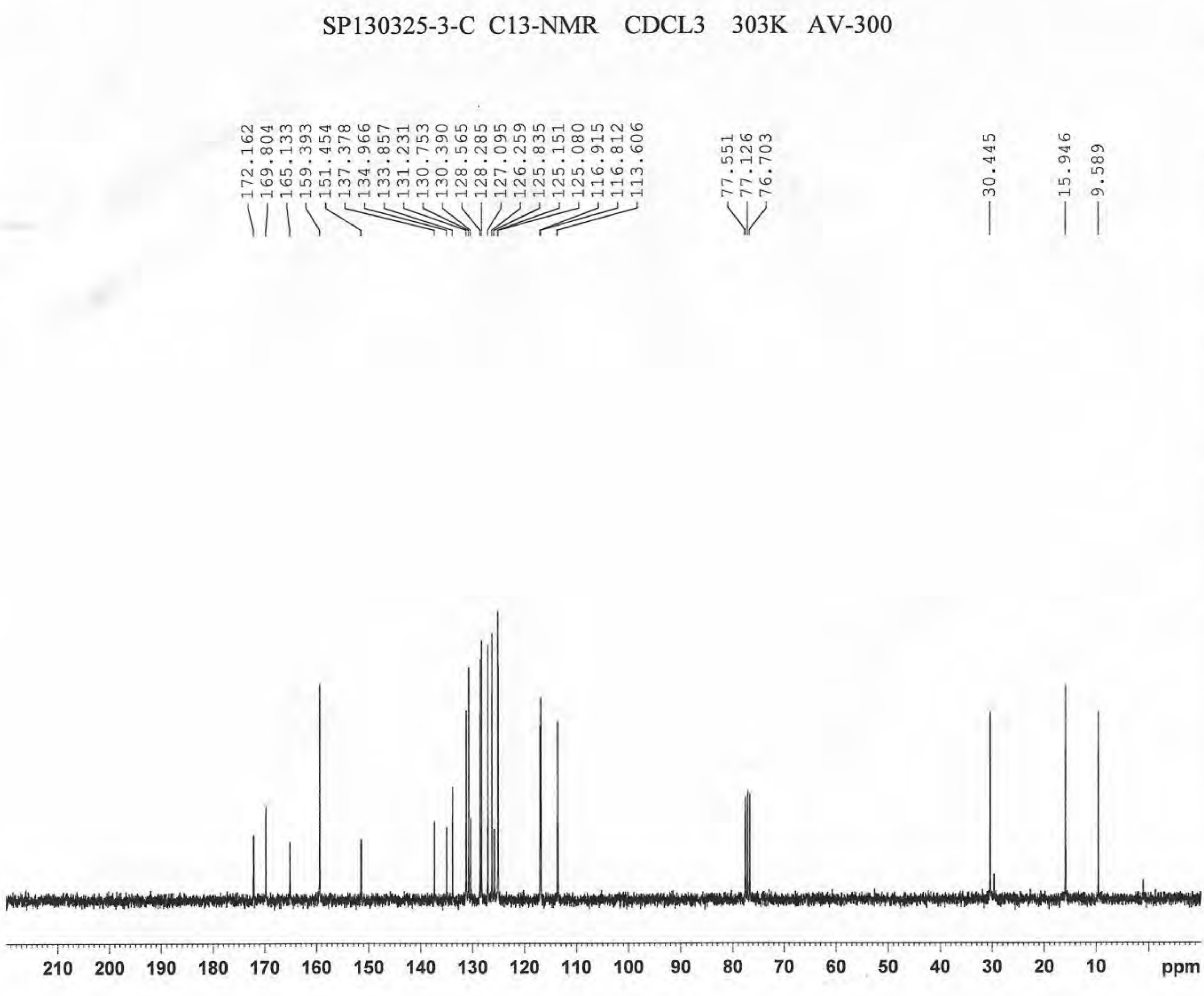
SP130325-3-C C13-NMR CDCL3 303K AV-300
172.162
169.804
165.133
159.393
151.454
137.378
134.966
133.857
131.231
130.753
130.390
128.565
128.285
127.095
126.259
125.835
125.151
125.080
116.915
116.812
113.606
77.551
77.126
76.703
30.445
15.946
9.589
ppm

C2

SP2013-03-22-3 CDCL3 1HNMR AV300

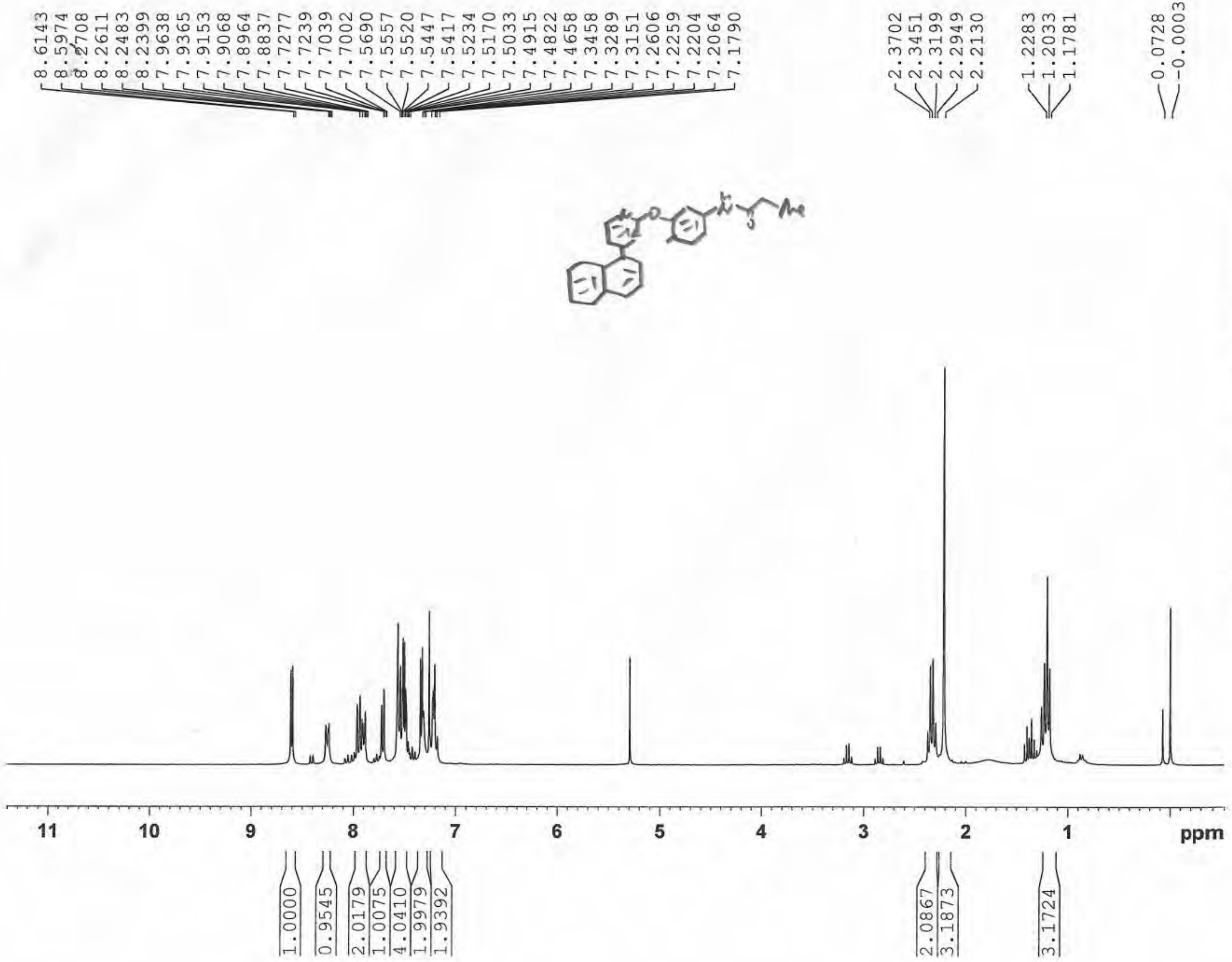

**Sample Name**		**Position**	p1A4	**Instrument Name**	Instrument 1
**User Name**	QTOF-PC\QTOF	**Inj Vol**	0.1	**InjPosition**	
**Sample Type**	Sample	**IRM Calibration Status**	Success	**Data Filename**	C2-p.d
**ACQ Method**	20110418-MSonly-p.m	**Comment**		**Acquired Time**	3/12/2018 3:59:34 PM

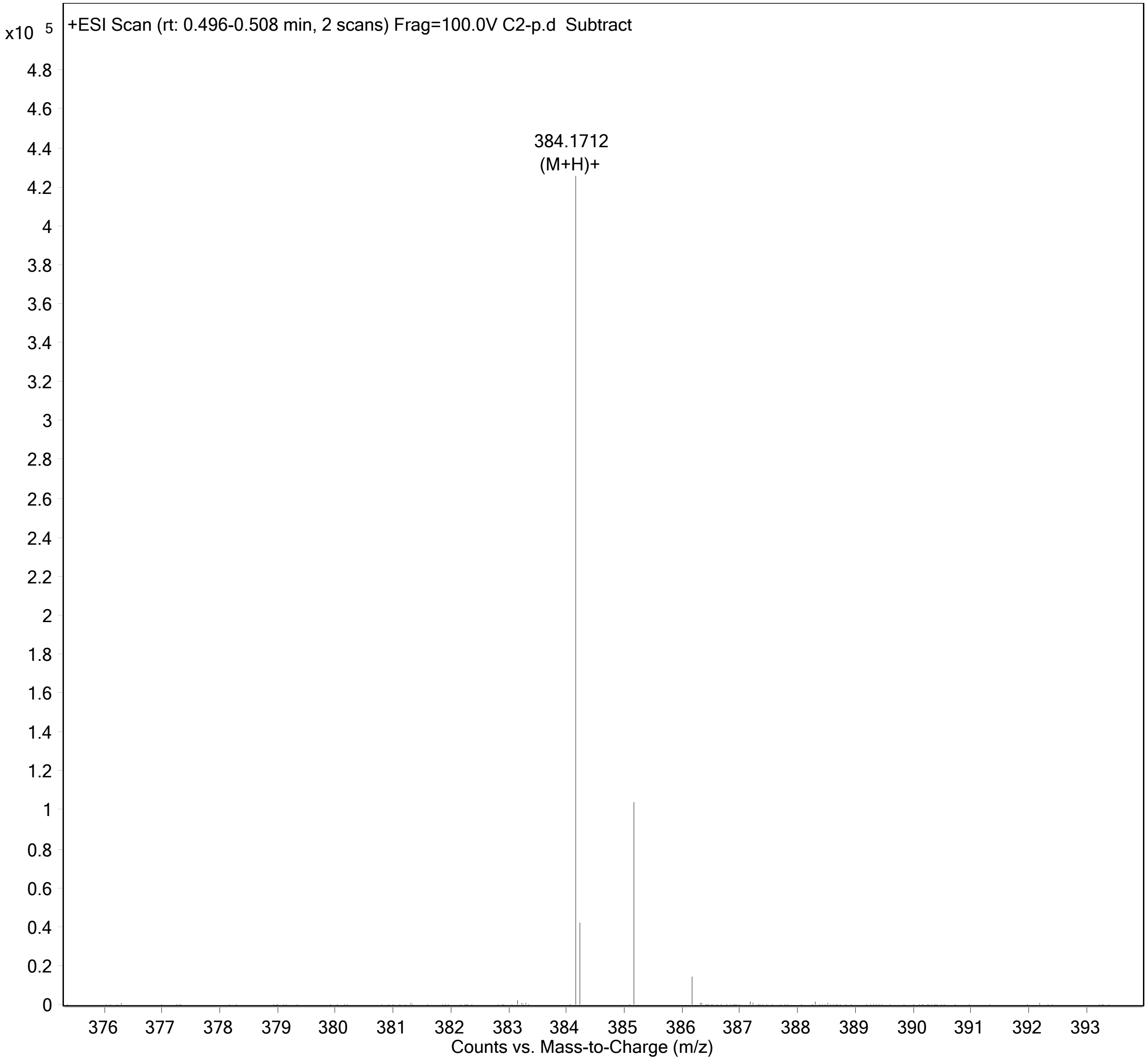

C3

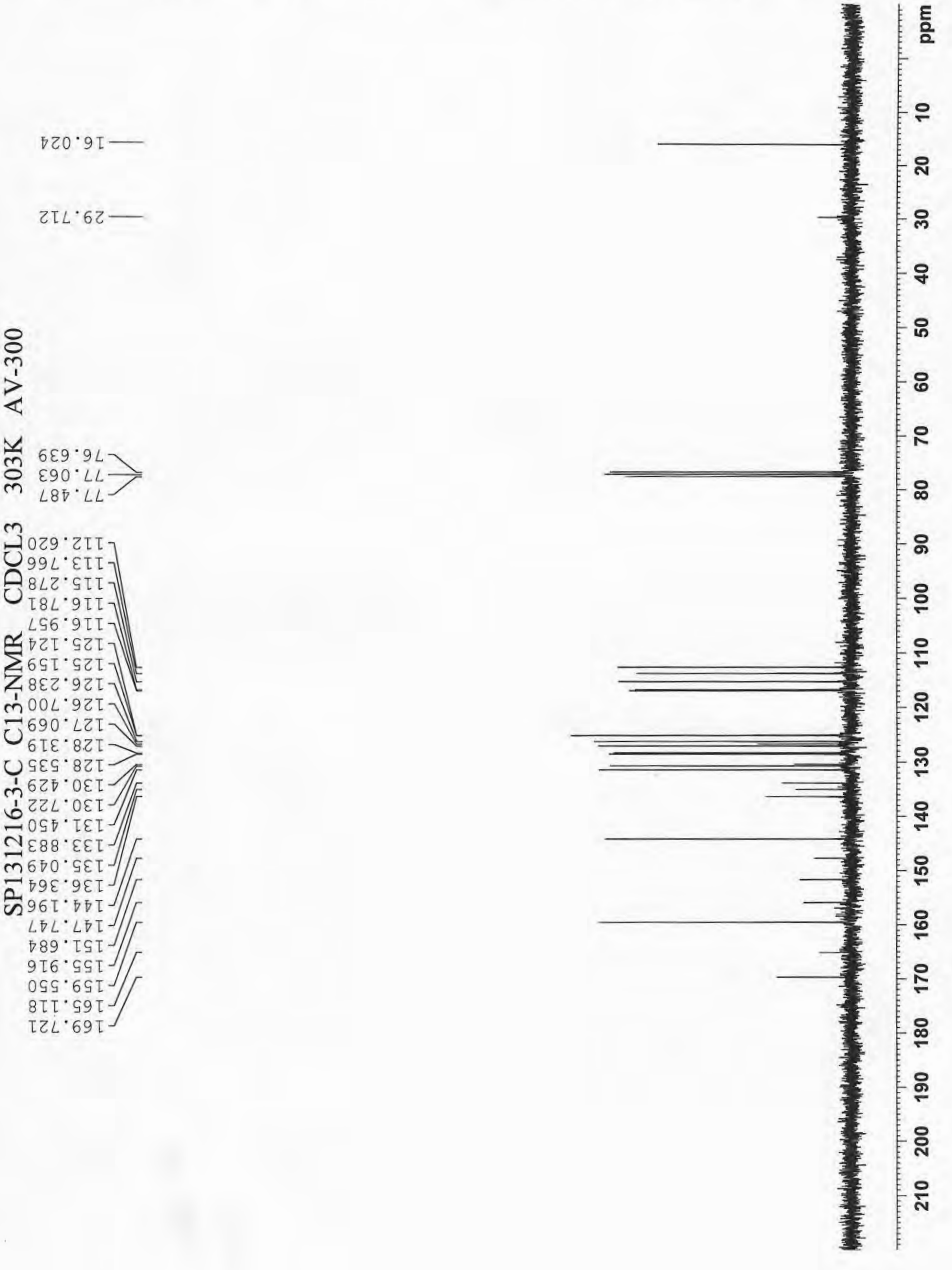
SP131216-3-C C13-NMR CDCL3 303K AV-300
16.024
29.712
76.639
77.063
77.487
112.620
113.766
115.278
116.781
116.957
125.124
125.159
126.238
126.700
127.069
128.319
128.535
130.429
130.722
131.450
133.883
135.049
136.364
144.196
147.747
151.684
155.916
159.550
165.118
169.721
ppm
210
200
190
180
170
160
150
140
130
120
110
100
90
80
70
60
50
40
30
20
10

SP131216-3 CDCL3 1HNMR AV300

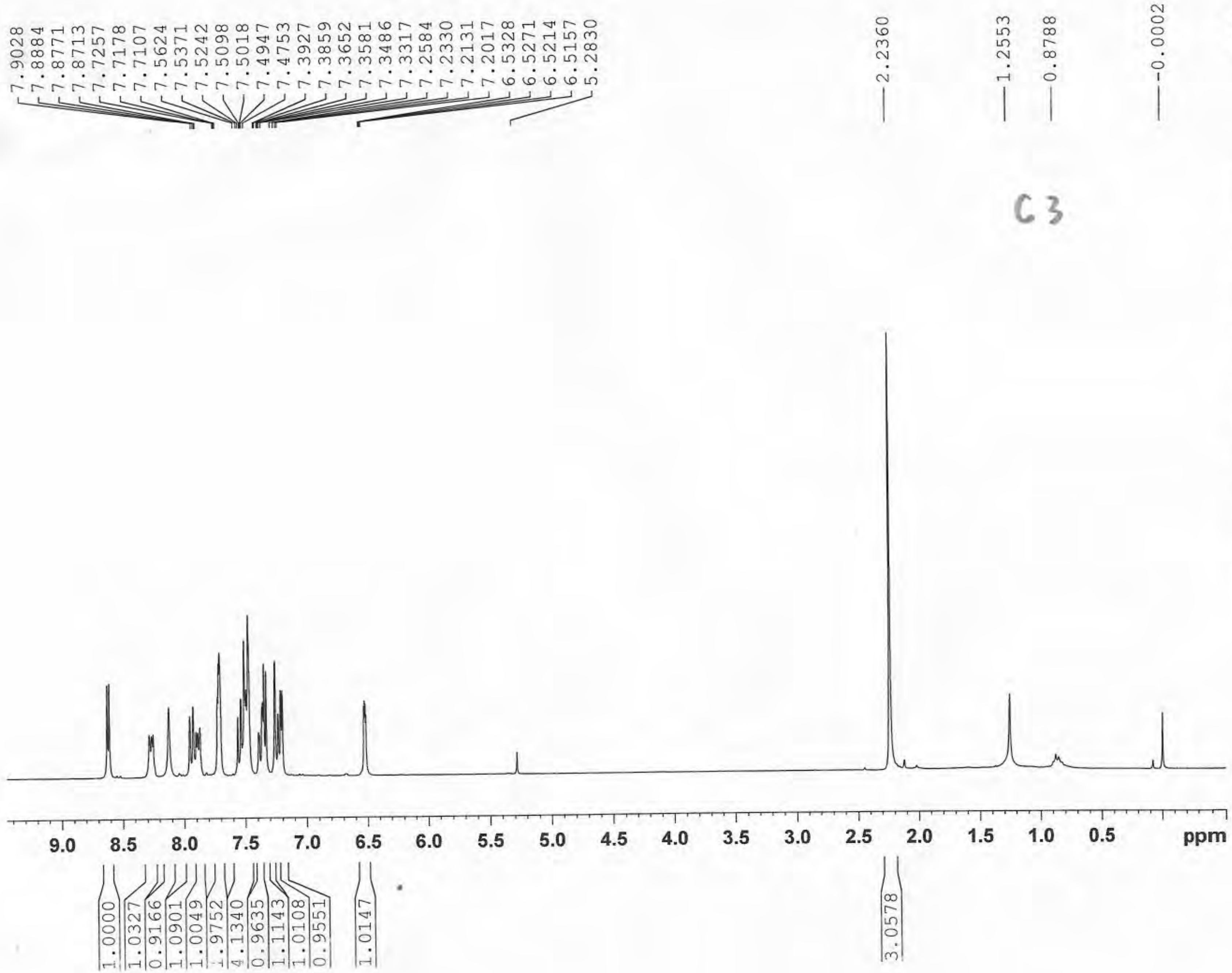

**Sample Name**		**Position**	p1A5	**Instrument Name**	Instrument 1
**User Name**	QTOF-PC\QTOF	**Inj Vol**	0.1	**InjPosition**	
**Sample Type**	Sample	**IRM Calibration Status**	Success	**Data Filename**	C3-p.d
**ACQ Method**	20110418-MSonly-p.m	**Comment**		**Acquired Time**	3/12/2018 4:01:18 PM

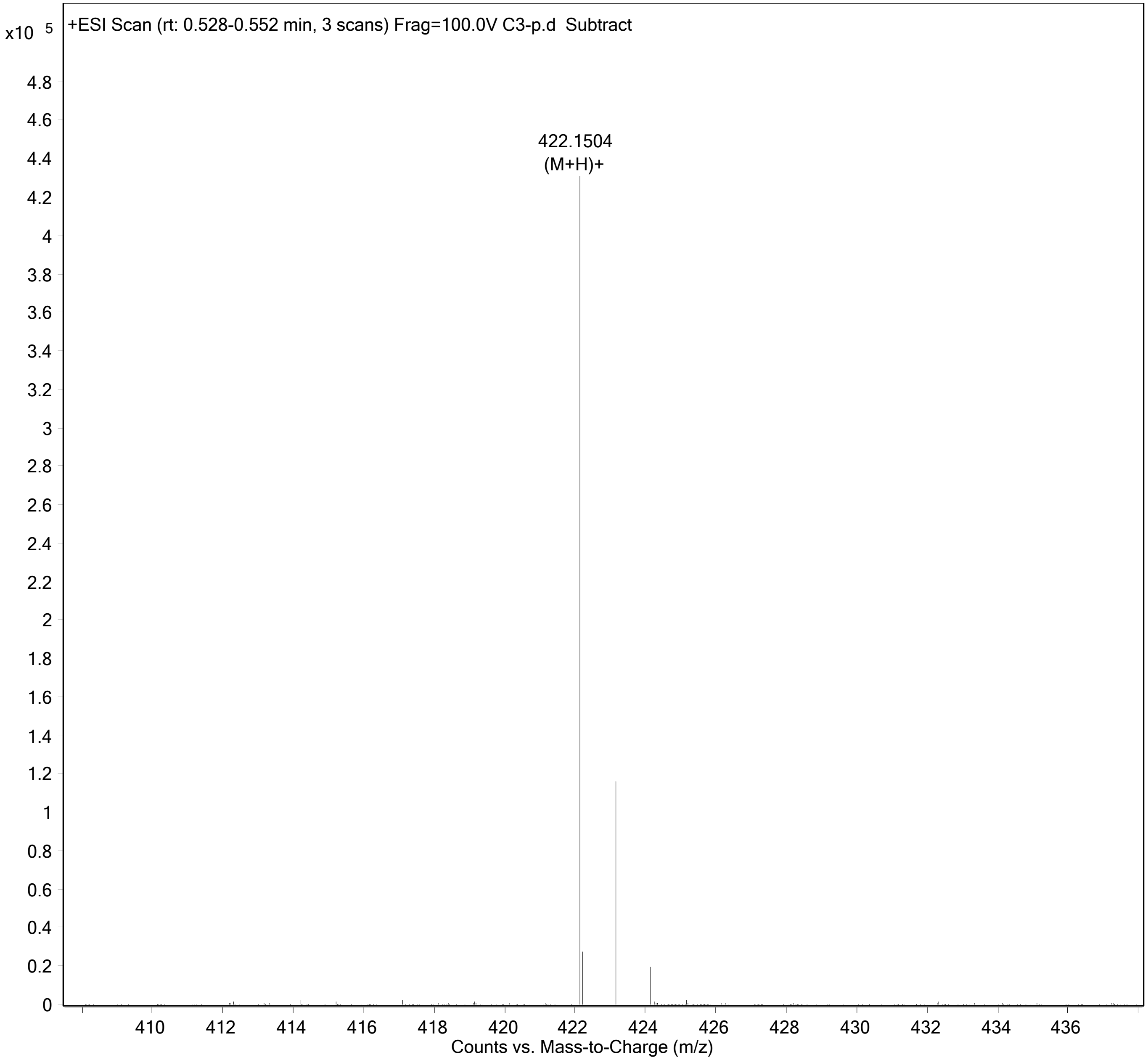

C4

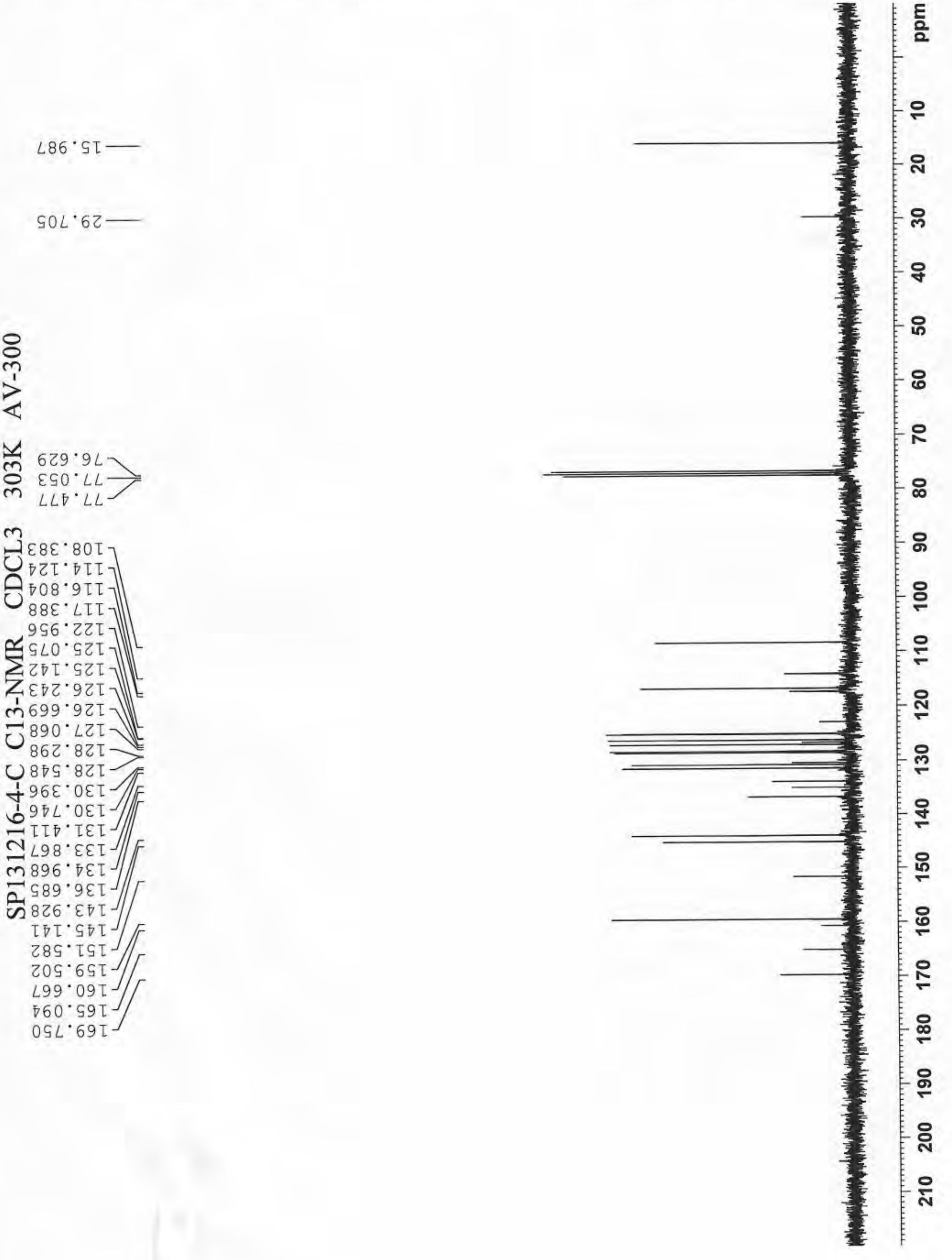
SP131216-4-C C13-NMR CDCL3 303K AV-300
15.987
29.705
76.629
77.053
77.477
108.383
114.124
116.804
117.388
122.956
125.075
125.142
126.243
126.669
127.068
128.298
128.548
130.396
130.746
131.411
133.867
134.968
136.685
143.928
145.141
151.582
159.502
160.667
165.094
169.750
ppm
10
20
30
40
50
60
70
80
90
100
110
120
130
140
150
160
170
180
190
200
210

C4

SP131216-4 CDCL3 1HNMR AV300

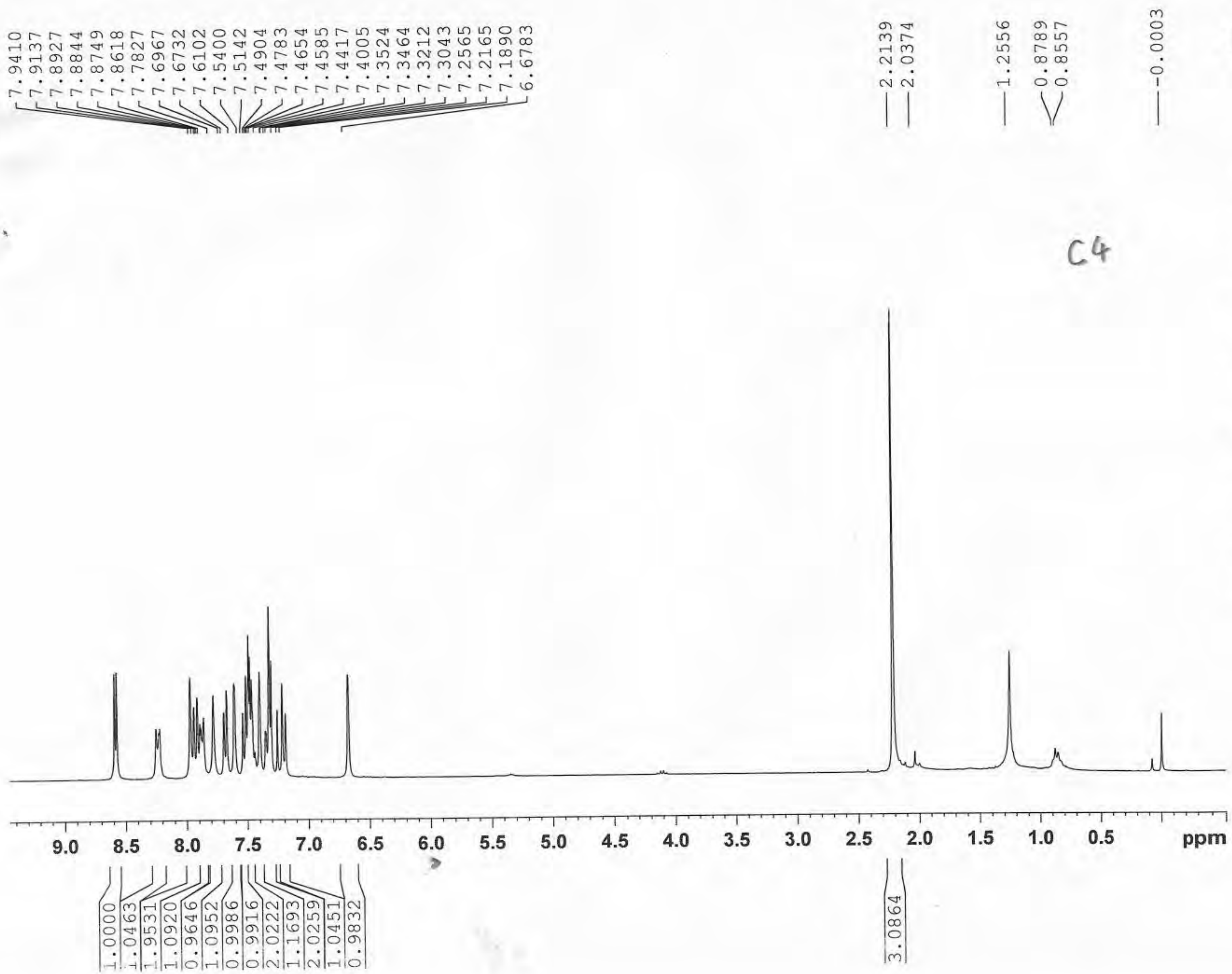

**Sample Name**		**Position**	p1A6	**Instrument Name**	Instrument 1
**User Name**	QTOF-PC\QTOF	**Inj Vol**	0.1	**InjPosition**	
**Sample Type**	Sample	**IRM Calibration Status**	Success	**Data Filename**	C4-p.d
**ACQ Method**	20110418-MSonly-p.m	**Comment**		**Acquired Time**	3/12/2018 4:03:38 PM

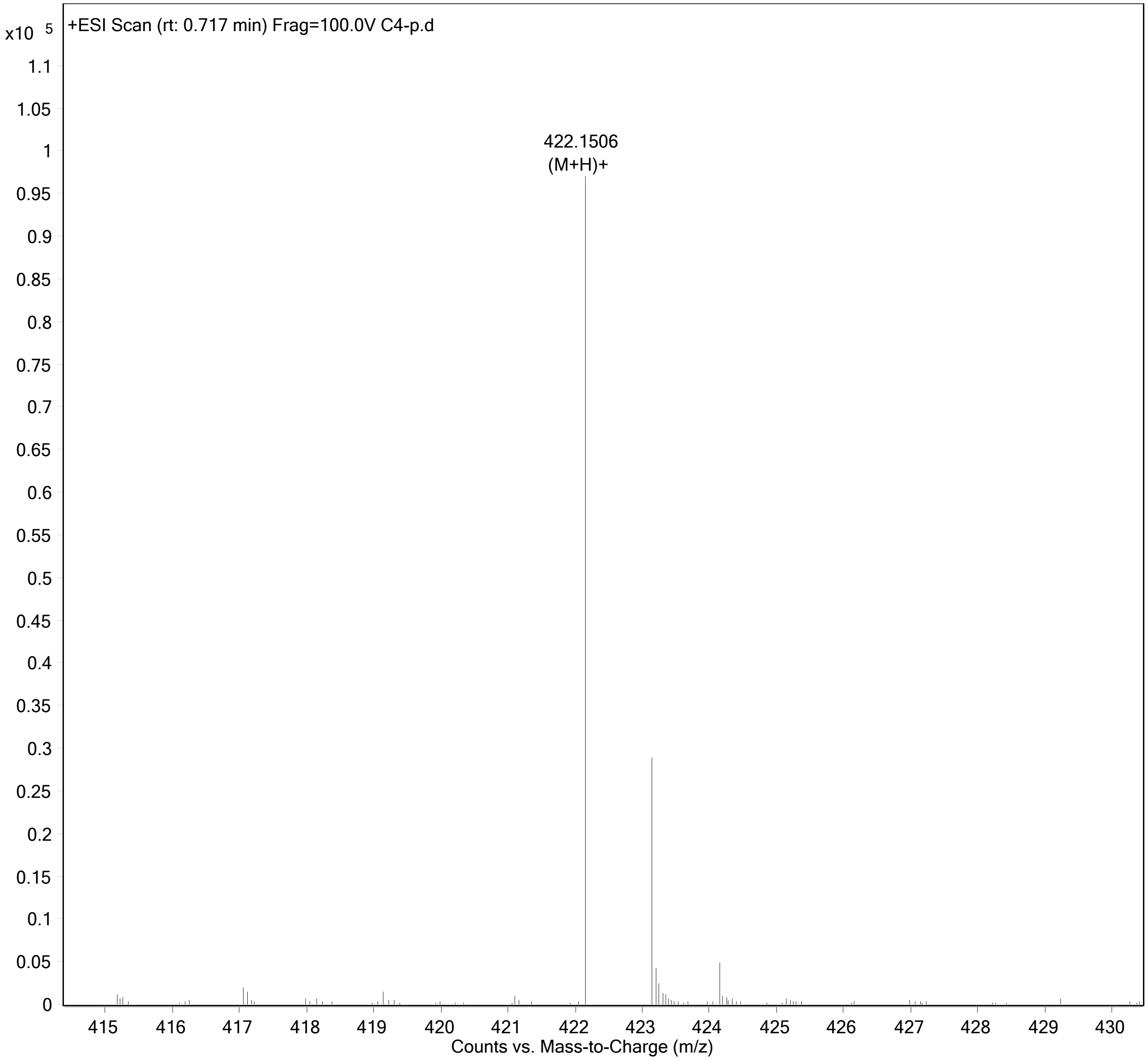

C5

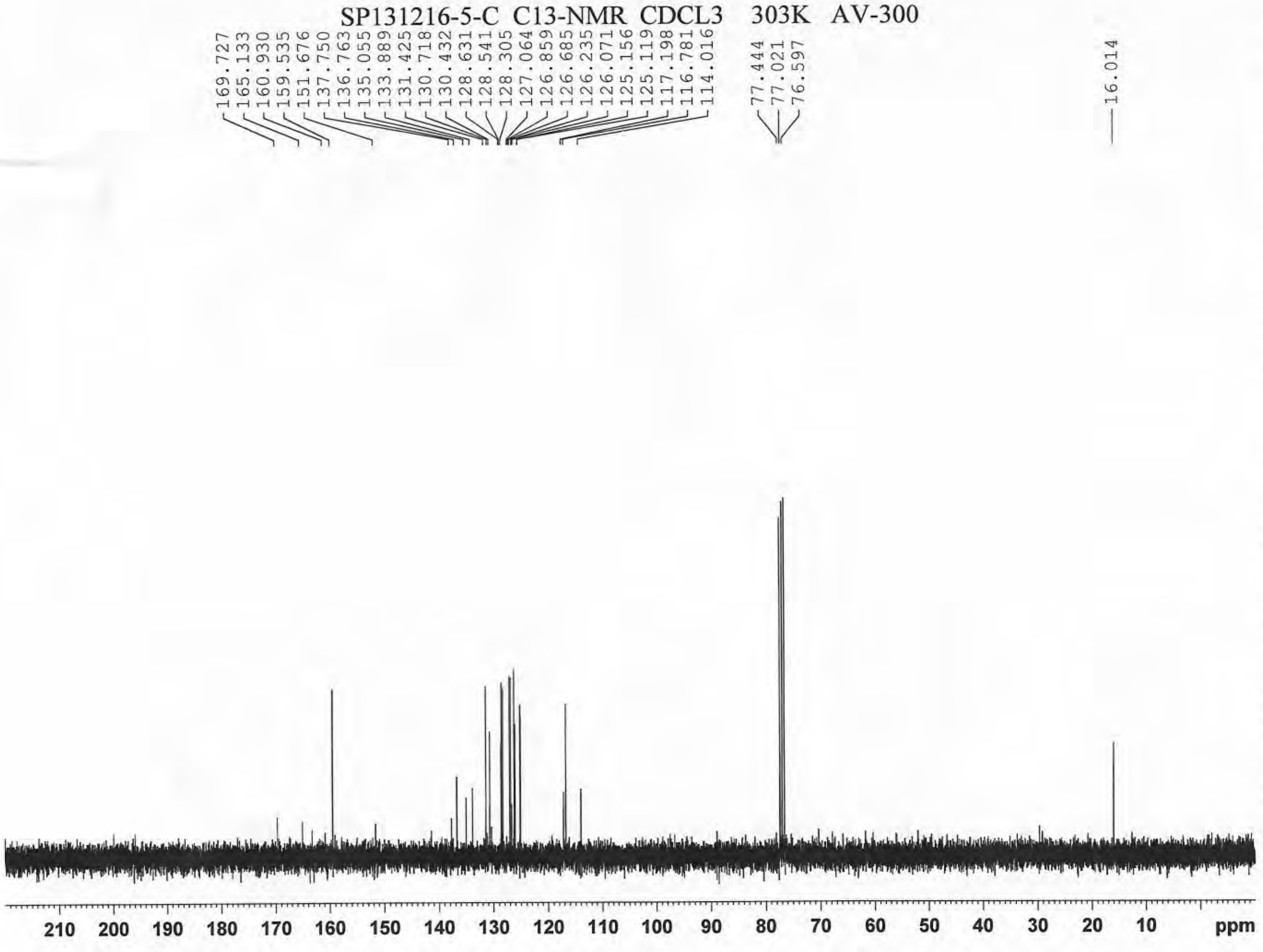
SP131216-5-C C13-NMR CDCL3 303K AV-300
169.727
165.133
160.930
159.535
151.676
137.750
136.763
135.055
133.889
131.425
130.718
130.432
128.631
128.541
128.305
127.064
126.859
126.685
126.235
126.071
125.156
125.119
117.198
116.781
114.016
77.444
77.021
76.597
16.014
210 200 190 180 170 160 150 140 130 120 110 100 90 80 70 60 50 40 30 20 10 ppm

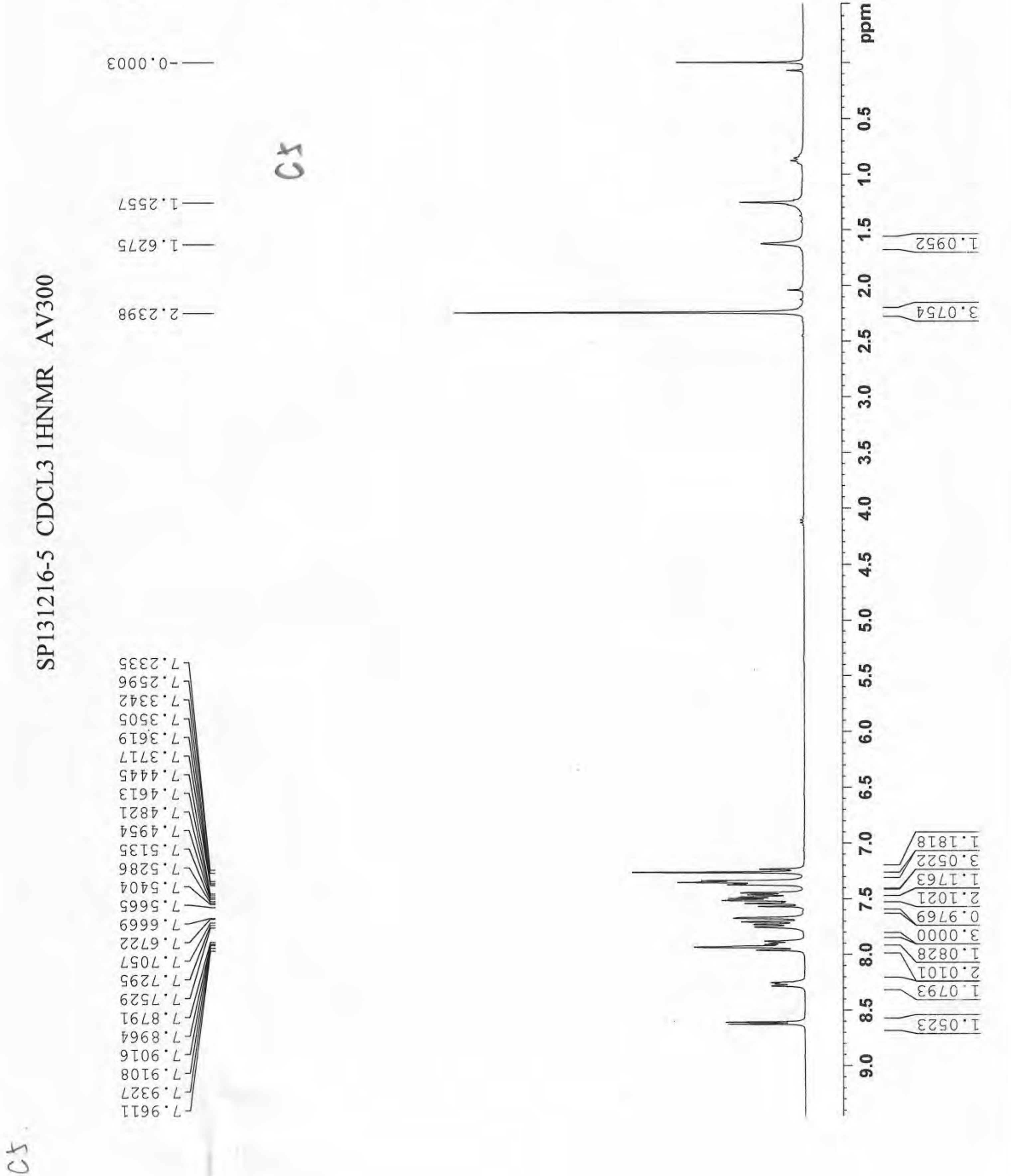
SP131216-5 CDCL3 1HNMR AV300
C5
-0.0003
1.2557
1.6275
2.2398
7.2335
7.2596
7.3342
7.3505
7.3619
7.3717
7.4445
7.4613
7.4821
7.4954
7.5135
7.5286
7.5404
7.5665
7.6669
7.6722
7.7057
7.7295
7.7529
7.8791
7.8964
7.9016
7.9108
7.9327
7.9611
1.0952
3.0754
1.1818
3.0522
1.1763
2.1021
0.9769
3.0000
1.0828
2.0101
1.0793
1.0523
ppm
0.5
1.0
1.5
2.0
2.5
3.0
3.5
4.0
4.5
5.0
5.5
6.0
6.5
7.0
7.5
8.0
8.5
9.0

**Sample Name**		**Position**	p1A7	**Instrument Name**	Instrument 1
**User Name**	QTOF-PC\QTOF	**Inj Vol**	0.1	**InjPosition**	
**Sample Type**	Sample	**IRM Calibration Status**	Success	**Data Filename**	C5-p.d
**ACQ Method**	20110418-MSonly-p.m	**Comment**		**Acquired Time**	3/12/2018 4:05:27 PM

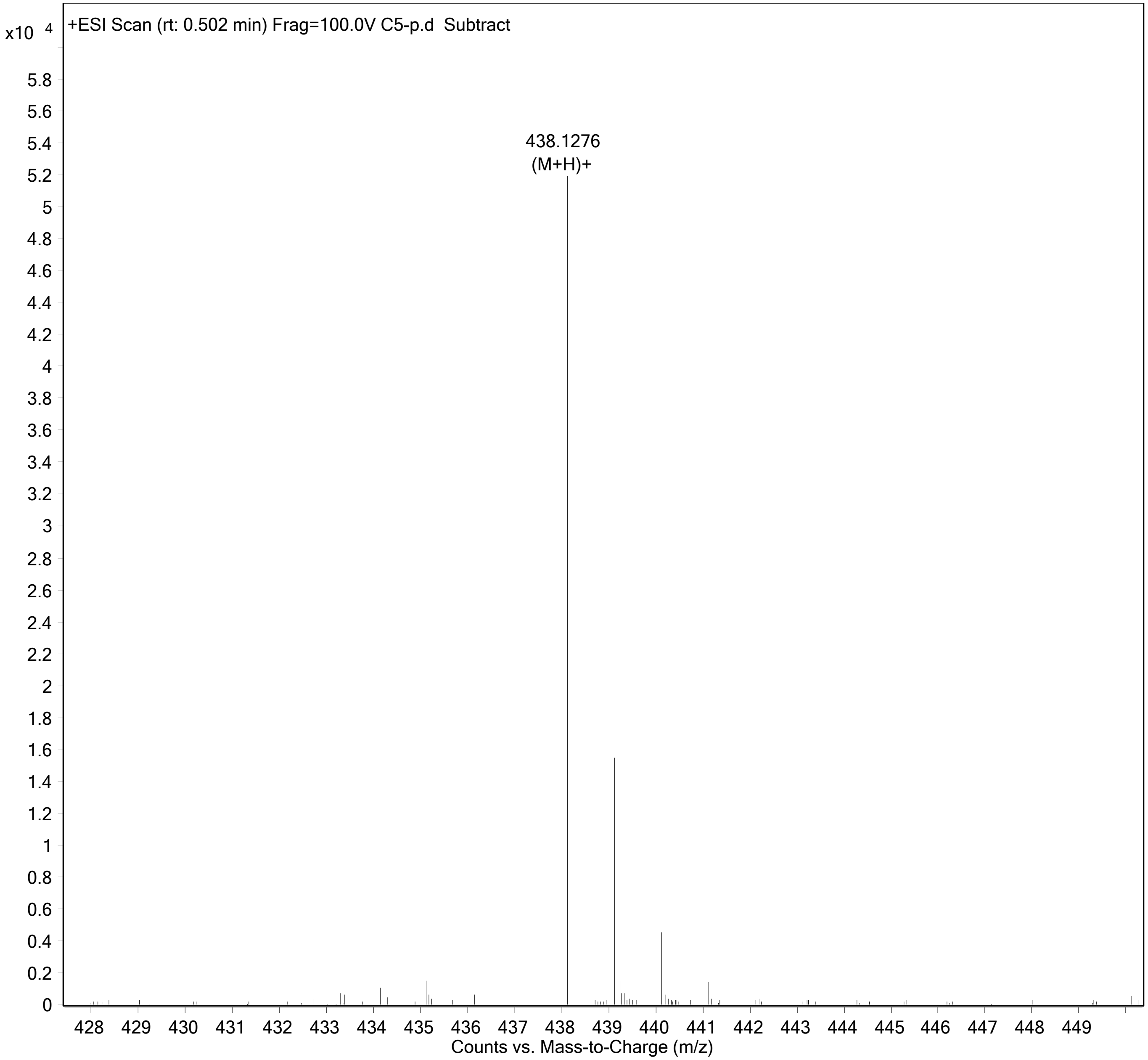

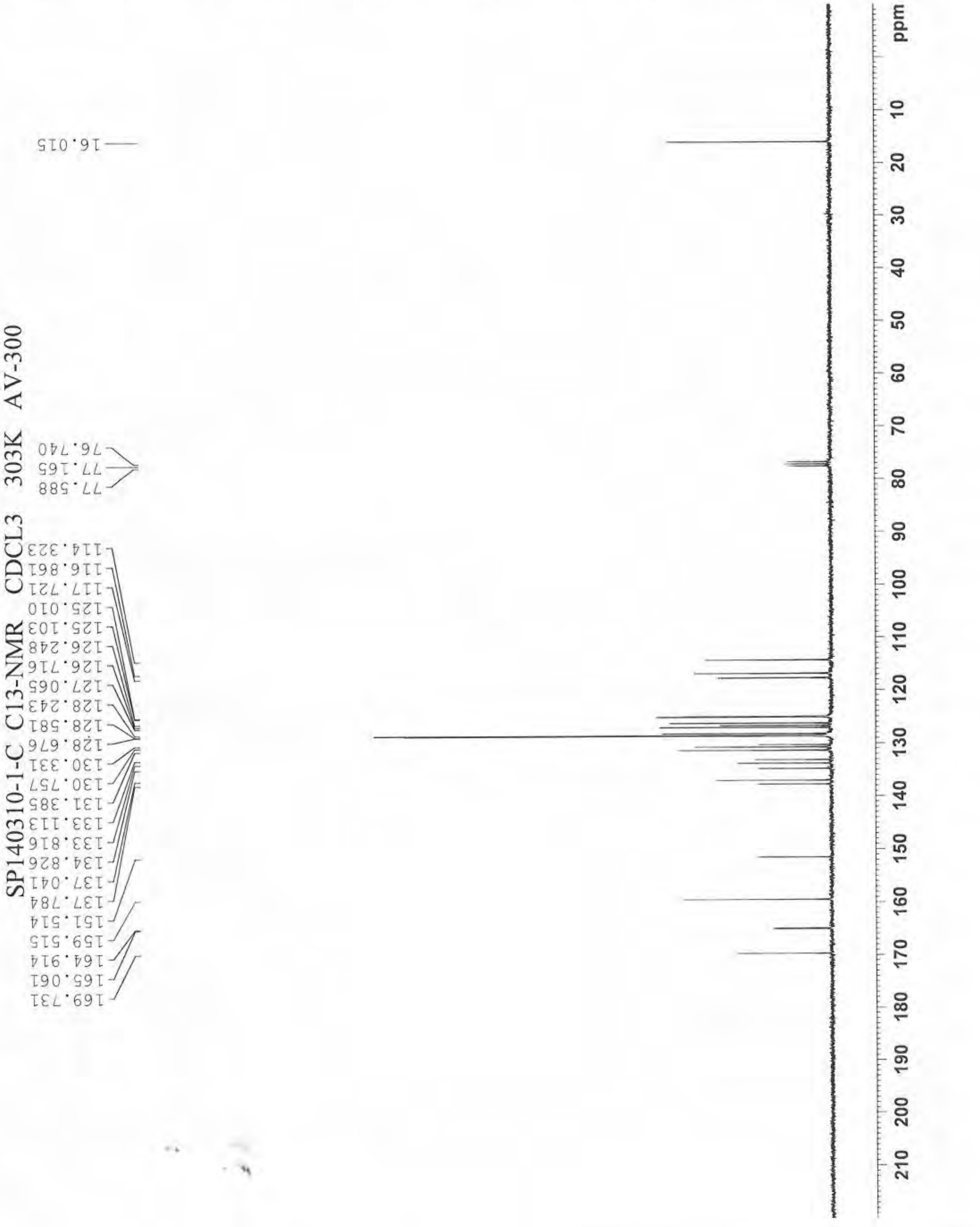
SP140310-1-C C13-NMR CDCL3 303K AV-300
16.015
76.740
77.165
77.588
114.323
116.861
117.721
125.010
125.103
126.248
126.716
127.065
128.243
128.581
128.676
130.331
130.757
131.385
133.113
133.816
134.826
137.041
137.784
151.514
159.515
164.914
165.061
169.731
ppm

C6

SP140310-1 CDCL3 1HNMR AV300

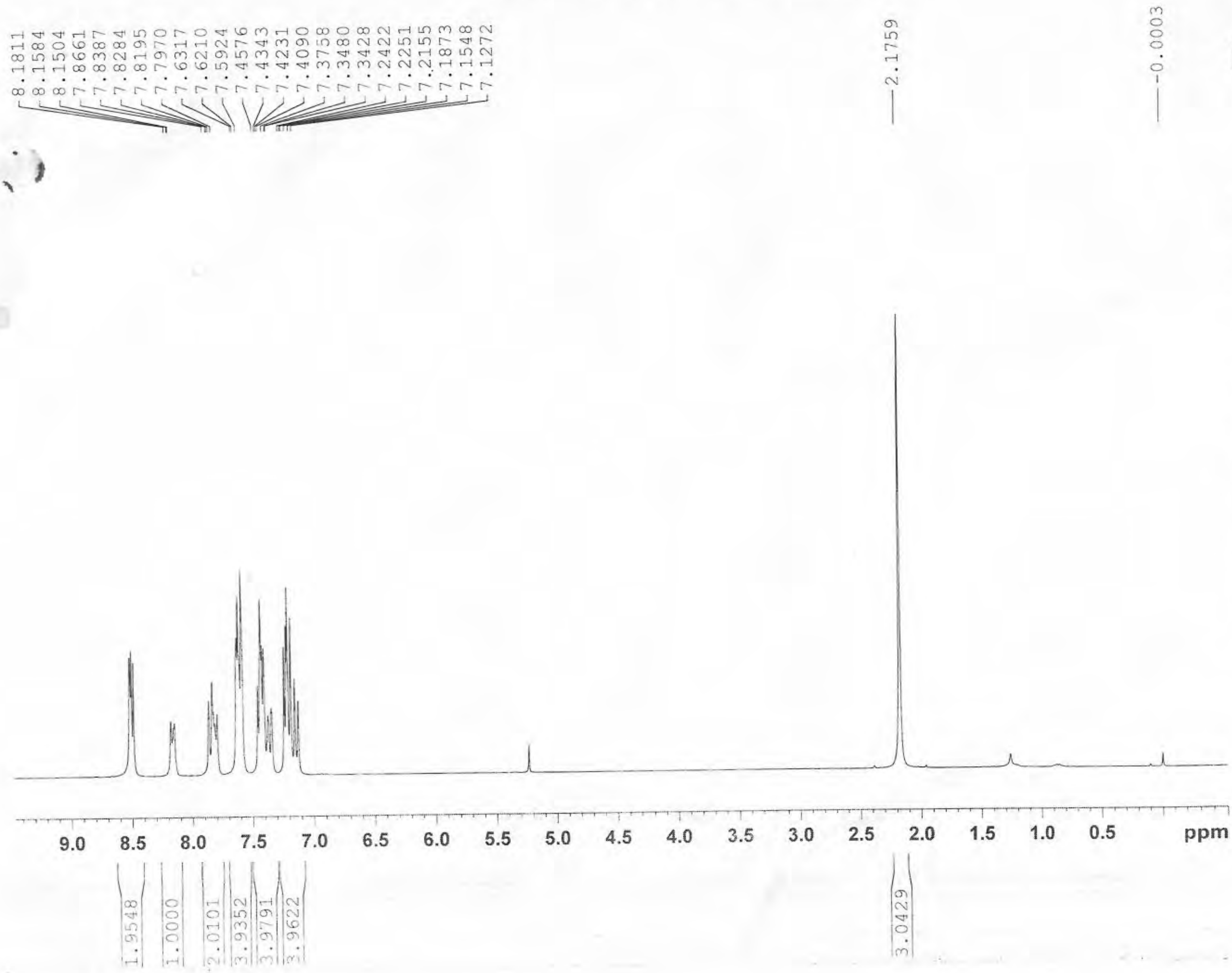

**Sample Name**		**Position**	p1A8	**Instrument Name**	Instrument 1
**User Name**	QTOF-PC\QTOF	**Inj Vol**	0.1	**InjPosition**	
**Sample Type**	Sample	**IRM Calibration Status**	Success	**Data Filename**	C6-p.d
**ACQ Method**	20110418-MSonly-p.m	**Comment**		**Acquired Time**	3/12/2018 4:11:03 PM

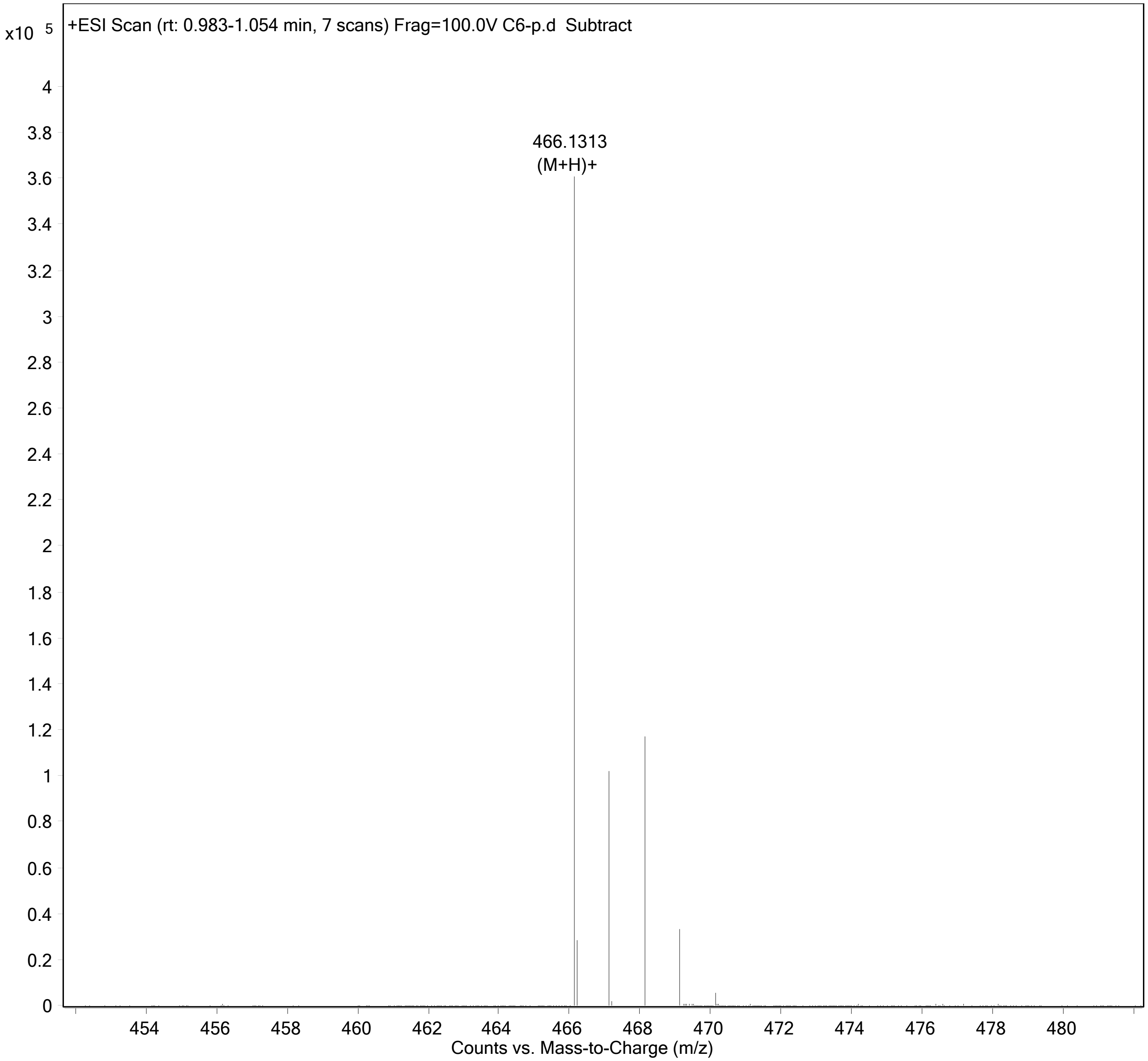

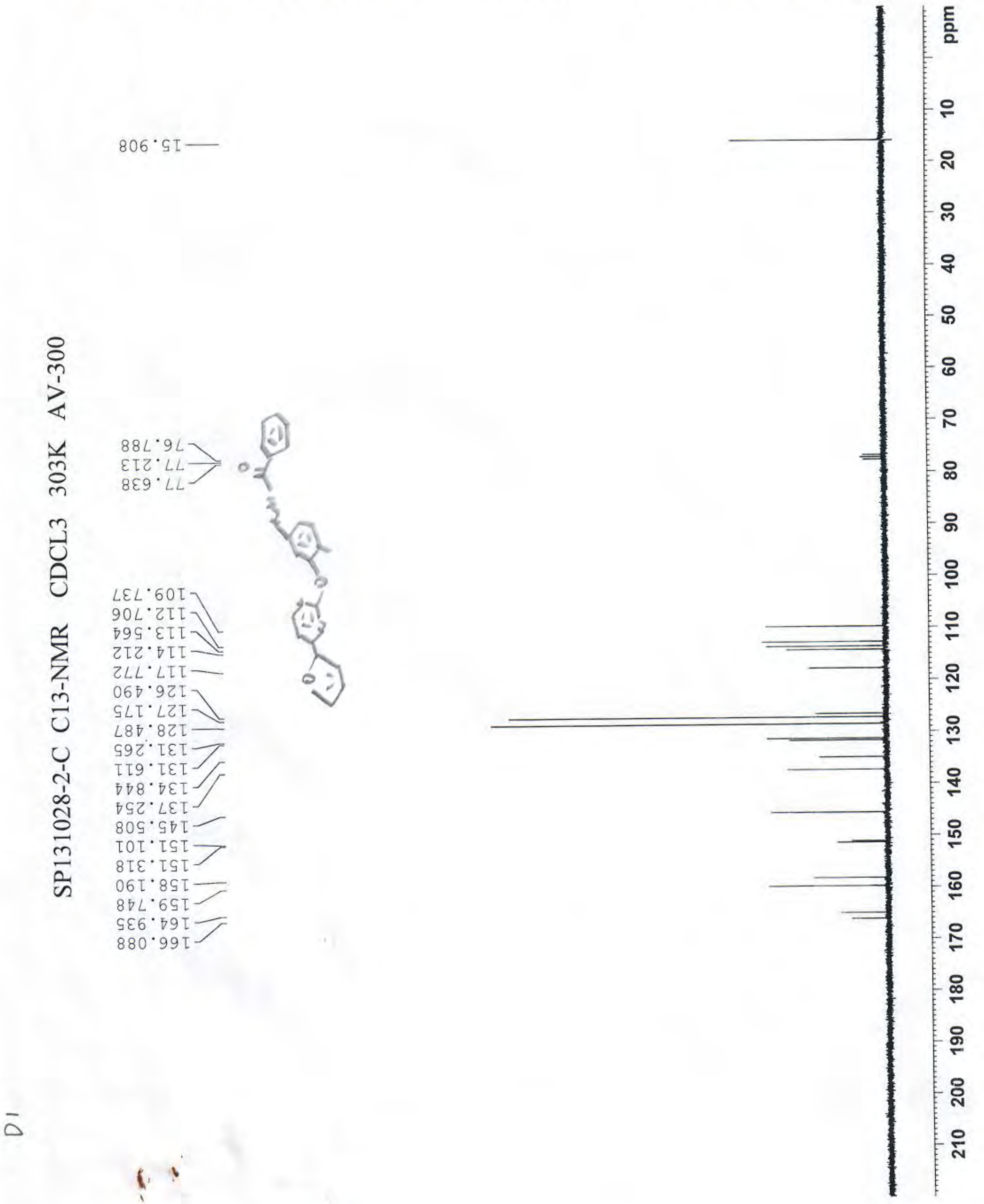

SP131028-2-C C13-NMR CDCL3 303K AV-300
15.908
76.788
77.213
77.638
109.737
112.706
113.564
114.212
117.772
126.490
127.175
128.487
131.265
131.611
134.844
137.254
145.508
151.101
151.318
158.190
159.748
164.935
166.088
D1
ppm
10
20
30
40
50
60
70
80
90
100
110
120
130
140
150
160
170
180
190
200
210

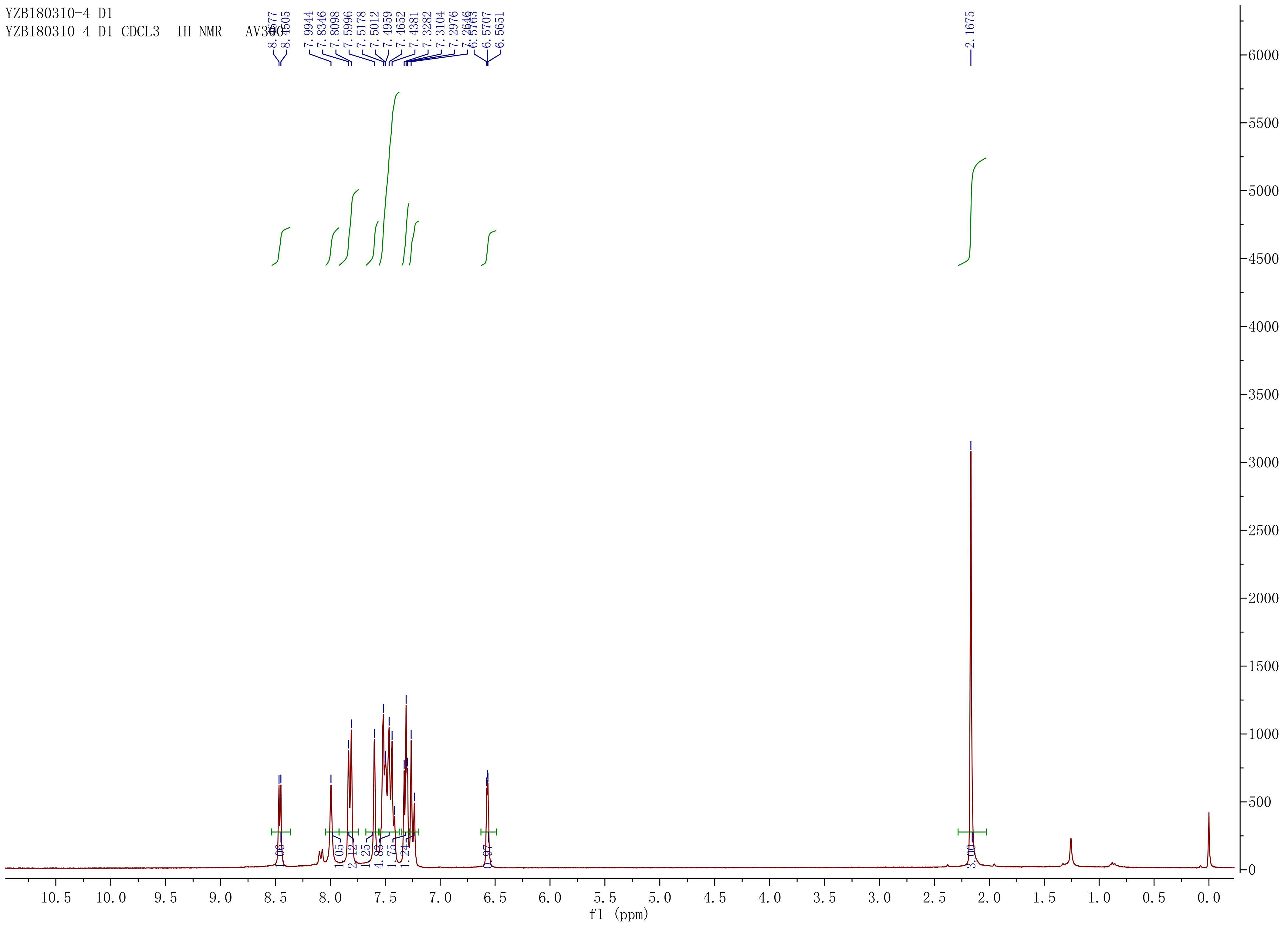
YZB180310-4 D1
YZB180310-4 D1 CDCL3 1H NMR AV300
8.4505
7.9944
7.8346
7.8098
7.5996
7.5178
7.5012
7.4959
7.4652
7.4381
7.3282
7.3104
7.2976
6.5763
6.5707
6.5651
2.1675
1.06
1.05
4.83
0.97
10.5
10.0
9.5
9.0
8.5
8.0
7.5
7.0
6.5
6.0
5.5
5.0
4.5
4.0
3.5
3.0
2.5
2.0
1.5
1.0
0.5
0.0
f1 (ppm)
6000
5500
5000
4500
4000
3500
3000
2500
2000
1500
1000
500
0

**Sample Name**		**Position**	p1A9	**Instrument Name**	Instrument 1
**User Name**	QTOF-PC\QTOF	**Inj Vol**	0.1	**InjPosition**	
**Sample Type**	Sample	**IRM Calibration Status**	Success	**Data Filename**	D1-p.d
**ACQ Method**	20110418-MSonly-p.m	**Comment**		**Acquired Time**	3/12/2018 4:13:43 PM

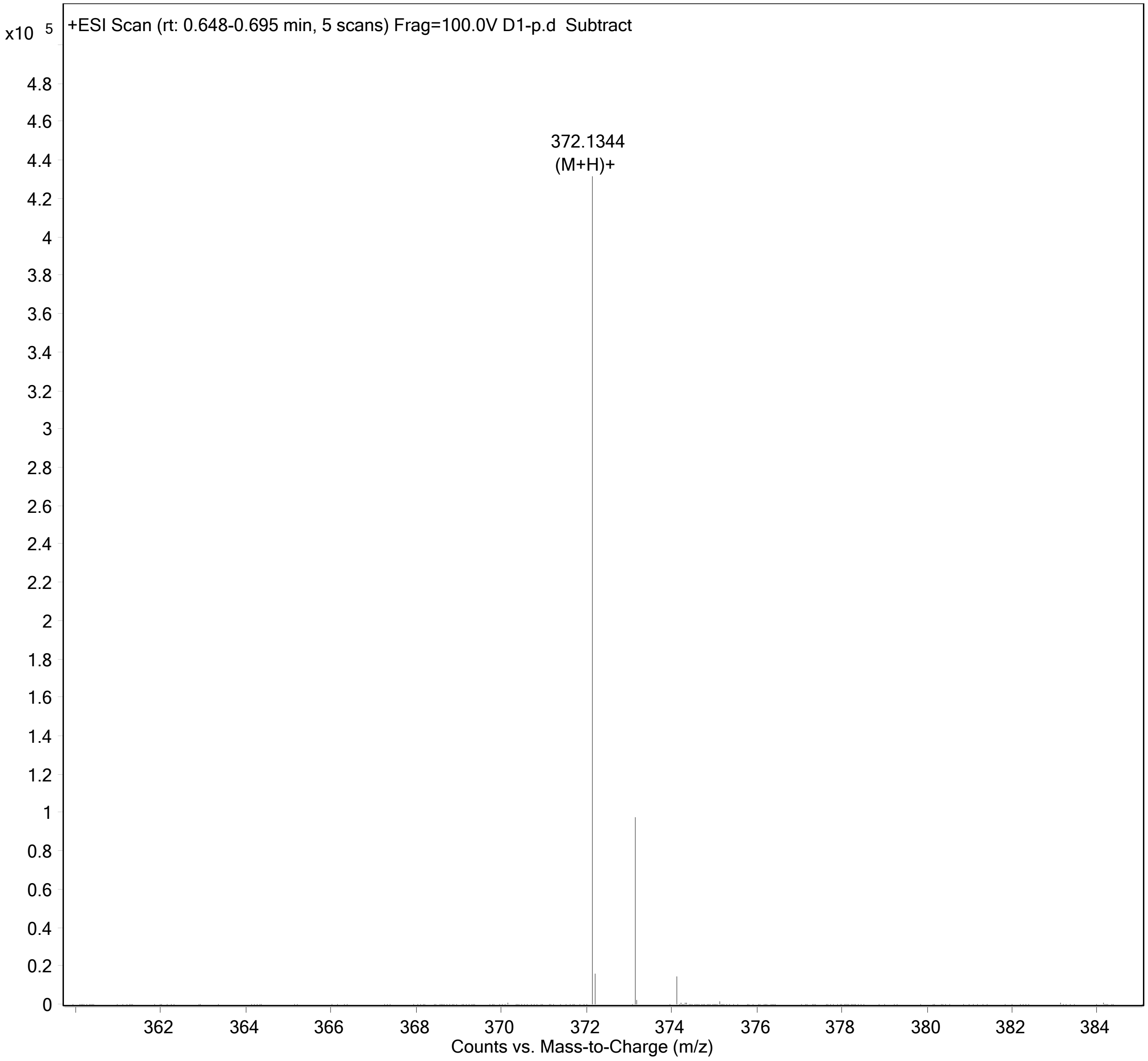

D2

SP130116-2-C C13-NMR CDCL3 303K AV-300

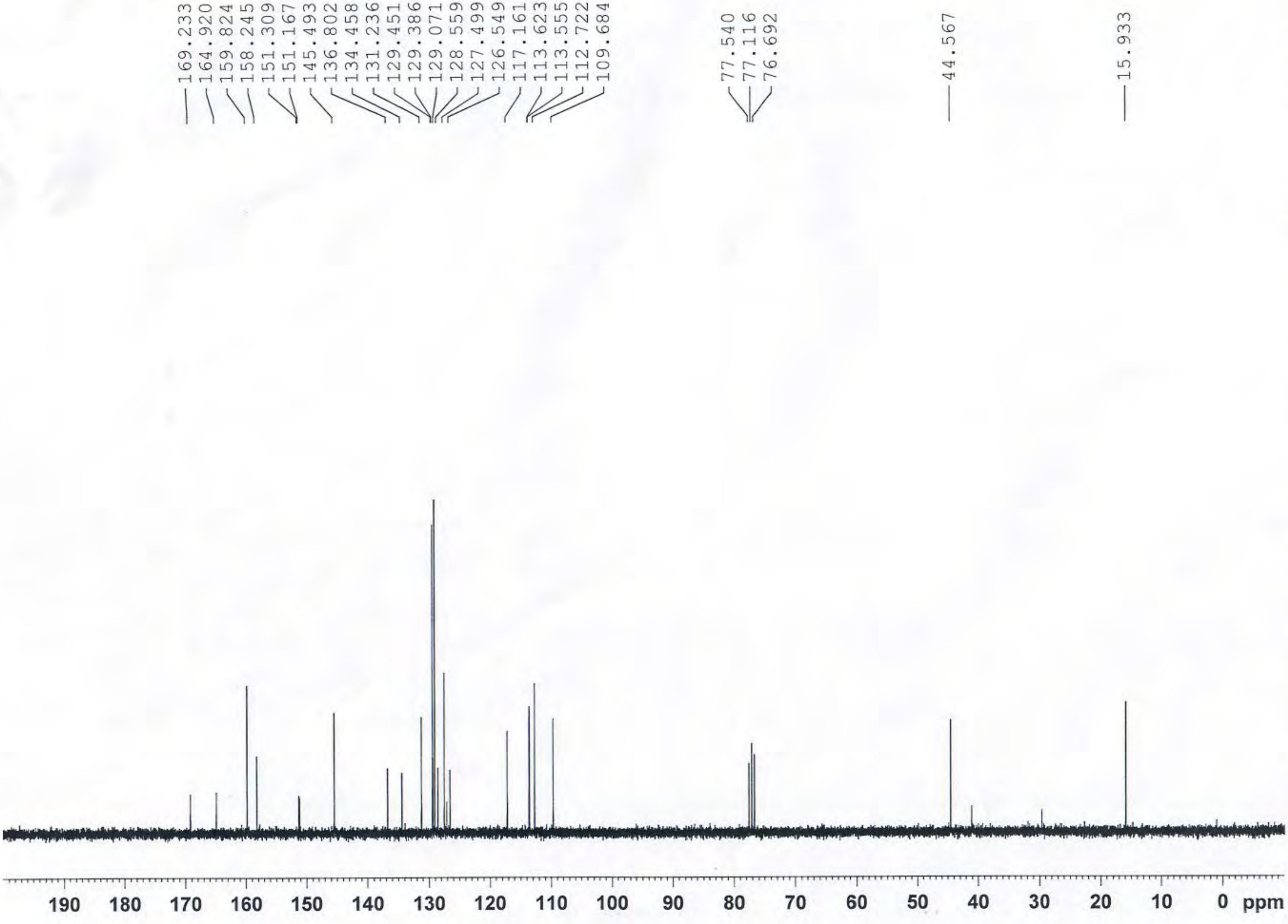

D2

SP130116-2 CDCL3 1HNMR AV300

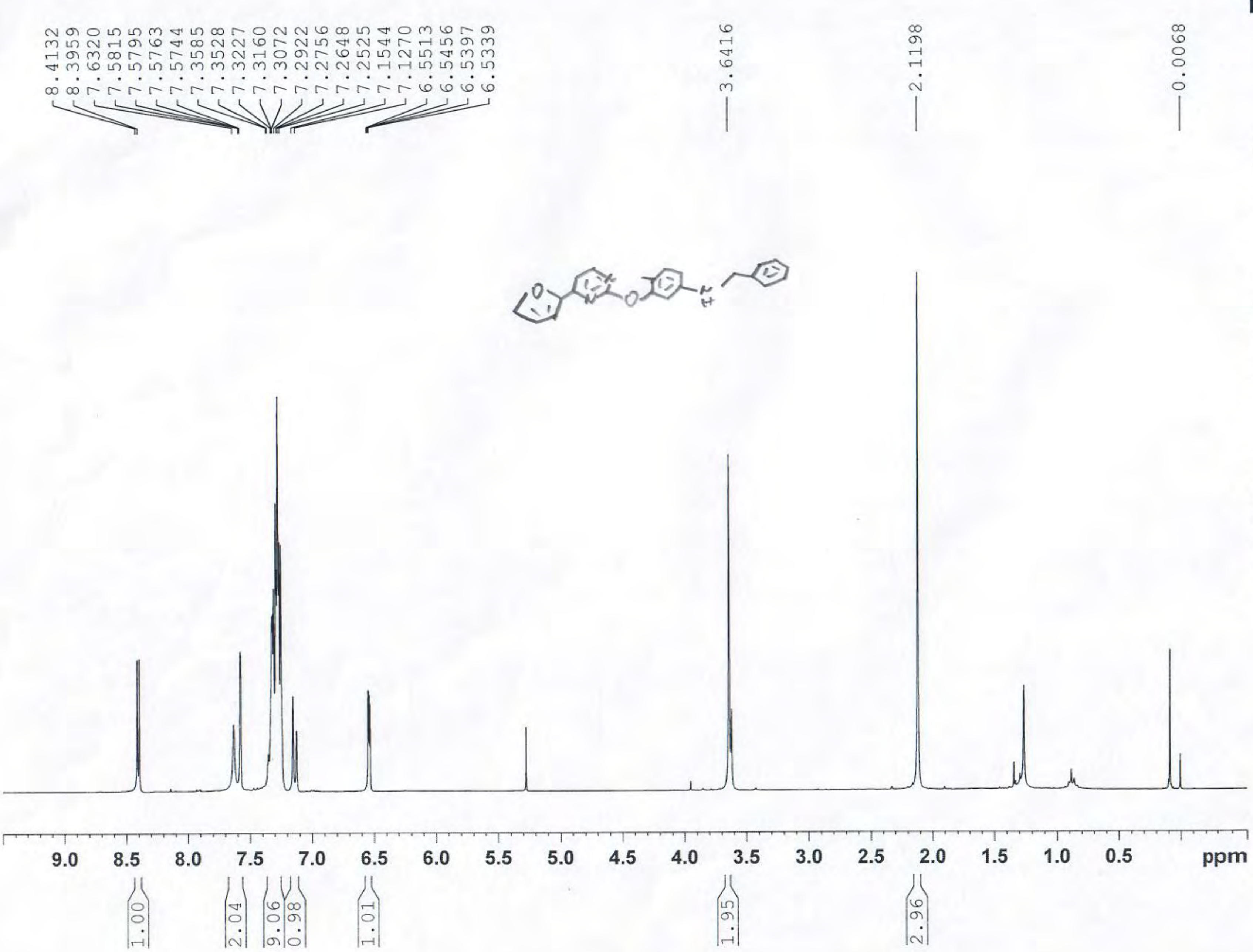

**Sample Name**		**Position**	p1B1	**Instrument Name**	Instrument 1
**User Name**	QTOF-PC\QTOF	**Inj Vol**	0.1	**InjPosition**	
**Sample Type**	Sample	**IRM Calibration Status**	Success	**Data Filename**	D2-p.d
**ACQ Method**	20110418-MSonly-p.m	**Comment**		**Acquired Time**	3/12/2018 4:16:05 PM

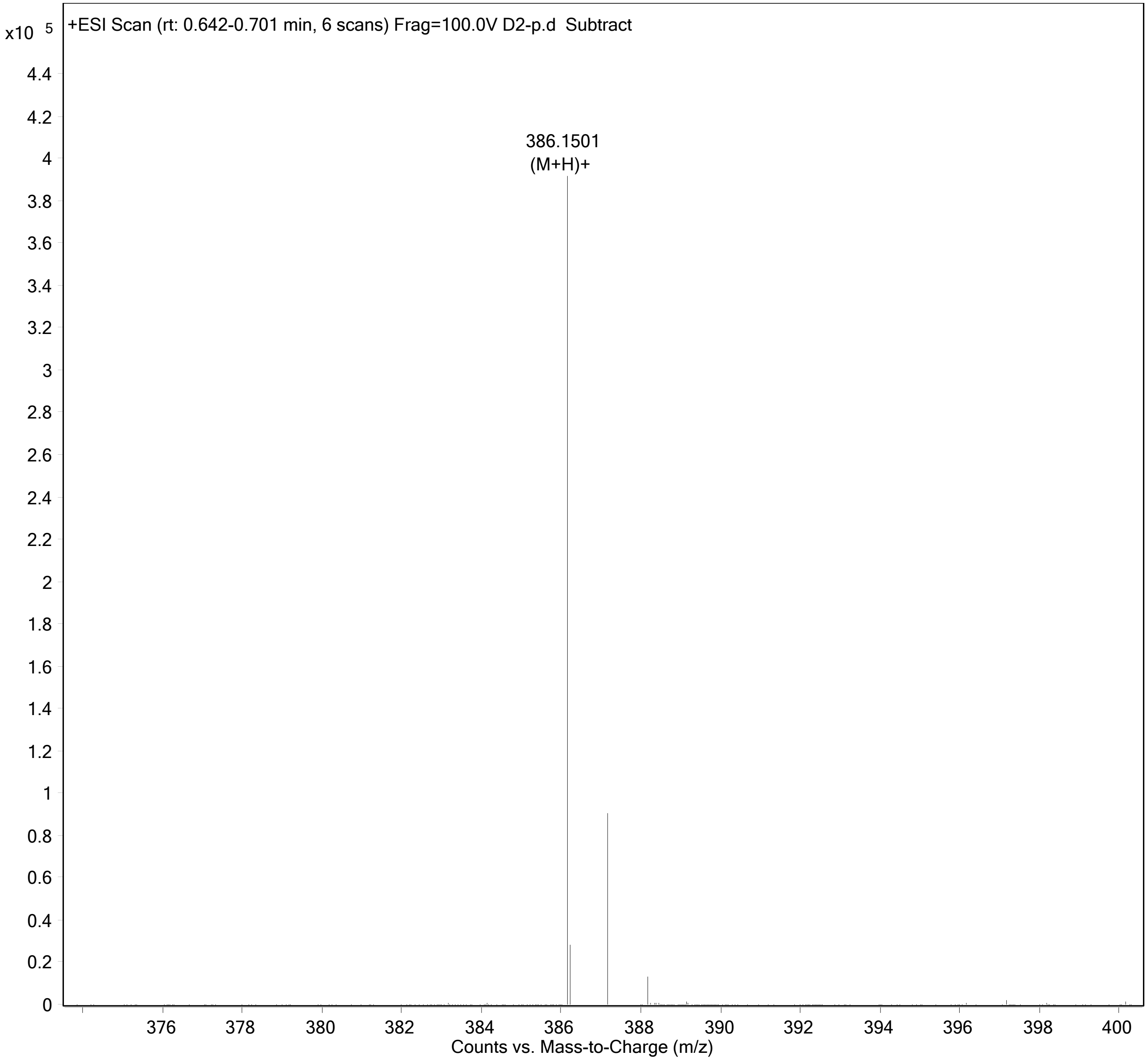

D3

SP130116-4-C C13-NMR CDCL3 303K AV-300

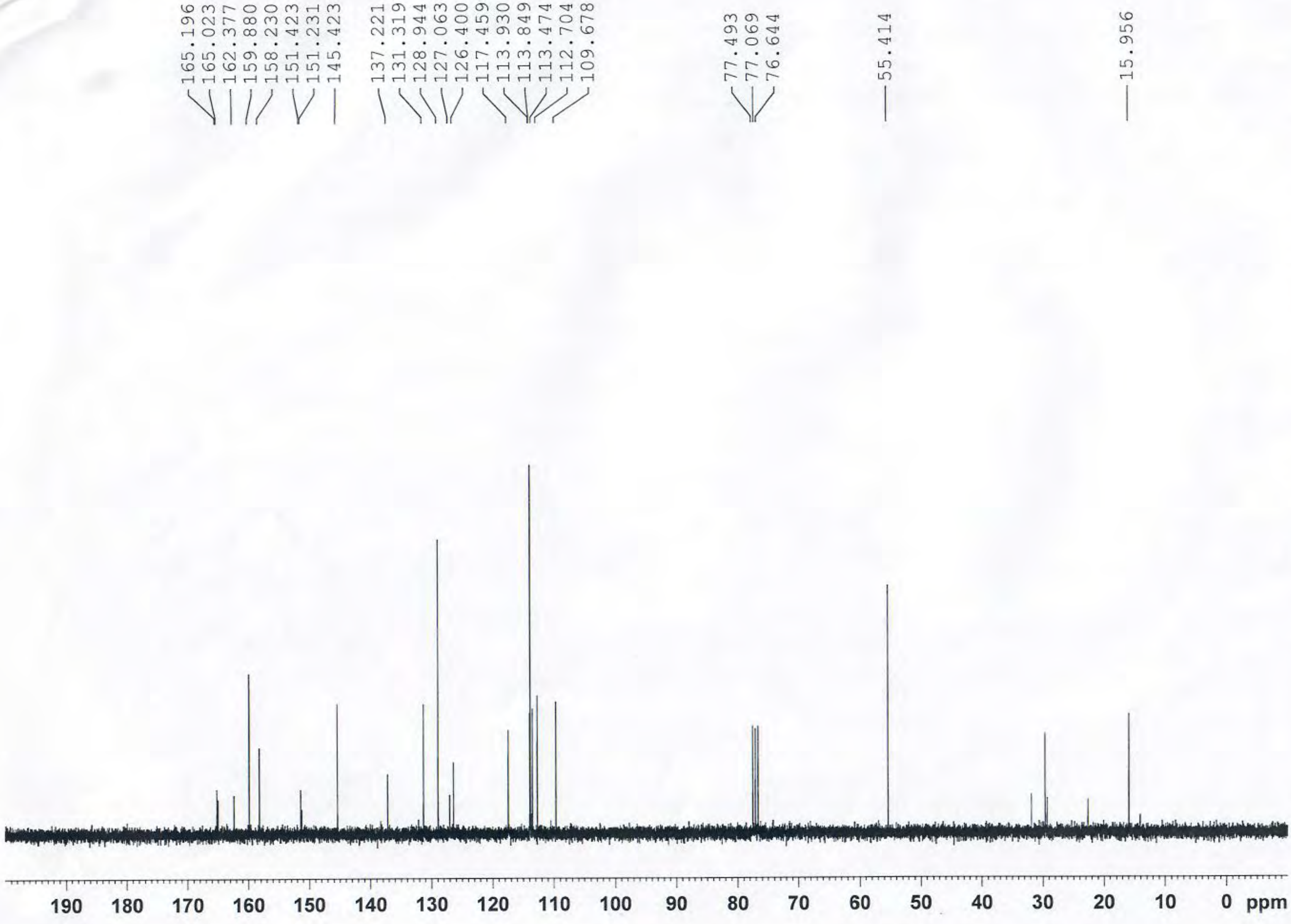

D3

SP130116-4 CDCL3 1HNMR AV300

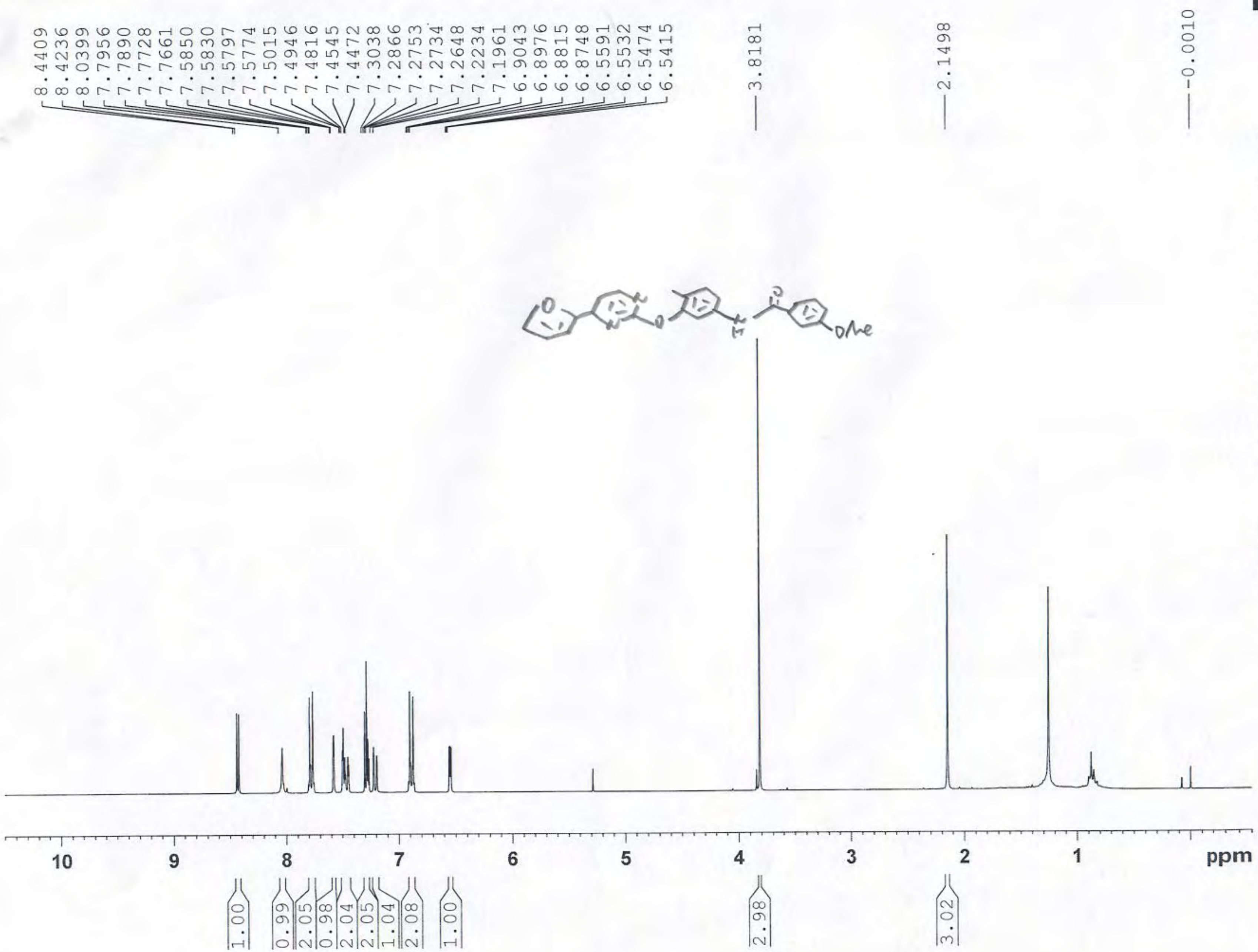

**Sample Name**		**Position**	p1B2	**Instrument Name**	Instrument 1
**User Name**	QTOF-PC\QTOF	**Inj Vol**	0.1	**InjPosition**	
**Sample Type**	Sample	**IRM Calibration Status**	Success	**Data Filename**	D3-p.d
**ACQ Method**	20110418-MSonly-p.m	**Comment**		**Acquired Time**	3/12/2018 4:18:57 PM

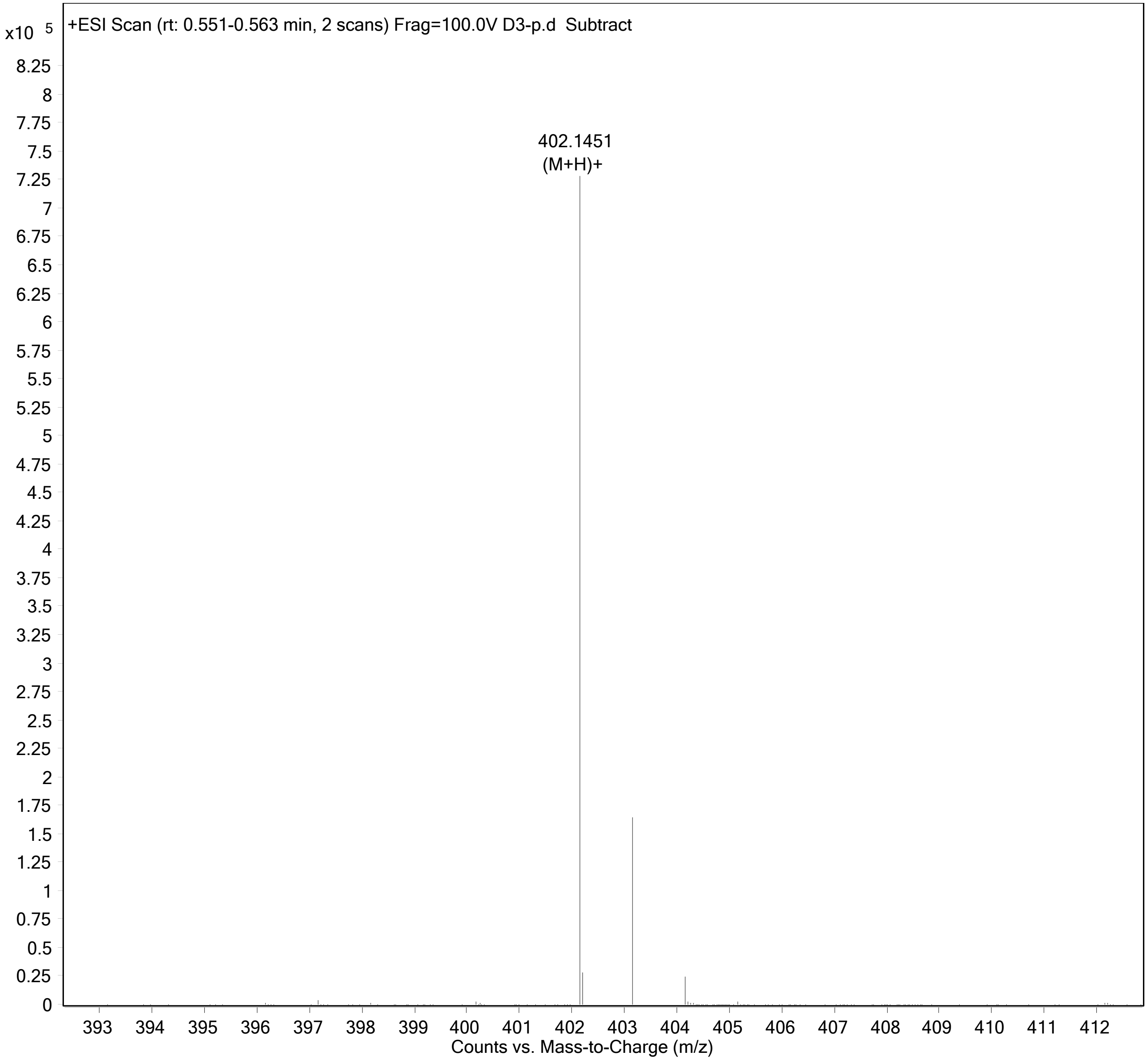

D4

SP130116-3-C C13-NMR CDCL3 303K AV-300

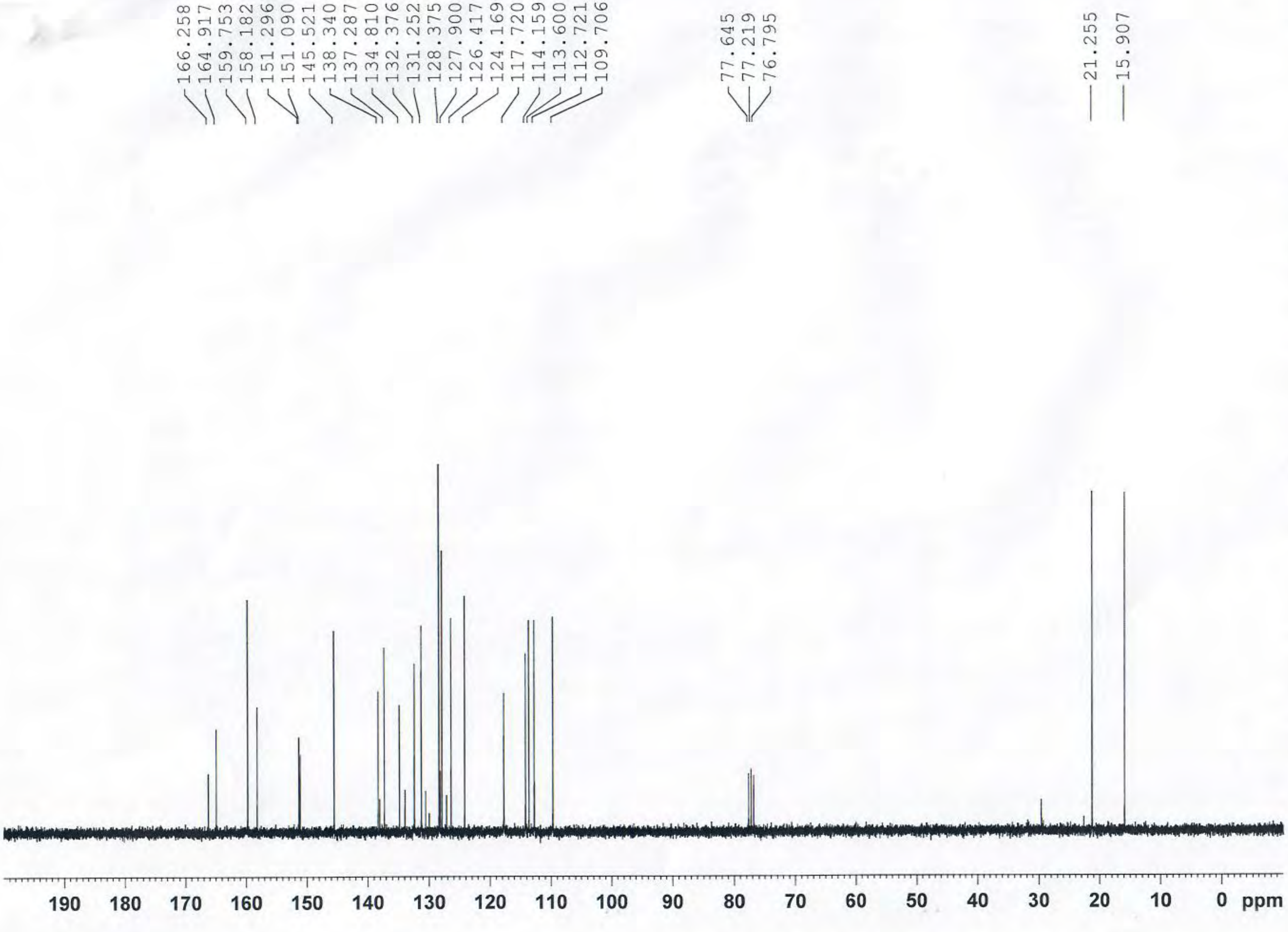

D4

SP130116-3 CDCL3 1HNMR AV300

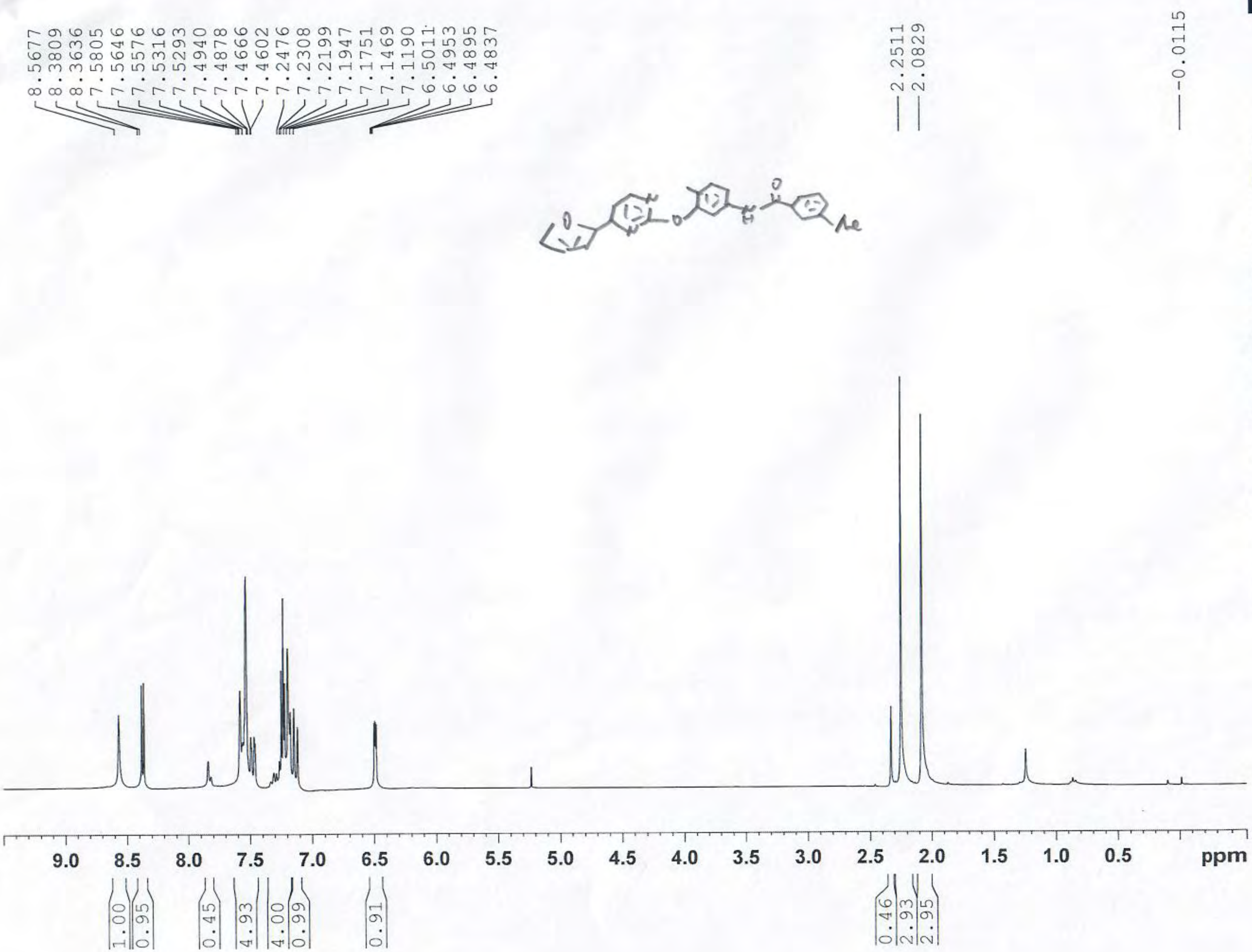

**Sample Name**		**Position**	p1B3	**Instrument Name**	Instrument 1
**User Name**	QTOF-PC\QTOF	**Inj Vol**	0.1	**InjPosition**	
**Sample Type**	Sample	**IRM Calibration Status**	Success	**Data Filename**	D4-p.d
**ACQ Method**	20110418-MSonly-p.m	**Comment**		**Acquired Time**	3/12/2018 4:21:07 PM

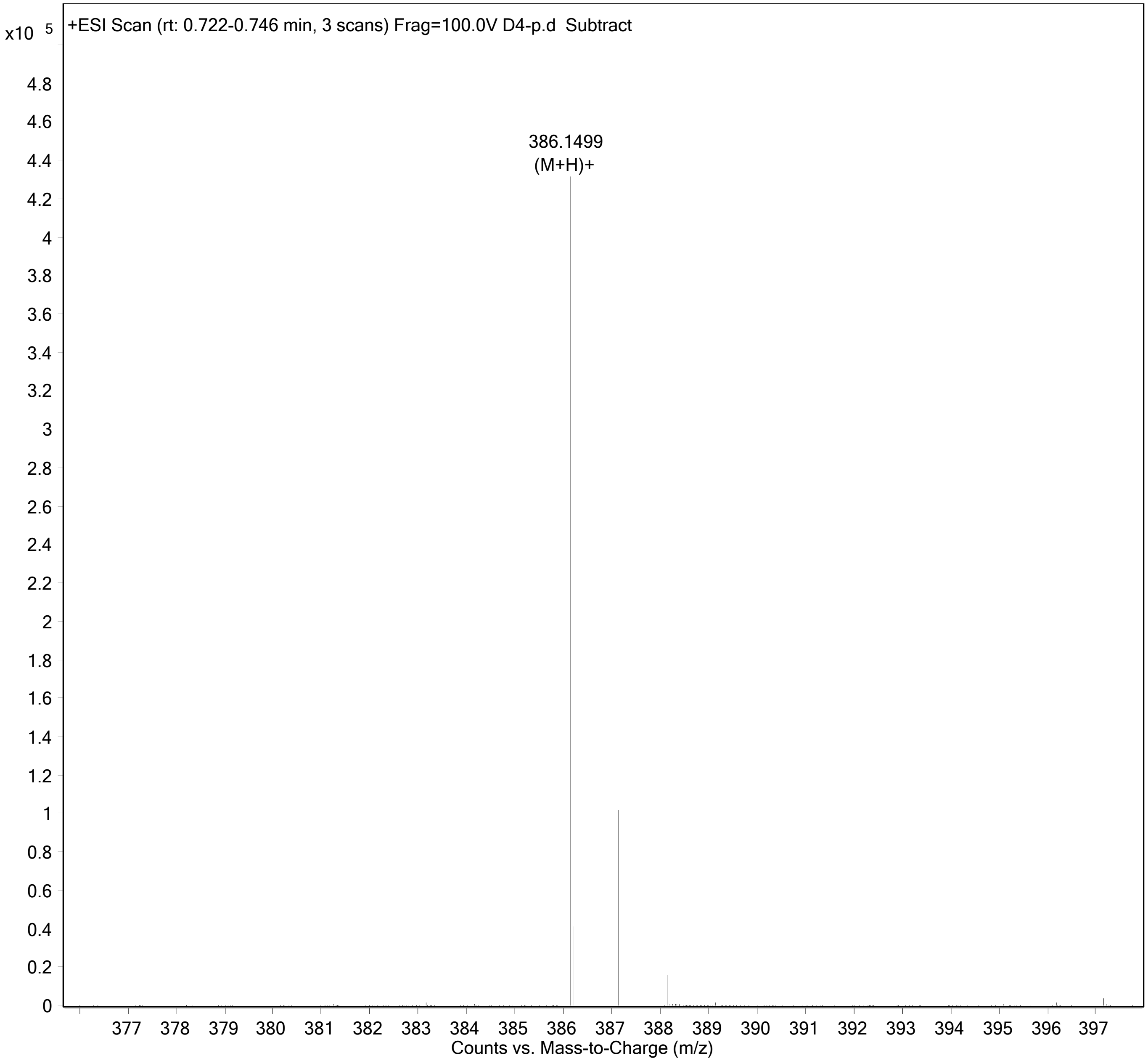

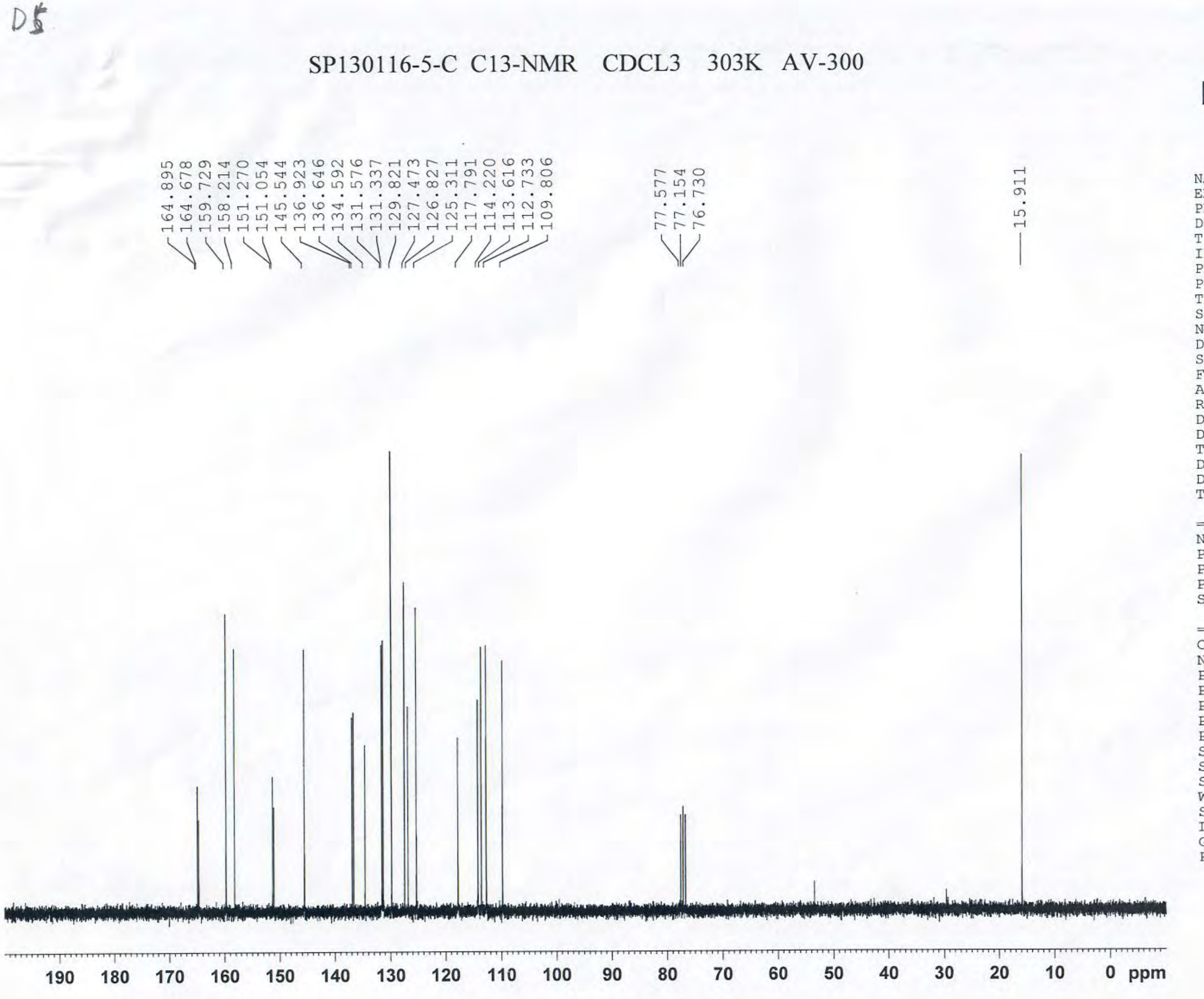
D5
SP130116-5-C C13-NMR CDCL3 303K AV-300
164.895
164.678
159.729
158.214
151.270
151.054
145.544
136.923
136.646
134.592
131.576
131.337
129.821
127.473
126.827
125.311
117.791
114.220
113.616
112.733
109.806
77.577
77.154
76.730
15.911
190 180 170 160 150 140 130 120 110 100 90 80 70 60 50 40 30 20 10 0 ppm

D5

SP130116-5 CDCL3 1HNMR AV300

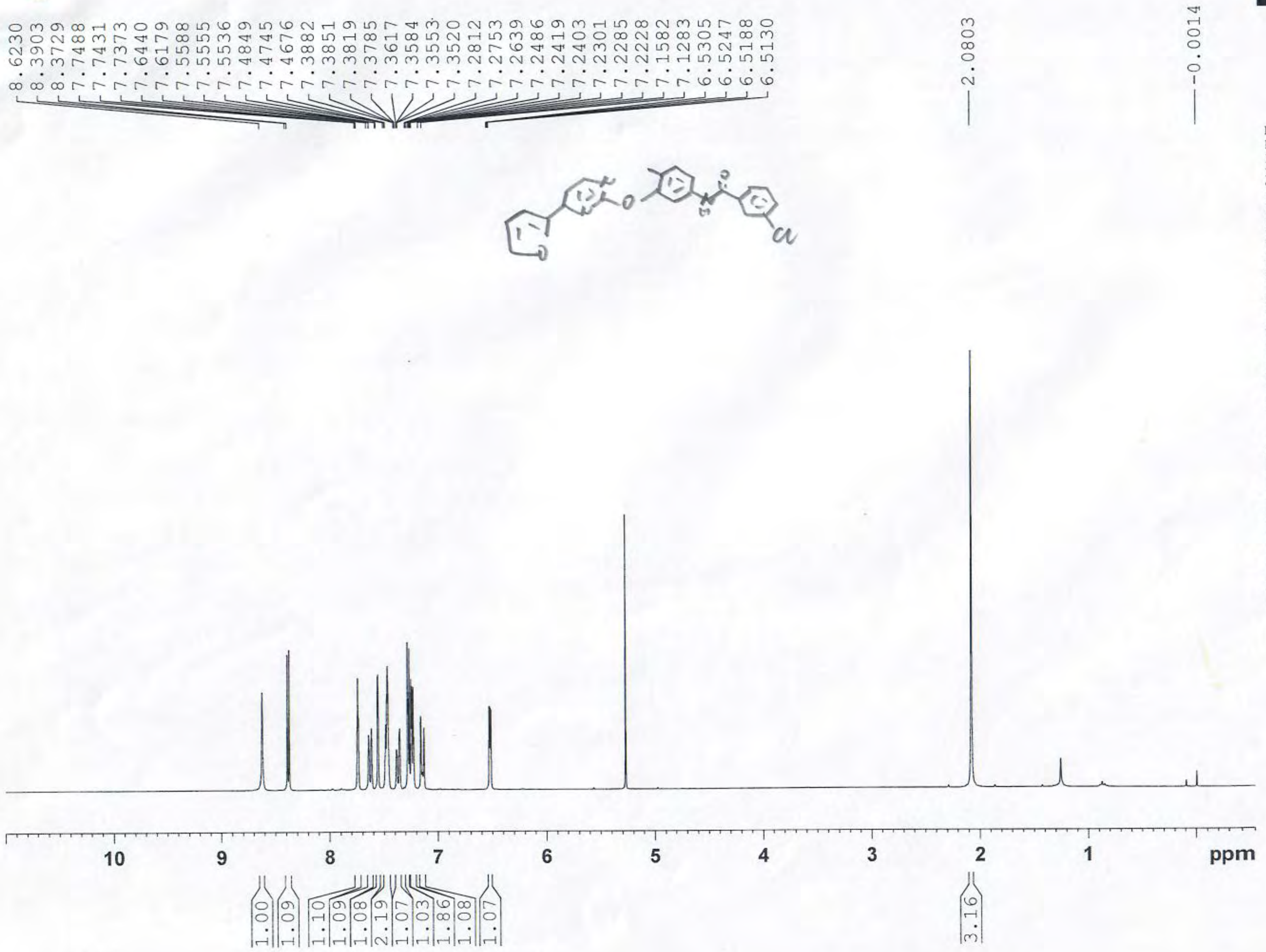

**Sample Name**		**Position**	p1B4	**Instrument Name**	Instrument 1
**User Name**	QTOF-PC\QTOF	**Inj Vol**	0.1	**InjPosition**	
**Sample Type**	Sample	**IRM Calibration Status**	Success	**Data Filename**	D5-p.d
**ACQ Method**	20110418-MSonly-p.m	**Comment**		**Acquired Time**	3/12/2018 4:23:06 PM

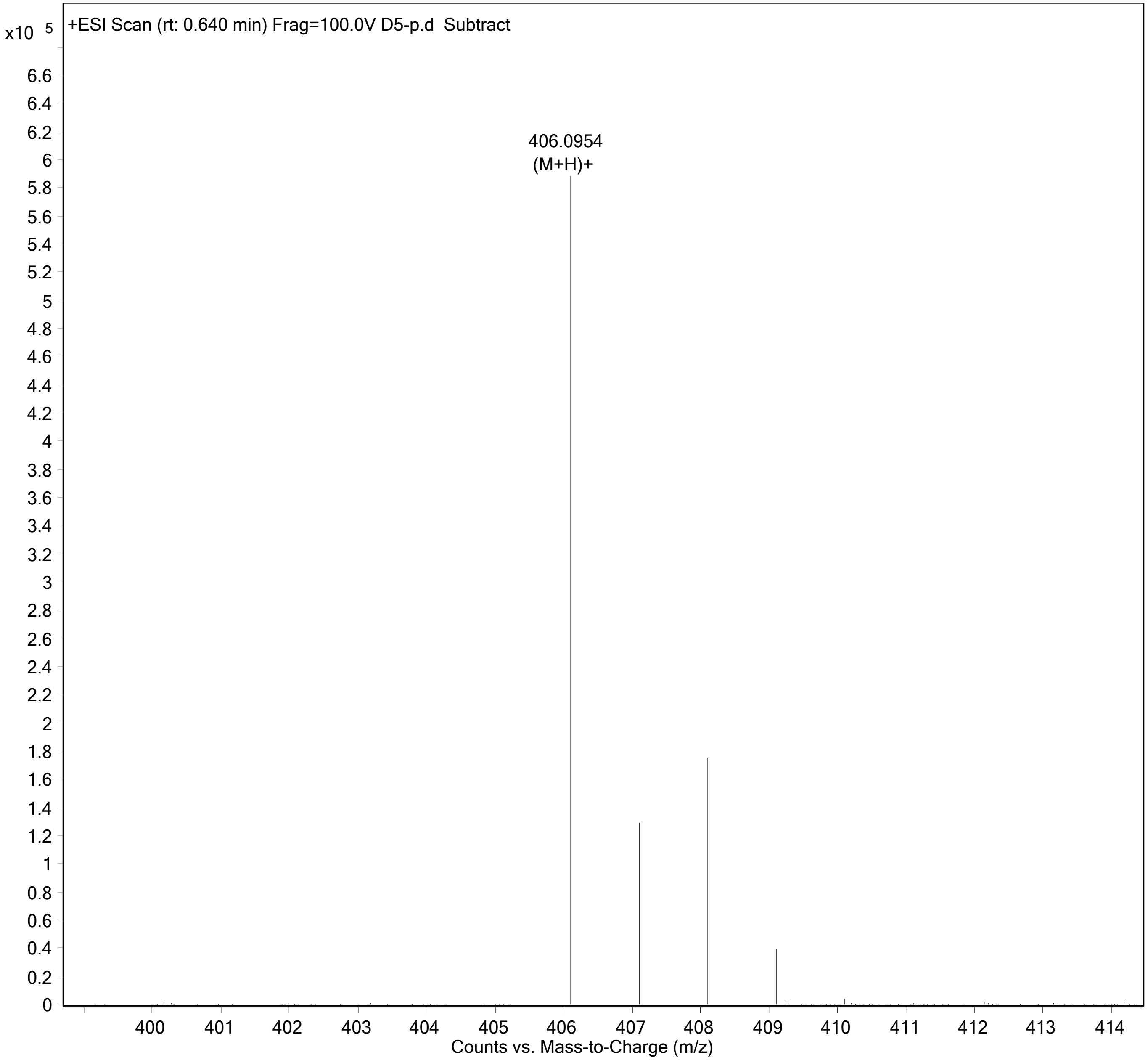

D6

SP140310-2-C C13-NMR CDCL3 303K AV-300

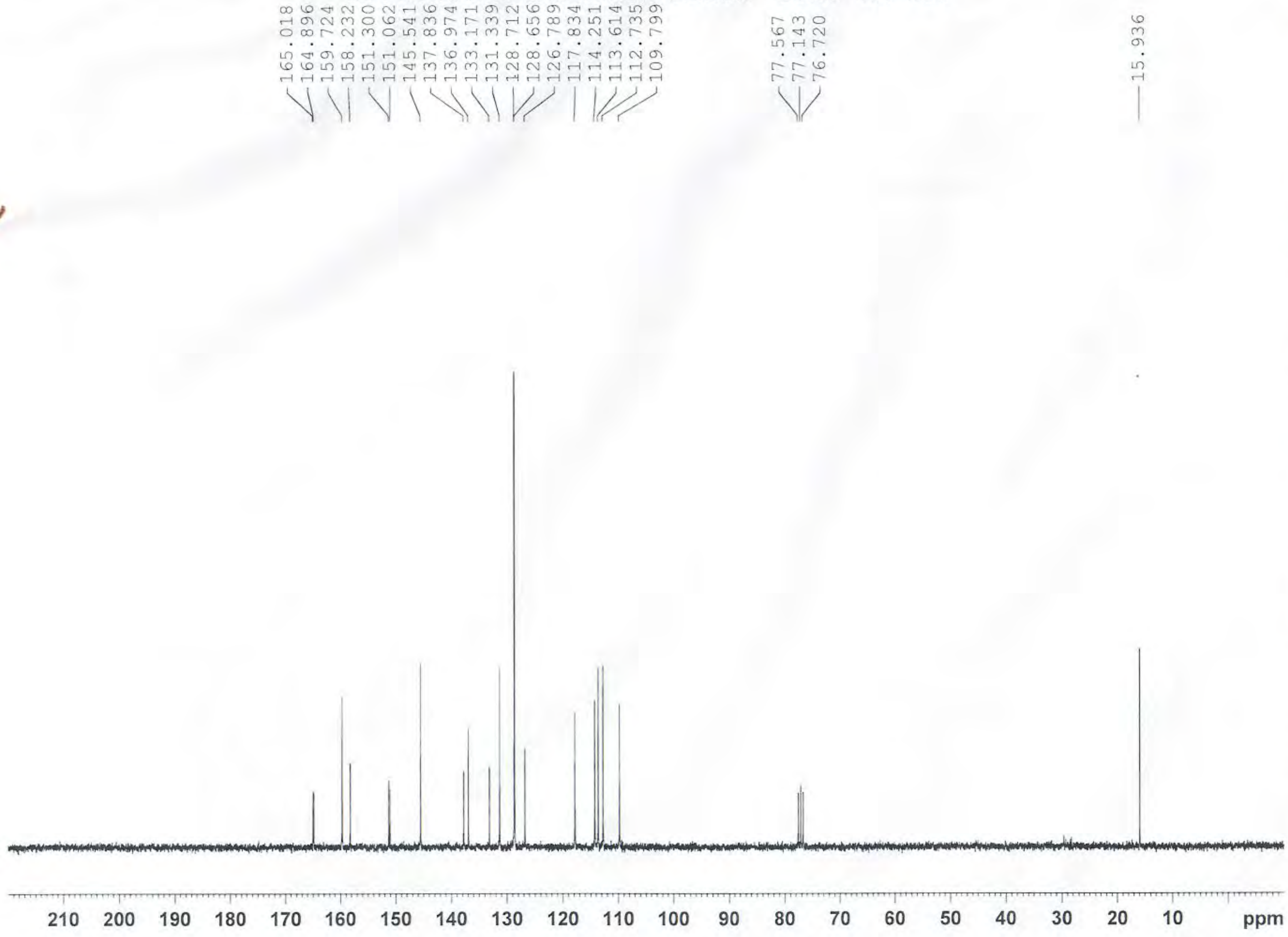

D6.

SP140310-2 CDCL3 1HNMR AV300

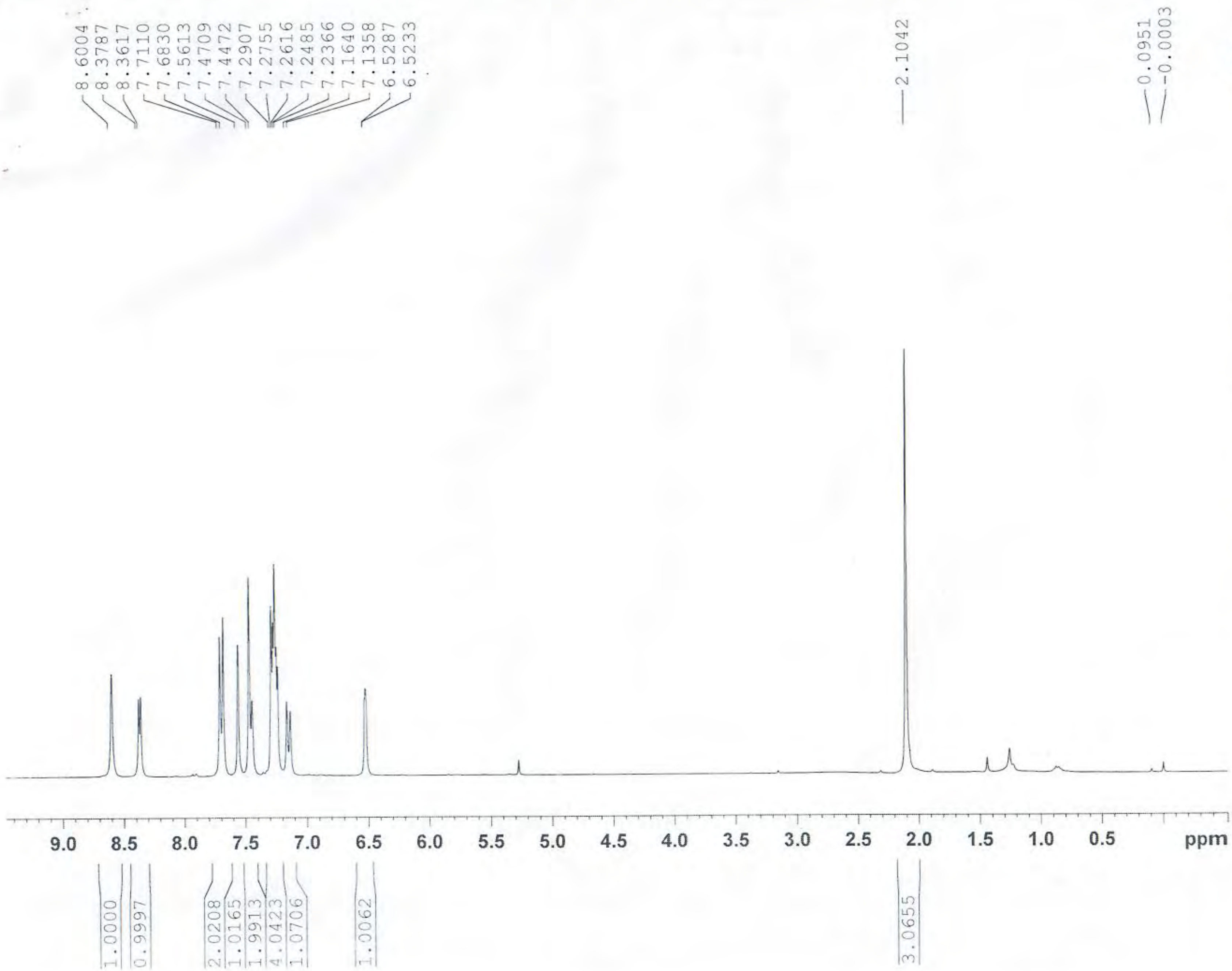

**Sample Name**		**Position**	p1B5	**Instrument Name**	Instrument 1
**User Name**	QTOF-PC\QTOF	**Inj Vol**	0.1	**InjPosition**	
**Sample Type**	Sample	**IRM Calibration Status**	Success	**Data Filename**	D6-p.d
**ACQ Method**	20110418-MSonly-p.m	**Comment**		**Acquired Time**	3/12/2018 4:25:23 PM

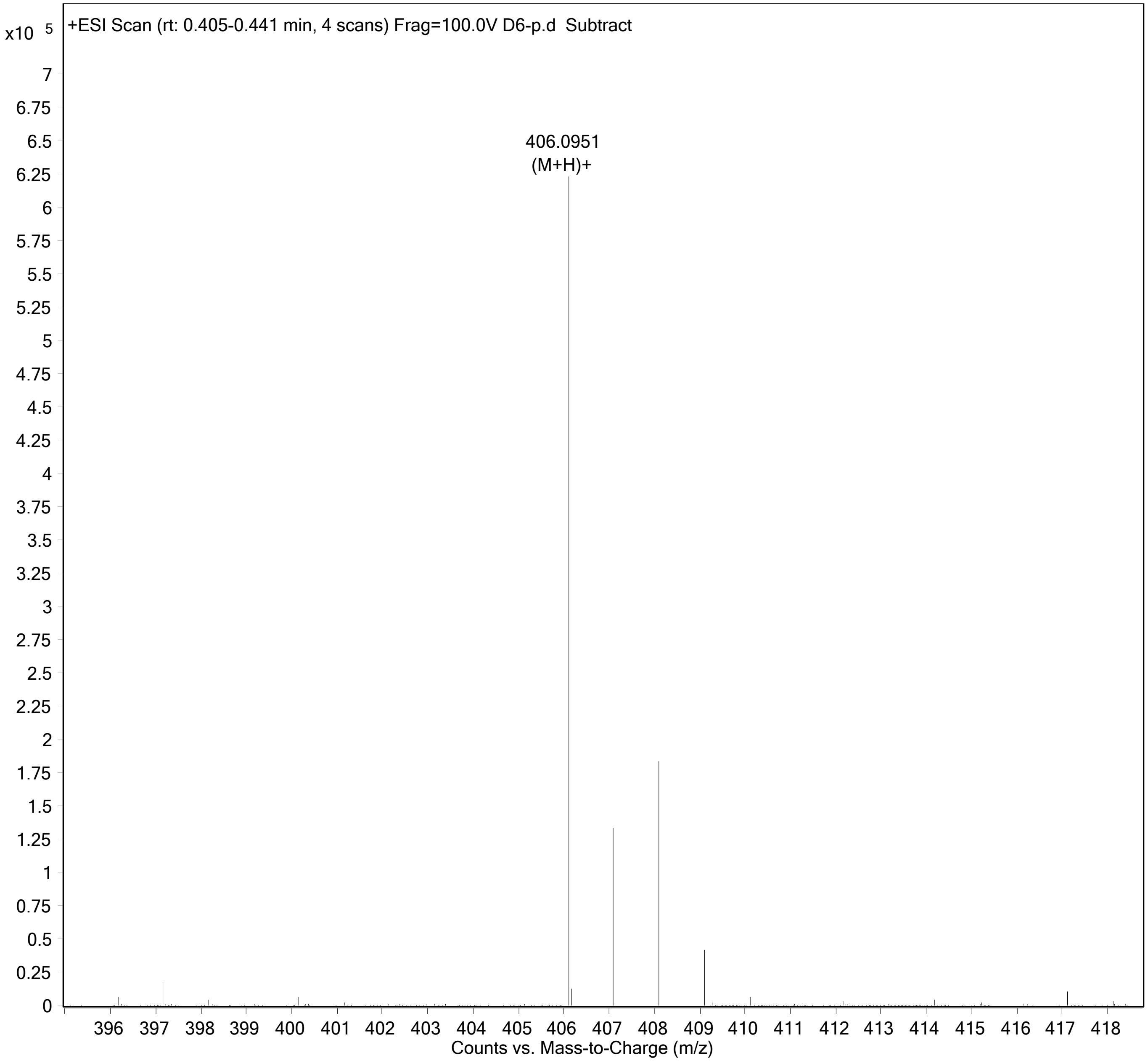

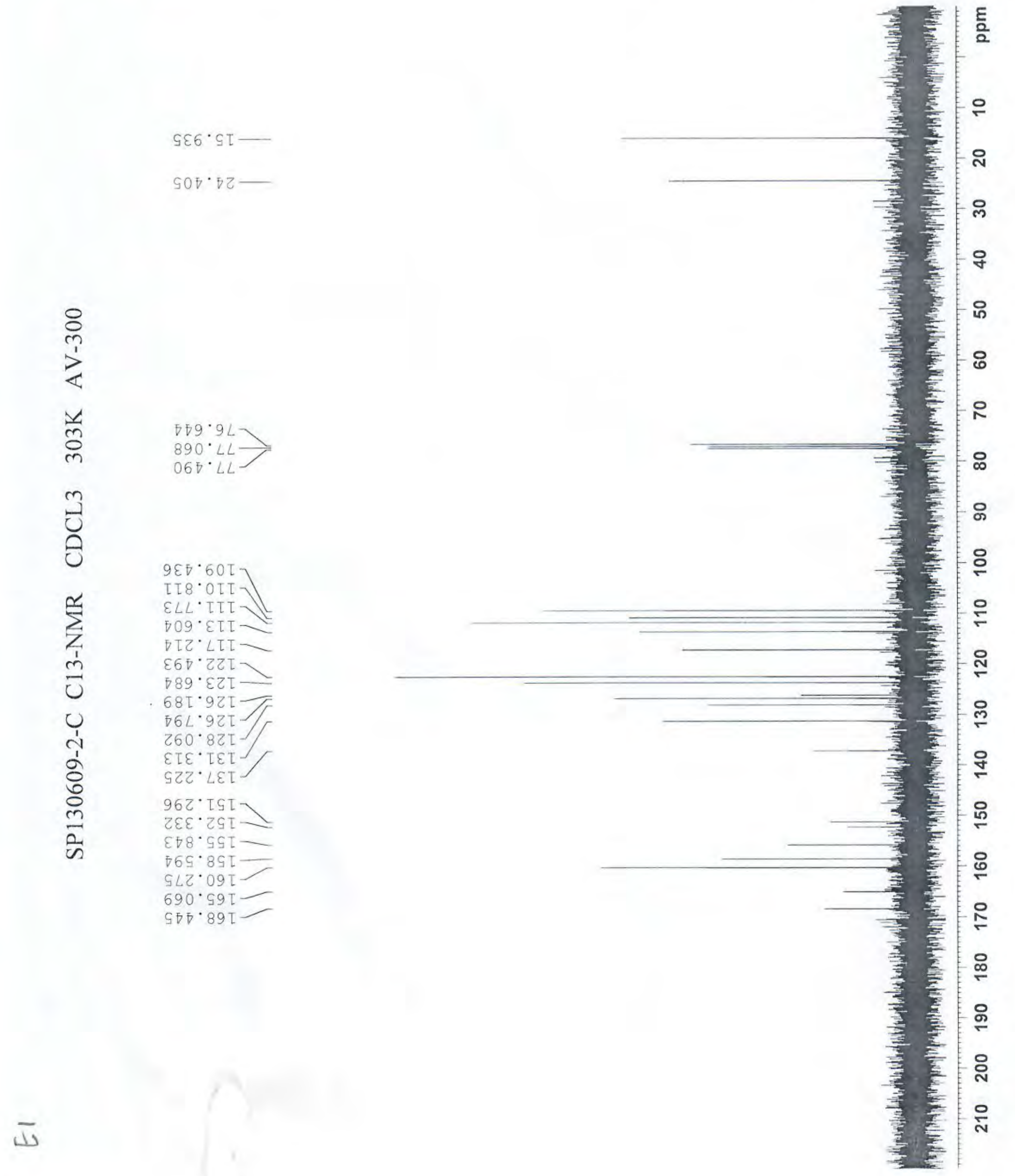
SP130609-2-C C13-NMR CDCL3 303K AV-300
15.935
24.405
76.644
77.068
77.490
109.436
110.811
111.773
113.604
117.214
122.493
123.684
126.189
126.794
128.092
131.313
137.225
151.296
152.332
155.843
158.594
160.275
165.069
168.445
ppm
10
20
30
40
50
60
70
80
90
100
110
120
130
140
150
160
170
180
190
200
210
13

E1

SP20130609-2 CDCL3 1HNMR AV300

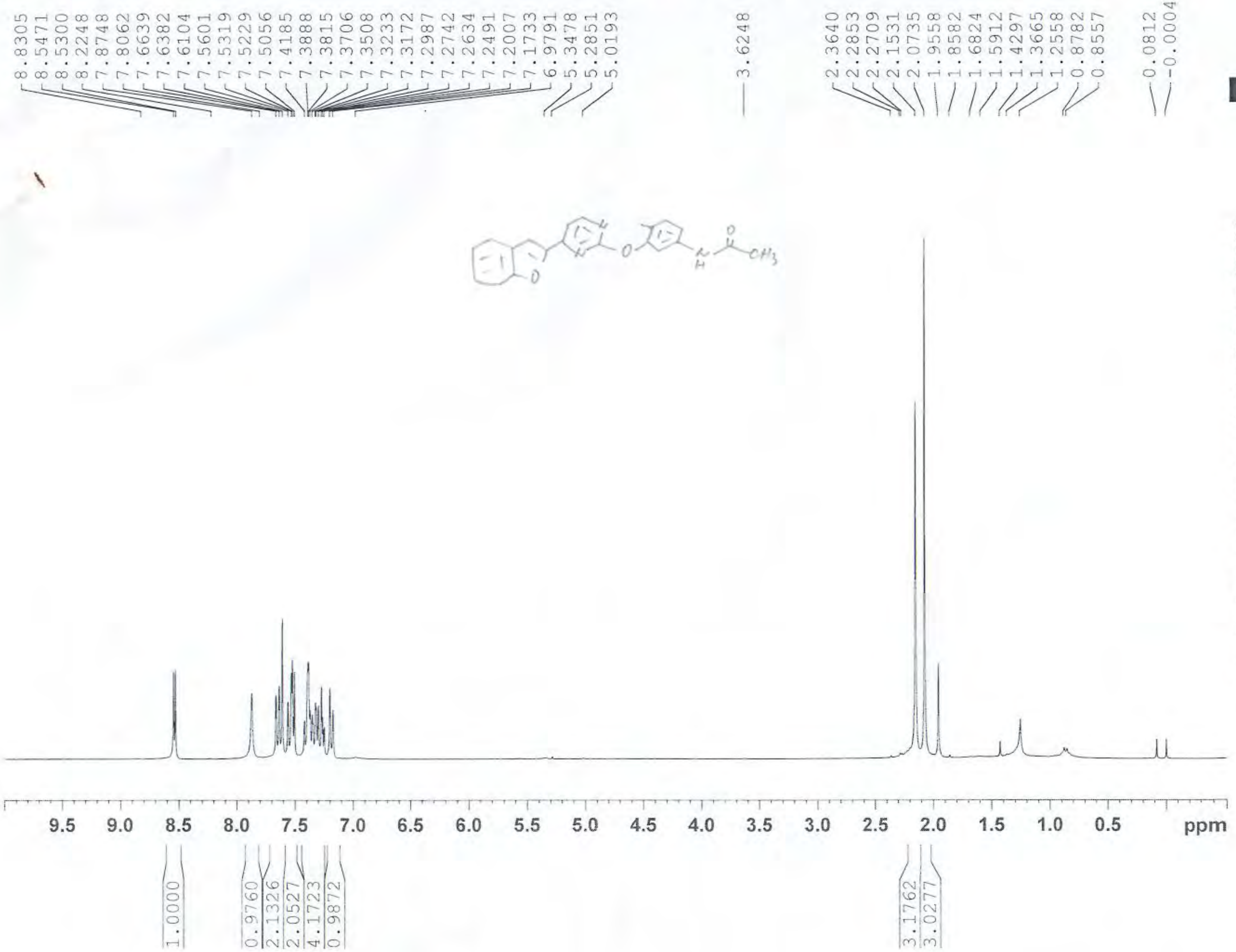

**Sample Name**		**Position**	p1B6	**Instrument Name**	Instrument 1
**User Name**	QTOF-PC\QTOF	**Inj Vol**	0.1	**InjPosition**	
**Sample Type**	Sample	**IRM Calibration Status**	Success	**Data Filename**	E1-p.d
**ACQ Method**	20110418-MSonly-p.m	**Comment**		**Acquired Time**	3/12/2018 4:27:24 PM

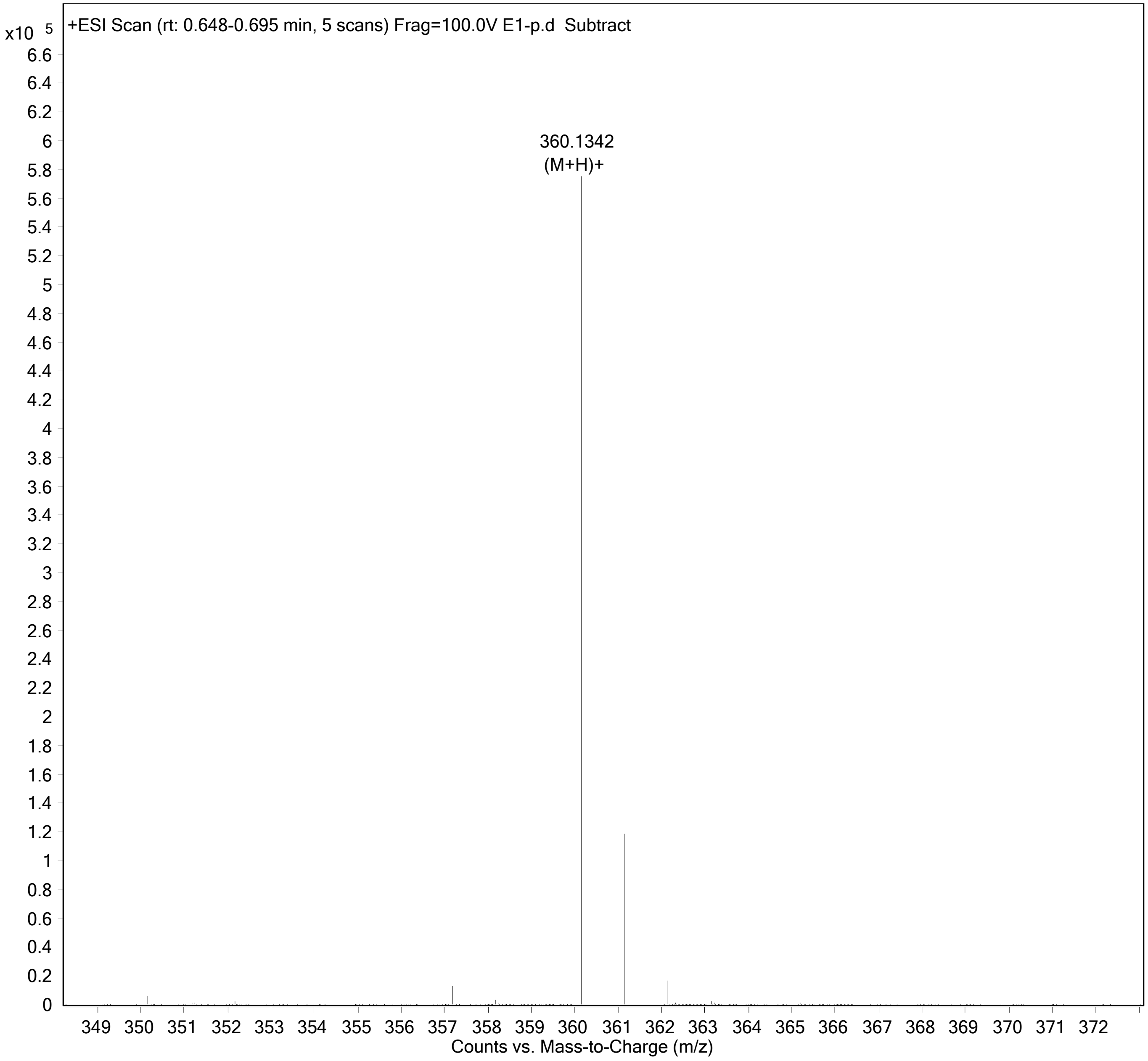

E2

SP130619-1-C C13-NMR CDCL3 303K AV-300

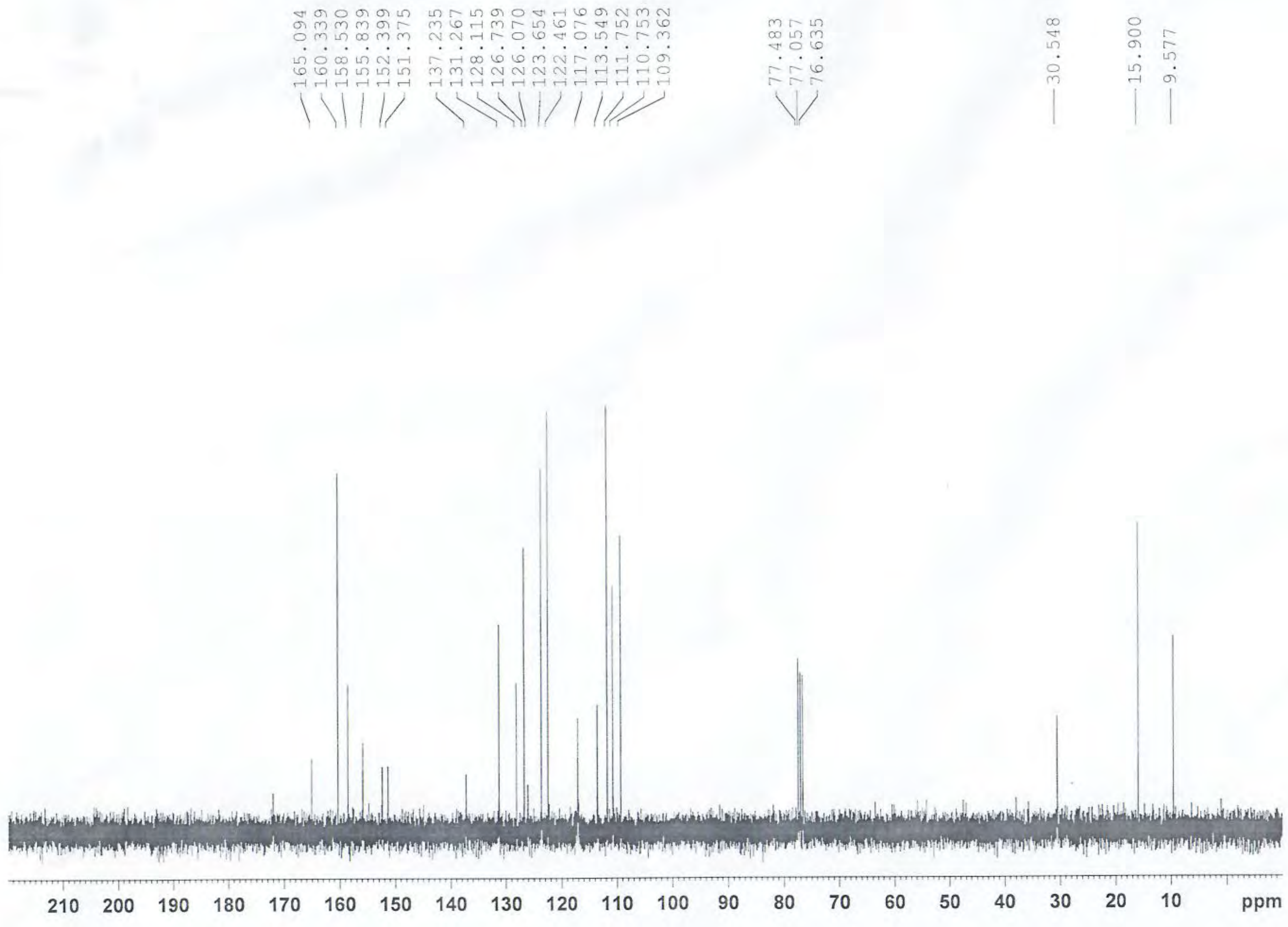

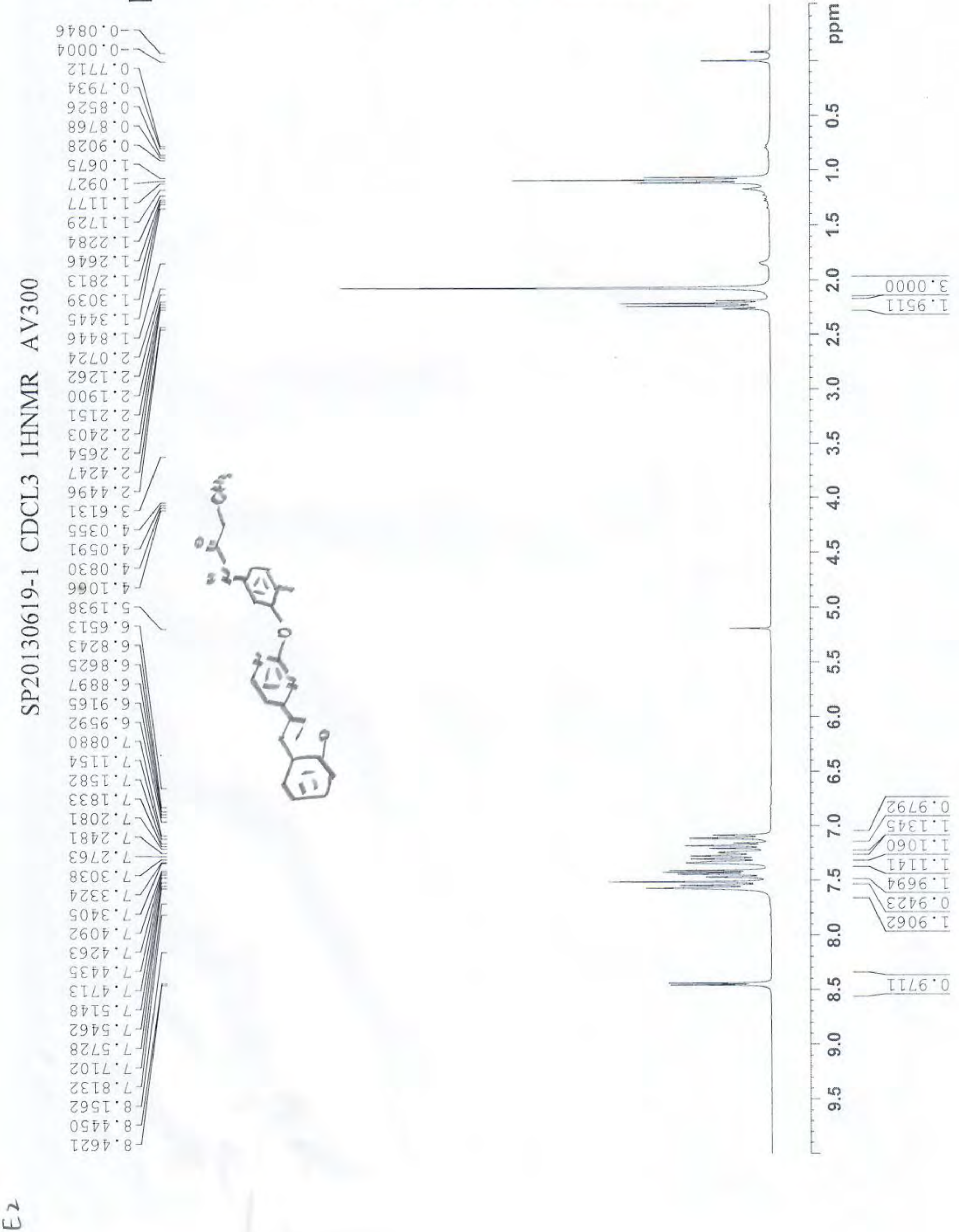
SP20130619-1 CDCL3 1HNMR AV300
-0.0846
-0.0004
0.7712
0.7934
0.8526
0.8768
0.9028
1.0675
1.0927
1.1177
1.1729
1.2284
1.2646
1.2813
1.3039
1.3445
1.8446
2.0724
2.1262
2.1900
2.2151
2.2403
2.2654
2.4247
2.4496
3.6131
4.0355
4.0591
4.0830
4.1066
5.1938
6.6513
6.8243
6.8625
6.8897
6.9165
6.9592
7.0880
7.1154
7.1582
7.1833
7.2081
7.2481
7.2763
7.3038
7.3324
7.3405
7.4092
7.4263
7.4435
7.4713
7.5148
7.5462
7.5728
7.7102
7.8132
8.1562
8.4450
8.4621
3.0000
1.9511
0.9792
1.1345
1.1060
1.1141
1.9694
0.9423
1.9062
0.9711
ppm
0.5
1.0
1.5
2.0
2.5
3.0
3.5
4.0
4.5
5.0
5.5
6.0
6.5
7.0
7.5
8.0
8.5
9.0
9.5
E2

**Sample Name**		**Position**	p1B7	**Instrument Name**	Instrument 1
**User Name**	QTOF-PC\QTOF	**Inj Vol**	0.1	**InjPosition**	
**Sample Type**	Sample	**IRM Calibration Status**	Success	**Data Filename**	E2-p.d
**ACQ Method**	20110418-MSonly-p.m	**Comment**		**Acquired Time**	3/12/2018 4:29:34 PM

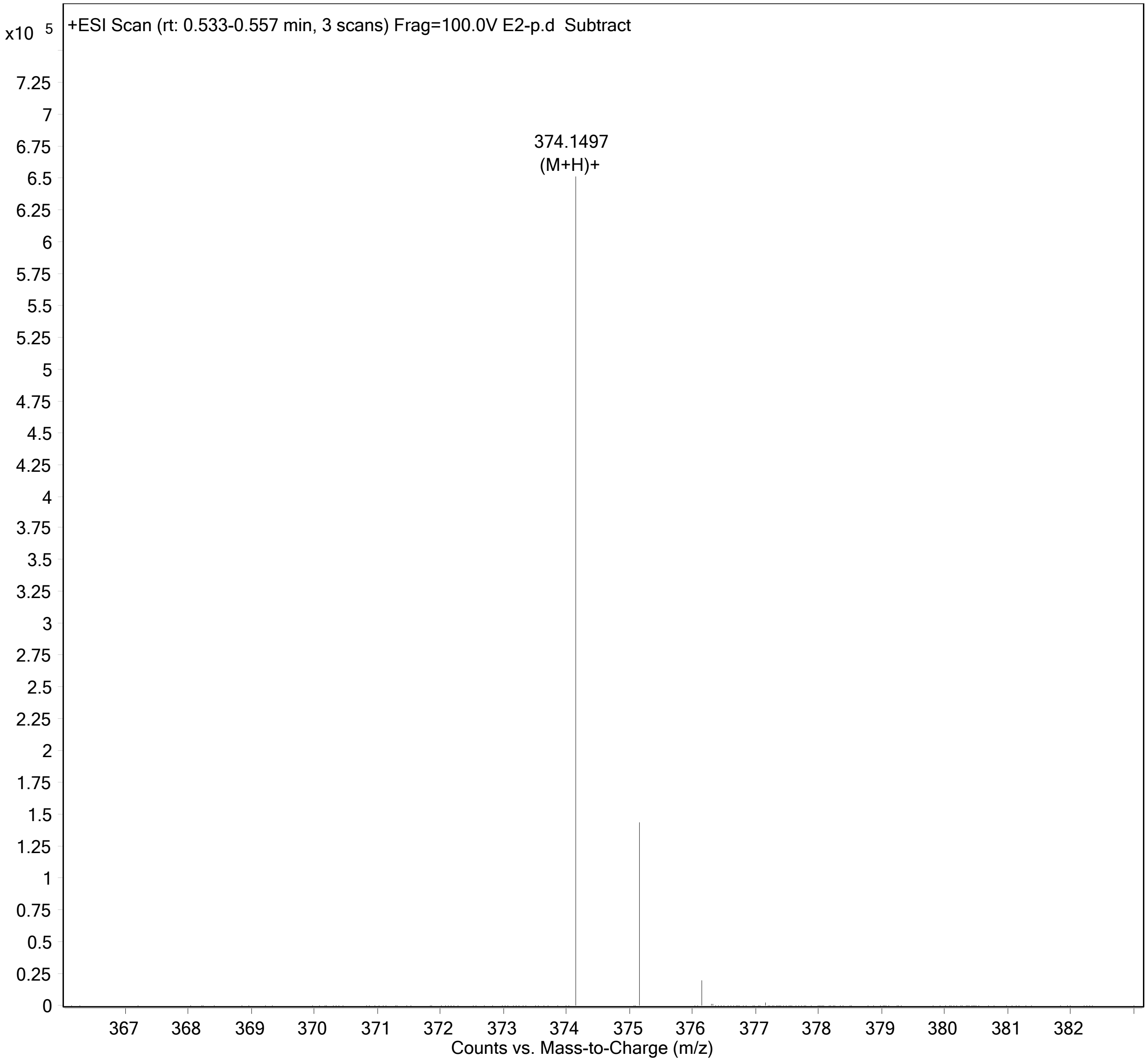

E3

SP130621-2-C C13-NMR CDCL3 303K AV-300

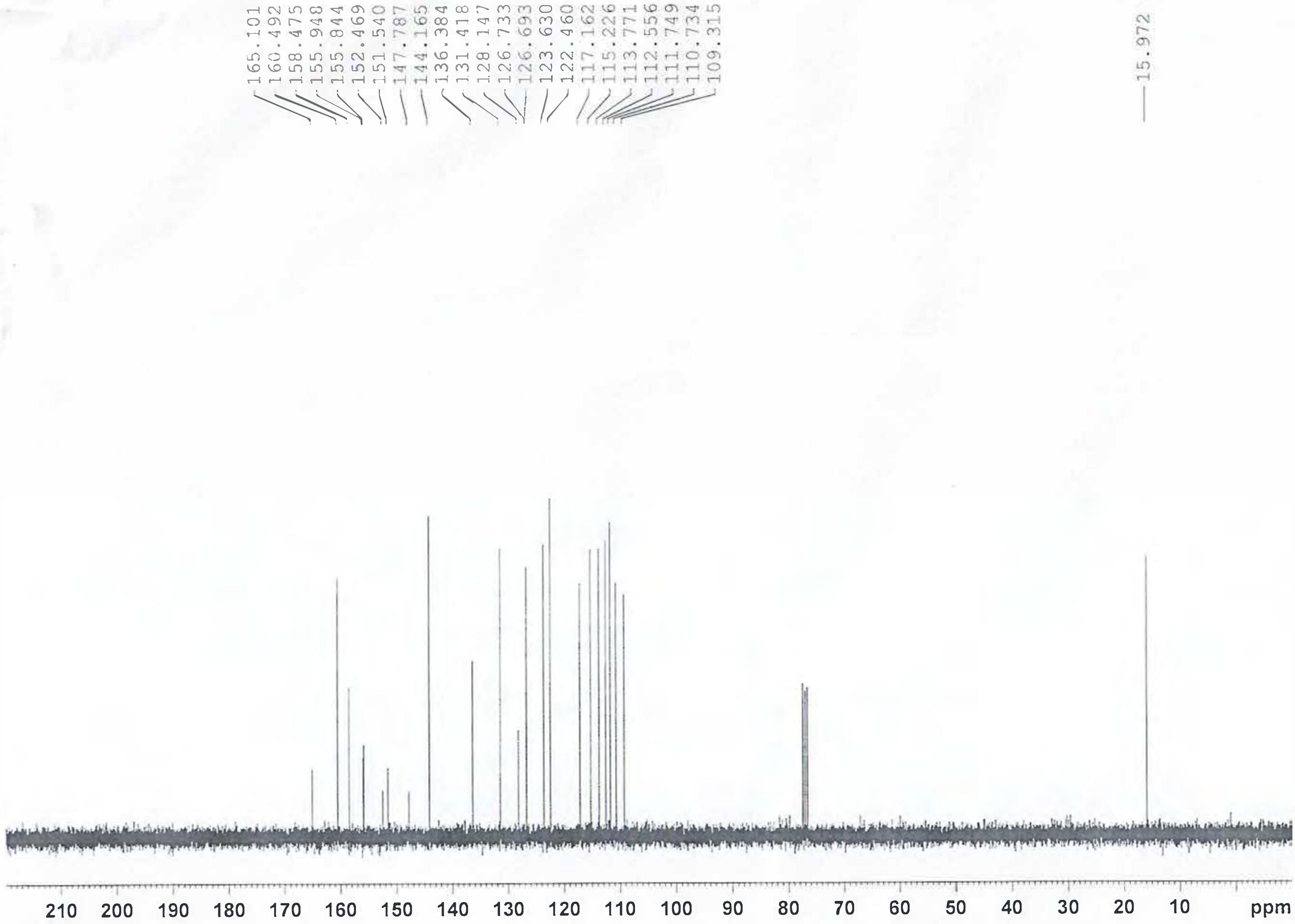

E3

SP20130621-2 CDCL3 1HNMR AV300

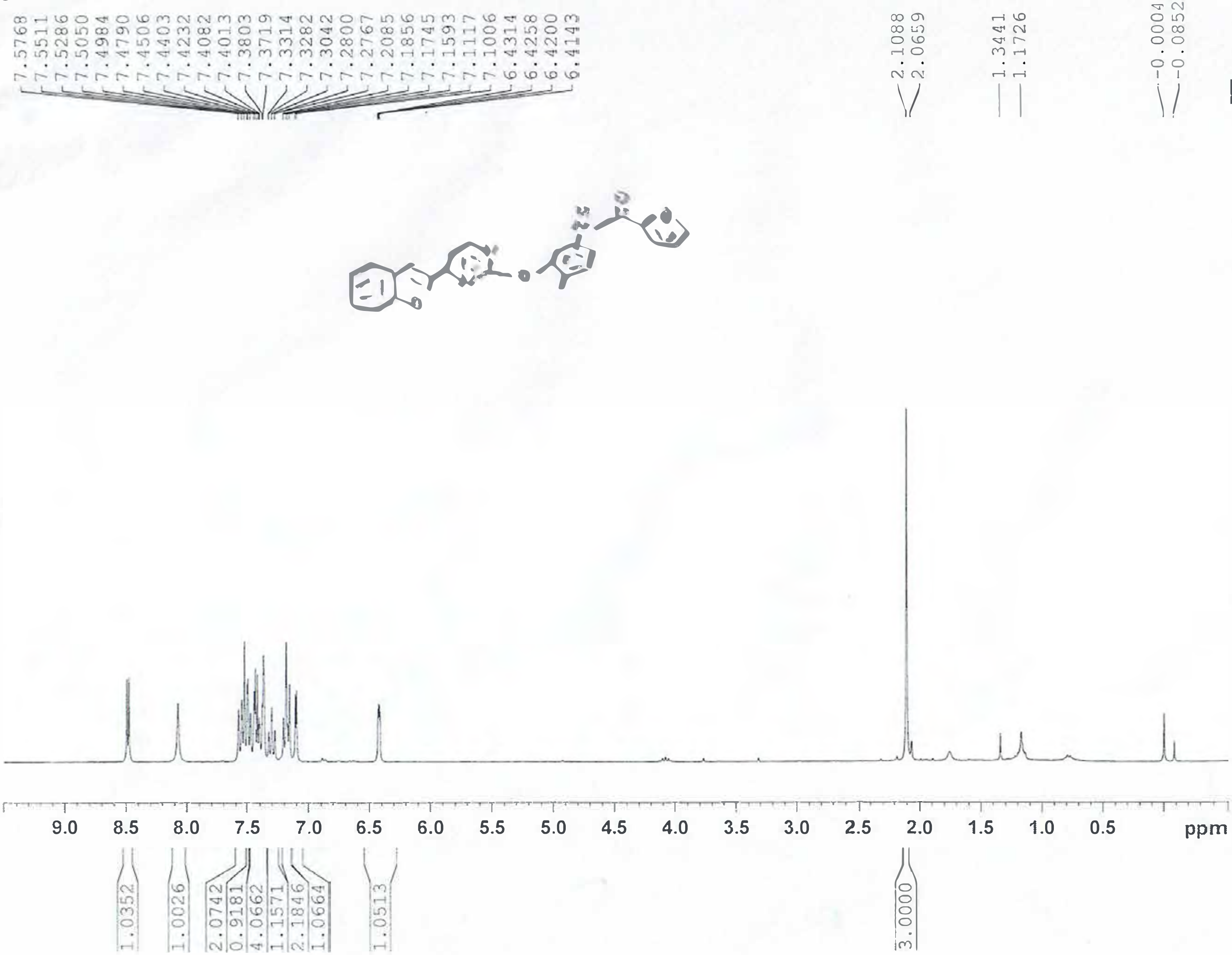

**Sample Name**		**Position**	p1B9	**Instrument Name**	Instrument 1
**User Name**	QTOF-PC\QTOF	**Inj Vol**	0.1	**InjPosition**	
**Sample Type**	Sample	**IRM Calibration Status**	Success	**Data Filename**	E3-p.d
**ACQ Method**	20110418-MSonly-p.m	**Comment**		**Acquired Time**	3/12/2018 4:33:55 PM

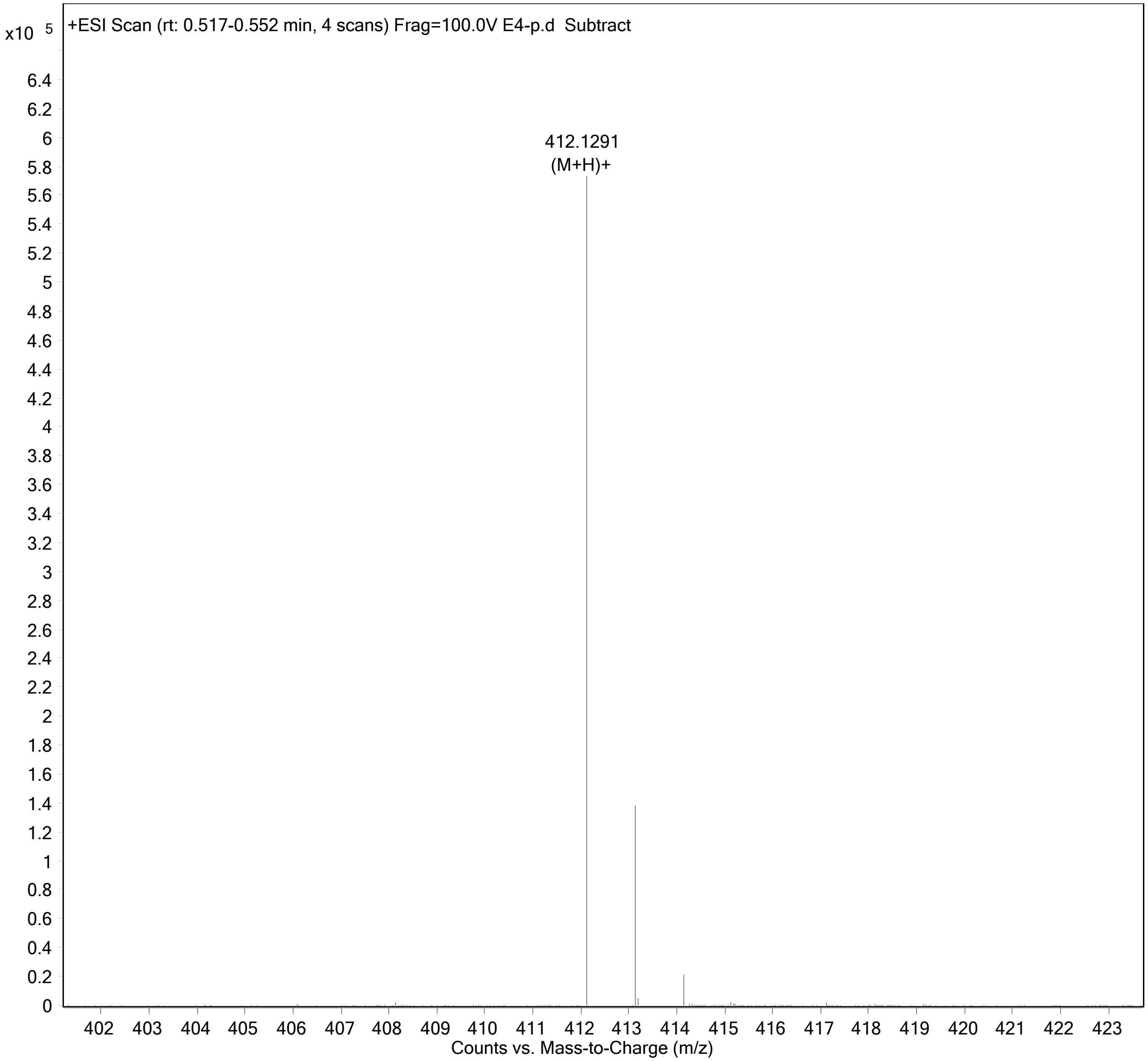

E4

SP130624-C C13-NMR cdcl3 303K AV-300

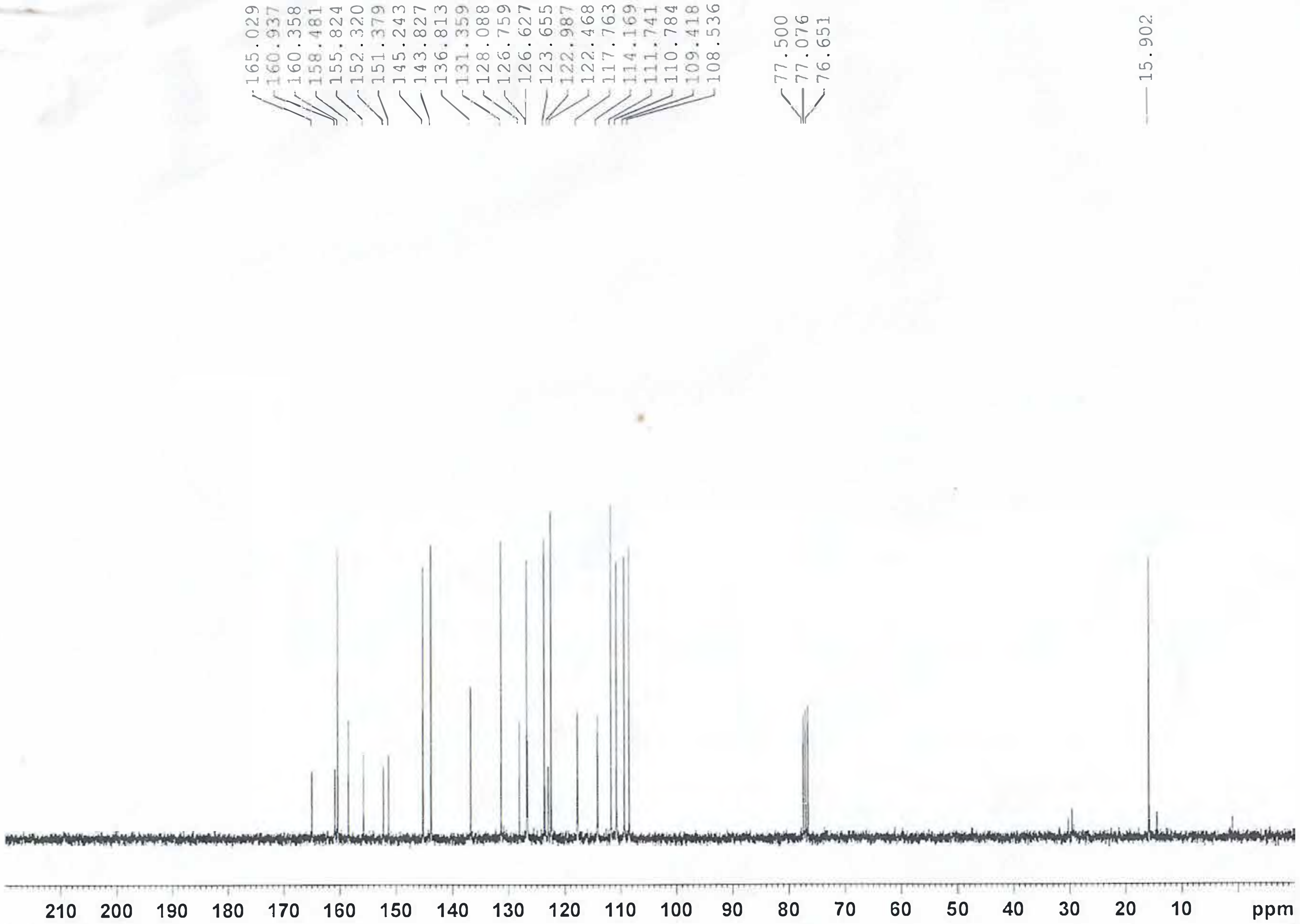

E4

SP20130624 CDCL3 1HNMR AV300

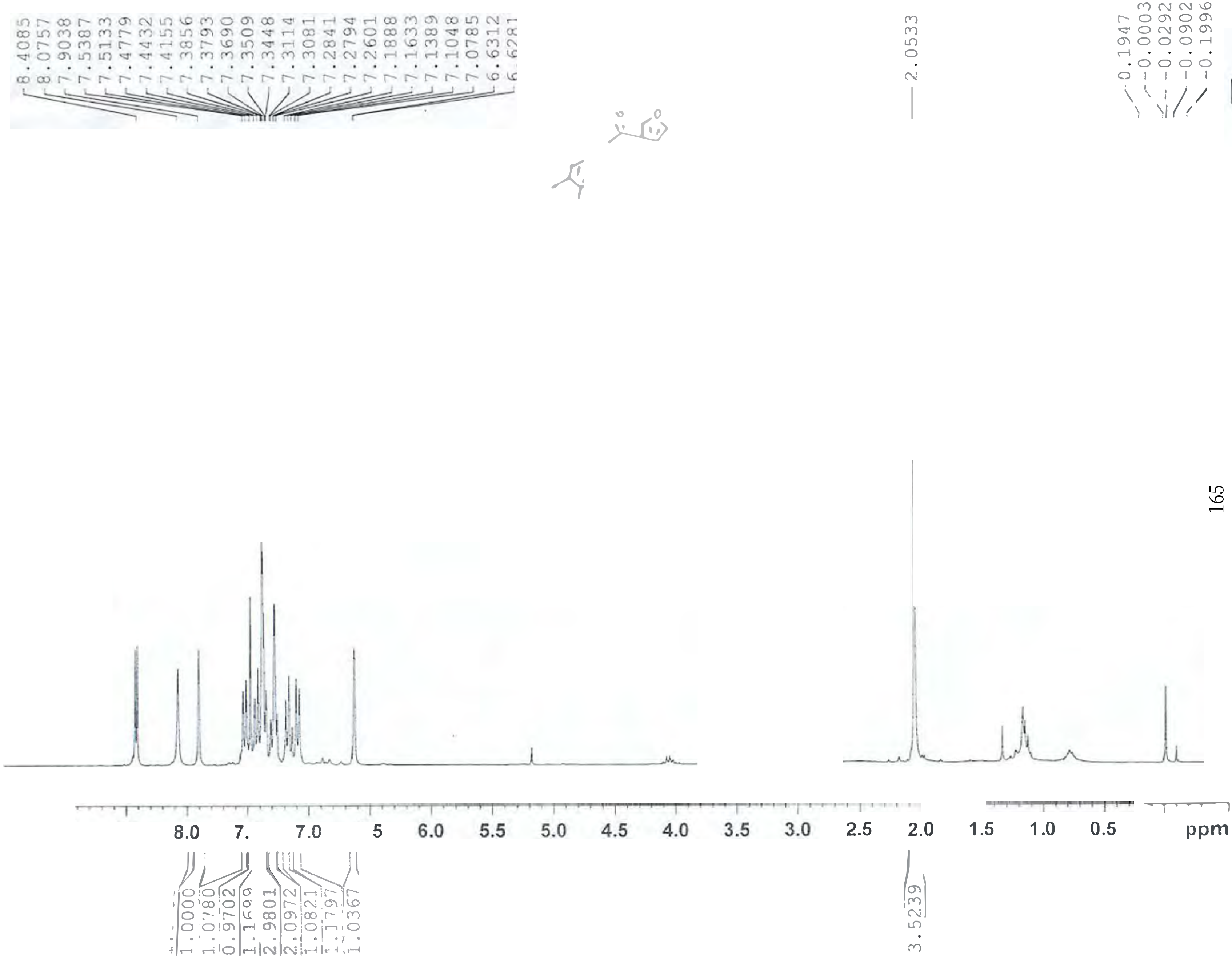

**Sample Name**		**Position**	P1C1	**Instrument Name**	Instrument 1
**User Name**	QTOF-PC\QTOF	**Inj Vol**	0.1	**InjPosition**	
**Sample Type**	Sample	**IRM Calibration Status**	Success	**Data Filename**	E4-p.d
**ACQ Method**	20110418-MSonly-p.m	**Comment**		**Acquired Time**	3/12/2018 4:36:07 PM

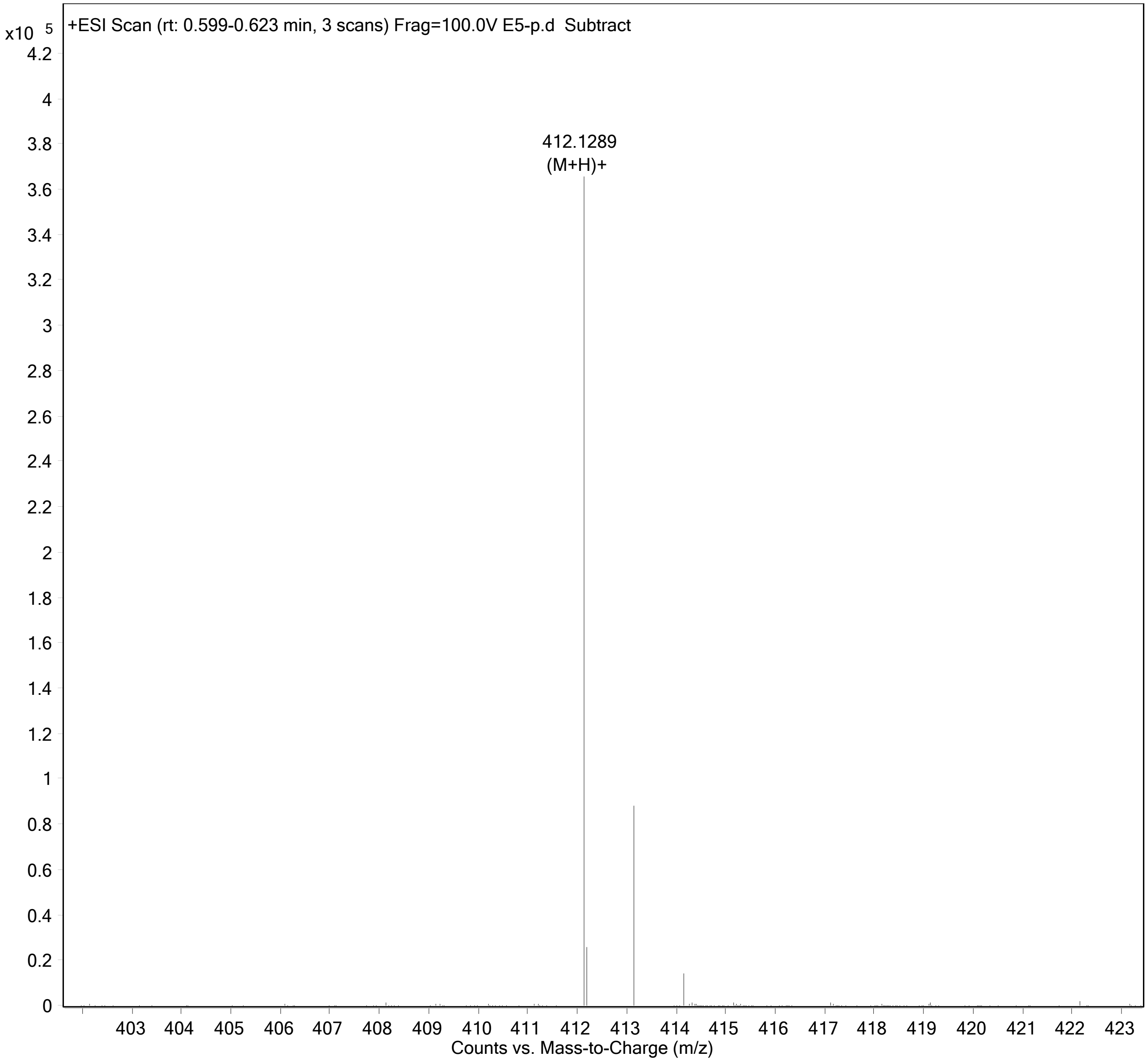

E5

SP131021-C C13-NMR CDCL3 303K AV-300

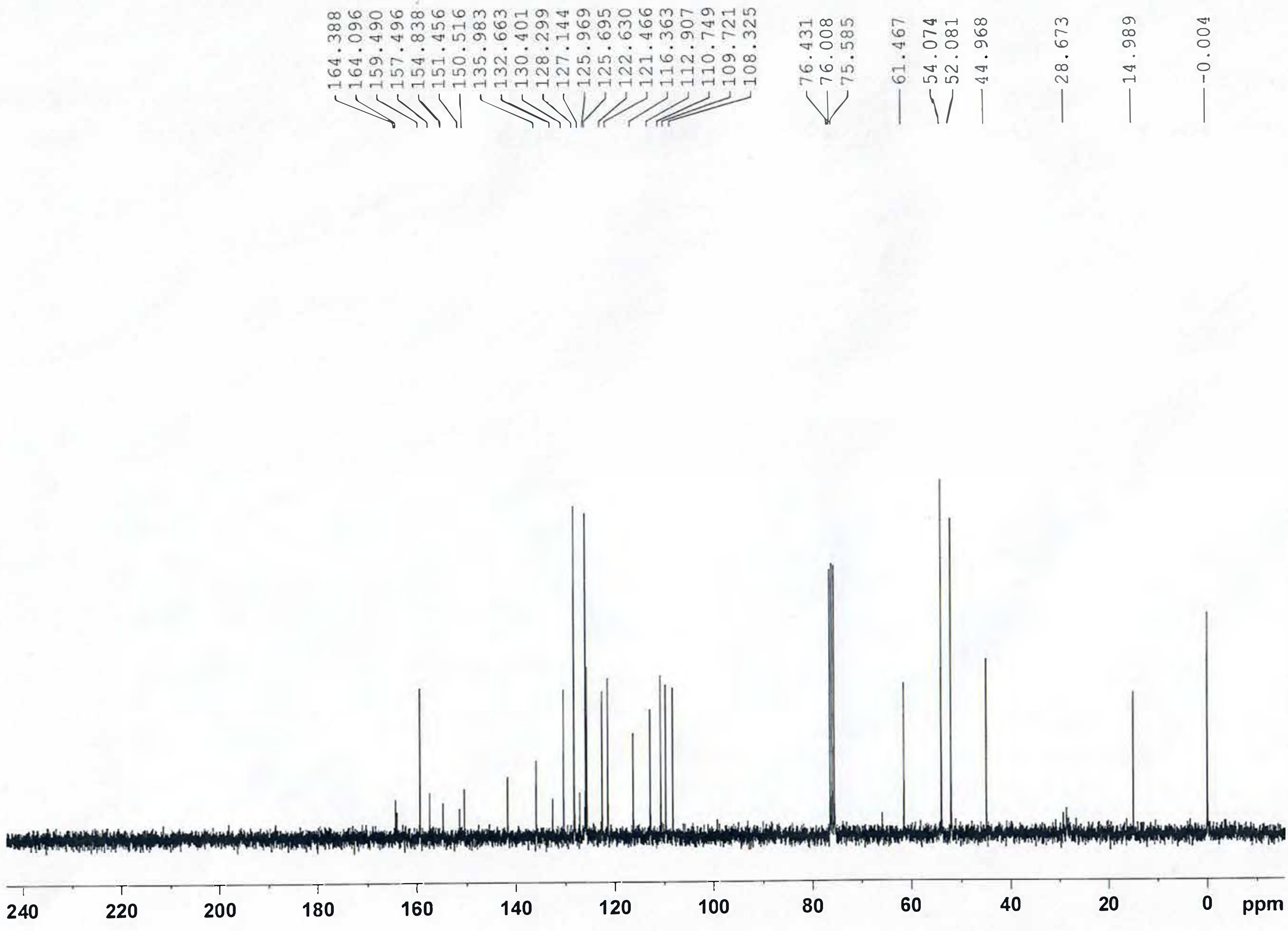

E5

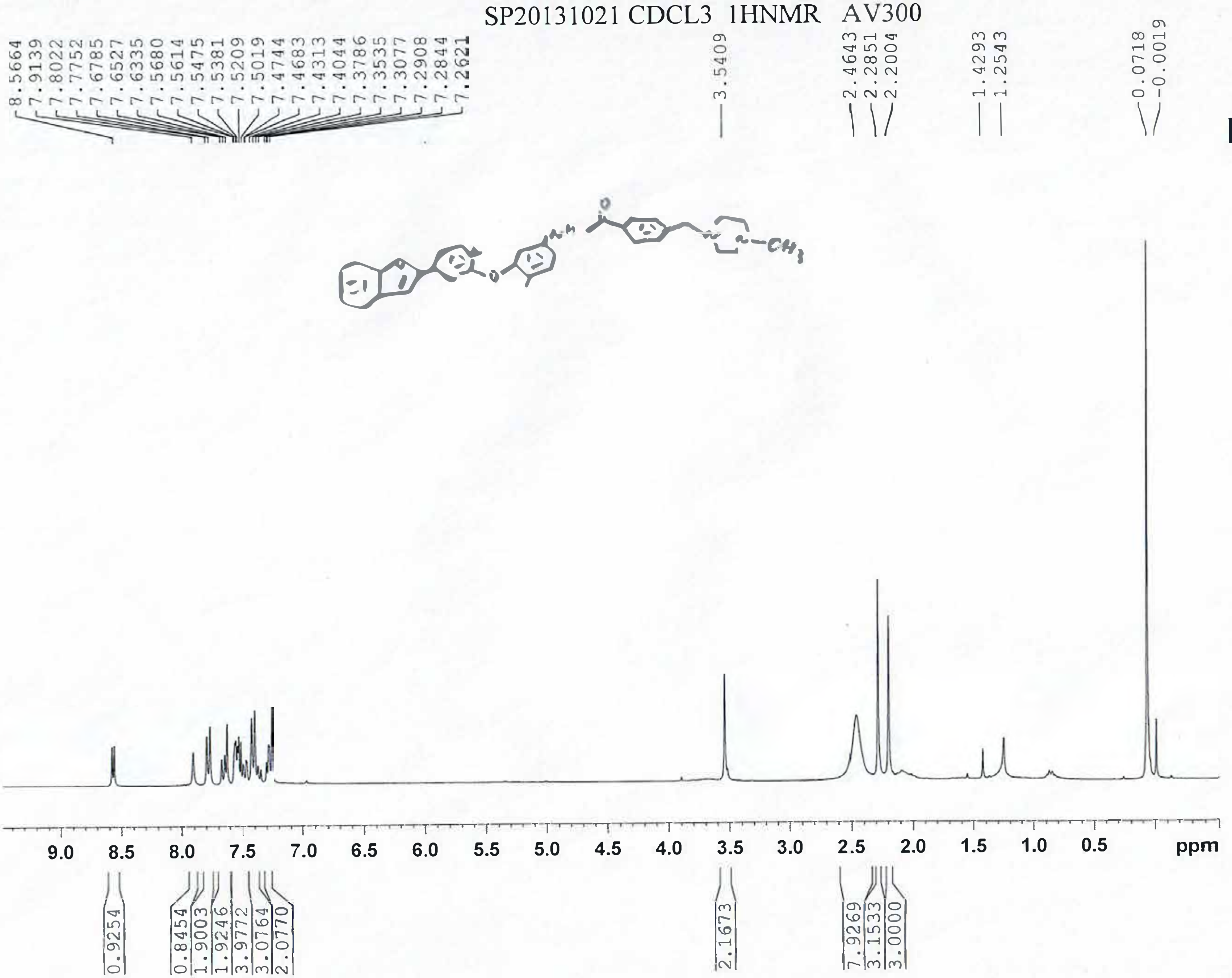
SP20131021 CDCL3 1HNMR AV300
8.5664
7.9139
7.8022
7.7752
7.6785
7.6527
7.6335
7.5680
7.5614
7.5475
7.5381
7.5209
7.5019
7.4744
7.4683
7.4313
7.4044
7.3786
7.3535
7.3077
7.2908
7.2844
7.2621
3.5409
2.4643
2.2851
2.2004
1.4293
1.2543
0.0718
-0.0019
9.0
8.5
8.0
7.5
7.0
6.5
6.0
5.5
5.0
4.5
4.0
3.5
3.0
2.5
2.0
1.5
1.0
0.5
ppm
0.9254
0.8454
1.9003
1.9246
3.9772
3.0764
2.0770
2.1673
7.9269
3.1533
3.0000

**Sample Name**		**Position**	P2a5	**Instrument Name**	Instrument 1
**User Name**	QTOF-PC\QTOF	**Inj Vol**	0.1	**InjPosition**	
**Sample Type**	Sample	**IRM Calibration Status**	Success	**Data Filename**	E5-p.d
**ACQ Method**	20110418-MSonly-p.m	**Comment**		**Acquired Time**	3/13/2018 1:50:42 PM

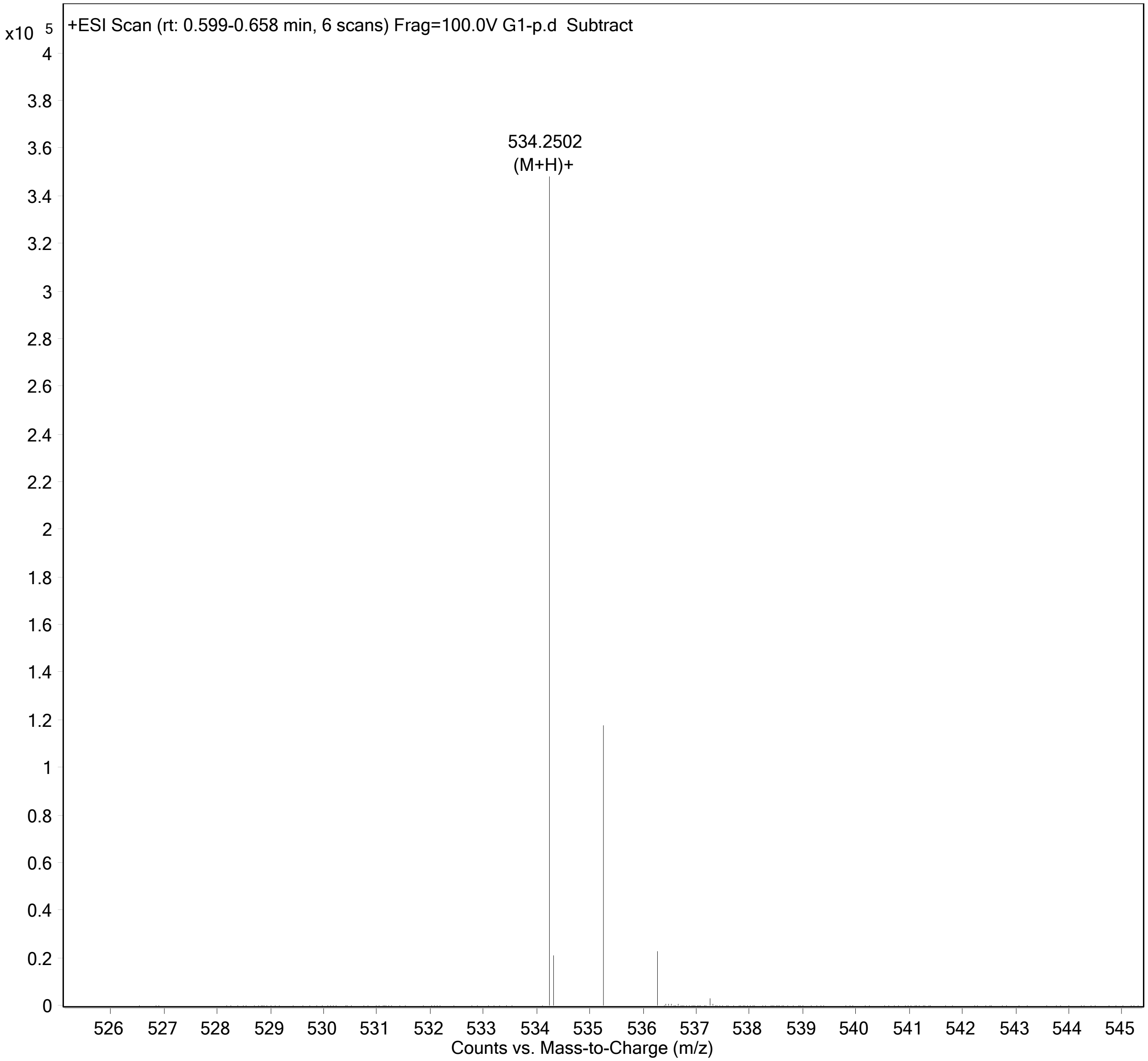

E6

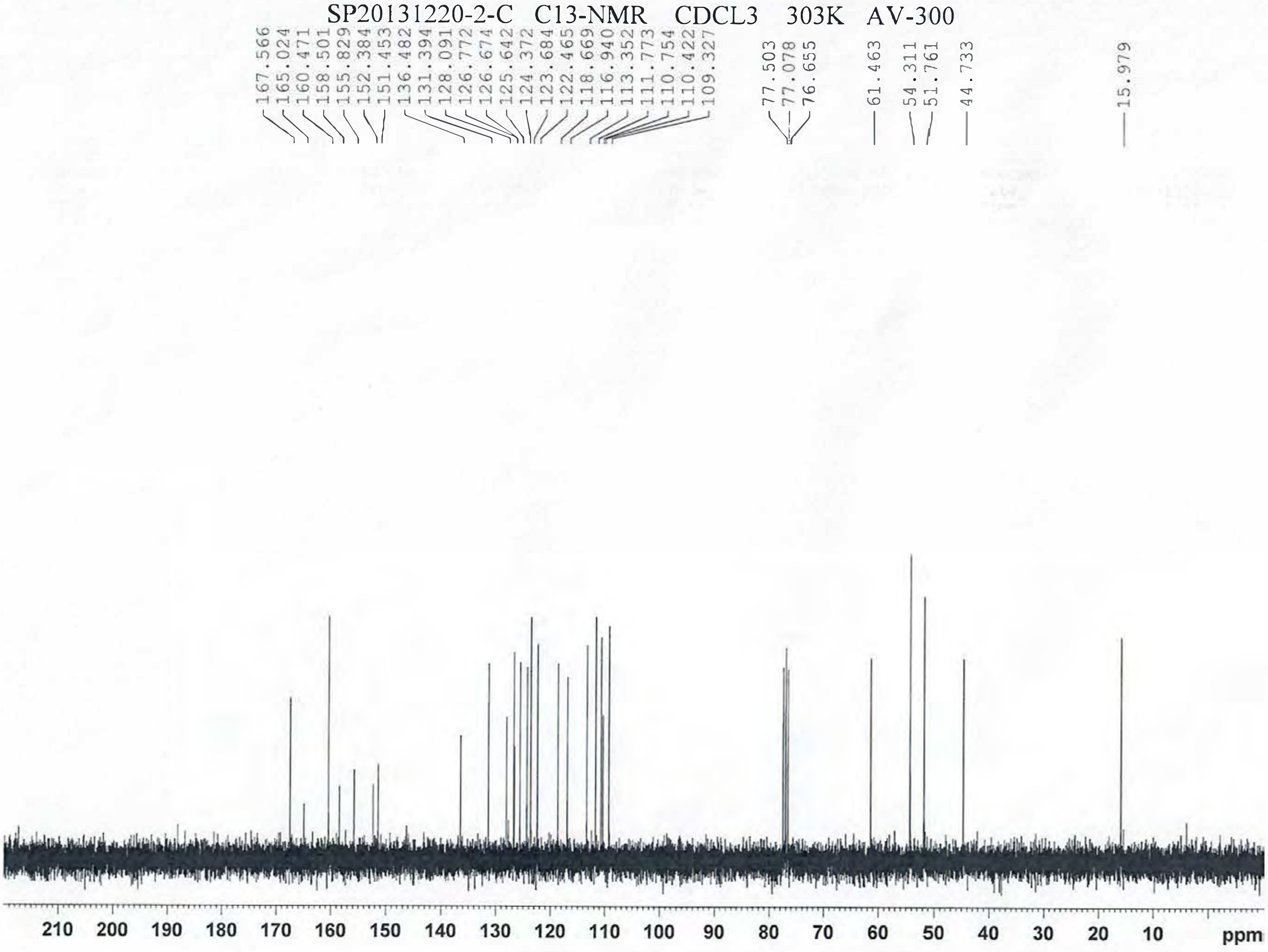

SP20131220-2-C C13-NMR CDCL3 303K AV-300
167.566
165.024
160.471
158.501
155.829
152.384
151.453
136.482
131.394
128.091
126.772
126.674
125.642
124.372
123.684
122.465
118.669
116.940
113.352
111.773
110.754
110.422
109.327
77.503
77.078
76.655
61.463
54.311
51.761
44.733
15.979
210 200 190 180 170 160 150 140 130 120 110 100 90 80 70 60 50 40 30 20 10 ppm

E6

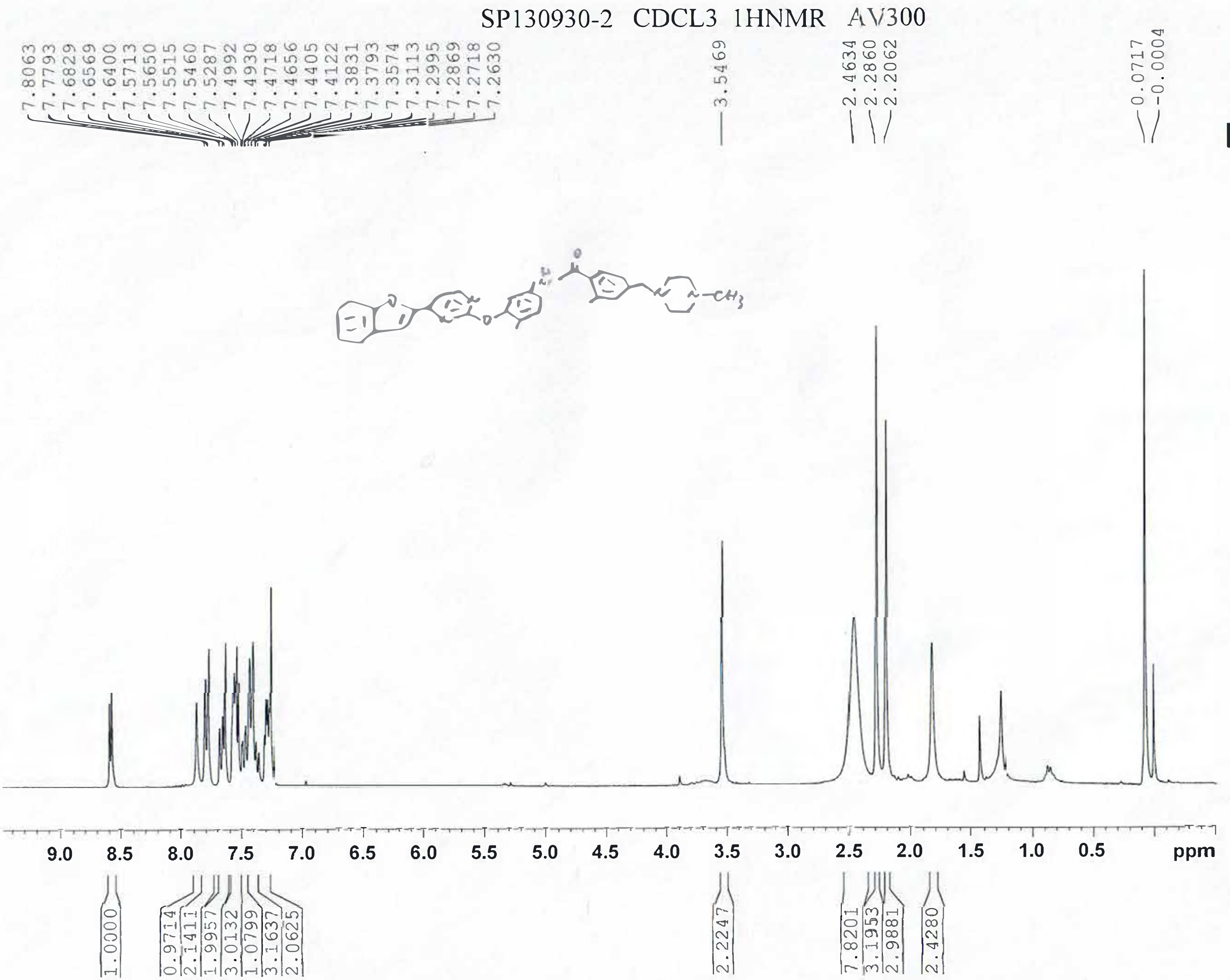

SP130930-2 CDCL3 1HNMR AV300
7.8063
7.7793
7.6829
7.6569
7.6400
7.5713
7.5650
7.5515
7.5460
7.5287
7.4992
7.4930
7.4718
7.4656
7.4405
7.4122
7.3831
7.3793
7.3574
7.3113
7.2995
7.2869
7.2718
7.2630
3.5469
2.4634
2.2860
2.2062
0.0717
-0.0004
9.0
8.5
8.0
7.5
7.0
6.5
6.0
5.5
5.0
4.5
4.0
3.5
3.0
2.5
2.0
1.5
1.0
0.5
ppm
1.0000
0.9714
2.1411
1.9957
3.0132
1.0799
3.1637
2.0625
2.2247
7.8201
3.1953
2.9881
2.4280

E7

SP-20131011 CDCL3 1HNMR AV300

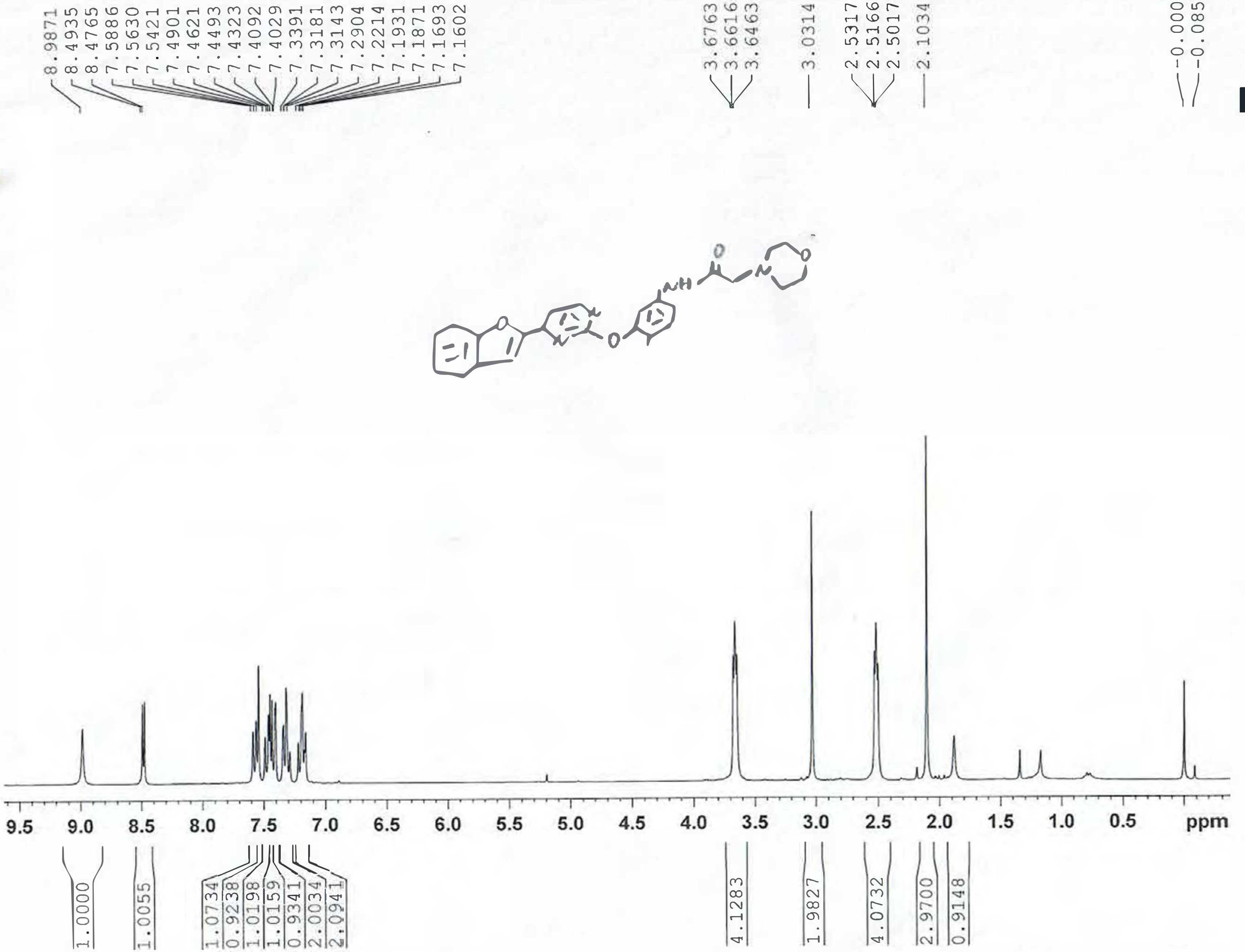

E7

SP20131018-C C13-NMR cdcl3 303K AV-300

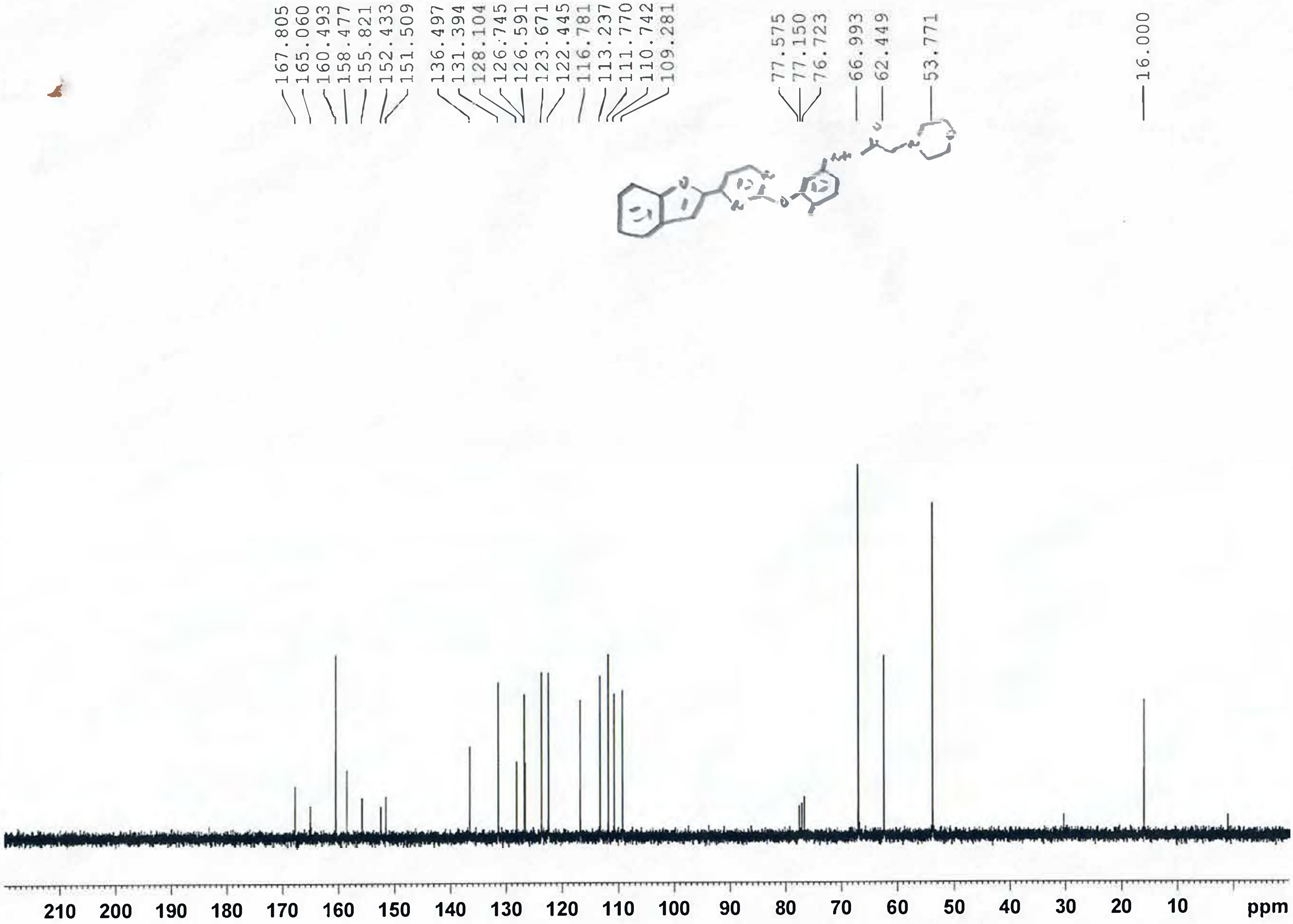

**Sample Name**		**Position**	P2a6	**Instrument Name**	Instrument 1
**User Name**	QTOF-PC\QTOF	**Inj Vol**	0.1	**InjPosition**	
**Sample Type**	Sample	**IRM Calibration Status**	Success	**Data Filename**	E7-p.d
**ACQ Method**	20110418-MSonly-p.m	**Comment**		**Acquired Time**	3/13/2018 1:53:04 PM

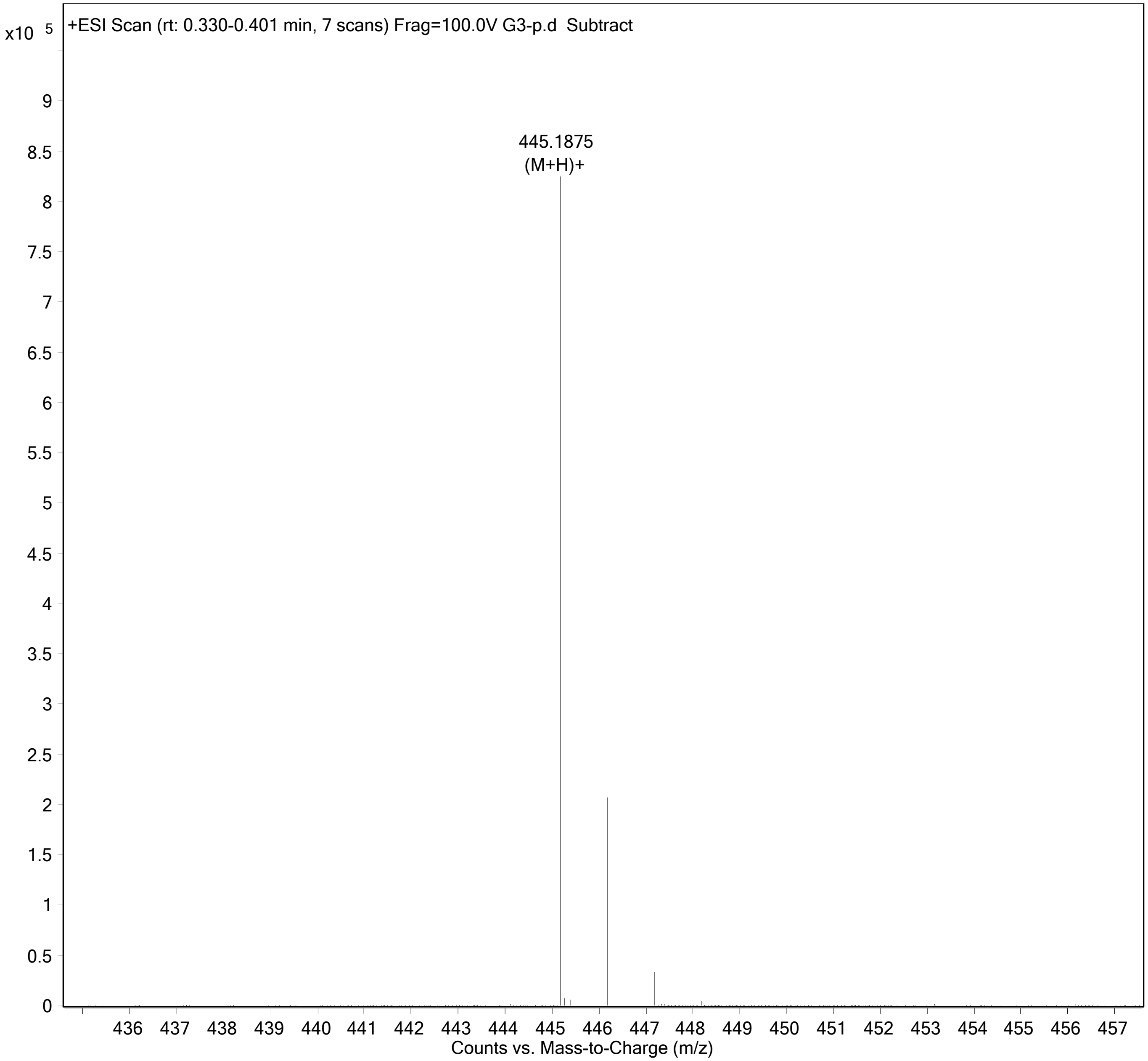

E8

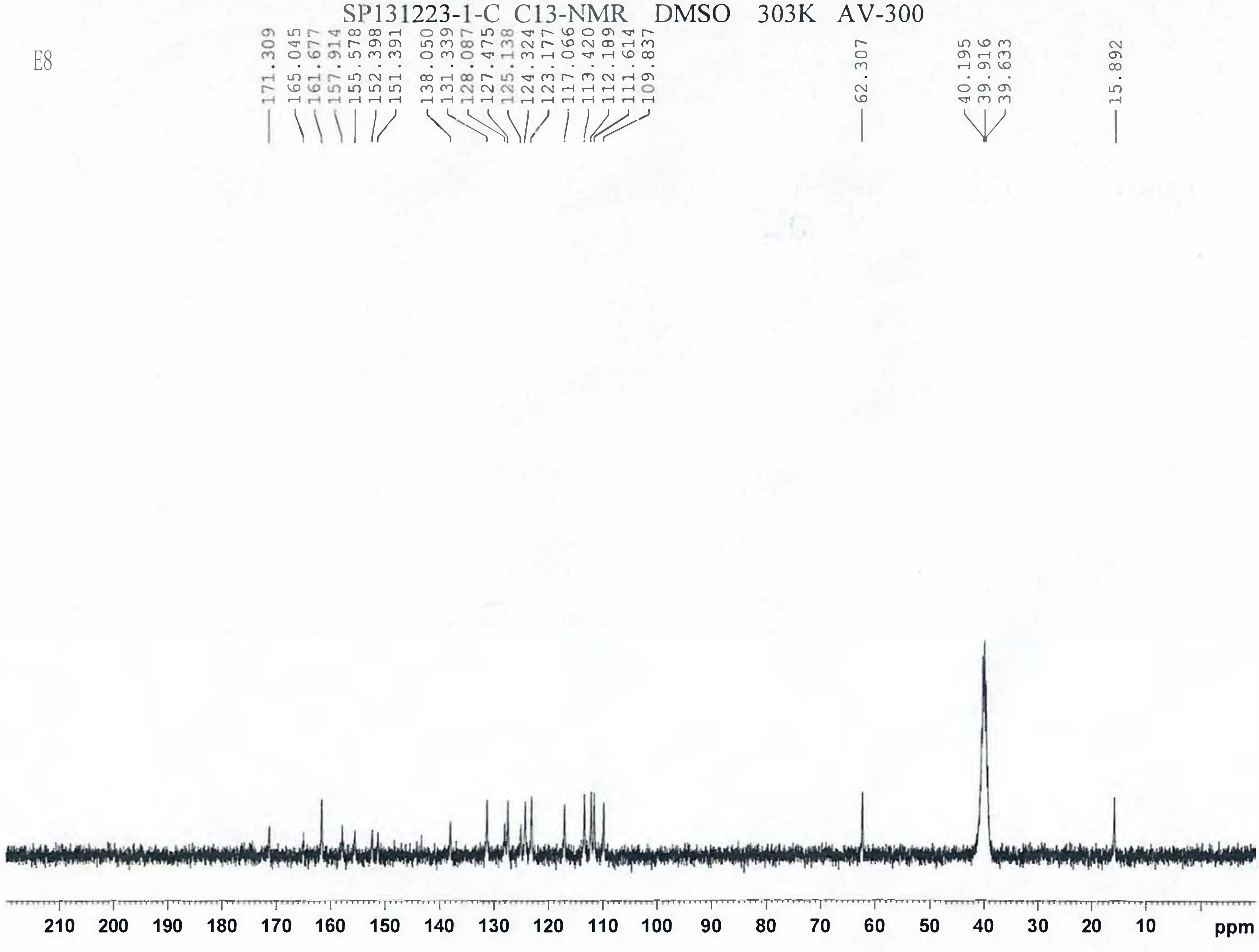
SP131223-1-C C13-NMR DMSO 303K AV-300
171.309
165.045
161.677
157.914
155.578
152.398
151.391
138.050
131.339
128.087
127.475
125.138
124.324
123.177
117.066
113.420
112.189
111.614
109.837
62.307
40.195
39.916
39.633
15.892
210 200 190 180 170 160 150 140 130 120 110 100 90 80 70 60 50 40 30 20 10 ppm

E8

SP130930-3 CDCL3 1HNMR AV300

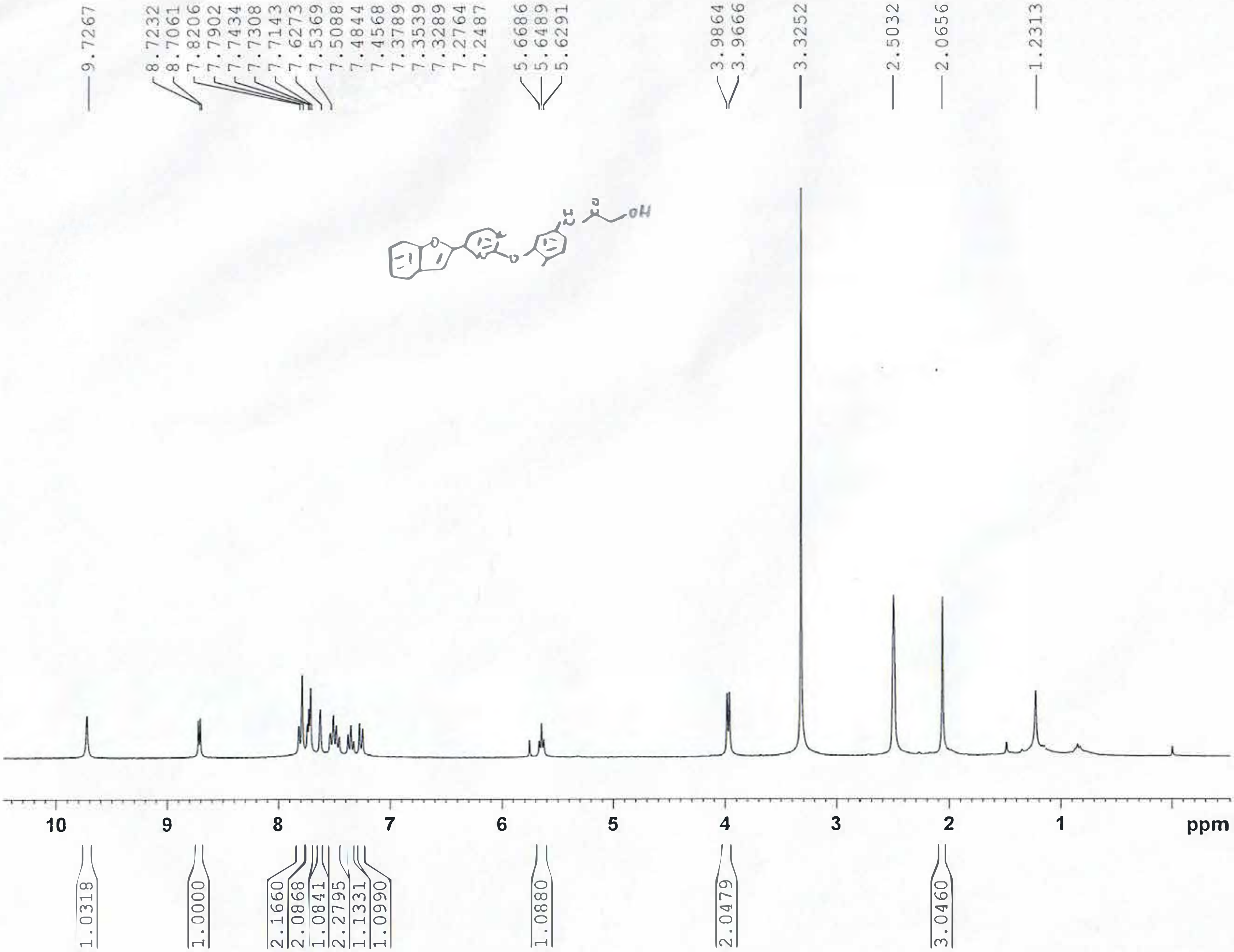

**Sample Name**		**Position**	P2a7	**Instrument Name**	Instrument 1
**User Name**	QTOF-PC\QTOF	**Inj Vol**	0.1	**InjPosition**	
**Sample Type**	Sample	**IRM Calibration Status**	Success	**Data Filename**	E8-p.d
**ACQ Method**	20110418-MSonly-p.m	**Comment**		**Acquired Time**	3/13/2018 1:56:22 PM

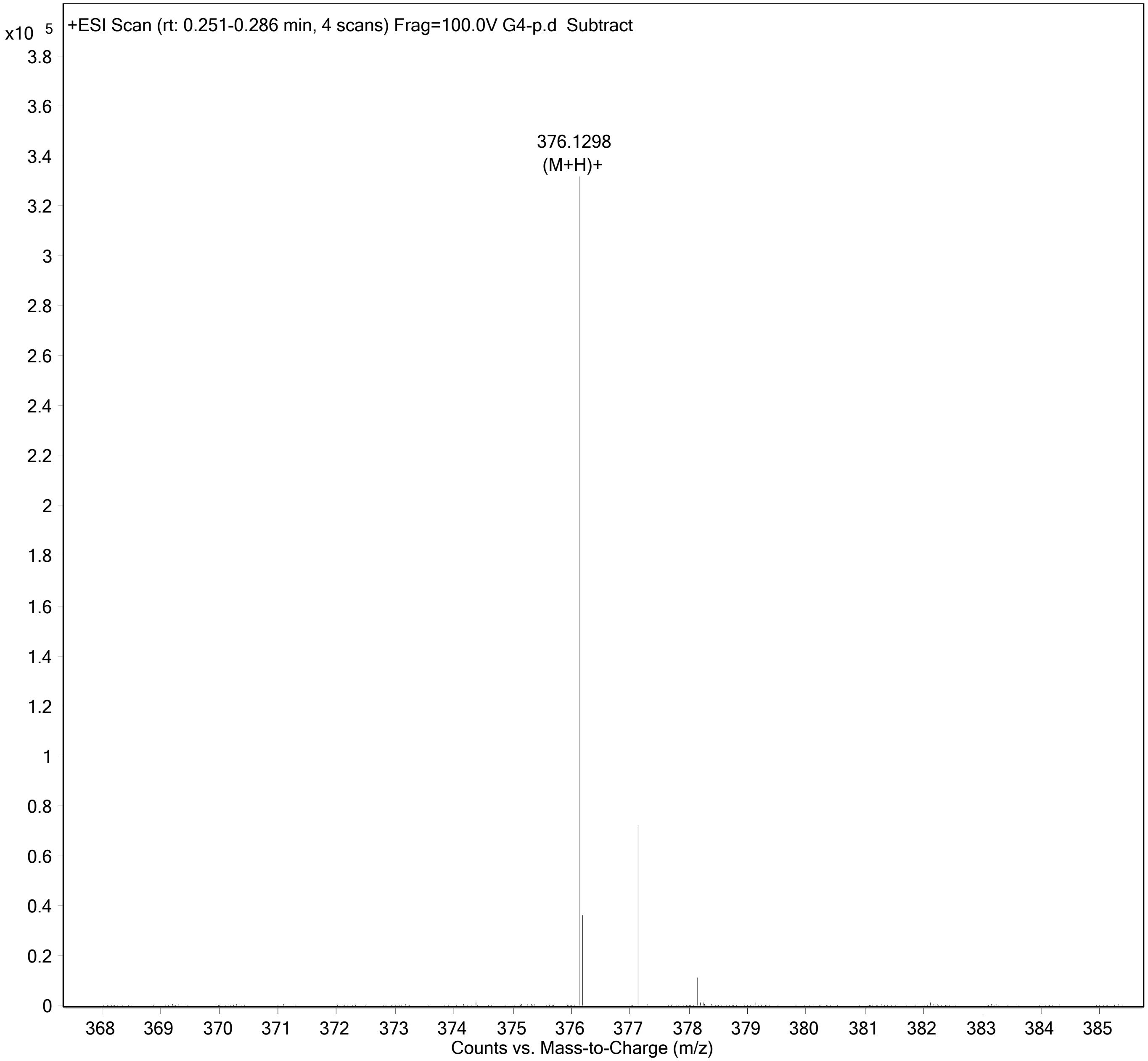

F1

SP1300701-C C13-NMR cdcl3 303K AV-300

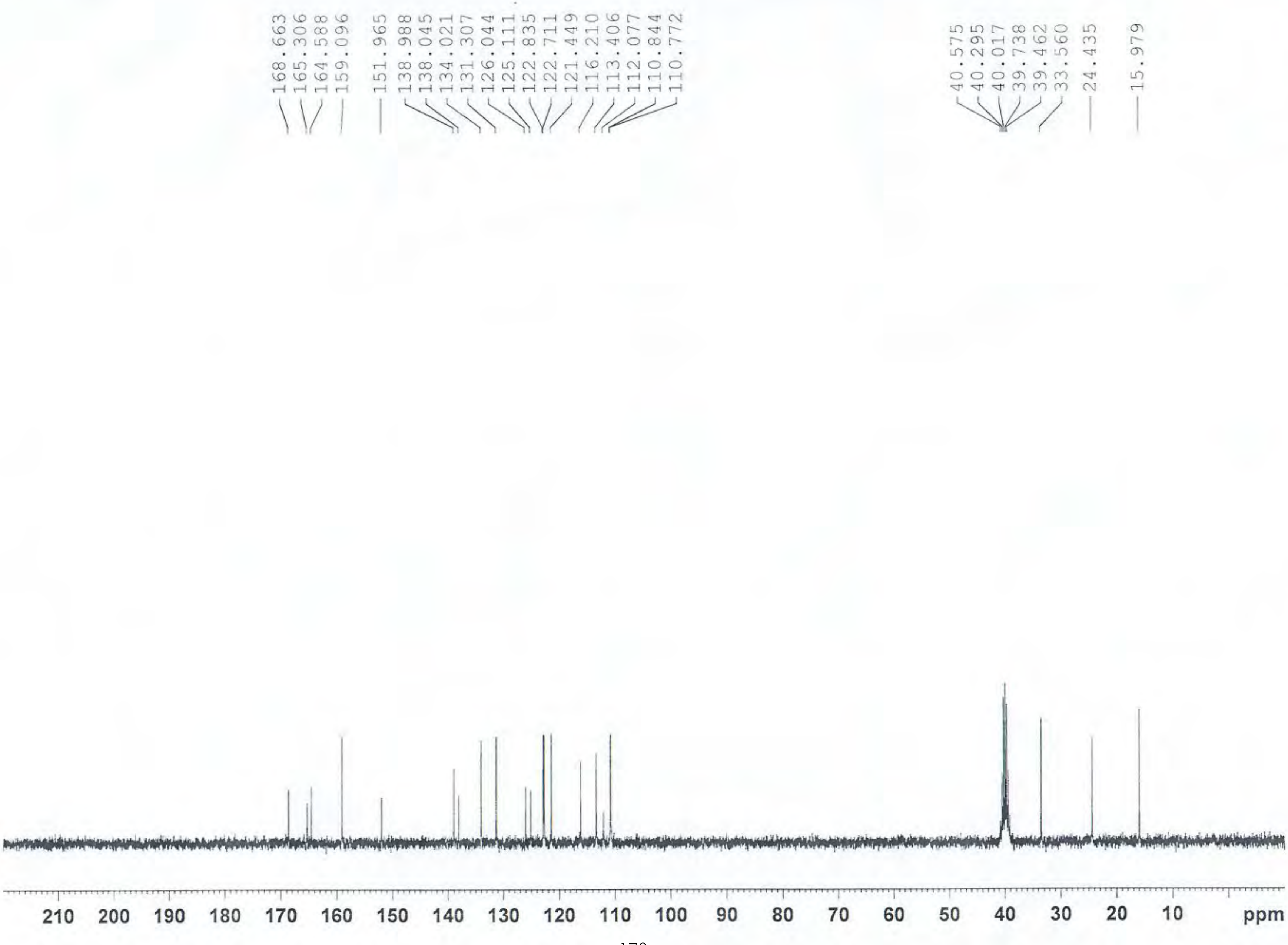

F1

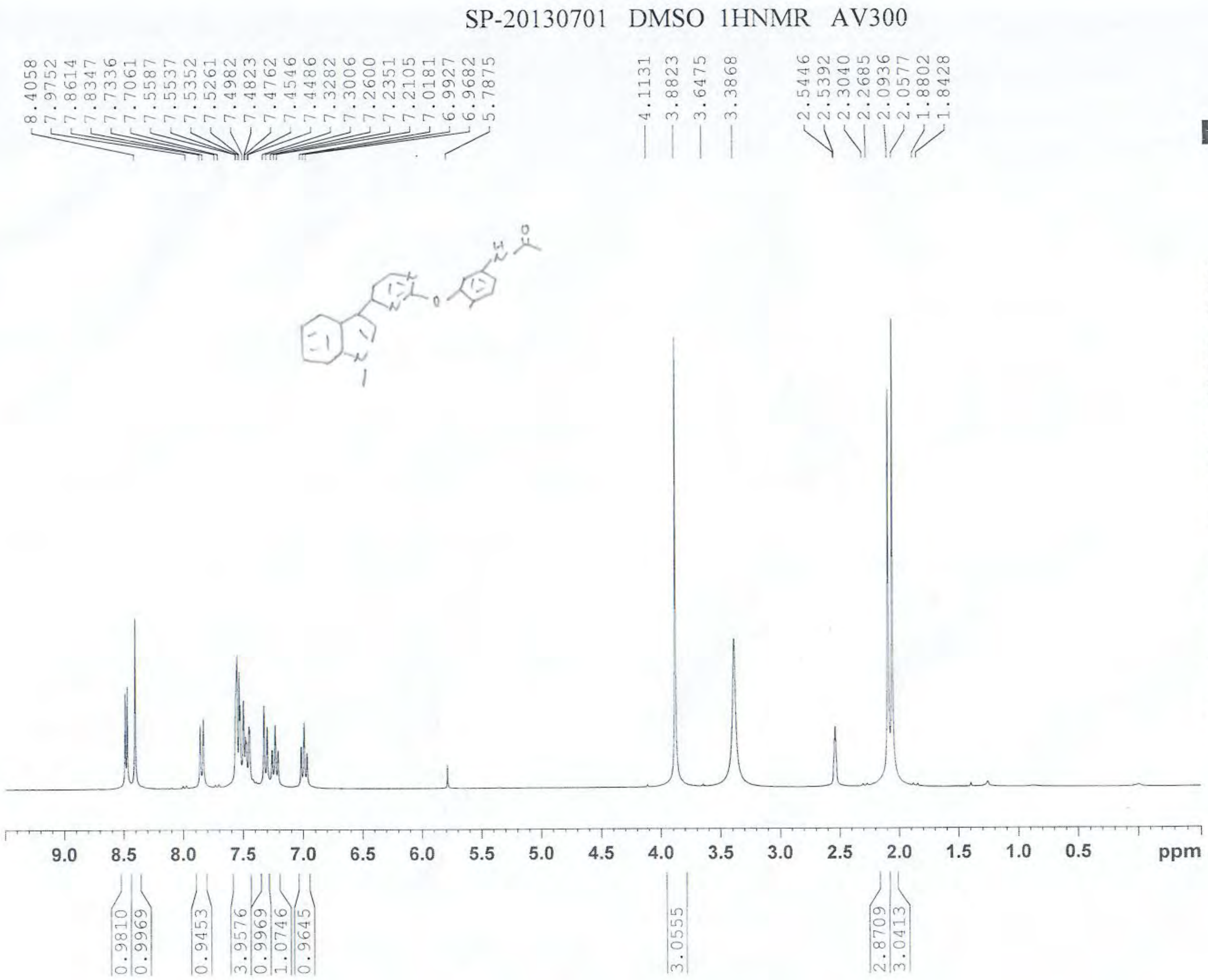
SP-20130701 DMSO 1HNMR AV300
8.4058
7.9752
7.8614
7.8347
7.7336
7.7061
7.5587
7.5537
7.5352
7.5261
7.4982
7.4823
7.4762
7.4546
7.4486
7.3282
7.3006
7.2600
7.2351
7.2105
7.0181
6.9927
6.9682
5.7875
4.1131
3.8823
3.6475
3.3868
2.5446
2.5392
2.3040
2.2685
2.0936
2.0577
1.8802
1.8428
9.0 8.5 8.0 7.5 7.0 6.5 6.0 5.5 5.0 4.5 4.0 3.5 3.0 2.5 2.0 1.5 1.0 0.5 ppm
0.9810
0.9969
0.9453
3.9576
0.9969
1.0746
0.9645
3.0555
2.8709
3.0413

**Sample Name**		**Position**	P1C2	**Instrument Name**	Instrument 1
**User Name**	QTOF-PC\QTOF	**Inj Vol**	0.1	**InjPosition**	
**Sample Type**	Sample	**IRM Calibration Status**	Success	**Data Filename**	F1-p.d
**ACQ Method**	20110418-MSonly-p.m	**Comment**		**Acquired Time**	3/12/2018 4:38:05 PM

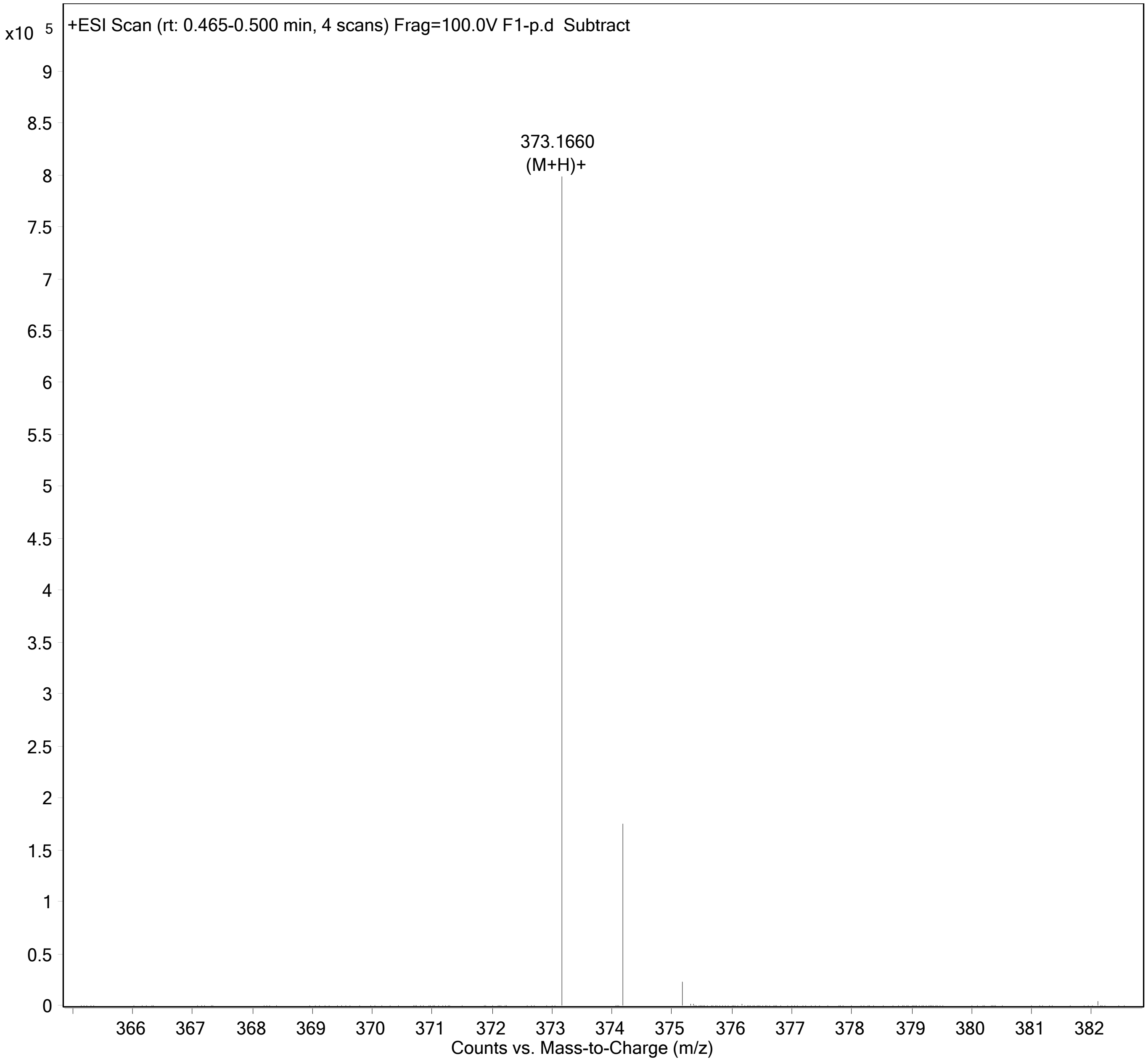

F2

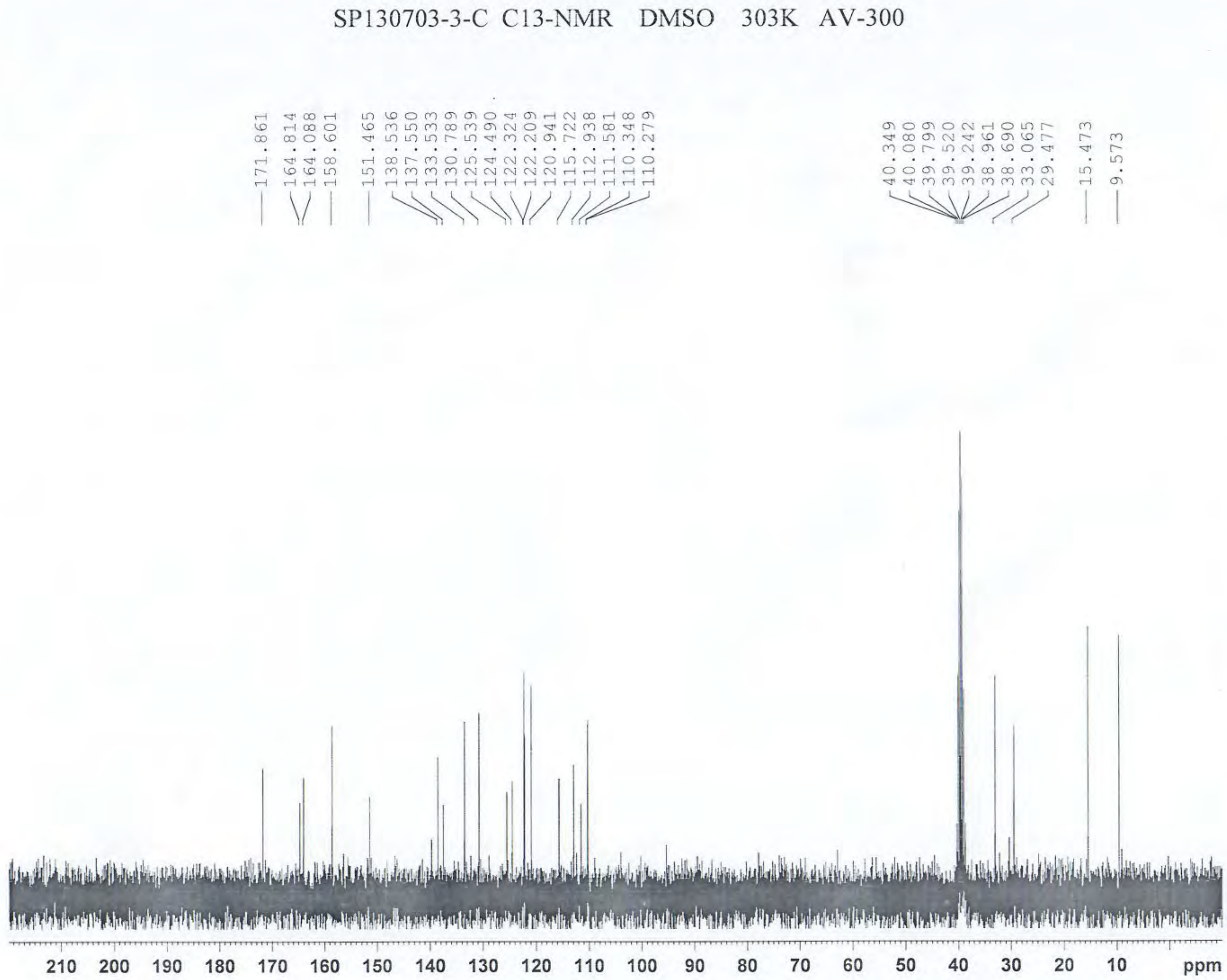
SP130703-3-C C13-NMR DMSO 303K AV-300
171.861
164.814
164.088
158.601
151.465
138.536
137.550
133.533
130.789
125.539
124.490
122.324
122.209
120.941
115.722
112.938
111.581
110.348
110.279
40.349
40.080
39.799
39.520
39.242
38.961
38.690
33.065
29.477
15.473
9.573
210 200 190 180 170 160 150 140 130 120 110 100 90 80 70 60 50 40 30 20 10 ppm

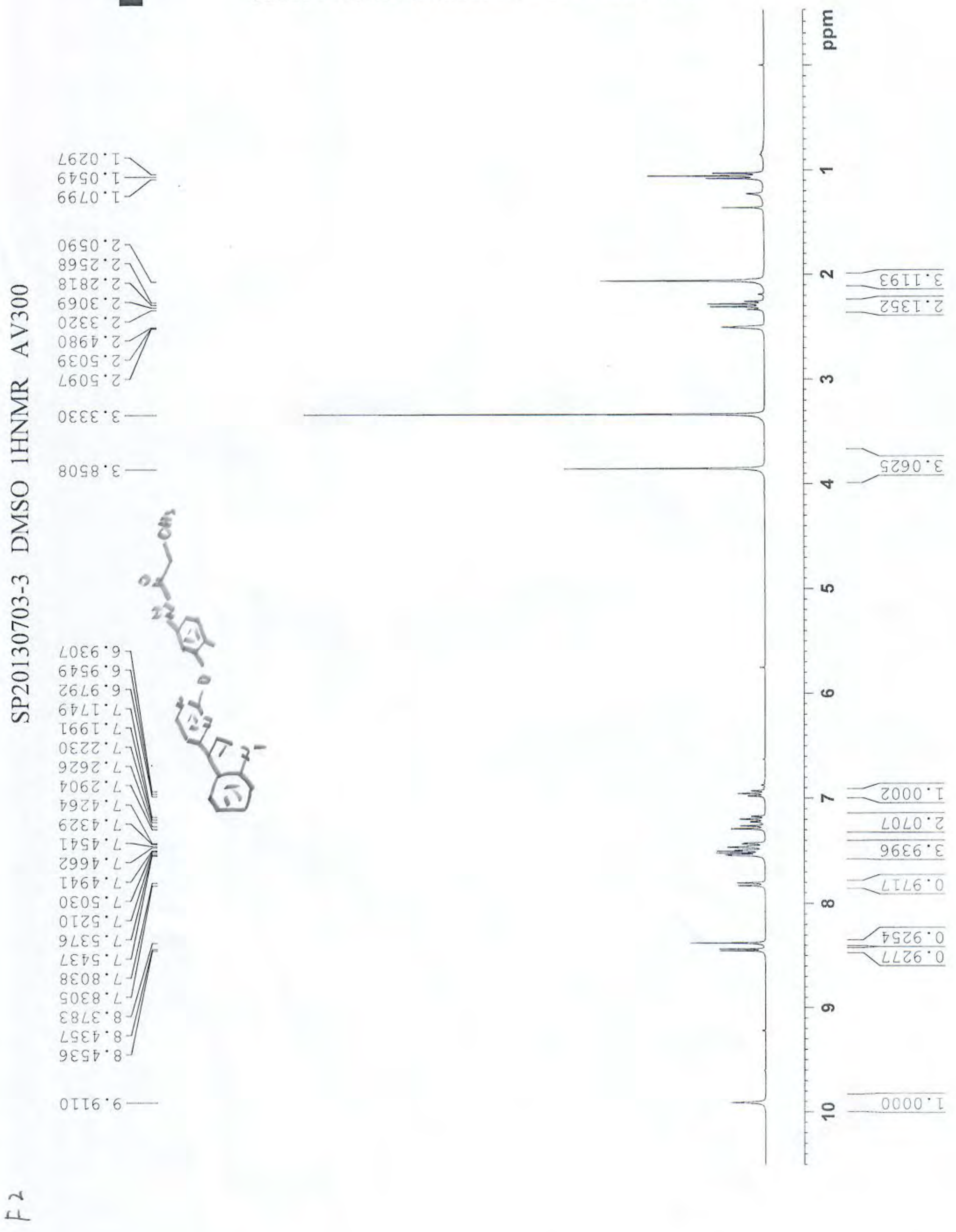
SP201307703-3 DMSO 1HNMR AV300
F2
ppm
1.0297
1.0549
1.0799
2.0590
2.2568
2.2818
2.3069
2.3320
2.4980
2.5039
2.5097
3.3330
3.8508
6.9307
6.9549
6.9792
7.1749
7.1991
7.2230
7.2626
7.2904
7.4264
7.4329
7.4541
7.4662
7.4941
7.5030
7.5210
7.5376
7.5437
7.8038
7.8305
8.3783
8.4357
8.4536
9.9110
3.1193
2.1352
3.0625
1.0002
2.0707
3.9396
0.9717
0.9254
0.9277
1.0000

**Sample Name**		**Position**	P2a1	**Instrument Name**	Instrument 1
**User Name**	QTOF-PC\QTOF	**Inj Vol**	0.1	**InjPosition**	
**Sample Type**	Sample	**IRM Calibration Status**	Success	**Data Filename**	F2-p.d
**ACQ Method**	20110418-MSonly-p.m	**Comment**		**Acquired Time**	3/13/2018 1:42:53 PM

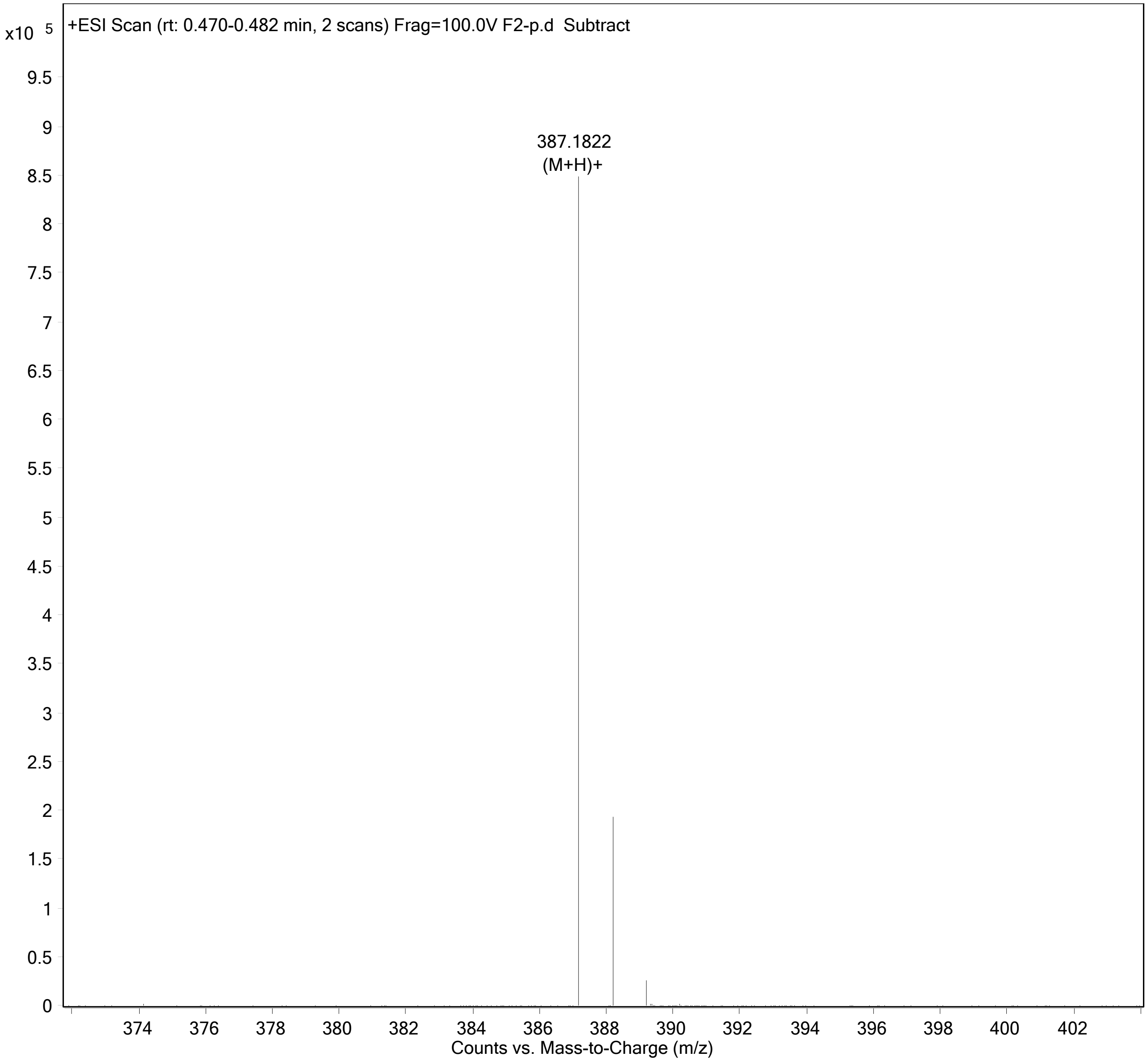

F3

SP130705-2-C C13-NMR DMSO 303K AV-300

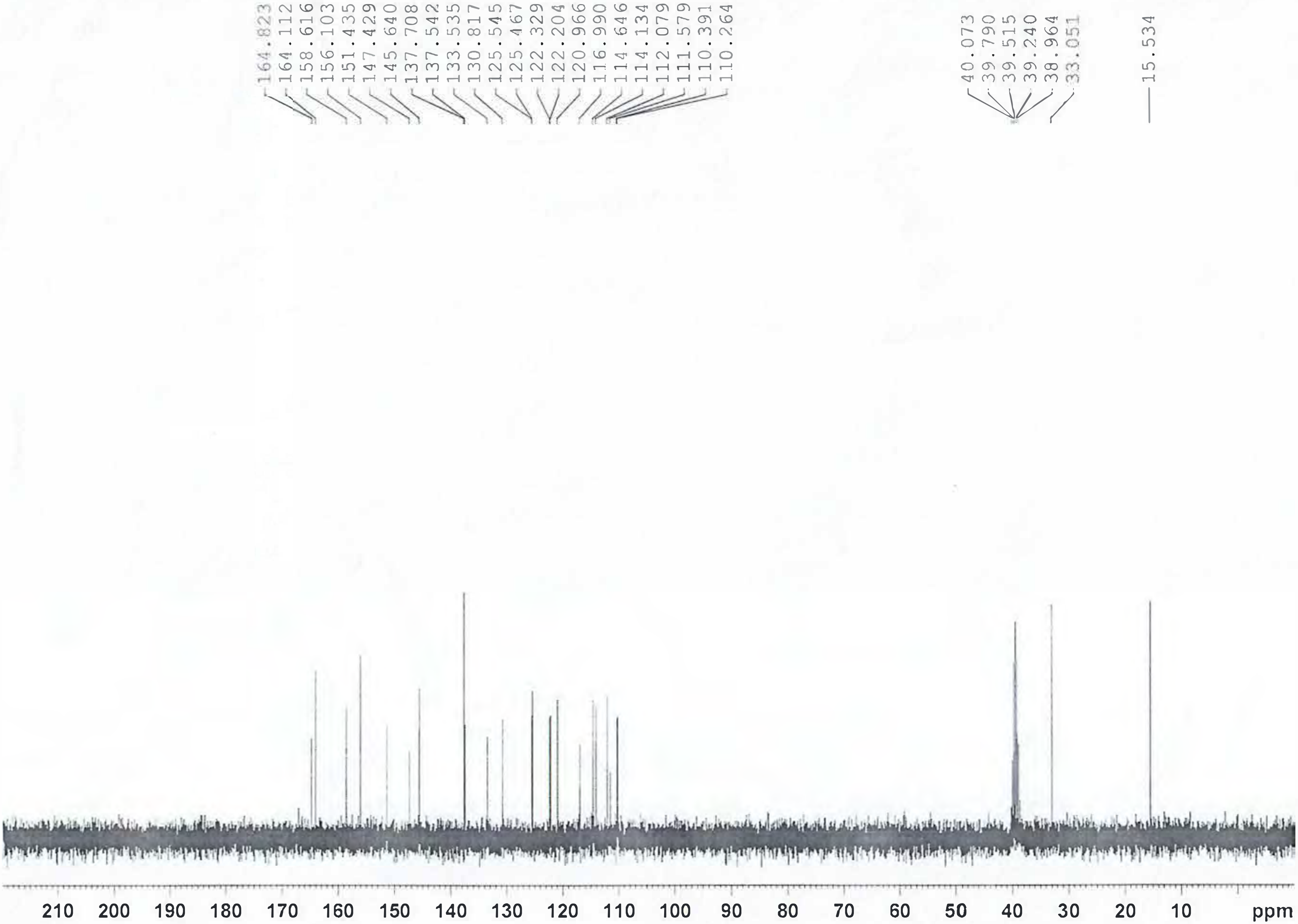

F3

SP20130705-2 DMSO 1HNMR AV300

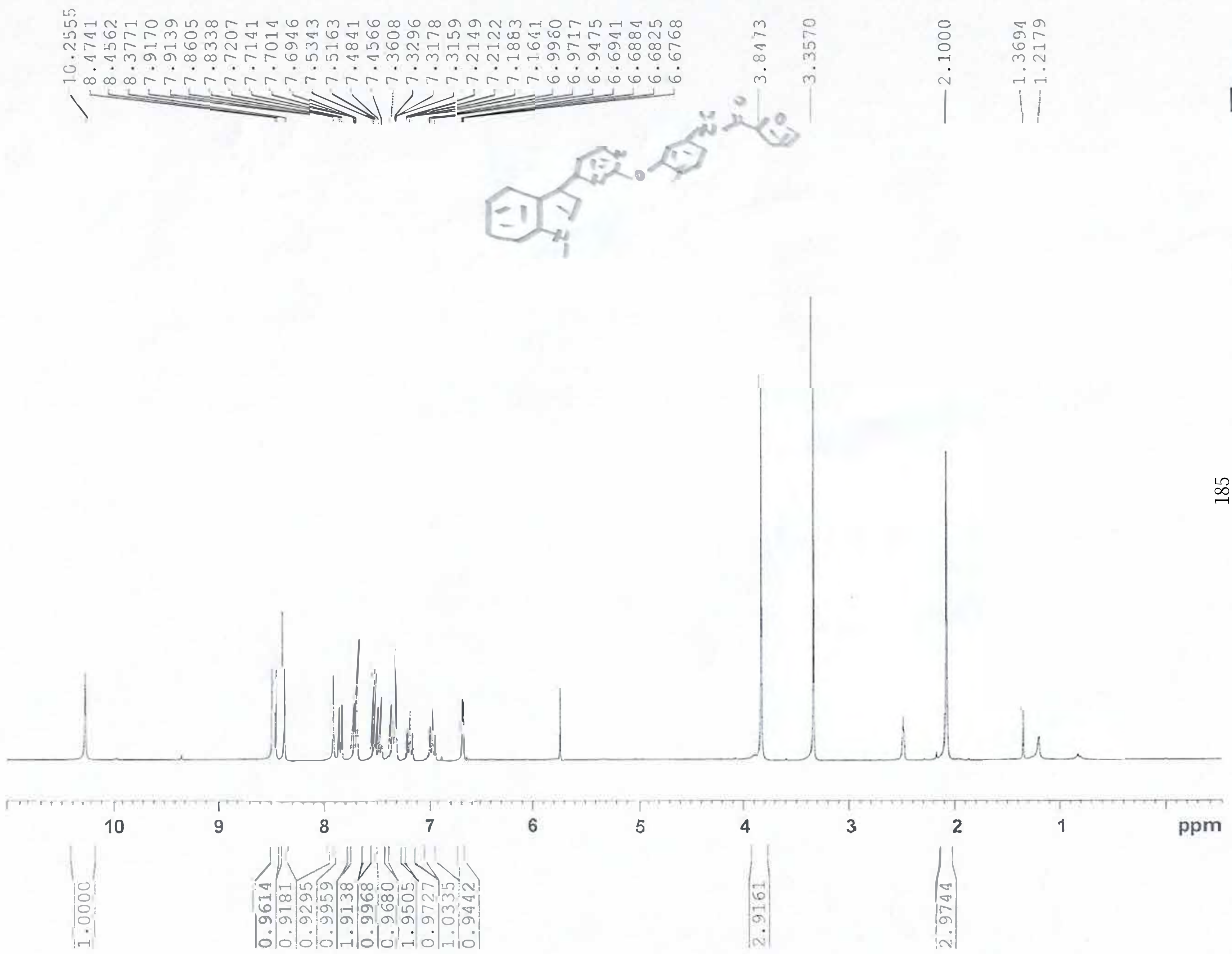

**Sample Name**		**Position**	P2a3	**Instrument Name**	Instrument 1
**User Name**	QTOF-PC\QTOF	**Inj Vol**	0.1	**InjPosition**	
**Sample Type**	Sample	**IRM Calibration Status**	Success	**Data Filename**	F3-p.d
**ACQ Method**	20110418-MSonly-p.m	**Comment**		**Acquired Time**	3/13/2018 1:46:49 PM

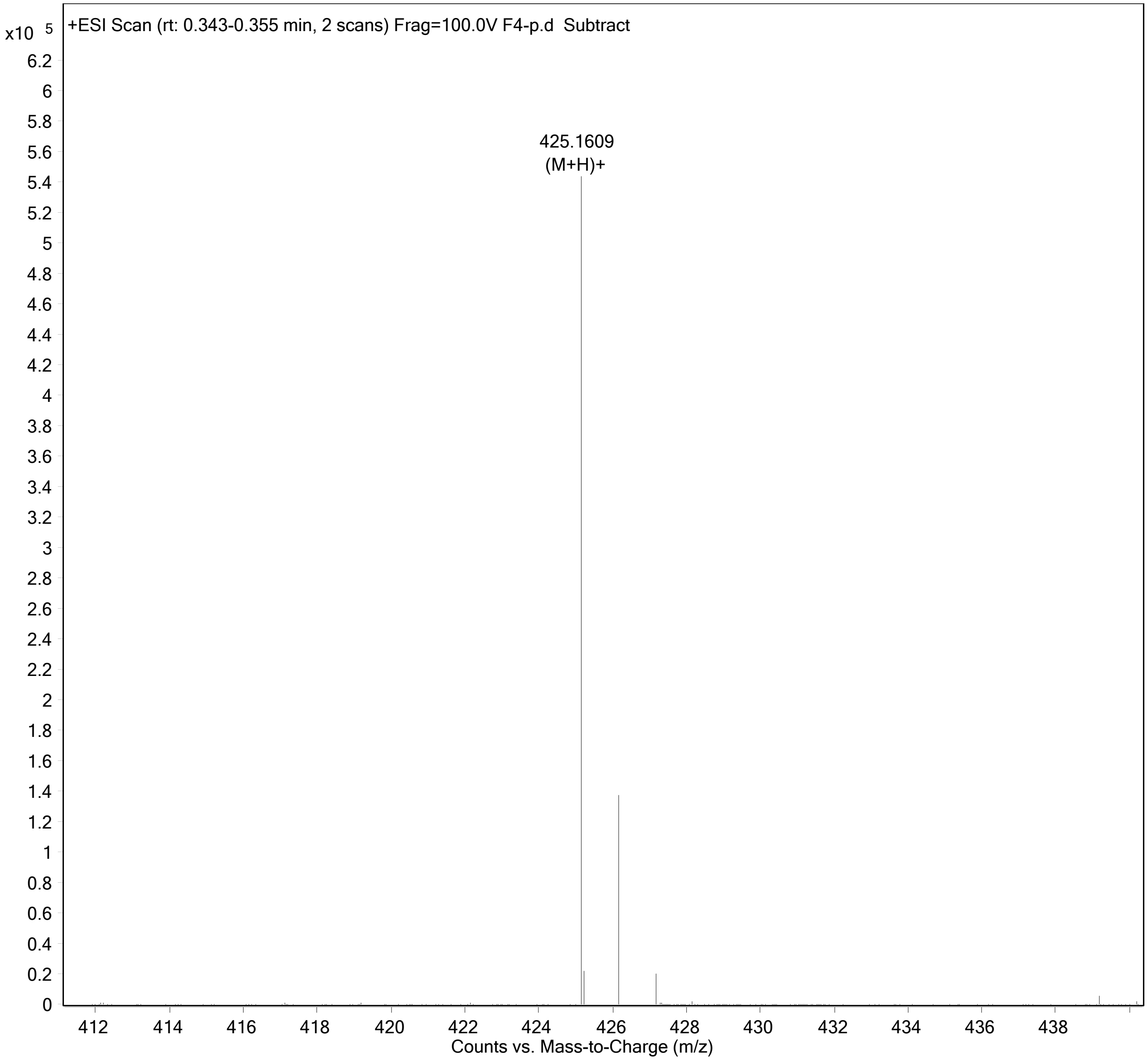

Supplement: Supplementary file 1 — Supplementary information [file 41598_2018_29199_MOESM1_ESM.pdf]
